# Immunologic signatures of response and resistance to nivolumab with ipilimumab in advanced metastatic cancer

**DOI:** 10.1084/jem.20240152

**Published:** 2024-08-27

**Authors:** Apostolia M. Tsimberidou, Farah A. Alayli, Kwame Okrah, Alexandra Drakaki, Danny N. Khalil, Shivaani Kummar, Saad A. Khan, F. Stephen Hodi, David Y. Oh, Christopher R. Cabanski, Shikha Gautam, Stefanie L. Meier, Meelad Amouzgar, Shannon M. Pfeiffer, Robin Kageyama, EnJun Yang, Marko Spasic, Michael T. Tetzlaff, Wai Chin Foo, Travis J. Hollmann, Yanyun Li, Matthew Adamow, Phillip Wong, Jonni S. Moore, Sharlene Velichko, Richard O. Chen, Dinesh Kumar, Samantha Bucktrout, Ramy Ibrahim, Ute Dugan, Lisa Salvador, Vanessa M. Hubbard-Lucey, Jill O’Donnell-Tormey, Sandra Santulli-Marotto, Lisa H. Butterfield, Diane M. Da Silva, Justin Fairchild, Theresa M. LaVallee, Lacey J. Padrón, Padmanee Sharma

**Affiliations:** 1 Department of Investigational Cancer Therapeutics, The University of Texas MD Anderson Cancer Center, Houston, TX, USA; 2 Parker Institute for Cancer Immunotherapy, San Francisco, CA, USA; 3 University of California, Los Angeles, Los Angeles, CA, USA; 4 Memorial Sloan Kettering Cancer Center, New York, NY, USA; 5 Stanford University, Stanford, CA, USA; 6 Dana-Farber Cancer Institute, Boston, MA, USA; 7 Parker Institute for Cancer Immunotherapy, Dana-Farber Cancer Institute, Boston, MA, USA; 8 University of California, San Francisco, San Francisco, CA, USA; 9 Department of Pathology, The University of Texas MD Anderson Cancer Center, Houston, TX, USA; 10 University of Pennsylvania, Philadelphia, PA, USA; 11 Natera Inc., Austin, TX, USA; 12 Personalis Inc., Fremont, CA, USA; 13 Bristol Myers Squibb, New York, NY, USA; 14 Cancer Research Institute, New York, NY, USA; 15 Department of Immunology, The University of Texas MD Anderson Cancer Center, Houston, TX, USA; 16 James P. Allison Institute, The University of Texas MD Anderson Cancer Center, Houston, TX, USA; 17 Immunotherapy Platform, James P. Allison Institute, The University of Texas MD Anderson Cancer Center, Houston, TX, USA; 18 Department of Genitourinary Medical Oncology, The University of Texas MD Anderson Cancer Center, Houston, TX, USA

## Abstract

Identifying pan-tumor biomarkers that predict responses to immune checkpoint inhibitors (ICI) is critically needed. In the AMADEUS clinical trial (NCT03651271), patients with various advanced solid tumors were assessed for changes in intratumoral CD8 percentages and their response to ICI. Patients were grouped based on tumoral CD8 levels: those with CD8 <15% (CD8-low) received nivolumab (anti-PD-1) plus ipilimumab (anti-CTLA4) and those with CD8 ≥15% (CD8-high) received nivolumab monotherapy. 79 patients (72 CD8-low and 7 CD8-high) were treated. The disease control rate was 25.0% (18/72; 95% CI: 15.8–35.2) in CD8-low and 14.3% (1/7; 95% CI: 1.1–43.8) in CD8-high. Tumors from 35.9% (14/39; 95% CI: 21.8–51.4) of patients converted from CD8 <15% pretreatment to ≥15% after treatment. Multiomic analyses showed that CD8-low responders had an inflammatory tumor microenvironment pretreatment, enhanced by an influx of CD8 T cells, CD4 T cells, B cells, and macrophages upon treatment. These findings reveal crucial pan-cancer immunological features for ICI response in patients with metastatic disease.

## Teaser

The AMADEUS trial reveals key biomarkers in blood and tumor tissue that could predict patient response to immunotherapy, offering a potential breakthrough for treating various solid tumors. This discovery addresses the pressing need for personalized cancer treatment strategies.

## Introduction

Immune checkpoint inhibitors (ICI), such as those targeting PD-1 (Cercek et al., 2022), PD-L1 (Powles et al., 2014), CTLA-4 (Hodi et al., 2010), and LAG3 (Tawbi et al., 2022)>, have revolutionized the landscape of cancer treatment by offering durable responses and even cures for some patients. However, many solid tumor types have limited response to ICIs (Chen and Mellman, 2017). It is imperative to understand the dynamics of tumors pre- and post-ICI treatment to elucidate resistance mechanisms, discover new therapies, and improve patient selection.

Tumor responsiveness to ICI often stems from its immune cell composition rather than its histology alone (Sharma et al., 2021). Tumor-agnostic biomarkers, including tumor mutational burden (TMB-high) (Rizvi et al., 2015)>, mismatch repair status (Marabelle et al., 2020), and PD-L1 expression (Herbst et al., 2016; Kowanetz et al., 2018) have emerged to identify “hot” tumors likely to benefit from anti-PD-1 treatment. These biomarkers often correlate with high tumoral CD8 T cell infiltrate (Maby et al., 2015; Thompson et al., 2017), an indicator of positive response to anti-PD-1 blockade. Additionally, posttreatment increases in tumoral CD8 cells are also associated with improved clinical outcomes (Chen et al., 2016; Ferris et al., 2019), highlighting its potential as a biomarker for hot tumors likely to respond to anti-PD-1 treatment.

PD-1 blockade has been shown to reinvigorate and expand exhausted tumor-reactive PD-1^+^ CD8 T cells and CTLA-4 blockade to promote T cell priming, clonal expansion, and CD4 and CD8 T cell trafficking into immunologically cold tumors (Kvistborg et al., 2014; Wei et al., 2018). However, this increase in tumoral T cell infiltrate, observed following CTLA-4 blockade, is accompanied by an upregulation of PD-(L)-1, which in turn can suppress T cell responses (Gao et al., 2017). Therefore, an increased tumoral T cell infiltration alone may be insufficient to confer antitumor responses, and anti-PD-1 and anti-CTLA4 combination treatment could result in improved responses in cold tumors (with low CD8). However, while combination anti-PD-1/anti-CTLA-4 treatment has shown clinical efficacy, it is often accompanied by increased toxicity (Subudhi et al., 2016; Wolchok et al., 2010), and this combination may be excessive for patients responsive to anti-PD-1 monotherapy.

To investigate this, we designed a multicenter, open-label study across tumor types to evaluate (1) the effectiveness of anti-PD-1 monotherapy in patients with CD8-high tumors and (2) the capacity of combined anti-PD-1/anti-CTLA-4 therapy to bolster CD8 T cell infiltration and elicit response in patients with CD8-low tumors. Through extensive multiomic profiling of tumor and blood samples pre- and on-treatment, this study aimed to identify pan-tumor biomarkers of response and resistance. Our findings provide insights into designing more effective tumor-agnostic patient stratification strategies and ICI treatments for the future.

## Results

### Trial design and patient characteristics

**Figure 1.**
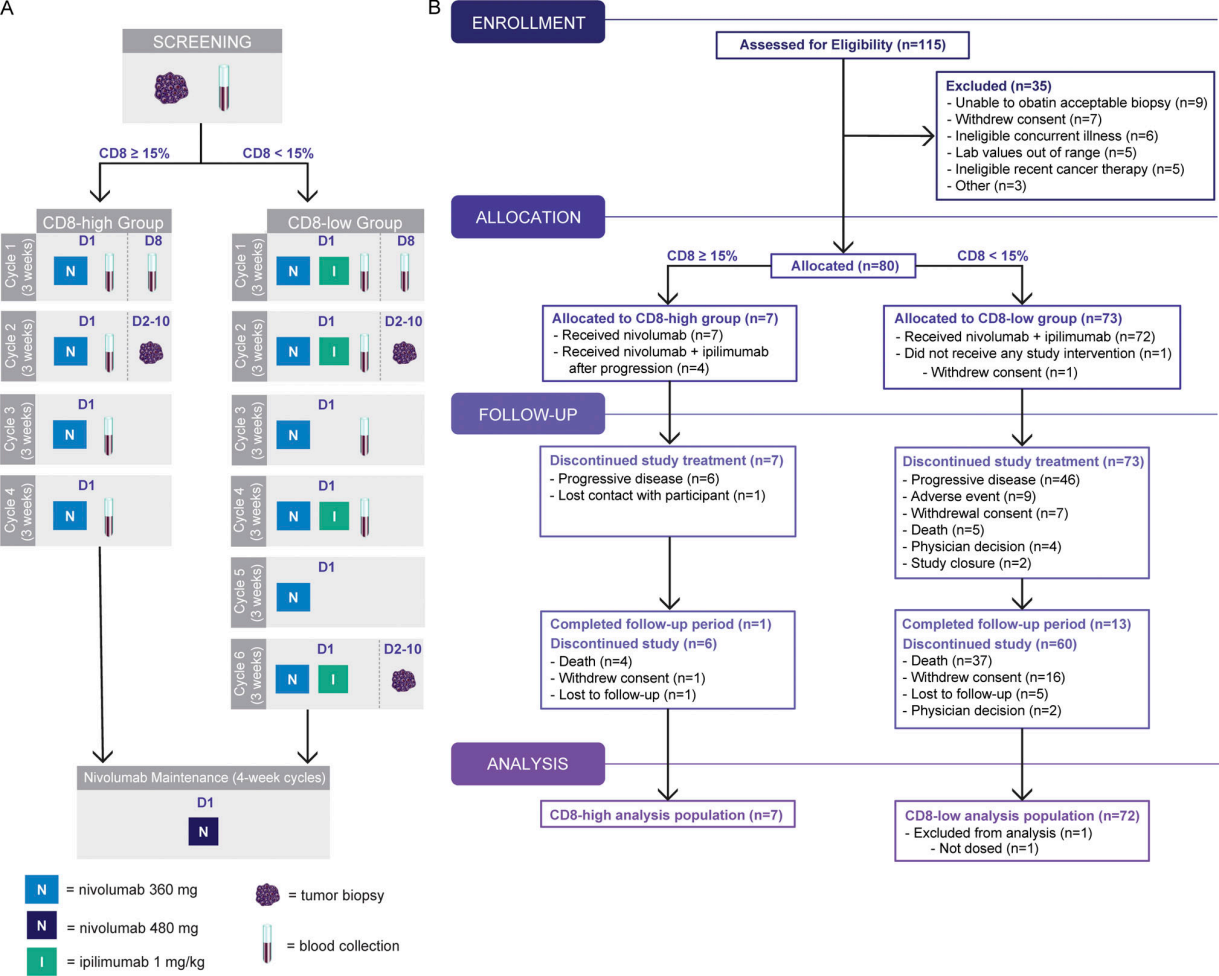
**AMADEUS study design and CONSORT (Consolidated Standards of Reporting Trials) diagram. (A)** AMADEUS was a clinical study that prospectively defined and assessed hot versus cold tumors using pretreatment percentages of CD8 cells. Patients in the CD8-high group received nivolumab monotherapy and those in the CD8-low group received nivolumab and ipilimumab combination. After completing four cycles in the CD8-high group or six cycles in the CD8-low group, patients continued to receive maintenance nivolumab. Tumor biopsies were mandatory at screening. Biopsies were also obtained Cycle 2 (both groups), Cycle 6 (CD8-low group), and at disease progression (CD8-high group), if medically feasible. Blood samples for translational analysis were collected at screening, Cycles 1–4, and the end of treatment visit. **(B)** CONSORT flow diagram. D = day.

Patients with metastatic cancer were assigned to receive nivolumab monotherapy or combination nivolumab and ipilimumab based on a cutoff of 15% tumoral CD8 T cells at screening. To ensure a reasonable likelihood of response to nivolumab monotherapy among CD8-high patients, while also avoiding high cutoffs that would hinder enrollment of such patients, we established a 15% threshold based on insights gained from unpublished retrospective data from multiple nivolumab studies (see Protocol). From November 5, 2018, through April 10, 2020, 79 patients were enrolled: 72 with low tumoral CD8 T cells (<15%) and 7 with high tumoral CD8 T cells (≥15%). Patients in the CD8-low group received nivolumab and ipilimumab combination treatment and patients in the CD8-high group (≥15%) received nivolumab monotherapy (Fig. 1). Efficacy and safety were assessed on the 79 patients who received at least one dose of study intervention. The cutoff date for data analysis was January 5, 2023.

The most common tumor types enrolled were prostate cancer (17%), colorectal cancer (10%), sarcoma (10%), head and neck cancer (8%), and ovarian cancer (7%) in the CD8-low group, and head and neck cancer in three out of seven (43%) patients in the CD8-high group (Table 1). In general, patients were heavily pretreated, having received a median of four prior lines of treatment. 15 (21%) patients in the CD8-low group and three (43%) in the CD8-high group received prior ICI; however, ICI as the most recent line of therapy prior to screening was an exclusion criterion. Patient demographic and baseline characteristics are provided in Table 1. Prior systemic therapies are summarized in Table S1.

**Table 1.** Demographic and baseline disease characteristics

	CD8-high (*N* = 7)	CD8-low (*N* = 72)	Total (*N* = 79)
Characteristic			
Age, years			
Median (range)	54.0 (39–70)	60.5 (32–80)	60.0 (32–80)
>65 years, *n* (%)	1 (14)	26 (36)	27 (34)
Sex, *n* (%)			
Female	1 (14)	35 (49)	36 (46)
Male	6 (86)	37 (51)	43 (54)
Race, *n* (%)			
Asian	0	5 (7)	5 (6)
Black	0	7 (10)	7 (9)
White	6 (86)	45 (63)	51 (65)
Other	1 (14)	15 (21)	16 (20)
Ethnicity, *n* (%)			
Hispanic	0	9 (13)	9 (12)
ECOG performance status at screening, *n* (%)			
0	4 (57)	23 (32)	27 (34)
1	3 (43)	47 (65)	50 (63)
Missing	0	2 (3)	2 (3)
CD8 Cells (%) at screening			
Median (range)	22.0 (16–36)	4.0 (0–14)	5.0 (0–36)
Tumor type			
Prostate (CRPC)	0	12 (17)	12 (15)
Head and Neck (HNCA)	3 (43)	6 (8)	9 (11)
Colorectal (CRCA)	0	7 (10)	7 (9)
Sarcoma (SARC)	0	7 (10)	7 (9)
Ovarian (OVCA)	1 (14)	5 (7)	6 (8)
Uterine (UTCA)	0	4 (6)	4 (5)
Breast (BRCA)	0	3 (4)	3 (4)
Hepatocellular cholangiocarcinoma (HECH)	0	3 (4)	3 (4)
Neuroendocrine (NEUC)	0	3 (4)	3 (4)
Renal (RNCA)	1 (14)	2 (3)	3 (4)
Thyroid (THYR)	0	3 (4)	3 (4)
Other^a^	2 (29)	17 (24)	19 (24)
Prior lines of cancer therapy			
0	0	3 (4)	3 (4)
1–2	3 (43)	18 (25)	21 (27)
3–4	3 (43)	19 (26)	22 (28)
5+	1 (14)	32 (44)	33 (42)
Received prior ICI therapy, *n* (%)	3 (43)	15 (21)	18 (23)

a Other tumor types include urethral (URET) and gastroesophageal (GEJC) (*n* = 1 each; 14%) in the CD8-high group and cervix (CVCX), gastric (GSCA), non-small cell lung (LUCA), pancreatic (PANC), pelvic (PELV), peritoneal (PRTC) (*n* = 2 each, 3%), hepatocellular carcinoma (HCCA), Merkel cell (NESK), penile (PENC), retroperitoneal teratoma (TERA), and papilla of vater (AMPV) (*n* = 1 each; 1%) in the CD8-low group.

At the time of analysis, all patients had discontinued treatment and follow-up. The median duration of follow-up was 26.9 mo (interquartile range [IQR] 5.8–30.1) and the minimum follow-up was 22 mo. The median time on treatment was 1.4 (IQR: 0.7–4.4) and 3.8 (3.5–5.7) months for the CD8-low and CD8-high groups, respectively (Table S2).

### Clinical activity

The coprimary endpoints were (1) disease control rate (DCR), defined as the proportion of patients with the best overall response of complete response (CR), partial response (PR), or stable disease (SD) lasting at least 24 wk, and (2) the proportion of patients in the CD8-low group whose tumors converted from CD8-low (<15%) to CD8-high (≥15%). Secondary endpoints included objective response rate (ORR), progression-free survival (PFS), overall survival (OS), and the association of CD8 percentage with clinical outcomes.

**Figure 2.**
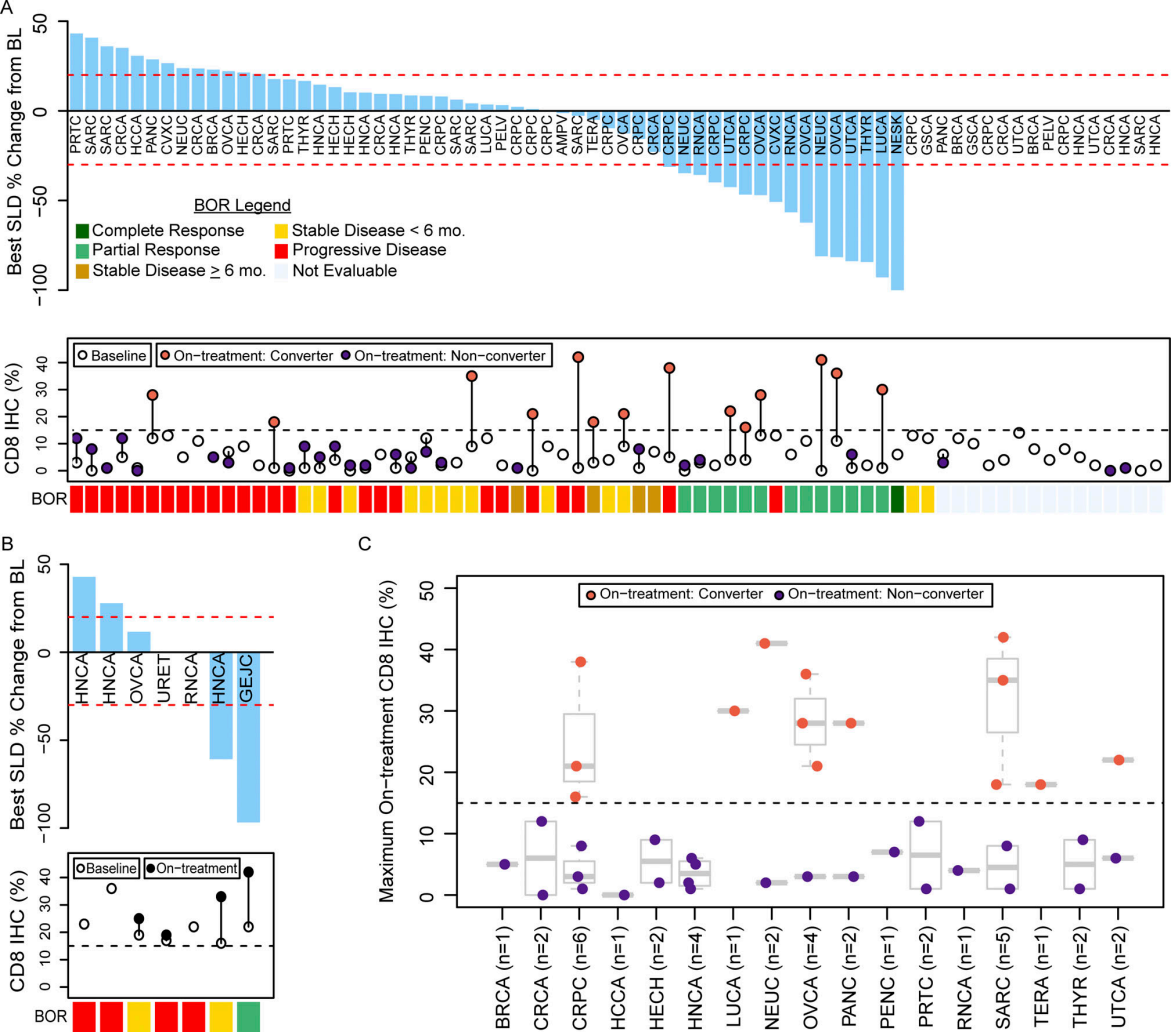
**Tumor response and change in tumoral percentage of CD8 T cells. (A and B)** Tumor type and maximum percentage change from baseline in the sum of the longest diameters of the target lesions (top) along with pretreatment (empty circles) and on-treatment (colored circles) tumoral CD8 IHC percentage (bottom) for each patient in the (A) CD8-low and (B) CD8-high groups. If a patient had multiple on-treatment biopsies, the largest on-treatment CD8 percentage is plotted. 14 of 39 patients in the CD8-low group with an on-treatment biopsy had tumors that converted from CD8-low (<15%) to CD8-high (≥15%; orange circles). **(C)** Maximum on-treatment CD8 percentage for patients in the CD8-low group, presented by tumor type. Box plots show the median and quartiles, and whiskers represent 1.5 times the interquartile range. Tumor-type abbreviations are defined in Table 1. BL = baseline/pretreatment; BOR = best overall response; SLD = sum of longest diameters.

In the CD8-low group, the DCR was 25.0% (18/72; 95% credible interval [CI]: 15.8–35.2) and the ORR was 19.4% (14/72; 95% CI: 11.3–29.1) (Fig. 2 A and Table S3). 39 (54%) CD8-low patients had an on-treatment biopsy. Patients who underwent an on-treatment biopsy generally had lower baseline tumoral CD8 levels, were less likely to have prior ICI exposure, and exhibited more favorable outcomes compared to patients without an on-treatment biopsy (Table S4). Of these 39 patients, 14 (35.9%, 95% CI: 21.8–51.4) had tumors that converted from CD8-low to CD8-high. The median change in CD8 percentage from baseline to on-treatment was 5.0 (range: −5 to 41). Baseline CD8 percentage was not significantly associated with ORR (P = 0.676) or DCR (P = 0.375). However, CD8 conversion (shift from CD8-low to CD8-high) was associated with ORR (P = 0.037) and DCR (P = 0.058). Similar, albeit slightly weaker, trends were observed when associating clinical response with the on-treatment CD8 percentage and with the change between baseline and on-treatment biopsies (Table S5). The median OS and PFS were 13.9 mo (95% CI: 8.9–21.1) and 2.3 mo (95% CI: 2.0–4.3), respectively.

In the CD8-high group, the DCR and the ORR were both 14.3% (1/7; 95% CI: 1.1–43.8) (Fig. 2 B). Of the four (57%) CD8-high patients with on-treatment biopsies, the median change in CD8 percentage from baseline to on-treatment was 11.5 (range: 2–20). Due to the small sample size, the association between tumoral CD8 levels and clinical response was not evaluated. The median OS and PFS were 15.8 mo (95% CI: 12.1—not estimable) and 2.0 mo (95% CI: 1.1—not estimable), respectively.

To further understand associations of CD8 with clinical benefit, we performed the following post-hoc analyses. First, patients who received prior ICI treatment had lower rates of response and CD8 conversion, although these differences were not statistically significant (Table S6). Second, in the CD8-low group, clinical response and CD8 conversion rates differed by tumor type. The highest rates of DCR and CD8 conversion were observed in prostate, ovarian, uterine, and neuroendocrine tumors (Fig. 2 C and Table S7).

### Safety

The spectrum, frequency, and severity of treatment-related adverse events (TRAEs), a secondary endpoint, and immune-related adverse events (IRAEs) were similar to the reported safety profiles of nivolumab and ipilimumab (Sznol et al., 2017). Overall, 62 (79%) patients reported at least one TRAE, including 20 (25%) who experienced grade 3/4 TRAEs. The most common TRAEs of any grade were fatigue, diarrhea, nausea, pruritus, and rash (Table S8).

IRAEs were observed in 42 (58%) patients in the nivolumab and ipilimumab group and two (29%) patients in the nivolumab group (Table S9). All IRAEs were grade 1–3. The most common IRAEs in the nivolumab and ipilimumab group were diarrhea (21%), pruritus (15%), and rash (15%); no IRAE was observed in more than one patient in the nivolumab group.

Three grade 5 AEs occurred in the nivolumab and ipilimumab group: cardio-respiratory arrest, myocardial infarction, and small intestinal obstruction. All were assessed as unrelated to study treatment. In the nivolumab and ipilimumab group, nine (13%) patients discontinued study treatment owing to an AE, all of which were grade 2–3 (Table S10). No treatment discontinuation or death owing to an AE was observed in the nivolumab group.

### Exploratory biomarker analysis of CD8-low tumors

To evaluate whether characteristics in the tumor tissue or the blood, agnostic of tumor type, correlate with clinical response and tumoral CD8 conversion, we performed comprehensive multiomic analyses on pre- and on-treatment tumor and blood samples. Tumor tissue analysis included bulk RNA sequencing (RNAseq), whole exome sequencing (WES), and multiplex immunofluorescence (mIF) imaging. Peripheral blood mononuclear cell (PBMC) analysis included high dimensional flow cytometry analysis (X50), broad immune profiling using Cytometry by Time of Flight (CyTOF), cellular indexing of transcriptomes and epitomes by sequencing (CITEseq), and single-cell T cell receptor (TCR) sequencing. Serum and plasma analysis included proteomics and circulating tumor DNA (ctDNA) quantification. Given the limited sample size of the CD8-high group, we present translational results solely for the CD8-low group.

### Pretreatment tumor inflammatory gene signatures associated with response

**Figure 3.**
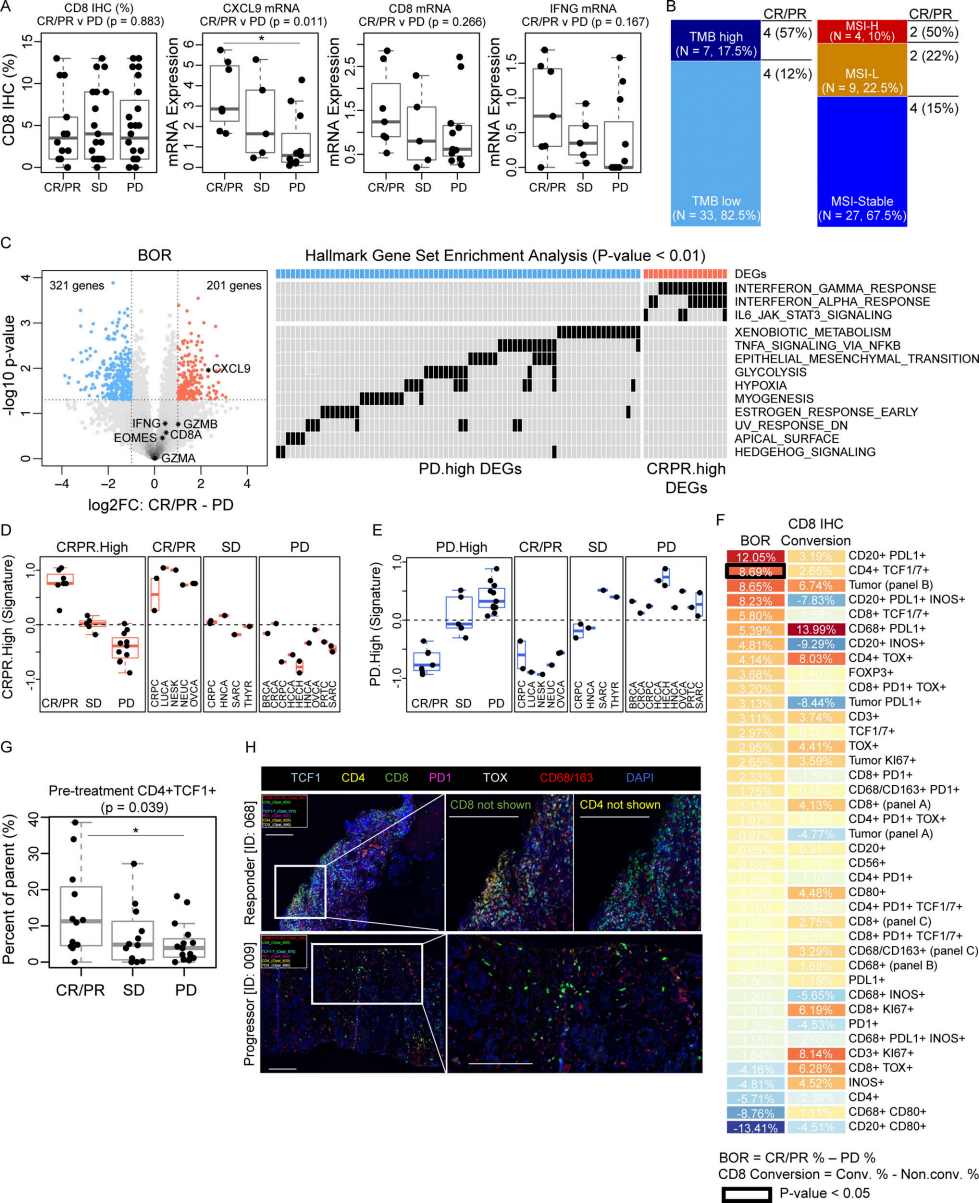
**Pretreatment tumor inflammatory gene expression and mIF imaging. (A)** Box plots of pretreatment CD8 IHC (%) and mRNA expression levels of *CXCL9*, *CD8A*, and *IFNG* genes grouped by best overall response (BOR). Expression levels were compared between responders (CR/PR, *n* = 7) and progressors (PD, *n* = 11) by the student’s *T* test. **(B)** Bar plots of TMB (left) and MSI (right) status in pretreatment biopsies. **(C)** Volcano plot of the DEGs in pretreatment tumor biopsies between responders (*n* = 7) versus progressors (*n* = 11) and Hallmark GSEA indicating the DEGs contributing to significantly enriched pathways (P < 0.01). The heatmap only displays DEGs within these pathways. **(D and E)** Box plots of the aggregate gene expression (signature) of the CRPR.high and PD.high DEGs plotted by tumor type. **(F)** Heatmap displaying mean differences in cell populations detected by mIF (Vectra; scale bar, 200 μm) imaging of pretreatment tumor samples, comparisons done by BOR: CR/PR (%, *n* = 14) − PD (%, *n* = 21) and CD8 conversion: converter (%, *n* = 13) − non-converter (%, *n* = 22). Markers featured in multiple panels are denoted with the respective panel label. **(G)** Box plots of tumoral TCF1^+^ CD4 T cells (%) grouped by BOR. **(H)** Representative ROI images from two patients with pretreatment tumor biopsies probed with antibodies from mIF (panel C). Top: Representative ROI from tumor tissue from an ovarian cancer patient (ID: 068) with BOR: PR, biopsy location: lymph node. Bottom: Representative ROI from a breast cancer patient (ID: 009) with BOR: PD, biopsy location: liver. Box plots show median and quartiles, and whiskers represent 1.5 times the IQR. Tumor-type abbreviations are defined in Table 1. *P < 0.05 by student’s *T* test (A, F, and G).

In pretreatment tumor tissue, response to nivolumab and ipilimumab correlated with higher messenger RNA (mRNA) expression of CXCL9 (P = 0.011) (Fig. 3 A), consistent with reported results in multiple tumor types (Litchfield et al., 2021). Additionally, non-significant trends showed higher mRNA expression levels of CD8A (P = 0.266) and IFN-γ (P = 0.266) in responders’ tumors relative to progressors. The prevalence of TMB-high, defined as ≥10 mutations per megabase, was 17.5% (7/40) and microsatellite instability (MSI)-high was 10% (4/40) with response rates of 57% (4/7; P = 0.020) and 50% (2/4; P = 0.172), respectively (Fig. 3 B).

**Figure S1.**
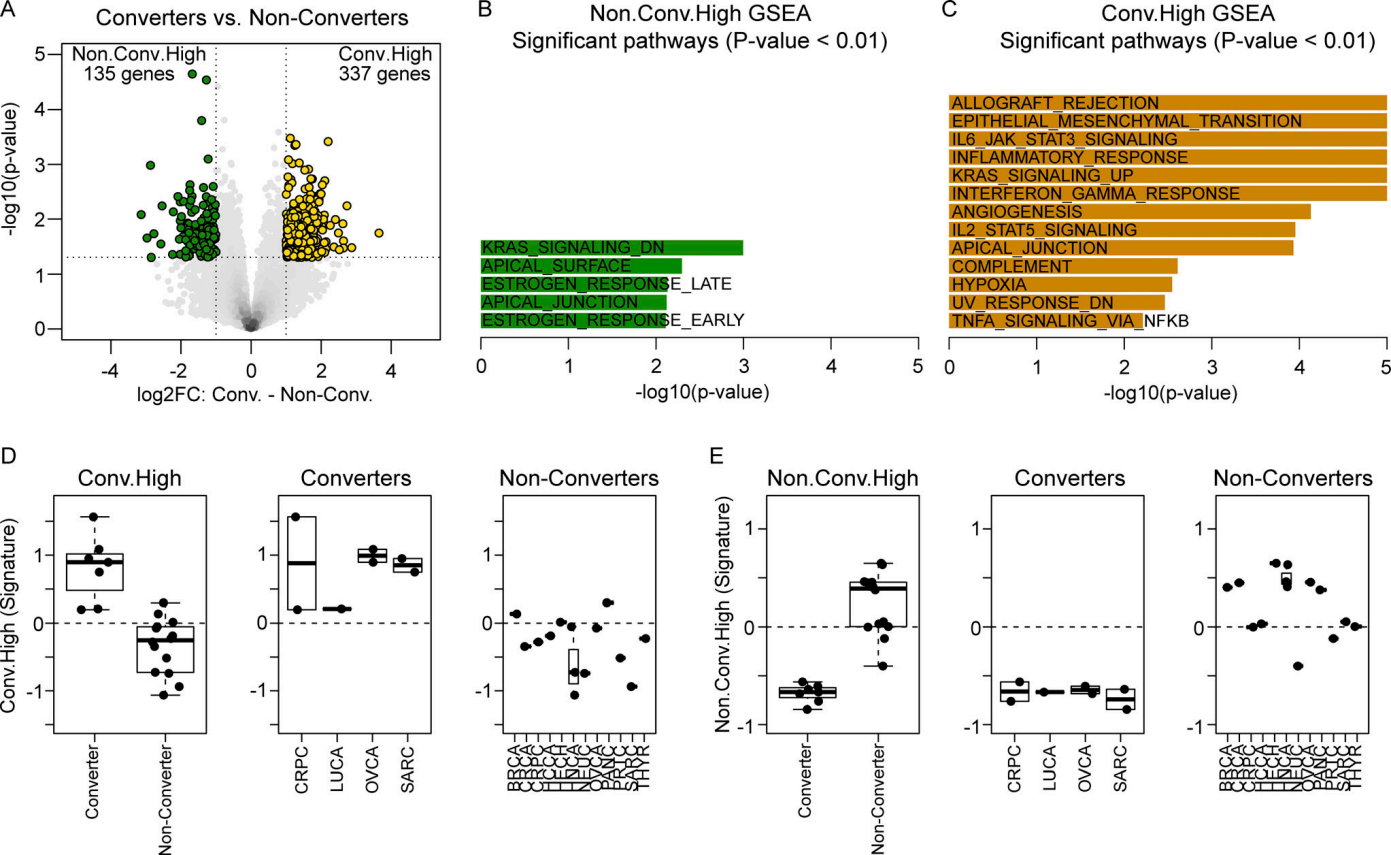
**Differential gene expression analysis on pretreatment tumor samples comparing CD8 converters versus non-converters in the CD8-low group. (A)** Volcano plot of the DEGs when comparing pretreatment tumor samples from CD8 converters (*n* = 7) versus CD8 non-converters (*n* = 14). **(B and C)** Hallmark GSEA results indicating pathways with genes overrepresented in the Non-converter.High DEGs and the Converter.High DEGs. **(D and E)** Box plots of aggregated gene expression (signature) of the DEGs plotted by tumor type. Box plots show median and quartiles and whiskers represent 1.5× IQR. Tumor-type abbreviations are defined in Table 1. FC = fold change.

Next, we investigated which genes from pretreatment tumor tissue were differentially expressed between responders and non-responders. We identified 522 differentially expressed genes (DEGs): 201 higher in responders (CR/PR.High gene set) and 321 higher in progressors (PD.High gene set; Fig. 3 C). Gene set enrichment analysis (GSEA) revealed that responders’ tumors contained an overrepresentation of genes in inflammatory pathways including IFN-γ and -α responses and IL-6/JAK/STAT3 signaling while progressors’ tumors mainly expressed genes in pathways such as xenobiotic metabolism and epithelial–mesenchymal transition. These gene signatures were consistent across different tumor types (Fig. 3, D and E). When comparing CD8 converters to non-converters, differential expression analysis (DEA) yielded 337 genes higher in converters’ tumors and 135 genes higher in non-converters’ tumors (Fig. S1 A). GSEA revealed that the converters’ tumors, similar to responders’ tumors, expressed genes relating to inflammatory pathways including IFN-γ response and IL-6/JAK/STAT3 signaling. However, other pathways typically associated with non-response to ICI (Chen et al., 2016; Lei et al., 2020), including angiogenesis, hypoxia, and epithelial-mesenchymal transition, were also represented in the converters’ gene set. In contrast, the non-converters’ gene set contained genes in pathways such as apical junction and Kristen rat sarcoma viral oncogene homolog (KRAS) downregulation (Fig. S1, B and C). The aggregate gene sets identified from DEA by conversion also did not vary by tumor type (Fig. S1, D and E). Altogether, these data indicate that although all patients in this group had low tumoral CD8 infiltrate prior to treatment, an existing inflammatory tumor microenvironment (TME) is nevertheless present in patients who are more likely to respond to nivolumab and ipilimumab.

mIF analysis on pretreatment tumor biopsies echoed the RNAseq findings. Specifically, responders’ tumors exhibited a pre-existing inflammatory TME (Fig. 3 F) and had a higher frequency of stem cell progenitor-like (TCF1^+^) CD4 T cells (P = 0.039) (Fig. 3 G). Moreover, many of these T cells also co-expressed PD-1 (Fig. 3 H). Recent studies have shown that the presence of tumor-residing stem cell–like T cells is predictive of response (Peng et al., 2021; Rong et al., 2022; Sade-Feldman et al., 2018), suggesting the potential of stem cell–like CD4 T cells as a pan-cancer predictive biomarker for treatment response.

### Higher pretreatment abundance of circulating biomarkers in progressors

**Figure 4.**
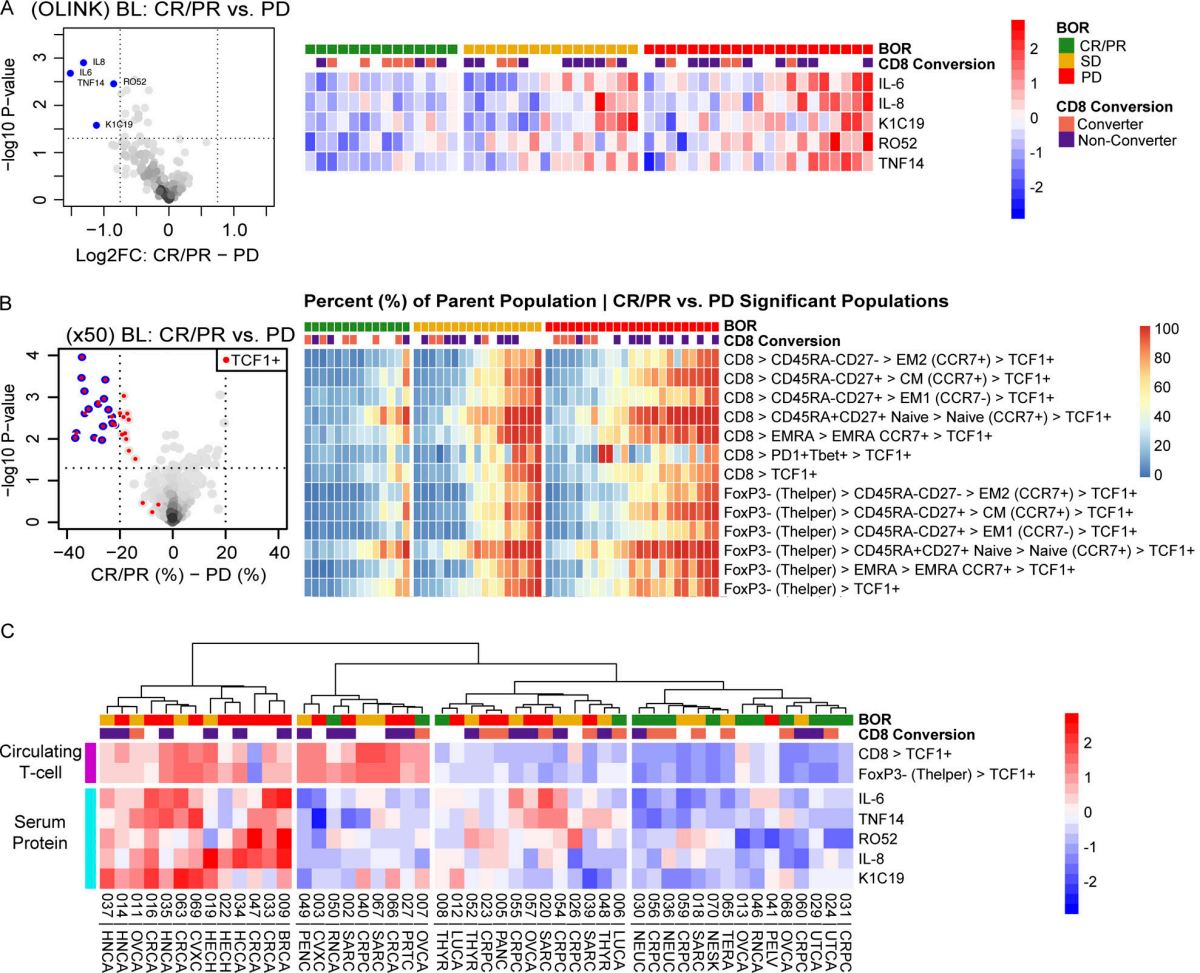
**Pretreatment peripheral blood-based biomarkers associated with progression. (A)** Left: Volcano plot of serum cytokines comparing responders (CR/PR, *n* = 14) to progressors (PD, *n* = 21); statistically significant cytokines highlighted in blue. Right: Heatmap of patients sorted by response and the aggregate z-scores of IL-6, IL-8, K1C19, RO52, and TNF14. **(B)** Left: Volcano plot of the X50 gated T cell subsets from pretreatment PBMCs comparing responders (CR/PR, *n* = 14) to progressors (PD, *n* = 23); statistically significant populations indicated in blue. Right: Heatmap of the percent of parent values of the significantly different T cell populations, sorted by response. **(C)** Heatmap of normalized OLINK and X50 expression of blood biomarkers associated with response. Tumor-type abbreviations are defined in Table 1. BL = baseline; BOR = best overall response; FC = fold change.

Proteomics analysis revealed a significantly higher abundance of IL-6, IL-8, K1C19, RO52, and TNF14 in progressors’ pretreatment serum (Fig. 4 A), consistent with prior studies (Laino et al., 2020; Sanmamed et al., 2017). Analysis using the X50 T cell panel revealed that progressors had significantly higher frequencies of circulating TCF1^+^ CD8^+^ and TCF1^+^ CD4^+^ T cells (P < 0.05) than responders (Fig. 4 B), which contrasts with our findings in the tumor tissue. Interestingly, the high frequency of circulating TCF1^+^ T cells pretreatment was not observed in the few progressors whose tumors converted from CD8-low to CD8-high on-treatment. Both findings from the blood were also correlated with each other and were agnostic of tumor type (Fig. 4 C).

### Higher circulating IFN-induced central memory CD4 T cells in responders

**Figure S2.**
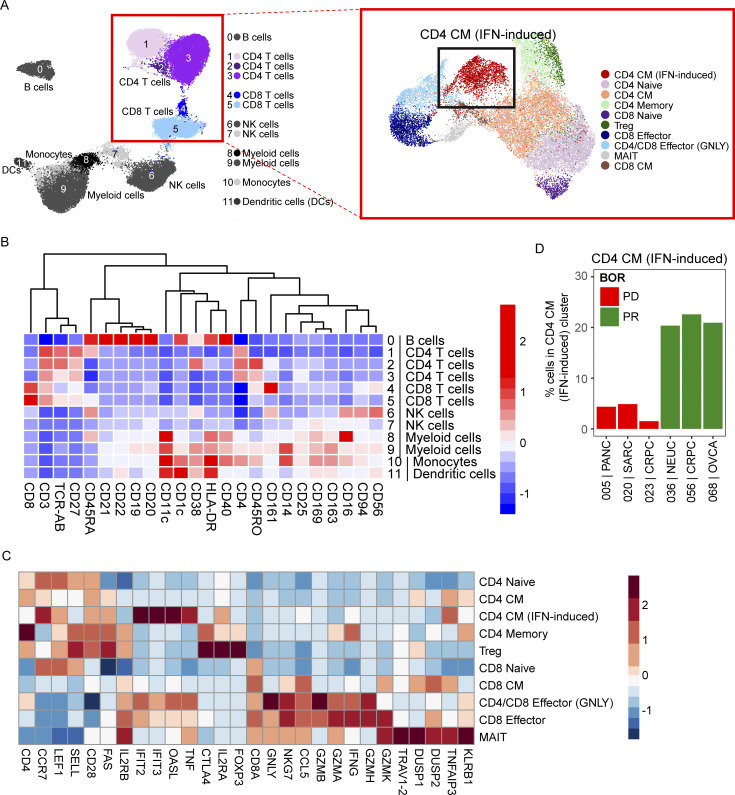
**Higher pretreatment frequencies of circulating IFN-induced central memory (CM) CD4 T cells are found in responders.** CITEseq analysis on pretreatment PBMCs from six patients (all CD8 converters: *n* = 3 partial responders, *n* = 3 progressors). **(A)** 10 T cell clusters identified using ADT and gene expression. **(B)** Gene expression profile of 12 cell clusters derived from ADTs. **(C)** Gene expression of T cell subset clusters. **(D)** Bar plots of the percentage of cells in the CD4 central memory cluster grouped by tumor type. Tumor-type abbreviations are defined in Table 1. BOR = best overall response; MAIT = mucosal-associated invariant T cells.

While CD8 conversion associated with clinical response, not all patients with conversion achieved radiographic response. Using CITEseq, which simultaneously analyzes transcriptional states and protein expression via antibody-derived tags (ADT), we analyzed pretreatment PBMCs from six patients (all CD8 converters, *n* = 3 responders, *n* = 3 progressors). Unsupervised clustering analysis using the ADT and gene expression data identified 10 unique T cell clusters (Fig. S2, A–C). The transcriptional analysis revealed a significantly higher frequency of circulating IFN-induced central memory CD4 T cells in responders (Fig. S2 D), further emphasizing the potential role of CD4 T cells not only in the tumor but also in the circulating T cell compartment in predicting nivolumab and ipilimumab response.

### Complementary biomarkers predictive of response to nivolumab and ipilimumab

**Figure 5.**
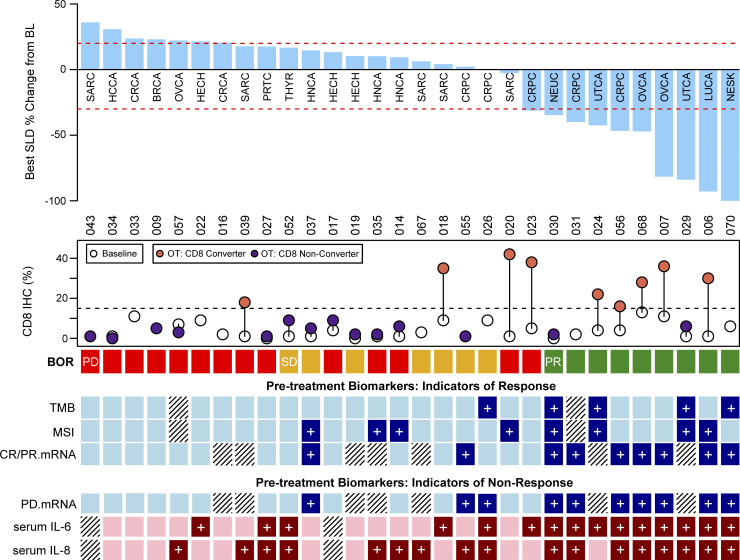
**Pretreatment biomarkers associated with response to nivolumab and ipilimumab.** Summary table of six biomarkers associated with response pretreatment (four from tumor tissue, colored blue: TMB, MSI, CR/PR.mRNA tumor gene expression signature, and PD.mRNA tumor gene expression signature; and two from blood, colored red: serum IL-6 and IL-8). Biomarker values associated with response are shown in a darker color with a “+”: TMB-high; MSI-high or MSI-low; CR/PR.mRNA expression above the median; PD.mRNA expression below the median; serum IL-6 expression below the median; and serum IL-8 expression below the median. Hatched lines indicate that biomarker data is not available. Data are shown for patients in the CD8-low group with at least four of the six biomarkers assessed. Tumor-type abbreviations are defined in Table 1. BL = baseline; BOR = best overall response; OT = on-treatment; SLD = sum of longest diameters.

Fig. 5 illustrates a manually curated set of pretreatment biomarkers from tumor tissue—specifically, TMB, MSI, and RNAseq genes sets characteristic of responders (CR/PR.High), and progressors (PD.High)—in addition to serum IL-6 and IL-8. These biomarkers are correlated with response to nivolumab and ipilimumab treatment in patients with CD8-low tumors. While each biomarker is associated with response, no single biomarker reliably distinguishes responders from non-responders across this pan-cancer study. The biomarkers appear to be complementary, suggesting a combined signature might enhance the prediction of treatment outcomes. Although the current study does not have the statistical power to build and validate such a composite biomarker, these findings underscore the promise of combining biomarkers from multiple data types to create a robust predictive signature.

### Higher on-treatment TME inflammatory signature in responders

**Figure S3.**
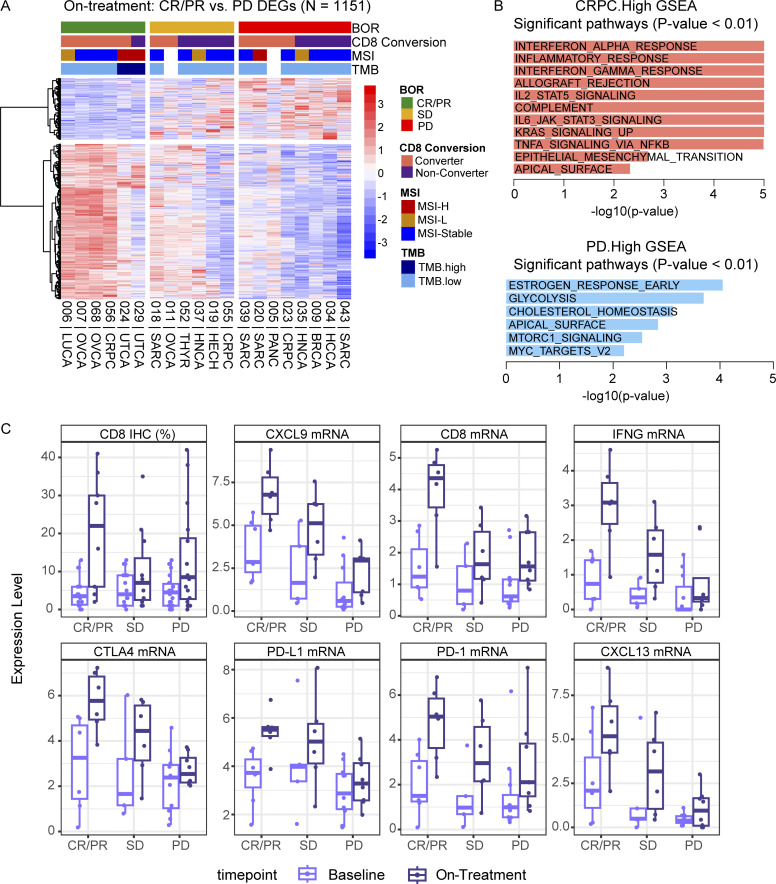
**On-treatment tumor inflammatory signatures associate with response to nivolumab and ipilimumab. (A)** Heatmap of DEGs from on-treatment tumor samples comparing responders (CR/PR, *n* = 6) to progressors (PD, *n* = 8). Patients (columns) are sorted by best overall response (BOR) and then CD8 conversion. **(B)** Hallmark GSEA of the statistically significant pathways (P < 0.01) for genes that are significantly higher in responders (CRPC.High) and progressors (PD.High). **(C)** Box plots of CD8 IHC (%) and mRNA expression levels of select genes, grouped by BOR. Box plots show median and quartiles and whiskers represent 1.5× IQR. Tumor-type abbreviations are defined in Table 1.

To evaluate the changes in the TME after nivolumab and ipilimumab treatment, RNAseq data from on-treatment tumor biopsies were analyzed. Analysis of gene expression in responders’ tumors (*n* = 6) compared with progressors (*n* = 8) revealed 1,151 DEGs (Fig. S3 A). Genes associated with proinflammatory immune responses, IFN-γ and -α pathways, and genes upregulated by KRAS activation were higher in responders’ tumors. On the other hand, genes associated with glycolysis and myleocytomatosis oncogene (MYC) targets were more frequent in progressors’ tumors (Fig. S3 B). Notably, responders’ tumors also had higher expression of CXCL9, CD8A, and IFN-γ, similar to the findings from pretreatment tissue (Fig. S3 C).

**Figure 6.**
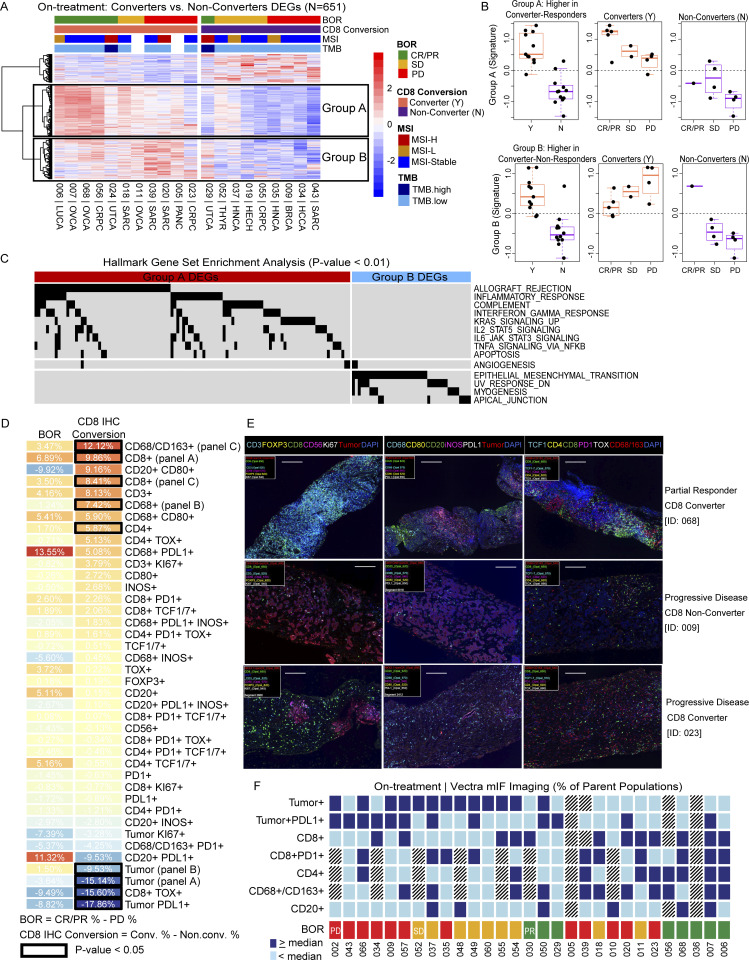
**On-treatment inflammatory TME and higher frequencies of circulating activated and proliferating T cells in responders. (A)** Heatmap of patients sorted by conversion then response with expression of the DEGs identified by comparing on-treatment tumor samples from CD8 converters to non-converters. Unsupervised clustering revealed three distinct signatures, including genes higher in responders who were CD8 converters (Group A) and genes higher in non-responders who were CD8 converters (Group B). The complete list of genes displayed in this figure is available as Table S15. **(B)** Box plots of Group A and Group B gene signatures by best overall response (BOR) and CD8 conversion (Y = converter, N = non-converter). **(C)** GSEA pathways enriched in Groups A and B. **(D)** Heatmap displaying mean differences in cell populations detected by mIF imaging (Vectra) of on-treatment tumor samples, comparisons done by BOR: CR/PR (%, *n* = 7) − PD (%, *n* = 12) and CD8 conversion: converter (%, *n* = 10) − non-converter (%, *n* = 21) by the student’s *T* test; significantly different populations are denoted by a black outline. Markers featured in multiple panels are denoted with the respective panel label. **(E)** Representative ROI images at 200× final magnification from three patients with on-treatment tumor biopsies from three mIF panels; left: panel A, middle: panel B, right: panel C (scale bar, 200 μm). **(F)** Selected mIF imaging results show the combination of cell types that are present (≥ median), absent (< median), or not evaluable (grey) for each patient with on-treatment mIF data. Box plots show median and quartiles, and whiskers represent 1.5 times the IQR. Tumor-type abbreviations are defined in Table 1.

When comparing converters’ and non-converters’ tumors on-treatment, 651 DEGs were identified (Fig. 6 A and Table S15). Generally, gene sets from non-converters, like progressors, were dominated by pathways such as glycolysis, MYC targets, and estrogen response. Using unsupervised clustering, genes that were higher in converters were divided into two distinct gene groups: Group A, consisting of genes that were expressed higher in responders who were also CD8 converters, and Group B, consisting of genes that were overexpressed in non-responders (SD/PD) who were CD8 converters (Fig. 6 B). Gene Groups A and B both showed higher abundance of genes related to inflammatory response pathways, albeit Group A to a much higher extent (Log_2_ fold change >0.5) (Fig. 6 C). Gene Group B additionally showed higher abundance of gene pathways associated with lack of response to PD-1 treatment such as epithelial–mesenchymal transition and myogenesis, which included collagen genes (*COL1A1*, *COL6A2*, *COL11A1*, *COL12A1*, *LOX*, *LOXL1*, *SERPINH1*) as well as *GPC1*, and *TGFB3* (full list of genes available as Table S15). The on-treatment gene set from converters/non-responders (Group B) suggests the presence of additional TME factors that are inhibiting the effector function of the intratumoral T cells. Overall, this analysis identifies pathways that can be targeted in combination with nivolumab and ipilimumab to overcome ICI resistance.

### Increased tumoral CD8 T cells necessary but insufficient for response

Consistent with our RNAseq findings, tumor imaging (mIF) results on-treatment demonstrate that, in addition to an increase in tumoral CD8 T cells, tumors classified as CD8 converters also had a significantly higher frequency of macrophages and CD4 T cells compared to non-converters on-treatment (Fig. 6, D and E). In contrast, non-converters’ tumors had a higher abundance of tumor cells and tumor cells expressing PD-L1, and more CD8 T cells expressing thymocyte selection-associated high mobility group box (TOX) protein, a marker of T cell exhaustion (Beltra et al., 2020; Scott et al., 2019). Analysis of the cell populations by response showed a higher presence of macrophages and B cells in responders’ tumors and higher abundance of tumor cells in progressors, albeit the differences were not significant. Representative mIF images from three on-treatment tumor biopsies with varying clinical responses and CD8 conversion status demonstrate some of these immune cell infiltrate differences (Fig. 6 E). Interestingly, mIF images from on-treatment tumor biopsies of two progressors with CD8 conversion (sarcoma [ID: 020] and prostate [ID: 023]) revealed an influx of CD8 T cells (>15%) while simultaneously lacking infiltration of B cells and CD4 T cells, or T cell co-expression of TCF-1 and PD-1 (Fig. 6 F). While this observation is based on a low sample size, it suggests that the presence of CD8 T cells without CD4 T cells, B cells, and the expression of key T cell phenotypic markers, is not sufficient to achieve an anti-tumor response.

### Increased on-treatment TCR clonality in the tumor of responders

**Figure 7.**
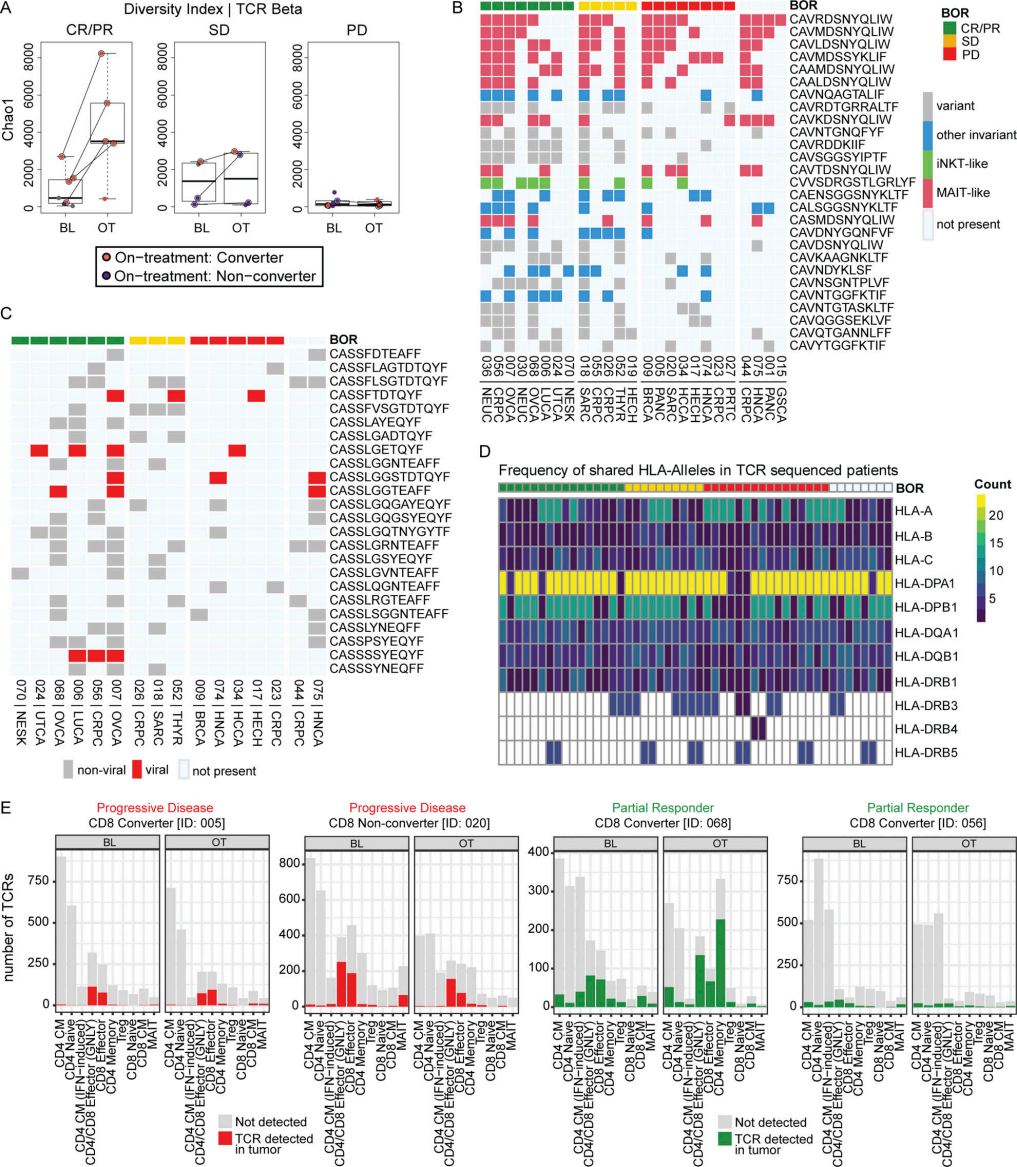
**Increased on-treatment TCR clonality in the tumor of responders. (A)** Box plots of TCR repertoire diversity index (Chao1) from baseline/pretreatment (BL) and on-treatment (OT) tumor biopsies by best overall response (BOR). **(B and C)** (B) TCA and (C) TCB clones shared across patients. Only patients with shared clones are displayed in the plots. **(D)** Overview of shared HLA alleles among patients with available TCR sequencing data. Each column corresponds to a patient and each row to a specific HLA allele. The numerical value in each cell represents the count of patients sharing a particular allele. **(E)** The total number of distinct TCRs found in the periphery sorted by T cell subsets identified by CITEseq analysis of PBMCs at baseline/pretreatment and C1D8 from four patients: two partial responders and two progressors. Also indicated are the number of TCRs found in both the periphery and tumor tissue for each T cell subset detected in the periphery. CM = central memory; iNKT = invariant NK T cells; MAIT = mucosal-associated invariant T cells.

Ipilimumab has been reported to enhance the priming of new T cell clones that could subsequently infiltrate into the tumor tissue (Kvistborg et al., 2014). Analysis of TCR sequences pre- and on-treatment showed that responders’ tumors, unlike progressors’ tumors, had a decrease in clonal diversity coupled with clonal expansion, as depicted by an increase in the Chao1 index (Fig. 7 A). We investigated whether specific T cell clones were shared among patients by comparing their TCR α (TRA) and TCR β (TRB) sequences. Many of the frequently shared TRA sequences were identical to sequences from TRAs of mucosal-associated invariant T cells and other innate-like cells (Kitaura et al., 2016). Additionally, TRA sequences that were shared among patients and derived from variant TCRs were primarily observed in responders regardless of tumor type. Despite fewer public TRB sequences and a reduced frequency of shared TRB sequences, the overall TRB findings paralleled those of the TRA analysis. This may indicate the presence of potentially beneficial public clones in various tumor types (Fig. 7, B and C). However, whether these TCR clones recognize shared cancer-associated antigens remains to be elucidated. We characterized the HLAs of these patients and did not identify any clear associations between HLA haplotypes and response or selection of a specific public TCR (Fig. 7 D). Additionally, for a subset of patients (*n* = 4) who also had single-cell TCR sequencing from PBMCs, significant overlaps were noted between the TCR repertoires of the T cells in the peripheral blood and the tumor. This observation was independent of the response to nivolumab and ipilimumab across different tumor types (Fig. 7 E).

### Decreased ctDNA levels after nivolumab and ipilimumab in responders

In addition to changes in the TME on-treatment, we sought to evaluate biomarker changes in peripheral blood. Baseline ctDNA levels were not significantly associated with response (P = 0.30) or CD8 conversion (P = 0.39). At Cycle 2 Day 1 (C2D1), ctDNA levels decreased ≥50% from pretreatment levels in six of seven responders, while only 3 of 13 patients with stable or progressive disease showed decreases ≥50%. This observation held across tumor types and is consistent with prior studies (Bratman et al., 2020).

### Increased proinflammatory cytokines and circulating activated T cells in responders

**Figure 8.**
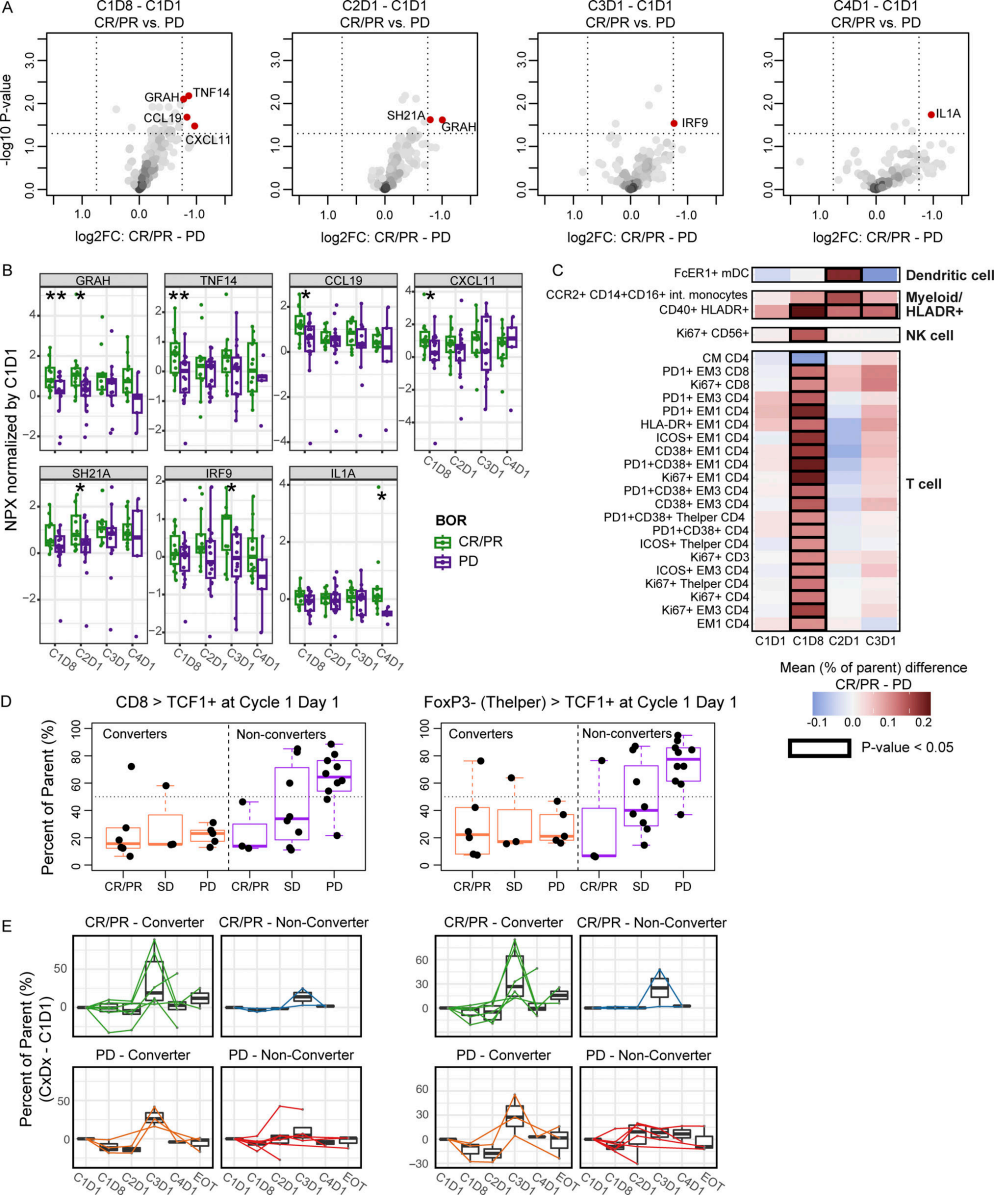
**On-treatment increase of proinflammatory cytokines and expansion of activated and stem cell progenitor-like T cells in the periphery of responders to nivolumab and ipilimumab. (A)** Volcano plots of differentially expressed cytokines (after normalization to pretreatment levels) between responders (CR/PR, *n* = 14) and progressors (PR, *n* = 20) by timepoint. **(B)** Box plots of significantly different cytokines that are differentially expressed between responders (CR/PR) and progressors (PD) by student’s *T* test (*P < 0.05; **P < 0.01). **(C)** Heatmap of gated immune cell populations from PBMCs analyzed by CyTOF showing mean differences (% of parent) between the responders (CR/PR, *n* = 11) and progressors (PD, *n* = 24) at pre- and on-treatment timepoints (C1D8, C2D1, and C3D1), significantly different populations (P < 0.05) by student’s *T* test are denoted by a black outline box. **(D)** Box plots of the C1D1 (pretreatment) circulating TCF1^+^ CD8 cells (left) and TCF1^+^ CD4 (FOXP3−/T helper) cells (right) as percent of parent grouped by BOR and CD8 conversion status. **(E)** Box and line plots of the circulating TCF1^+^ CD8 T cells (left) and TCF1^+^ CD4 (FOXP3−/T helper) cells (right) at each on-treatment timepoint (C1D8, C2D1, C3D1, C4D1, EOT) normalized to pretreatment levels grouped by BOR and CD8 conversion. Box plots show median and quartiles and whiskers represent 1.5 times the IQR. BOR = best overall response; CM = central memory; EM = effector memory; EOT = end of treatment; FC = fold change.

Soluble PD-1, IFNg, CXCL9, and CXCL10 were increased in all patients at C1D8, which aligns with the expected pharmacodynamic effect of nivolumab (Wang et al., 2023). Further analysis of serum proteins by response (CR/PR; *n* = 14 versus PD; *n* = 20) revealed a higher abundance of CCL19, CXCL11, TNF14, and Granzyme H in the responders at C1D8 (Fig. 8, A and B). As expected, responders had a higher systemic inflammatory response after nivolumab and ipilimumab treatment, reflected by increased inflammatory cytokines and higher frequencies of activated T cells and other immune cell populations such as natural killer (NK) cells and dendritic cells. Except for Granzyme H, these proteins were no longer differentially expressed at C2D1.

Broad immune profiling using CyTOF on PBMCs revealed responders had a higher magnitude of circulating T cell proliferation (Ki67^+^) and activation (CD38^+^, HLADR^+^, ICOS^+^) at C1D8. This was accompanied by higher frequencies of proliferating NK cells (Ki67^+^ CD56^+^) and myeloid cell populations, such as CD40^+^ HLADR^+^ cells and FcER1^+^ myeloid dendritic cells (mDCs) at Cycles 2 and 3 in responders (Fig. 8 C).

### Expansion of peripheral blood stem-like T cells in responders

To better understand the stem cell progenitor-like (TCF1^+^) T cell populations that differentiated the responders and progressors in the pretreatment PBMC samples, the on-treatment frequencies of TCF1^+^ CD4 and TCF1^+^ CD8 T cells were assessed. Progressors whose tumors did not convert to CD8-high maintained high TCF1^+^ CD4 and CD8 T cell frequencies that were found pretreatment (Fig. 8 D). However, an increase in the frequency of these populations was uniquely observed in the responders at C3D1 (Fig. 8 E). This finding suggests that while a high frequency of circulating stem cell progenitor-like (TCF1^+^) T cells was associated with poor response to nivolumab and ipilimumab prior to treatment, an expansion on-treatment after two nivolumab and ipilimumab doses could be a mechanistic feature of response.

### Single-cell trajectory analysis revealed differences in transcriptional programs

**Figure 9.**
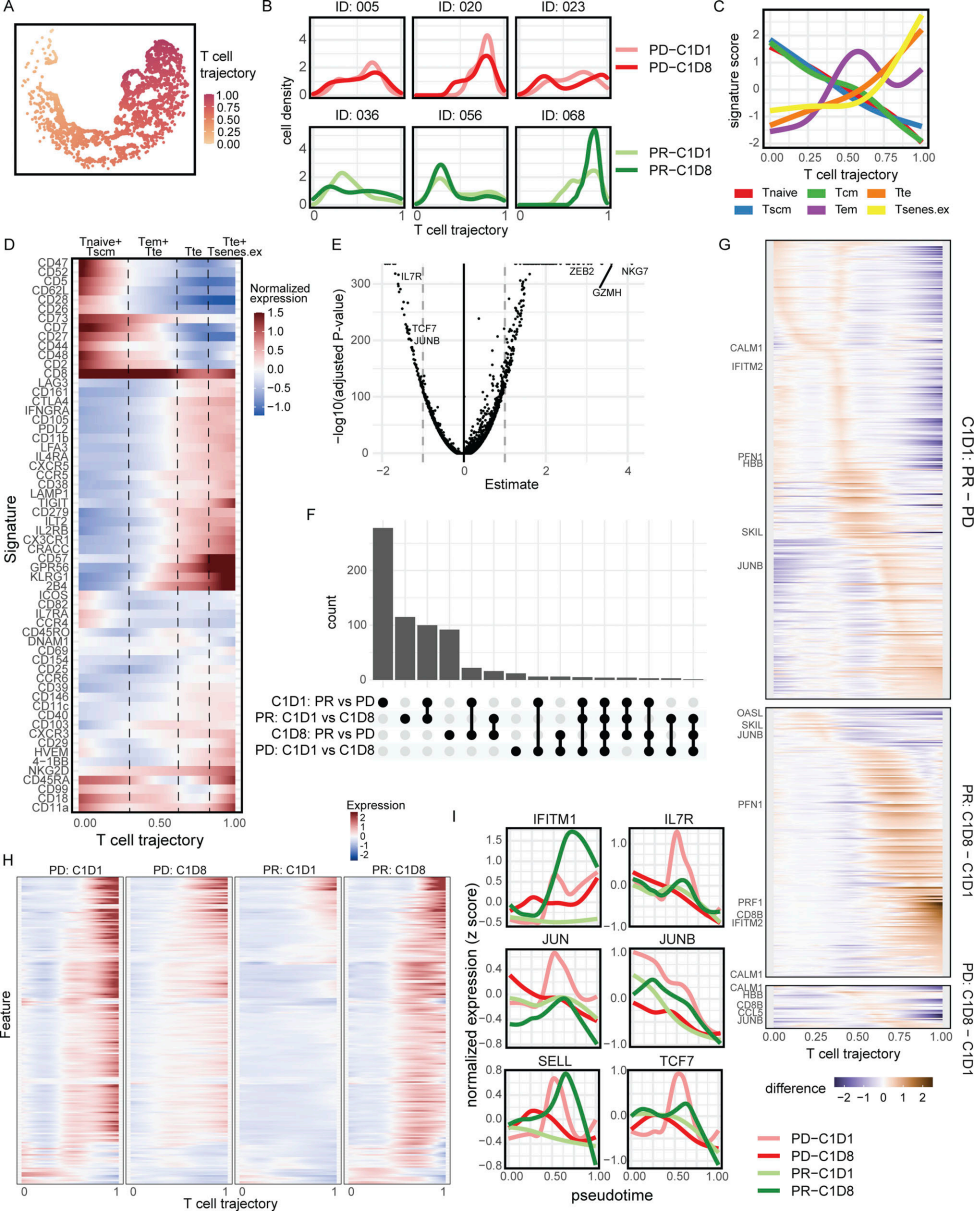
**Single-cell trajectory analysis for circulating CD8 T cells pre- and on-treatment in the CD8-low group.** CITEseq was performed on PBMCs pretreatment (C1D1) and on-treatment (C1D8) from six patients (three partial responders [PR], three progressors [PD], all CD8 converters). **(A)** Pseudotime trajectory clustering of CD8 T cells constructed from ADT components of T cell protein targets. **(B)** CD8 T cell density trends are depicted for progressors (PD, top row) and responders (PR, bottom row) pretreatment (C1D1, lighter lines) and on-treatment (C1D8, darker lines). **(C)** Normalized signatures of Tnaive, Tscm, Tcm, Tem, Tte, and T senescent/exhausted cells (Tsenes.ex) along the T cell transcriptome trajectory. **(D)** Heatmap of normalized expression for all ADT features used for trajectory inference. **(E)** Volcano plot showing results of differential gene expression analysis comparing genes significantly associated with the T cell trajectory. Consistent with the ADT data, naive and stem-like genes including *IL7R* and *TCF7 *significantly enriched earlier in the trajectory, and activated or effector genes like *ZEB2*, Granzymes, and *NKG7 *significantly enriched later in the trajectory. **(F)** UpSet plot summarizing the number of genes significantly associated with pretreatment (C1D1) and on-treatment (C1D8) phenotypes, and responder (PR) and non-responder (PD) pharmacodynamics along the T cell trajectory. **(G)** Heatmap showing DEGs (normalized expression) between comparison groups versus the T cell trajectory pseudotime. Larger differences indicate where along this T cell state trajectory that the gene expression differences are the largest. **(H)** Heatmap of normalized expression for genes significantly changing with nivolumab and ipilimumab treatment among responders for all four clinical groups: C1D1 responders, C1D8 responders, C1D1 non-responders, and C1D8 non-responders. **(I)** Normalized transcript expression of genes associated with the T cell trajectory in a response- or timepoint-dependent manner. JUNB expression was particularly high among non-responder Tn and Tcm/Tscm states at C1D1 and decreased on-treatment but remained stable among responders. Non-responders had high JUNB and TCF7 expression along the Tem and early Tte states. Responders had increased SELL and IFITM1 expression among the effector states compared to non-responder.

Baseline and on-treatment (C1D8) peripheral blood samples from six patients underwent CITEseq analysis to delineate the differences in the epitomic and transcriptional state of T cells. Dimensionality reduction of T cell–relevant ADT markers showed T cells organized along a continuum of cellular states following a continuous model of human CD8 T cell activation and differentiation (Fig. 9, A and B). Transcriptome analysis showed early pseudotime values were enriched for signatures of naive (Tnaive), stem cell memory (Tscm), and central memory (Tcm) states. These early signatures decreased along the continuum and coincided with increased effector memory (Tem) and terminally differentiated effector cells (Tte) states, followed by a peak of effector signatures that coincided with exhaustion signatures at the trajectory end (Fig. 9 C). Transcript expression validated this trajectory model of T cell activation and differentiation (Fig. 9, D and E). The pretreatment transcriptional state of T cells had the most significantly different genes between responders and progressors (Fig. 9, F and G). The number of different genes among CD8 T cells after nivolumab and ipilimumab treatment was also higher in responders. Interestingly, CD8 T cell transcriptional reprogramming on-treatment had the greatest effect on CD8 T cells with Tem and Tte states among responders. Although many of these genes were highly expressed among progressors prior to treatment, these genes, in contrast, decreased on-treatment (Fig. 9, H and I).

## Discussion

The pan-tumor AMADEUS trial aimed to prospectively evaluate the efficacy of nivolumab monotherapy in CD8-high tumors and the impact of nivolumab and ipilimumab combination treatment on tumoral CD8 T cell infiltration and response rates in CD8-low tumors. Ipilimumab was not provided to the CD8-high group because nivolumab and ipilimumab combination is accompanied by increased toxicity (Subudhi et al., 2016; Wolchok et al., 2010) and may be unnecessarily burdensome for patients who are likely to respond to anti-PD-1 monotherapy.

This study primarily enrolled patients with “cold” tumors, with only seven patients meeting the CD8-high threshold (≥15%). The 14% DCR observed in the CD8-high group is numerically lower than historical response rates in approved tumor types (Sharma et al., 2021). However, the limited number of patients in the CD8-high group hinders meaningful analysis and definitive conclusions. Conversely, for patients with CD8-low tumors, the study demonstrated that nivolumab and ipilimumab effectively increased tumoral CD8 levels and elicited antitumor responses in some patients.

Tumor biopsy analysis by transcriptomics and imaging revealed that, despite having low CD8 T cell infiltration, responders’ tumors are more likely to have an existing inflammatory gene signature prior to treatment. This signature is augmented on-treatment to include several immune cell types that orchestrate the immune-mediated antitumor response within a favorable TME. Additionally, tumor TCR analysis provided evidence of new T cell clones expanding and infiltrating into the tumor tissue on-treatment in responders. In contrast, progressors’ tumors start with a metabolic and cell cycle–dominated gene signature pretreatment that remains on-treatment likely due to lack of immune infiltrate. These biomarkers have emerged from analyses across a variety of biopsy sites and tumor types, encompassing both primary tumors and metastases.

We observed that CD8 conversion can occur in various tumor types. This conversion was associated with response, although some patients did not exhibit tumor shrinkage despite converting from CD8-low to CD8-high. This suggests that CD8 infiltration alone is not sufficient to confer an antitumor response. Patients who experienced CD8 conversion without response had a unique gene expression signature expressed in their tumors both pre- and on-treatment that had elements of inflammatory pathways, likely related to the CD8 T cell infiltration. However, their tumors also expressed gene signature pathways typically associated with non-response to ICI and a tumor microenvironment composition that was potentially immunosuppressive (Chen et al., 2016; Lei et al., 2020). Additionally, tumor imaging revealed that these patients may also lack the presence of other key immune cell types such as CD4 T cells and B cells to help confer an anti-tumor response. These findings suggest that while these tumors are CD8 T cell permissive, they maintain mechanisms of immune suppression that prevent a productive antitumor response to nivolumab and ipilimumab.

Broad immune profiling of on-treatment PBMCs revealed an enhanced peripheral inflammatory response in the responders, represented by higher frequencies of activated and proliferating T cells, NK cells, and dendritic cells as well as a higher abundance of pro-inflammatory cytokines in the serum. CITEseq-based trajectory analysis of the circulating T cells also suggests that CD8 T cells in responders have a different transcriptional state compared to progressors prior to treatment, which is reprogrammed after ICI treatment and was more evident in responders. Furthermore, a decrease in ctDNA was observed in responders 3 wk after a single dose of nivolumab and ipilimumab, suggesting the potential of this assay to identify tumor-agnostic antitumor responses early on-treatment.

In peripheral blood analyses, several pretreatment biomarkers, including high levels of serum IL-6 and IL-8, as well as a high frequency of circulating stem cell progenitor-like (TCF1^+^) T cells, correlated with poor clinical outcomes. Interestingly, while tumor tissue TCF1^+^ CD4 T cells were associated with a favorable response, as previously reported (Peng et al., 2021; Rong et al., 2022; Sade-Feldman et al., 2018), a high abundance of these cells (and TCF1^+^ CD8^+^ T cells) circulating in the bloodstream prior to treatment was unfavorable. However, after two doses of nivolumab and ipilimumab, we observed an expansion in these circulating TCF1^+^ T cell populations in responders but not progressors. This suggests the peripheral expansion of T cells with a stem cell progenitor phenotype might indicate a T cell response linked to positive clinical outcomes. Further studies are needed to understand the predictive role of TCF1^+^ T cells both pre- and post-ICI treatment in the tumor and periphery.

Our results suggest that biomarkers including IL-6, IL-8, MSI, TMB, and mRNA tumor gene signatures have predictive relevance across tumor types, particularly for cold tumors. Although some biomarkers broadly categorize tumors into cold and hot phenotypes, a multifactorial approach may refine these categories, better predicting response to combination ICI treatment. Ongoing research aims to incorporate additional pan-tumor datasets for the development and validation of a robust biomarker signature for predicting ICI treatment benefit.

Although the AMADEUS trial yielded valuable insights, several limitations merit attention. First, the study was halted prematurely after enrolling 79 of a planned 200 patients due to strategic considerations by the sponsor. This early cessation, coupled with a lower-than-expected enrollment of CD8-high patients, somewhat constrained our interpretative capacity of the clinical and translational data. Second, the trial design, where CD8-high and CD8-low groups received distinct treatments, did not allow us to definitively ascertain whether baseline tumoral CD8 levels can predict ICI response effectively. Lastly, the chosen 15% CD8 threshold was an educated estimate derived from data available at the time of study design to differentiate between hot and cold tumors and to define CD8 conversion. This selected threshold may not be optimal. Further research should aim to calibrate this cutoff more precisely with the currently available data, including results from this study.

Our findings establish a robust groundwork for future biomarker-driven clinical trials and in-depth mechanistic studies. Further understanding of solid tumor T cell permissiveness will play a pivotal role in shaping the rational design of ICI combinations by employing a “precision immunology” approach. This approach holds immense potential to enhance clinical outcomes, particularly for patients with advanced, heavily pretreated, and immunologically cold tumors.

## Materials and methods

### Study design

In this exploratory study, patients ≥18 years of age with histologically or cytologically confirmed cancer that was metastatic, unresectable, or recurrent were enrolled from six academic hospitals in the U.S. In June 2019, the protocol was amended to limit enrollment to tumor types known to be responsive to immunotherapy, have high prevalence of CD8 ≥15% tumors, and/or were observed in the study to have tumors convert from CD8-low (<15%) to CD8-high (≥15%) following initiation of nivolumab and ipilimumab treatment. Patients with these tumor types were eligible to participate regardless of baseline CD8 results (either <15% or ≥15%). Prior immunotherapy, including anti-PD-1 or anti-PD-L1, was allowed if it was not the patient’s most recent line of therapy. Additional key eligibility criteria included a newly obtained core needle or incisional biopsy of a non-bone tumor lesion not previously irradiated, Eastern Cooperative Oncology Group (ECOG) performance status of 0–1, and measurable disease as defined by Response Evaluation Criteria in Solid Tumors version 1.1 (RECIST version 1.1). Key exclusion criteria included having experienced any grade 3 or higher symptomatic IRAE on prior immunotherapy; any known, untreated brain metastases; active, known, or suspected autoimmune disease; any condition requiring systemic treatment with either corticosteroids (>10 mg daily prednisone equivalent) or other immunosuppressive medications within 14 days of the start of study intervention; anticancer chemotherapy, radiotherapy, immunotherapy, or investigational agents within 14 days of the start of study intervention.

This was a non-randomized, open-label, multicenter study to evaluate nivolumab with or without ipilimumab, with treatment assignment based on the percentage of tumoral CD8 cells at the time of treatment. This study planned to enroll up to 200 total patients, which would have allowed for sufficient sample sizes in the CD8-high and CD8-low groups to report clinical response and CD8 conversion rates within multiple tumor subgroups (full details available in Protocol). There are two distinct cohorts enrolled in this study. The advanced metastatic cancer cohort enrolled patients with varying advanced solid tumors and is reported in this manuscript. An additional cohort of advanced prostate cancer patients is currently ongoing, and results for this prostate cohort will be reported separately.

After consenting to participate in this study, patients underwent a core needle or incisional biopsy; fine needle aspiration was not acceptable. The tumor tissue was sent to the University of Texas MD Anderson Cancer Center Immunohistochemistry and Image Analysis Laboratory (Clinical Laboratory Improvement Amendment [CLIA] and College of American Pathologists certified) to determine the percentage of tumoral CD8 cells. The test for CD8 expression is a laboratory-developed immunohistochemistry (IHC) test used to determine patient eligibility. This test is not a Food and Drug Administration–approved device, and its use is investigational.

Treatment was assigned to patients according to the percentage of CD8 cells in their baseline tumor biopsy. Patients with CD8 ≥15% were assigned to the CD8-high group and were treated with nivolumab monotherapy. Patients with CD8 <15% were assigned to the CD8-low group and were treated with nivolumab in combination with ipilimumab. At the time of disease progression, patients assigned to the CD8-high group had the option to subsequently crossover and receive combination nivolumab and ipilimumab. After receiving four cycles in the CD8-high group or six cycles in the CD8-low group, patients continued to receive maintenance nivolumab.

The protocol and all amendments were reviewed and approved by the University of Texas MD Anderson Cancer Center Institutional Review Board before the study was initiated and/or implementation of any changes made to the study design, except for changes necessary to eliminate an immediate hazard to study patients. The study was conducted in accordance with the principles of the Declaration of Helsinki and the International Conference on Harmonization Good Clinical Practice guidelines. All patients provided written informed consent before any study procedures were performed. The trial protocol and statistical analysis plan are available as supplementary material.

### Procedures

Within the CD8-high group, nivolumab was administered at 360 mg intravenously (iv) every 3 wk (Q3W). Starting at Cycle 5, CD8-high patients who continued to show clinical benefit received maintenance nivolumab at 480 mg iv every 4 wk (Q4W) until disease progression or intolerable toxicity. Within the CD8-low group, nivolumab was administered at 360 mg iv Q3W and ipilimumab was administered ∼30 min later at 1 mg/kg iv Q3W for the first two doses and then every 6 wk for the third and fourth doses. Starting at Cycle 7, CD8-low patients who continued to show clinical benefit received maintenance nivolumab at 480 mg iv Q4W until disease progression or intolerable toxicity.

Dose reductions of nivolumab and ipilimumab were not permitted but doses could be held for toxicity management. If ipilimumab was discontinued and/or the patient had received four doses of ipilimumab, a repeat dose of ipilimumab at a later date was not permitted. Patients who discontinued ipilimumab dosing early due to toxicities were allowed to continue to receive nivolumab, including maintenance nivolumab.

Patients were assessed radiographically within 7 days prior to Day 1 of Cycle 4, Cycle 6, and every even cycle thereafter until death, radiographic disease progression, or initiation of subsequent therapy, whichever occurred first. Patients were subsequently followed for survival. Safety assessments included vital signs, physical examinations, and laboratory tests. AEs were graded according to the National Cancer Institute Common Terminology Criteria for AEs, version 5.0. AE terms were coded using the Medical Dictionary for Regulatory Activities version 25.0.

Blood samples for isolation of PBMCs were collected longitudinally at participating clinical sites, shipped overnight, and processed at a central location (Infinity Biologix) over a Ficoll gradient and cryopreserved. Serum was processed within 2 h of collection at each site and frozen immediately at −80°C, then batch shipped to a central biorepository. Blood sampling for immune biomarkers occurred during screening, at C1D1 and C1D8, and Day 1 of each subsequent treatment cycle through Cycle 4, and again at the end of treatment visit. Core needle tumor biopsies were collected during the screening period for mandatory CD8 IHC assessment (baseline). In the CD8-low group, on-treatment biopsies were collected if medically feasible during Cycle 2 and Cycle 6 (after the second and fourth doses of ipilimumab, respectively). On-treatment tumor biopsies in the CD8-high group were collected during Cycle 2 (after the second dose of nivolumab) and optionally at disease progression (PD). The first two core biopsies were formalin fixed and paraffin embedded for CD8 assessment. Any remaining cores were immediately snap-frozen and shipped to a central biorepository (Brooks Life Sciences) for tumor and immune biomarker analysis. After CD8 IHC assessment, fixed and paraffin-embedded tissue was shipped to the central biorepository for storage and further analysis.

### Outcomes

The coprimary endpoints were DCR and the proportion of patients in the CD8-low group whose tumors converted from CD8-low (<15%) to CD8-high (≥15%) at any on-treatment biopsy. Secondary endpoints were ORR, PFS, OS, the association of CD8 percentage with clinical outcomes, and the incidence of AEs. Key exploratory endpoints included the evaluation of tumor and immune biomarkers pre- and on-treatment.

### CD8 IHC

IHC studies were performed in a CLIA-certified laboratory using an automated slide stainer (Leica Bond Max; Leica Biosystems) and an anti-human CD8 primary antibody (Lab Vision, clone: C8/144B; Dilution 1:20; Thermo Fisher Scientific) with 3,3′-diaminobenzidine chromogen, and counterstained with hematoxylin. All slides were stained using previously optimized conditions with positive and negative control tissue placed on the same slide adjacent to the test tissue. IHC and hematoxylin & eosin–stained slides were converted into high-resolution digital images at 20× magnification using an Aperio slide scanner. Pathologists identified and marked areas for analysis, focusing on regions of tissue with neoplastic cells for pretreatment biopsies and including treated tumor beds for posttreatment biopsies. Specifically, in pretreatment biopsies, areas of fibroadipose, fibrous, or lymphoid tissues containing neoplastic cells were delineated, while regions where neoplastic cells were unattached and intermingled with blood or lymphocytes were not included. In posttreatment biopsies, this process was replicated, with the addition of assessing treated tumor beds regardless of the presence of residual neoplastic cells. Image analysis software (Aperio ImageScope) was then applied to quantify the number and percentage of IHC-positive lymphocytes within the designated areas marked by the pathologist. The Aperio image analysis software quantified the percentage of CD8-positive lymphocytes and the number of CD8-positive lymphocytes in a given area (the latter reported as CD8^+^ cells/mm^2^).

### Statistical analysis

Efficacy and safety analyses were conducted on the modified intent-to-treat population, defined as all patients who received at least one dose of study intervention. Analyses of changes in CD8 percentage were conducted on the on-treatment biopsy population, defined as all patients with at least one on-treatment biopsy with sufficient CD8 results. For all analyses, patients were grouped according to the treatment group assigned at enrollment. This study was not intended or powered for statistical comparison between groups and no adjustment for multiple comparisons was performed for the clinical endpoints.

DCR (referred to as clinical benefit rate in the protocol and statistical analysis plan), a co-primary endpoint, was defined as the proportion of patients with best overall response of CR or PR, or SD lasting at least 6 mo per investigator-assessed RECIST version 1.1. Confirmation of response by a repeat tumor assessment was required for a best overall response of CR or PR. CIs for DCR were calculated using a beta (0.4, 1.6) prior. CIs for the proportion of patients whose tumors converted from CD8-low to CD8-high, the second co-primary endpoint, were calculated assuming no prior.

For secondary endpoints, ORR was defined as the proportion of patients with a best response of CR or PR; PFS as the time from treatment initiation until radiographic disease progression or death (whichever occurred first); and OS as the time from treatment initiation until death due to any cause. CIs for ORR were calculated using a beta (0.4, 1.6) prior. The Kaplan–Meier method was used to estimate PFS, OS, and the corresponding confidence intervals. Logistic regression models were fit to assess the relationship between CD8 percentage (at baseline, on-treatment, change from baseline to on-treatment, and conversion from CD8-low to CD8-high as a binary variable) and clinical response (DCR and ORR). Each regression model included an intercept term. P values were calculated using a Wald test to test whether the coefficient for the CD8 variable was significantly different from zero. Statistical analyses of clinical data were performed using SAS version 9.4 and R version 4.1.1.

All statistical analyses of biomarker data were conducted using R version 4.1.2.

### Translational analysis

Tumor tissue analysis included bulk RNAseq, WES, and mIF imaging. PBMC analysis included high dimensional flow cytometry analysis (X50), broad immune profiling using CyTOF, CITEseq, and single-cell TCR sequencing. Serum and plasma analysis included proteomics and ctDNA quantification, respectively. Additional information on assay methods is detailed in the supplementary materials. Sample sizes for the various assays are provided in Table S11.

### Immunophenotyping by mass CyTOF

A broad immunophenotyping panel was used on cryopreserved PBMC by CyTOF analysis run under uniform protocols (Hartmann et al., 2019) at Primity Bio in a blinded fashion. Cryopreserved PBMCs were thawed in 37°C prewarmed RPMI-1640 containing 10% FBS and 25 U/ml of benzonase. Samples were washed once more in RPMI-1640 containing 10% FBS and 25 U/ml of benzonase and a third time in 37°C prewarmed RPMI-1640 containing 10% FBS. Samples were resuspended in 1,000 nM of cisplatin for viability discrimination, prepared in PBS containing 0.1% BSA, for 5 min at room temperature, and then washed with staining buffer. Human BD Fc block (BD Biosciences) was added to the cells for 10 min at 4°C followed by the surface antibody cocktail. The surface staining cocktail was incubated for 30 min at 4°C. Samples were washed out of the stain twice with staining buffer. The cells were then resuspended in FOXP3 Transcription Factor 1× Fix/Perm buffer (eBioscience) for 1 h at room temperature to prepare the cells for intracellular staining. The fixation was then followed by a wash in 1× permeabilization buffer. The intracellular staining cocktail was prepared in the permeabilization buffer and added to the samples and incubated at room temperature for 1 h. Following the intracellular stain, the samples were washed twice with the permeabilization buffer and once with staining buffer. Prior to acquisition on the CyTOF, samples were resuspended in an iridium-intercalating solution for at least 24 h and stored at 4°C. On the day of acquisition, the samples were washed five times in cell culture grade water (HyClone) and run on the CyTOF Helios instrument (Fluidigm). Details on the CyTOF panel are displayed in Table S12. Data were analyzed using CellEngine version 1 cloud-based flow cytometry analysis software (CellCarta).

**Figure S4.**
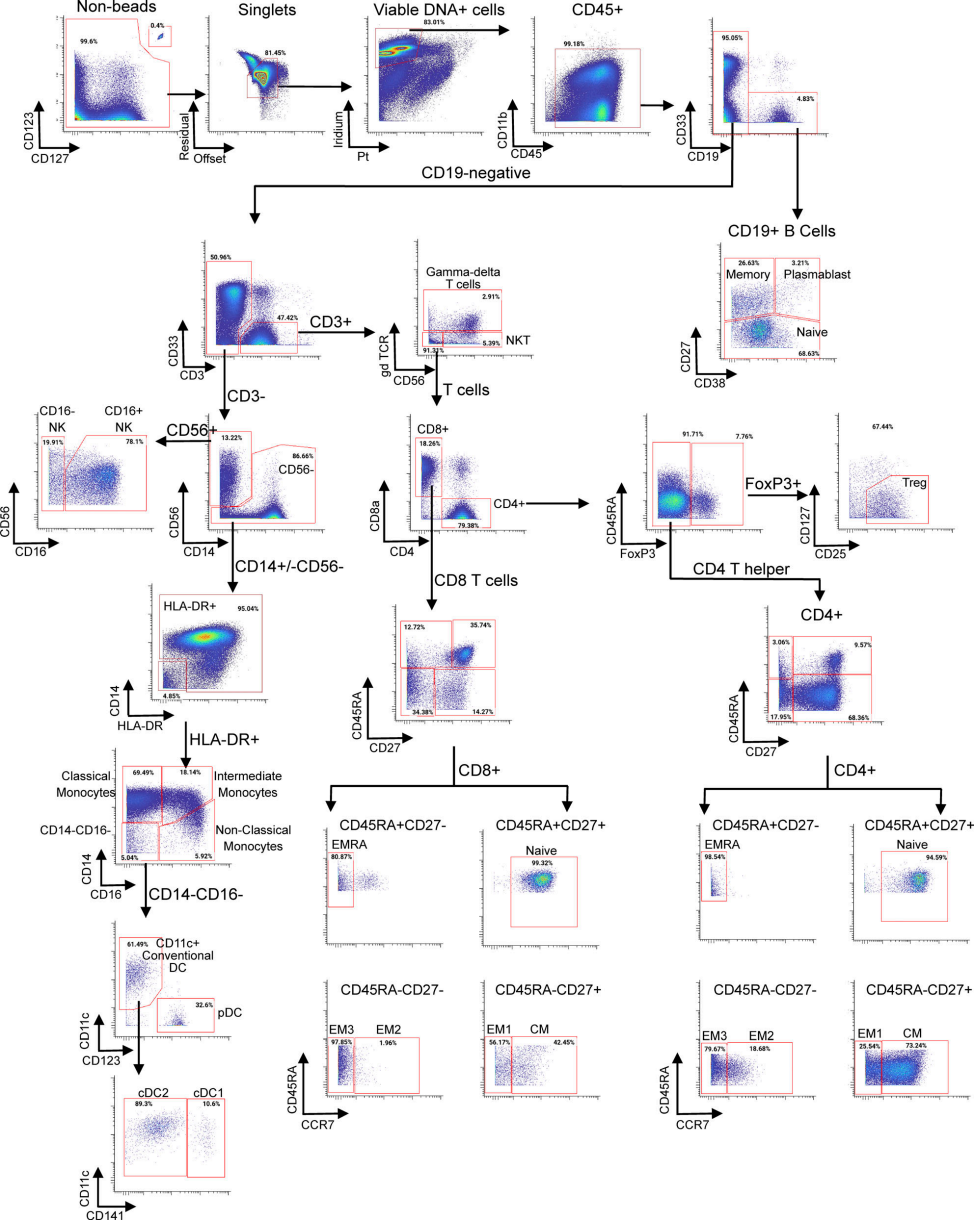
**CyTOF broad immune profile panel gating strategy.** CM = central memory; EM = effector memory; EMRA = terminally differentiated effector memory.

Supervised gating was performed manually by a scientist without reference to clinical outcome with a secondary review completed by a different scientist. High-level gates were tailored per sample. Single marker gates were drawn uniformly for analysis across patients and timepoints. After gating for live singlets, immune populations were defined as following, as shown in Fig. S4. B cells were identified based on CD19 expression and further distinguished into memory versus naive versus plasmablast based on expression of CD38 versus CD27. NK cells were identified based on CD56 expression and further subdivided based on CD56 versus CD16 expression. Monocytes were identified based on expression of CD14 and HLA-DR and further subdivided into classical, non-classical, and intermediate based on the expression of CD14 versus CD16. Dendritic cells were defined as HLA-DR^+^CD14^−^CD16^−^ non-lymphocytes and further distinguished between mDC and plasmacytoid (pDC) based on expression of CD11c versus CD123, respectively. mDCs were further subdivided based on CD141 expression into conventional dendritic cell (cDC) type 1 (CD141^+^) and cDC type 2 (CD141^−^). Conventional T cells were identified based on CD3 expression and the absence of γδ TCR or CD56. T cells were further subdivided into CD4 and CD8 subsets; CD8 and CD4 T helper naive, effector, and memory populations were identified based on CD45RA, CD27, and CCR7 expression. Regulatory T cells were identified based on FOXP3, CD25, and CD127 expression.

Optimized concentrations/dilutions for antibodies used in CyTOF experiments were: CD45, CD3, CD19, CD117, CD11b, CD4, CD8a, CD11c, CD14, FcER1, CD123, gdTCR, CD45RA, CD366, CD274, CD27, Tbet, CD152, CD278, FOXP3, CD33, CD45RO, CD127, CD197, Ki67, CD25, TCRVa24-Ja18, CD38, HLA-DR, CD56, CD16 (all used at 1:100 per manufacturer’s recommendation); CD39, 3 µg/ml; CD40, 1.5 µg/ml; CD69, 6 µg/ml; CD86, 6 µg/ml; CD1c, 3 µg/ml; CD64, 6 µg/ml; CD141, 3 µg/ml; CD154, 3 µg/ml; CD40, 1.5 µg/ml; CD192, 6 µg/ml; nivolumab, 1 µg/ml; anti-human IgG4, 1 µg/ml.

Populations were manually gated in CellEngine, and the percent of parents of each population for each sample was exported for downstream analysis in R. The percent of the parent is calculated from the immediately preceding population in the gating hierarchy. In the CD8-low group, the percent of parent of each cell type at pretreatment was compared between responders (CR/PR) and PD and between CD8 converters and CD8 non-converters using the student’s *T* test. After treatment, the percent of parent of each cell type at each timepoint was normalized to baseline by subtracting the pretreatment levels, and a comparison was made between responders and progressive disease and between CD8 converters and CD8 non-converters using the student’s *T* test. A comparison was deemed significant if the P value was <0.01.

### High parameter flow cytometry of T lymphocytes

**Figure S5.**
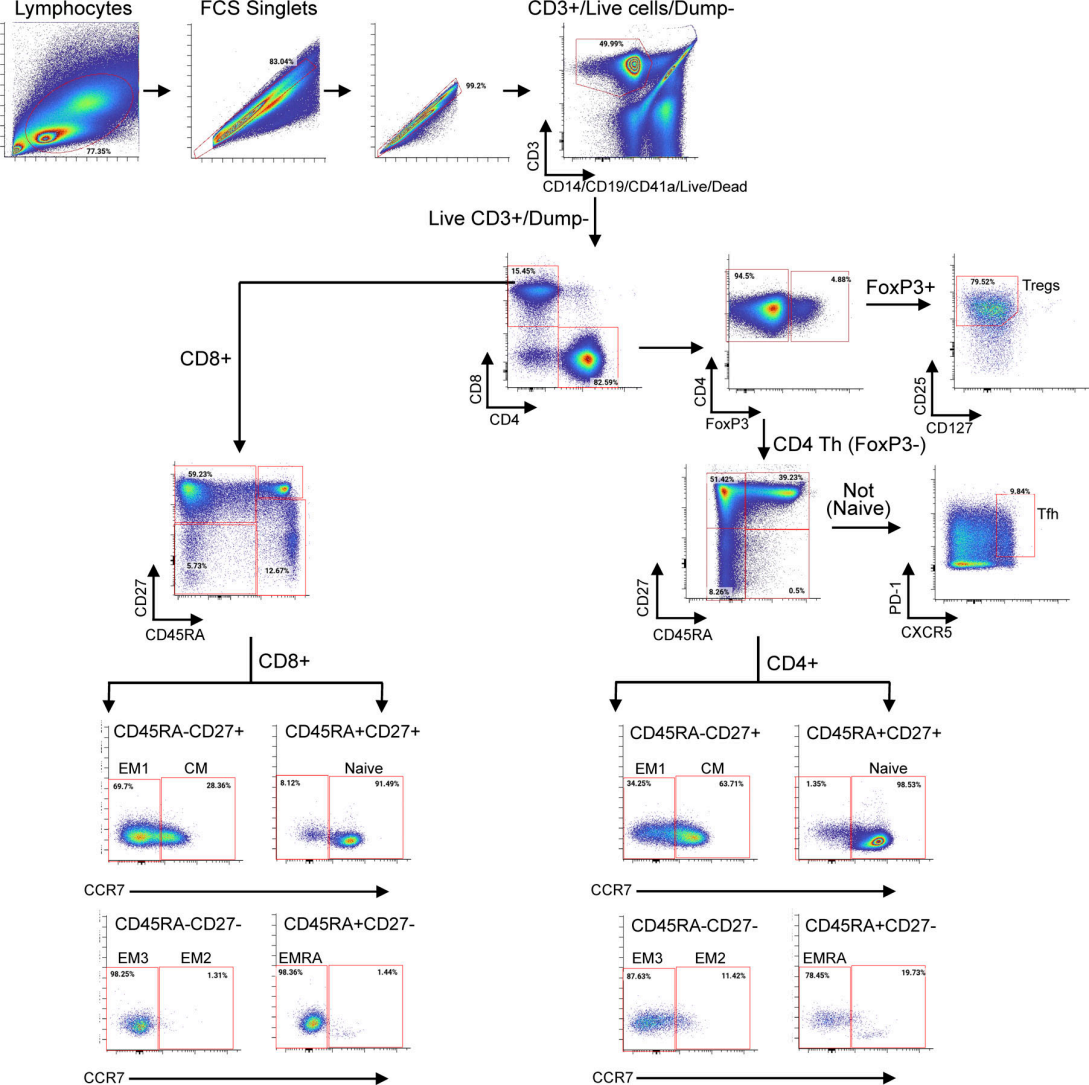
**X50 T cell panel gating strategy.** CM = central memory; EM = effector memory; EMRA = terminally differentiated effector memory.

Cryopreserved PBMC samples for fluorescent flow cytometry were analyzed in the Translational Cytometry Laboratory of the Penn Cytomics and Cell Sorting Shared Resource (University of Pennsylvania, Philadelphia, PA, USA) on an extensively prequalified 28-color BD Symphony A5 cytometer (BD Biosciences). Staff were blinded to treatment group and clinical outcome. At the time of analysis, cryopreserved PBMC samples were thawed in 37°C prewarmed RPMI-1640 medium (Gibco) containing 10% FBS and 100 U/ml of penicillin-streptomycin (Gibco). Samples were washed, counted, and resuspended in medium containing 1 mg/ml DNase I (Roche) and 5 mM magnesium chloride, and incubated at 37°C for 1 h. After resting, cells were washed with PBS without additives (Corning) and transferred to staining tubes. PBMC was incubated with 1 µl (0.2 µg) of 0.2 mg/ml nivolumab antibody (Selleck Chemicals) for 5 min at room temperature, followed by the addition of a Fixable Viability Stain 510 for 10 min at room temperature in the dark. Cells were then washed twice with FACS wash buffer (PBS, 1% BSA, 2 mM EDTA). A surface antibody cocktail (T cell phenotyping antibody panel, Table S13) was prepared daily and used to stain up to 1 × 10^7^ cells per tube. Cells were incubated for 20 min at room temperature followed by washing twice with FACS staining buffer. The cells were resuspended in FOXP3 Transcription Factor Staining Buffer Fix/Perm solution (eBiosciences) and incubated for 1 h at room temperature to prepare the cells for intracellular staining. After fixation, the samples were washed with FOXP3 permeabilization buffer. A freshly prepared cytoplasmic/intracellular staining cocktail master mix was added to the samples and incubated overnight at 4°C. The following day, the samples were washed with permeabilization buffer and resuspended in FACS wash buffer. Cells were stored at 4°C in the dark and acquired within 2 h. Following daily quality control, the instrument was standardized by setting hard-dyed beads (BD Biosciences, Cytometer Setup and Tracking Beads) to predetermined target channels. Compensation controls (Invitrogen UltraComp eBeads or cells for Live/Dead stain) were prepared daily along with a frozen PBMC process control. The compensation matrix was calculated in Diva software (BD Biosciences) and used only for that day’s run. Data were analyzed using CellEngine. High-level gates were tailored per patient across all timepoints by at least two investigators blinded to patient outcome. Single marker gates were drawn uniformly for analysis across patients and timepoints, with a representative gating strategy provided in Fig. S5.

After gating for live cells and the CD3^+^ population, T cell populations were defined as follows: a combination of CD45RA, CD27, and CCR7 expression on CD4^+^ and CD8^+^ T cells was used to define naive (CD45RA^+^CD27^+^CCR7^+^), Tcm (CD45RA^−^CD27^+^CCR7^+^), Tem1 (CD45RA^−^CD27^+^CCR7^−^), Tem2 (CD45RA^−^CD27^−^CCR7^+^), Tem3 (CD45RA^−^CD27^−^CCR7^−^), and terminally differentiated effector memory (CD45RA^+^CD27^−^CCR7^−^) subpopulations. CD4^+^ regulatory T cells were defined as FOXp3^+^CD25^hi^CD127^−^/low. Expression of additional differentiation, activation, and inhibitory markers were evaluated within each of these compartments.

Optimized concentrations/dilutions for antibodies used in the high parameter flow cytometry experiments were CD45RA, 1:200; CD8a, 1:160; CD185, 1:400; CD25, 1:200; CD226, 1:65; CD27, 1:500; CD4, 1:800; CD197, 1:40; CD223, 1:100; CD14, 1:40; CD19, 1:160; CD41a, 1:260; CD3, 1:65; CD137, 1:100; CD244, 1:20; CD366, 1:200; CD39, 1:100; CD28, 1:100; CD278, 1:100; CD127, 1:160; CD38, 1:160; TIGIT, 1:40; Eomes, 1:100; CD152, 1:400; FOXP3, 1:400; T-bet, 1:600; TCF1, 1:125; Ki67, 1:600; KLRG1, 1:100; nivolumab, 1 mg/ml; and anti-human IgG4, 1:200.

Populations were manually gated in CellEngine and the percent of parent of each population for each sample was exported for downstream analysis in R. In the CD8-low group, the percent of parent of each cell type at pretreatment was compared between responders (CR/PR) and PD; and between CD8 converters and CD8 non-converters using the student’s *T* test. After treatment, the percent of parent of each cell type at each timepoint was normalized to baseline by subtracting the pretreatment levels, and a comparison was made between responders and PD and between CD8 converters and CD8 non-converters using the student’s *T* test. A comparison was deemed significant if the P value was <0.05 and the absolute difference between the group means was >20%.

### Serum proteomics profiling

Serum proteins were quantified using Olink multiplex proximity extension assay (PEA) panels (Olink Proteomics) according to the manufacturer’s instructions (Assarsson et al., 2014). The assay was performed at the Olink Analysis Service Center. The basis of PEA is a dual-recognition immunoassay, where two matched antibodies labeled with unique DNA oligonucleotides simultaneously bind to a target protein in solution. This brings the two antibodies into proximity, allowing their DNA oligonucleotides to hybridize, serving as a template for a DNA polymerase-dependent extension step. This creates a double-stranded DNA “barcode,” which is unique for the specific antigen and quantitatively proportional to the initial concentration of the target protein. The hybridization and extension are immediately followed by PCR amplification and the amplicon is then finally quantified by microfluidic quantitative PCR using Fluidigm BioMark HD system (Fluidigm Corporation). Data were normalized using internal controls in every single sample, inter-plate control and negative controls, and correction factor, and expressed as log_2_-scale, which is proportional to the protein concentration. The final assay readout is reported as normalized protein expression (NPX) values, which is an arbitrary unit on a log_2_-scale where a higher value corresponds to a higher protein expression. One NPX difference equals the doubling of the protein concentration. In this study, two Olink panels (Target96 Immuno-Oncology and Target96 Immune Response) were used that consist of 172 unique analytes. Additional details about the analytes, detection range, data normalization, and standardization are available at https://www.olink.com/resources-support/document-download-center/.

For the CD8-low group, pretreatment (baseline) differential expression analysis was performed for each cytokine comparing responders (CR/PR) to progressors (PD) and comparing CD8 converters to non-converters using the student’s *T* test. Differentially expressed cytokines were defined as those with P value <0.05 and a log_2_-fold-change greater ±0.75. After treatment, the NPX value was normalized to pretreatment by subtracting the pretreatment levels, and a comparison was made between responders and PD and between CD8 converters and CD8 non-converters using the student’s *T* test. Differentially expressed cytokines were defined as those with P value <0.05 and a log_2_-fold-change greater ±0.75.

### Whole exome and transcriptome sequencing

Formalin-fixed and paraffin-embedded (FFPE) tumor and normal PBMC samples were profiled using ImmunoID NeXT (Personalis, Inc.)—an augmented exome/transcriptome platform and analysis pipeline, which produces comprehensive tumor mutation information, gene expression quantification, neoantigen characterization, HLA typing, and allele-specific HLA loss of heterozygosity data, TCR repertoire profiling, and TME profiling. Whole exome library preparation and sequencing were performed by Personalis, Inc. as a service using augmented exome sequencing. DNA extracted from tumor and PBMCs was used to generate whole exome capture libraries using the KAPA HyperPrep Kit and Agilent’s SureSelect Target Enrichment Kit, according to manufacturers’ recommendations, with the following amendments: (1) Target probes were used to enhance coverage of biomedically and clinically relevant genes. (2) Protocols were modified to yield an average library insert length of ∼250 bp. (3) KAPA HiFi DNA Polymerase (Kapa Biosystems) was used in place of Herculase II DNA polymerase (Agilent). Paired-end sequencing was performed on NovaSeq instrumentation (Illumina).

Whole transcriptome sequencing results were aligned using STAR, and normalized expression values in transcripts per million (TPM) were calculated using Personalis’ ImmunoID NeXT tool, Expressionist. For RNAseq and alignment quality control, the following metrics were evaluated: average read length, average mapped read pair length, percentage of uniquely mapped reads, number of splice sites, mismatch rate per base, deletion/insertion rate per base, mean deletion/insertion length, and anomalous read pair alignments including interchromosomal and orphaned reads. The ImmunoID NeXT DNA and RNA Analysis Pipeline aligns reads to the hs37d5 reference genome build. The pipeline performs alignment, duplicate removal, and base quality score recalibration using best practices outlined by the Broad Institute (DePristo et al., 2011; McKenna et al., 2010). The pipeline uses Picard to remove duplicates and the Genome Analysis Toolkit to improve sequence alignment and correct base quality scores. Aligned sequence data are returned in compressed binary (BAM) format according to sequence alignment map specification. TPM values were quantile normalized to remove batch effects.

Differential expression analysis was performed at pretreatment and on-treatment, comparing responders (CR/PR) to progressors (PD), and comparing CD8 converters to non-converters using a student’s *T* test. A gene was called differentially expressed if the P value of the comparison was <0.05 and the absolute log_2_-fold-change was >1. To calculate gene expression signatures on a given gene set, z-scores were obtained for each gene and averaged across patients. Fisher’s exact test was used to assess the relationship between TMB and MSI with response.

Clonal diversity of TCR sequences, measured by the Chao1 repertoire diversity index, was compared pre- and on-treatment between responders and non-responders. TRA and TRB sequences were investigated to identify whether specific T cell clones were shared among patients. Public TCR sequences were compared with those in the McPAS_TCR (Tickotsky et al., 2017) and VDJdb (Shugay et al., 2018) databases, focusing on invariant T cells, viral antigens, and other recognized antigens.

### Multiplex tissue staining and imaging

Tissues were fixed in formalin followed by paraffin embedding. All tissue imaging was performed under the guidance of an expert pathologist (T.J. Hollman) in the Advanced Immunomorphology Platform Laboratory at Memorial Sloan Kettering Cancer Center (New York, NY). Primary antibody staining conditions were optimized using standard immunohistochemical staining on the Leica Bond RX automated research stainer with diaminobenzidine detection (DS9800; Leica Bond Polymer Refine Detection). Using 4-µm tissue sections and serial antibody titrations on control tonsil tissue, the optimal antibody concentration was determined followed by transition to a seven-color multiplex assay with equivalency. Multiplex assay antibodies and conditions are described in Table S14.

FFPE tissue sections were baked for 3 h at 62°C in a vertical slide orientation with subsequent deparaffinization performed on the Leica Bond RX followed by 30 min of antigen retrieval with Leica Bond ER2 followed by six sequential cycles of staining with each round including a 30-min combined block and primary antibody incubation (Akoya antibody diluent/block). For Ki-67 and panCK, detection was performed using a secondary horseradish peroxidase (HRP)–conjugated polymer (Akoya Opal polymer HRP Ms + Rb; 10-min incubation). Detection of all other primary antibodies was performed using a goat anti-mouse Poly HRP secondary antibody or goat anti-rabbit Poly HRP secondary antibody (10-min incubation; Invitrogen). The HRP-conjugated secondary antibody polymer was detected using fluorescent tyramide signal amplification using Opal dyes 520, 540, 570, 620, 650, and 690 (Akoya Biosciences). The covalent tyramide reaction was followed by heat-induced stripping of the primary/secondary antibody complex using Akoya AR9 buffer and Leica Bond ER2 (90% AR9 and 10% ER2) at 100°C for 20 min preceding the next cycle. After six sequential rounds of staining, sections were stained with Hoechst 33342 (Invitrogen) to visualize nuclei and mounted with ProLong Gold antifade reagent mounting medium (Invitrogen).

#### Multispectral imaging and spectral unmixing

Seven-color multiplex stained slides were imaged using the Vectra Multispectral Imaging System version 3 (Akoya). Scanning was performed at 20× (200× final magnification). Filter cubes used for multispectral imaging were DAPI, FITC, Cy3, Texas Red, and Cy5. A spectral library containing the emitted spectral peaks of the fluorophores in this study was created using the Vectra image analysis software (Akoya). Using multispectral images from single-stained slides for each marker, the spectral library was used to separate each multispectral cube into individual components (spectral unmixing) allowing for identification of the seven marker channels of interest using Inform 2.4 image analysis software.

#### mIF image analysis

Individual region of interest (ROI) images were exported to TIFF files and run through a machine-learning algorithm to segment individual whole cells along the membrane border using the nuclear as well as multiple membrane markers using the Mask-R-CNN algorithm. Cell phenotyping for each marker was obtained for each image as follows: first, a background threshold was determined, and a cell was deemed positive for the maker if >50% of the pixels in that cell exceeded the threshold. The threshold was obtained via k-means clustering (k = 3 centers) of the pixel intensities of all the cells in the image. The threshold was set to equal the average of the two maximum of the three k-means centers; after cell phenotyping, the percent of parent of each cell type was computed for each ROI and averaged across the ROIs in the image. In the CD8-low group, the percent of parent of each cell type at pre- and post-treatment was compared between responders (CR/PR) and PD, and between CD8 converters and CD8 non-converters using the student’s *T* test. A comparison was deemed significant if the P value was <0.05 and the absolute difference between the group means was >5%.

### ctDNA

ctDNA was detected and quantified using a personalized, tumor-informed, multiplex PCR (mPCR) next-generation sequencing-based assay (Signatera, Natera, Inc.) as previously described (Reinert et al., 2019). Briefly, a set of 16 high-ranked, patient-specific, somatic, single nucleotide variants (SNVs) were selected for mPCR testing by WES performed on FFPE tumor tissue and matched normal blood sample. The mPCR primers targeting the selected personalized SNVs were designed, synthesized, and used to track ctDNA in the patient’s longitudinal plasma samples. Plasma samples with at least 2 out of 16 SNVs detected were considered ctDNA positive. ctDNA concentration was reported as mean tumor molecules (MTM) per ml of plasma.

Associations between baseline ctDNA levels, response, and CD8 conversion were calculated using logistic regression. A student *t* test was used to assess the difference between responders (CR/PR) and progressors (PD) of the baseline-adjusted C2D1 MTM/ml, where all MTM/ml values were transformed to a log_10_ scale and the baseline values subtracted from the C2D1 values.

### CITEseq, single-cell TCR, and B cell receptor (BCR) sequencing

Cryopreserved pre- and on-treatment (C1D1 and C1D8) PBMC samples from six patients in the CD8-low group were selected for analysis using CITEseq and single-cell TCR sequencing. Three vendors (Abiosciences, Q2 Solutions, and MedGenome) were utilized for sample processing using the same uniform processes. For each specimen, PBMCs were thawed in a water bath, resuspended into single-cell suspensions in Cell Staining Buffer (Cat # 420201; BioLegend), and assessed and normalized using the Countess II Automated Cell Counter (Thermo Fisher Scientific). Cells were then blocked with the Human TruStain FcX Fc Receptor Blocking Solution (Cat # 422302; BioLegend), stained with the TotalSeq-C Human Universal Cocktail, V1.0 (Cat # 399905; BioLegend), then washed and filtered to obtain single-cell suspensions. Stained single-cell suspensions were assessed once more for concentration and viability using the Countess II Automated Cell Counter (Thermo Fisher Scientific) and captured using the 10x Chromium Controller (10x Genomics) with the Next GEM Single Cell 5ʹ Reagent Kit v2 (10x Genomics). Gel Bead-in-Emulsions were created from each sample, followed by cDNA amplification and enrichment for gene expression (GEX), feature barcoding (FB), and immune repertoire profiling of TCR and BCR library preparations. The resulting libraries were pooled and sequenced using NovaSeq (Illumina) targeting 40,000, 5,000, and 10,000 paired-end reads per cell for GEX, FB, and TCR/BCR libraries, respectively (read 1: 26, read 2: 90, index 1 and 2: 10). Secondary data analysis was performed using Cell Ranger software v4.0.0 (10x Genomics). GEX, TCR/BCR, and FB reads were mapped to appropriate regions and/or proteins using the 10x-compatible GRCh38-3.0.0, GRCh38-alts-4.0.0 references, and Total-Seq-C Human Universal barcoding file, respectively. Samples were evaluated across multiple quality metrics such as the fraction of reads in cells and reads mapped to the genome to ensure no outlying samples from the study batch. Additionally, the forms of barcode rank plots for each gene expression sample were reviewed to ensure automated cell-calling by Cell Ranger produced an appropriate delineation of cell-related and background barcodes.

#### Data processing and dimensionality reduction

Data processing and dimensionality reduction using principal component analysis (PCA), Uniform Manifold Approximation and Projection (UMAP), and Harmony (Korsunsky et al., 2019) were performed using the Seurat package (Hao et al., 2021). For RNA features, we calculated 5,000 of the most variable features prior to PCA. The top 30 PCs were inputted into UMAP or Harmony for batch-effect correction across vendors to generate 10 Harmony Components (HCs). All ADT features were used for PCA, UMAP, and Harmony unless otherwise specified for dimensionality reduction, clustering, or trajectory inference. Integration of RNA and ADT features for visualization was done using the weighted nearest-neighbor algorithm in Seurat using the provided vignette.

#### CITEseq clustering

Clustering was tested on RNA-only, ADT-only, and integrated features. RNA features were processed with PCA and Harmony as described above, and the 10 HCs were used to cluster the data. ADT-only features were similarly processed with harmony and the 10 HCs were used to cluster the data.

#### CITEseq trajectory analysis

Trajectory inference of T cells in the CITEseq data was performed using the principal curve algorithm in R, as similarly implemented in the SCORPIUS algorithm. Notably, this analysis does not mandate defining a start or end cell as input for the root population, which contrasts with many standard pseudotime analyses. Principal curve was run using a smooth spline smoother and 1,000 iterations. Trajectory inference was performed on 10 Harmony components generated using only the curated T cell–specific ADT features. Features were mean-centered and scaled prior to running Harmony. To statistically identify significant genes associated with the trajectory, as well as response, or treatment timepoint along the trajectory, we used a simple Gaussian link function with the generalized linear model (glm) function in the stats package of R to statistically compare trajectories in an interpretable linear model. Each gene was scaled and centered prior to statistical analysis. Vendor was included as a covariate in the model to control for batch effects. Multiple hypothesis correction was performed using Bonferroni correction across each analysis. Statistical significance of each gene was determined using a Bonferroni-adjusted value cutoff of 0.10 in combination with a coefficient magnitude cutoff of ±1 for assessing genes associated with the trajectory, and ±0.25 for comparing clinical response or pharmacodynamics. Statistical significance of genes associated with the trajectory in a response- or timepoint-dependent manner was determined by including response or timepoint as a parameter in the model, and the same Bonferroni-adjusted P value and coefficient cutoffs as above were used to determine whether response or timepoint had a significant effect on the estimating the trajectory in the glm.

### Online supplemental material

The supplemental information includes five figures, 15 tables, and one protocol. Fig. S1 shows the differential gene expression analysis on pretreatment tumor samples comparing CD8 converters versus non-converters in the CD8-low group. Fig. S2 shows CITEseq analysis on pretreatment PBMCs from six CD8 converters. Fig. S3 shows on-treatment tumor inflammatory signatures by response. Fig. S4 shows the CyTOF broad immune profile panel gating strategy. Fig. S5 shows the X50 T cell panel gating strategy. Table S1 shows prior systemic cancer therapies. Table S2 summarizes exposure to nivolumab and ipilimumab. Table S3 summarizes clinical outcomes. Table S4 compares disease characteristics and clinical outcomes between patients with and without an on-treatment biopsy. Table S5 summarizes the association between CD8 and clinical outcomes in the CD8-low group. Table S6 summarizes the association between prior ICI use and clinical outcomes. Table S7 summarizes clinical outcomes and CD8 conversion by tumor type in the CD8-low group. Table S8 summarizes TRAEs. Table S9 summarizes IRAEs. Table S10 lists treatment discontinuations owing to an AE in the CD8-low group. Table S11 lists sample size by translational assay, timepoint, and response in the CD8-low group. Table S12 lists the CyTOF antibody panel. Table S13 lists the X50 T cell phenotyping antibody panel. Table S14 lists the multiplex imaging staining panels. Table S15 lists the genes that are differentially expressed on treatment between CD8 converters and non-converters. Protocol contains the clinical study protocol and statistical analysis plan.

## Supplementary Material

Table S1 shows prior systemic cancer therapies with incidence ≥5%.

Table S2 shows exposure to nivolumab and ipilimumab.

Table S3 shows clinical activity.

Table S4 shows comparison between patients with and without an on-treatment biopsy in the CD8-low group.

Table S5 shows association of CD8 with clinical outcomes in the CD8-low group.

Table S6 shows association of prior immune checkpoint inhibitor therapy use with clinical outcomes.

Table S7 shows clinical outcomes and CD8 conversion by tumor type in the CD8-low group.

Table S8 shows TRAEs with incidence ≥10% in any group.

Table S9 shows IRAEs.

Table S10 shows treatment discontinuations owing to an AE in the nivolumab and ipilimumab group.

Table S11 shows sample size by assay, timepoint, and response in the CD8-low group.

Table S12 shows the CyTOF antibody panel.

Table S13 shows the T cell phenotyping antibody panel (X50).

Table S14 shows multiplex imaging staining panels.

Table S15 lists the genes that are differentially expressed on treatment between CD8 converters and non-converters.

Protocol contains the clinical study protocol and statistical analysis plan.

## Data Availability

Summary clinical and biomarker datasets are available at https://github.com/ParkerICI/amadeus-trial-data. Requests for additional data should be emailed to the corresponding author and should include a brief description of the proposed analysis. Data might be shared in the form of aggregate data summaries and may require a data transfer agreement, which will outline any potential restrictions on data use. Individual patient-level raw data containing confidential or identifiable patient information are subject to patient privacy and cannot be shared.

## References

[bib1] Assarsson, E., M. Lundberg, G. Holmquist, J. Björkesten, S.B. Thorsen, D. Ekman, A. Eriksson, E. Rennel Dickens, S. Ohlsson, G. Edfeldt, . 2014. Homogenous 96-plex PEA immunoassay exhibiting high sensitivity, specificity, and excellent scalability. PLoS One. 9:e95192. 10.1371/journal.pone.0095192 24755770 PMC3995906

[bib2] Beltra, J.-C., S. Manne, M.S. Abdel-Hakeem, M. Kurachi, J.R. Giles, Z. Chen, V. Casella, S.F. Ngiow, O. Khan, Y.J. Huang, . 2020. Developmental relationships of four exhausted CD8^+^ T cell subsets reveals underlying transcriptional and epigenetic landscape control mechanisms. Immunity. 52:825–841.e8. 10.1016/j.immuni.2020.04.014 32396847 PMC8360766

[bib3] Bratman, S.V., S.Y.C. Yang, M.A.J. Iafolla, Z. Liu, A.R. Hansen, P.L. Bedard, S. Lheureux, A. Spreafico, A.A. Razak, S. Shchegrova, . 2020. Personalized circulating tumor DNA analysis as a predictive biomarker in solid tumor patients treated with pembrolizumab. Nat. Cancer. 1:873–881. 10.1038/s43018-020-0096-5 35121950

[bib4] Cercek, A., M. Lumish, J. Sinopoli, J. Weiss, J. Shia, M. Lamendola-Essel, I.H. El Dika, N. Segal, M. Shcherba, R. Sugarman, . 2022. PD-1 blockade in mismatch repair-deficient, locally advanced rectal cancer. N. Engl. J. Med. 386:2363–2376. 10.1056/NEJMoa2201445 35660797 PMC9492301

[bib5] Chen, D.S., and I. Mellman. 2017. Elements of cancer immunity and the cancer-immune set point. Nature. 541:321–330. 10.1038/nature21349 28102259

[bib6] Chen, P.-L., W. Roh, A. Reuben, Z.A. Cooper, C.N. Spencer, P.A. Prieto, J.P. Miller, R.L. Bassett, V. Gopalakrishnan, K. Wani, . 2016. Analysis of immune signatures in longitudinal tumor samples yields insight into biomarkers of response and mechanisms of resistance to immune checkpoint blockade. Cancer Discov. 6:827–837. 10.1158/2159-8290.CD-15-1545 27301722 PMC5082984

[bib7] DePristo, M.A., E. Banks, R. Poplin, K.V. Garimella, J.R. Maguire, C. Hartl, A.A. Philippakis, G. del Angel, M.A. Rivas, M. Hanna, . 2011. A framework for variation discovery and genotyping using next-generation DNA sequencing data. Nat. Genet. 43:491–498. 10.1038/ng.806 21478889 PMC3083463

[bib8] Ferris, R.L., L. Licitra, J. Fayette, C. Even, G. Blumenschein Jr., K.J. Harrington, J. Guigay, E.E. Vokes, N.F. Saba, R. Haddad, . 2019. Nivolumab in patients with recurrent or metastatic squamous cell carcinoma of the head and neck: Efficacy and safety in CheckMate 141 by prior cetuximab use. Clin. Cancer Res. 25:5221–5230. 10.1158/1078-0432.CCR-18-3944 31239321 PMC7721346

[bib9] Gao, J., J.F. Ward, C.A. Pettaway, L.Z. Shi, S.K. Subudhi, L.M. Vence, H. Zhao, J. Chen, H. Chen, E. Efstathiou, . 2017. VISTA is an inhibitory immune checkpoint that is increased after ipilimumab therapy in patients with prostate cancer. Nat. Med. 23:551–555. 10.1038/nm.4308 28346412 PMC5466900

[bib10] Hao, Y., S. Hao, E. Andersen-Nissen, W.M. Mauck III, S. Zheng, A. Butler, M.J. Lee, A.J. Wilk, C. Darby, M. Zager, . 2021. Integrated analysis of multimodal single-cell data. Cell. 184:3573–3587.e29. 10.1016/j.cell.2021.04.048 34062119 PMC8238499

[bib11] Hartmann, F.J., J. Babdor, P.F. Gherardini, E.D. Amir, K. Jones, B. Sahaf, D.M. Marquez, P. Krutzik, E. O’Donnell, N. Sigal, . 2019. Comprehensive immune monitoring of clinical trials to advance human immunotherapy. Cell Rep. 28:819–831.e4. 10.1016/j.celrep.2019.06.049 31315057 PMC6656694

[bib12] Herbst, R.S., P. Baas, D.-W. Kim, E. Felip, J.L. Pérez-Gracia, J.-Y. Han, J. Molina, J.-H. Kim, C.D. Arvis, M.-J. Ahn, . 2016. Pembrolizumab versus docetaxel for previously treated, PD-L1-positive, advanced non-small-cell lung cancer (KEYNOTE-010): A randomised controlled trial. Lancet. 387:1540–1550. 10.1016/S0140-6736(15)01281-7 26712084

[bib13] Hodi, F.S., S.J. O’Day, D.F. McDermott, R.W. Weber, J.A. Sosman, J.B. Haanen, R. Gonzalez, C. Robert, D. Schadendorf, J.C. Hassel, . 2010. Improved survival with ipilimumab in patients with metastatic melanoma. N. Engl. J. Med. 363:711–723. 10.1056/NEJMoa1003466 20525992 PMC3549297

[bib14] Kitaura, K., T. Shini, T. Matsutani, and R. Suzuki. 2016. A new high-throughput sequencing method for determining diversity and similarity of T cell receptor (TCR) α and β repertoires and identifying potential new invariant TCR α chains. BMC Immunol. 17:38. 10.1186/s12865-016-0177-5 27729009 PMC5059964

[bib15] Korsunsky, I., N. Millard, J. Fan, K. Slowikowski, F. Zhang, K. Wei, Y. Baglaenko, M. Brenner, P.R. Loh, and S. Raychaudhuri. 2019. Fast, sensitive and accurate integration of single-cell data with Harmony. Nat. Methods. 16:1289–1296. 10.1038/s41592-019-0619-0 31740819 PMC6884693

[bib16] Kowanetz, M., W. Zou, S.N. Gettinger, H. Koeppen, M. Kockx, P. Schmid, E.E. Kadel III, I. Wistuba, J. Chaft, N.A. Rizvi, . 2018. Differential regulation of PD-L1 expression by immune and tumor cells in NSCLC and the response to treatment with atezolizumab (anti-PD-L1). Proc. Natl. Acad. Sci. USA. 115:E10119–E10126. 10.1073/pnas.1802166115 30297397 PMC6205493

[bib17] Kvistborg, P., D. Philips, S. Kelderman, L. Hageman, C. Ottensmeier, D. Joseph-Pietras, M.J.P. Welters, S. van der Burg, E. Kapiteijn, O. Michielin, . 2014. Anti-CTLA-4 therapy broadens the melanoma-reactive CD8^+^ T cell response. Sci. Transl. Med. 6:254ra128. 10.1126/scitranslmed.3008918 25232180

[bib18] Laino, A.S., D. Woods, M. Vassallo, X. Qian, H. Tang, M. Wind-Rotolo, and J. Weber. 2020. Serum interleukin-6 and C-reactive protein are associated with survival in melanoma patients receiving immune checkpoint inhibition. J. Immunother. Cancer. 8:e000842. 10.1136/jitc-2020-000842 32581042 PMC7312339

[bib19] Lei, Q., D. Wang, K. Sun, L. Wang, and Y. Zhang. 2020. Resistance mechanisms of anti-PD1/PDL1 therapy in solid tumors. Front. Cell Dev. Biol. 8:672. 10.3389/fcell.2020.00672 32793604 PMC7385189

[bib20] Litchfield, K., J.L. Reading, C. Puttick, K. Thakkar, C. Abbosh, R. Bentham, T.B.K. Watkins, R. Rosenthal, D. Biswas, A. Rowan, . 2021. Meta-analysis of tumor- and T cell-intrinsic mechanisms of sensitization to checkpoint inhibition. Cell. 184:596–614.e14. 10.1016/j.cell.2021.01.002 33508232 PMC7933824

[bib21] Maby, P., J. Galon, and J.-B. Latouche. 2015. Frameshift mutations, neoantigens and tumor-specific CD8(+) T cells in microsatellite unstable colorectal cancers. OncoImmunology. 5:e1115943. 10.1080/2162402X.2015.1115943 27467916 PMC4910722

[bib22] Marabelle, A., D.T. Le, P.A. Ascierto, A.M. Di Giacomo, A. De Jesus-Acosta, J.-P. Delord, R. Geva, M. Gottfried, N. Penel, A.R. Hansen, . 2020. Efficacy of pembrolizumab in patients with noncolorectal high microsatellite instability/mismatch repair-deficient cancer: Results from the phase II KEYNOTE-158 study. J. Clin. Oncol. 38:1–10. 10.1200/JCO.19.02105 31682550 PMC8184060

[bib23] McKenna, A., M. Hanna, E. Banks, A. Sivachenko, K. Cibulskis, A. Kernytsky, K. Garimella, D. Altshuler, S. Gabriel, M. Daly, and M.A. DePristo. 2010. The genome analysis Toolkit: A MapReduce framework for analyzing next-generation DNA sequencing data. Genome Res. 20:1297–1303. 10.1101/gr.107524.110 20644199 PMC2928508

[bib24] Peng, Y., L. Xiao, H. Rong, Z. Ou, T. Cai, N. Liu, B. Li, L. Zhang, F. Wu, T. Lan, . 2021. Single-cell profiling of tumor-infiltrating TCF1/TCF7^+^ T cells reveals a T lymphocyte subset associated with tertiary lymphoid structures/organs and a superior prognosis in oral cancer. Oral Oncol. 119:105348. 10.1016/j.oraloncology.2021.105348 34044317

[bib25] Powles, T., J.P. Eder, G.D. Fine, F.S. Braiteh, Y. Loriot, C. Cruz, J. Bellmunt, H.A. Burris, D.P. Petrylak, S.L. Teng, . 2014. MPDL3280A (anti-PD-L1) treatment leads to clinical activity in metastatic bladder cancer. Nature. 515:558–562. 10.1038/nature13904 25428503

[bib26] Reinert, T., T.V. Henriksen, E. Christensen, S. Sharma, R. Salari, H. Sethi, M. Knudsen, I. Nordentoft, H.-T. Wu, A.S. Tin, . 2019. Analysis of plasma cell-free DNA by ultradeep sequencing in patients with stages I to III colorectal cancer. JAMA Oncol. 5:1124–1131. 10.1001/jamaoncol.2019.0528 31070691 PMC6512280

[bib27] Rizvi, N.A., M.D. Hellmann, A. Snyder, P. Kvistborg, V. Makarov, J.J. Havel, W. Lee, J. Yuan, P. Wong, T.S. Ho, . 2015. Cancer immunology. Mutational landscape determines sensitivity to PD-1 blockade in non-small cell lung cancer. Science. 348:124–128. 10.1126/science.aaa1348 25765070 PMC4993154

[bib28] Rong, H., T. Cai, Y. Peng, X. Wang, T. Lan, Z. Ou, L. Qiu, Q. Li, L. Zhang, F. Wu, . 2022. Correlation between TCF7^+^ T cells and prognosis of patients with oral squamous cell carcinoma. Front. Oncol. 12:782058. 10.3389/fonc.2022.782058 35345446 PMC8957207

[bib29] Sade-Feldman, M., K. Yizhak, S.L. Bjorgaard, J.P. Ray, C.G. de Boer, R.W. Jenkins, D.J. Lieb, J.H. Chen, D.T. Frederick, M. Barzily-Rokni, . 2018. Defining T cell states associated with response to checkpoint immunotherapy in melanoma. Cell. 175:998–1013.e20. 10.1016/j.cell.2018.10.038 30388456 PMC6641984

[bib30] Sanmamed, M.F., J.L. Perez-Gracia, K.A. Schalper, J.P. Fusco, A. Gonzalez, M.E. Rodriguez-Ruiz, C. Oñate, G. Perez, C. Alfaro, S. Martín-Algarra, . 2017. Changes in serum interleukin-8 (IL-8) levels reflect and predict response to anti-PD-1 treatment in melanoma and non-small-cell lung cancer patients. Ann. Oncol. 28:1988–1995. 10.1093/annonc/mdx190 28595336 PMC5834104

[bib31] Scott, A.C., F. Dündar, P. Zumbo, S.S. Chandran, C.A. Klebanoff, M. Shakiba, P. Trivedi, L. Menocal, H. Appleby, S. Camara, . 2019. TOX is a critical regulator of tumour-specific T cell differentiation. Nature. 571:270–274. 10.1038/s41586-019-1324-y 31207604 PMC7698992

[bib32] Sharma, P., B.A. Siddiqui, S. Anandhan, S.S. Yadav, S.K. Subudhi, J. Gao, S. Goswami, and J.P. Allison. 2021. The next decade of immune checkpoint therapy. Cancer Discov. 11:838–857. 10.1158/2159-8290.CD-20-1680 33811120

[bib33] Shugay, M., D.V. Bagaev, I.V. Zvyagin, R.M. Vroomans, J.C. Crawford, G. Dolton, E.A. Komech, A.L. Sycheva, A.E. Koneva, E.S. Egorov, . 2018. VDJdb: A curated database of T-cell receptor sequences with known antigen specificity. Nucleic Acids Res. 46:D419–D427. 10.1093/nar/gkx760 28977646 PMC5753233

[bib34] Subudhi, S.K., A. Aparicio, J. Gao, A.J. Zurita, J.C. Araujo, C.J. Logothetis, S.A. Tahir, B.R. Korivi, R.S. Slack, L. Vence, . 2016. Clonal expansion of CD8 T cells in the systemic circulation precedes development of ipilimumab-induced toxicities. Proc. Natl. Acad. Sci. USA. 113:11919–11924. 10.1073/pnas.1611421113 27698113 PMC5081579

[bib35] Sznol, M., P.F. Ferrucci, D. Hogg, M.B. Atkins, P. Wolter, M. Guidoboni, C. Lebbé, J.M. Kirkwood, J. Schachter, G.A. Daniels, . 2017. Pooled analysis safety profile of nivolumab and ipilimumab combination therapy in patients with advanced melanoma. J. Clin. Oncol. 35:3815–3822. 10.1200/JCO.2016.72.1167 28915085

[bib36] Tawbi, H.A., D. Schadendorf, E.J. Lipson, P.A. Ascierto, L. Matamala, E. Castillo Gutiérrez, P. Rutkowski, H.J. Gogas, C.D. Lao, J.J. De Menezes, . 2022. Relatlimab and nivolumab versus nivolumab in untreated advanced melanoma. N. Engl. J. Med. 386:24–34. 10.1056/NEJMoa2109970 34986285 PMC9844513

[bib37] Thompson, E.D., M. Zahurak, A. Murphy, T. Cornish, N. Cuka, E. Abdelfatah, S. Yang, M. Duncan, N. Ahuja, J.M. Taube, . 2017. Patterns of PD-L1 expression and CD8 T cell infiltration in gastric adenocarcinomas and associated immune stroma. Gut. 66:794–801. 10.1136/gutjnl-2015-310839 26801886 PMC4958028

[bib38] Tickotsky, N., T. Sagiv, J. Prilusky, E. Shifrut, and N. Friedman. 2017. McPAS-TCR: A manually curated catalogue of pathology-associated T cell receptor sequences. Bioinformatics. 33:2924–2929. 10.1093/bioinformatics/btx286 28481982

[bib39] Wang, R., V. Baxi, Z. Li, D. Locke, C. Hedvat, Y. Sun, A.M. Walsh, X. Shao, T. Basavanhally, D.M. Greenawalt, . 2023. Pharmacodynamic activity of BMS-986156, a glucocorticoid-induced TNF receptor-related protein agonist, alone or in combination with nivolumab in patients with advanced solid tumors. ESMO Open. 8:100784. 10.1016/j.esmoop.2023.100784 36863094 PMC10163007

[bib40] Wei, S.C., C.R. Duffy, and J.P. Allison. 2018. Fundamental mechanisms of immune checkpoint blockade therapy. Cancer Discov. 8:1069–1086. 10.1158/2159-8290.CD-18-0367 30115704

[bib41] Wolchok, J.D., B. Neyns, G. Linette, S. Negrier, J. Lutzky, L. Thomas, W. Waterfield, D. Schadendorf, M. Smylie, T. Guthrie Jr., . 2010. Ipilimumab monotherapy in patients with pretreated advanced melanoma: A randomised, double-blind, multicentre, phase 2, dose-ranging study. Lancet Oncol. 11:155–164. 10.1016/S1470-2045(09)70334-1 20004617

