# Supplementary material for: Immunologic signatures of response and resistance to nivolumab with ipilimumab in advanced metastatic cancer
Source: J Exp Med. 2024 Aug 27;221(10):e20240152. doi: 10.1084/jem.20240152 (PMC11349049; doi:10.1084/jem.20240152)
Supplement: Table S1 — shows prior systemic cancer therapies with incidence ≥5%. [file JEM_20240152_TableS1.docx]

**Table S1. Prior systemic cancer therapies with incidence** $\boldsymbol{\geq}$ **5%.**

| **WHODrug Preferred Name, n (%)** | **Nivolumab**  **(N = 7)** | **Nivolumab + Ipilimumab**  **(N = 72)** | **Total**  **(N = 79)** |
| --- | --- | --- | --- |
| Paclitaxel | 2 (29) | 16 (22) | 18 (23) |
| Carboplatin | 2 (29) | 15 (21) | 17 (22) |
| Cisplatin | 3 (43) | 13 (18) | 16 (20) |
| Investigational antineoplastic drugs | 2 (29) | 13 (18) | 15 (19) |
| Bevacizumab | 1 (14) | 13 (18) | 14 (18) |
| Docetaxel | 2 (29) | 12 (17) | 14 (18) |
| Gemcitabine | 1 (14) | 10 (14) | 11 (14) |
| Fluorouracil; folinic acid; oxaliplatin | 1 (14) | 9 (13) | 10 (13) |
| Doxorubicin | 0 | 9 (13) | 9 (11) |
| Enzalutamide | 0 | 9 (13) | 9 (11) |
| Carboplatin; paclitaxel | 2 (29) | 7 (10) | 9 (11) |
| Calcium folinate; fluorouracil; irinotecan hydrochloride | 0 | 8 (11) | 8 (10) |
| Leuprorelin acetate | 0 | 8 (11) | 8 (10) |
| Nivolumab | 2 (29) | 6 (8) | 8 (10) |
| Abiraterone | 0 | 7 (10) | 7 (9) |
| Bicalutamide | 0 | 7 (10) | 7 (9) |
| Doxorubicin hydrochloride | 0 | 7 (10) | 7 (9) |
| Cetuximab | 3 (43) | 4 (6) | 7 (9) |
| Capecitabine | 0 | 6 (8) | 6 (8) |
| Ifosfamide | 1 (14) | 5 (7) | 6 (8) |
| Cabazitaxel | 0 | 5 (7) | 5 (6) |
| Docetaxel; gemcitabine | 0 | 5 (7) | 5 (6) |
| Etoposide | 0 | 5 (7) | 5 (6) |
| Cyclophosphamide | 1 (14) | 4 (6) | 5 (6) |
| Pazopanib hydrochloride | 1 (14) | 4 (6) | 5 (6) |
| Abiraterone acetate | 0 | 4 (6) | 4 (5) |
| Capecitabine; oxaliplatin | 0 | 4 (6) | 4 (5) |
| Carboplatin; docetaxel | 0 | 4 (6) | 4 (5) |
| Carboplatin; etoposide | 0 | 4 (6) | 4 (5) |
| Degarelix | 0 | 4 (6) | 4 (5) |
| Irinotecan | 0 | 4 (6) | 4 (5) |
| Leuprorelin | 0 | 4 (6) | 4 (5) |
| Pembrolizumab | 0 | 4 (6) | 4 (5) |
| Prednisone | 0 | 4 (6) | 4 (5) |
| Topotecan | 0 | 4 (6) | 4 (5) |
| Vinorelbine | 0 | 4 (6) | 4 (5) |
| Atezolizumab | 1 (14) | 3 (4) | 4 (5) |
| Fluorouracil | 2 (29) | 2 (3) | 4 (5) |

Prior therapies were coded using the WHODrug Global B3 (September 2021) dictionary.
