# Supplementary material for: Immunologic signatures of response and resistance to nivolumab with ipilimumab in advanced metastatic cancer
Source: J Exp Med. 2024 Aug 27;221(10):e20240152. doi: 10.1084/jem.20240152 (PMC11349049; doi:10.1084/jem.20240152)
Supplement: Table S2 — shows exposure to nivolumab and ipilimumab. [file JEM_20240152_TableS2.docx]

**Table S2. Exposure to nivolumab and ipilimumab.**

|  | **CD8-high**  **(N = 7)** | **CD8-low**  **(N = 72)** |
| --- | --- | --- |
| **Treatment exposure** |  |  |
| Treatment duration (months), median (range) | 3.8 (2.5 – 26.2) | 1.4 (0.0 – 42.8) |
| Interquartile Range | 3.5 – 5.7 | 0.7 – 4.4 |
| Nivolumab |  |  |
| Patients who received $\geq$1 dose, n (%) | 7 (100) | 72 (100) |
| Number of doses received, median (range) | 5.0 (3 – 29) | 3.0 (1 – 49) |
| Cumulative dose (mg), median (range) | 1,920 (1,080 – 13,440) | 1,080 (360 – 22,800) |
| Ipilimumab |  |  |
| Patients who received $\geq$1 dose, n (%) | 0 | 72 (100) |
| Number of doses received, n (%) |  |  |
| 1 | -- | 13 (18) |
| 2 | -- | 29 (40) |
| 3 | -- | 10 (14) |
| 4 | -- | 20 (28) |
| Cumulative dose (mg), median (range) | -- | 179.9 (40.8 – 485.2) |

Abbreviations: n or N = number.
