# Supplementary material for: Immunologic signatures of response and resistance to nivolumab with ipilimumab in advanced metastatic cancer
Source: J Exp Med. 2024 Aug 27;221(10):e20240152. doi: 10.1084/jem.20240152 (PMC11349049; doi:10.1084/jem.20240152)
Supplement: Table S3 — shows clinical activity. [file JEM_20240152_TableS3.docx]

**Table S3. Clinical activity.**

|  | **CD8-high**  **(N = 7)** | **CD8-low**  **(N = 72)** |
| --- | --- | --- |
| Best response, n (%) |  |  |
| Complete response (CR) | 0 | 1 (1.4) |
| Partial response (PR) | 1 (14.3) | 13 (18.1) |
| Stable disease (SD) | 2 (28.6) | 17 (23.6) |
| SD $\geq$ 6 months | 0 | 4 |
| SD < 6 months | 2 | 13 |
| Progressive disease | 4 (57.1) | 26 (36.1) |
| No available post-treatment tumor assessment^a^ | 0 | 15 (20.8) |
| Objective response rate, n (%) | 1 (14.3) | 14 (19.4) |
| 95% CI | 1.1 – 43.8 | 11.3 – 29.1 |
| Disease control rate^b^, n (%) | 1 (14.3) | 18 (25.0) |
| 95% CI | 1.1 – 43.8 | 15.8 – 35.2 |
| CD8 conversion from low to high^c^, n / number with on-treatment biopsy (%) | -- | 14 / 39 (35.9) |
| 95% CI | -- | 21.8 – 51.4 |
| CD8 conversion and CR or PR | -- | 6 / 14 (42.9) |
| CD8 conversion and CR, PR, or SD $\geq$ 6 months | -- | 7 / 14 (50.0) |
| Maximum absolute change of CD8 percentage from Baseline at any on-treatment biopsy |  |  |
| Number with on-treatment biopsy (%) | 4 (57.1) | 39 (54.2) |
| Median (range) | 11.5 (2 – 20) | 5.0 (-5 – 41) |
| Number of deaths (%) | 4 (57.1) | 39 (54.2) |
| Overall survival, months |  |  |
| Median (95% CI) | 15.8 (12.1 – NE) | 13.9 (8.9 – 21.1) |
| Progression-free survival, months |  |  |
| Median (95% CI) | 2.0 (1.1 – NE) | 2.3 (2.0 – 4.3) |

Abbreviations: CI = confidence/credible interval; CR = complete response; N or n = number; NE = not estimable; PR = partial response; SD = stable disease.

^a^ Reasons for patients not having any post-baseline tumor assessments include death (n=6), inability due to clinical deterioration (n=5), and withdrawal of consent / lost to follow-up (n=4).

^b^ Disease control rate is defined as the proportion of patients with a best overall response of confirmed complete or partial response or stable disease at least 6 months after study drug initiation.

^c^ CD8 conversion from low to high is defined as participants that had a tumor biopsy of CD8-low (< 15%) at screening and CD8-high ($\geq$ 15%) at any on-treatment biopsy.
