# Supplementary material for: Immunologic signatures of response and resistance to nivolumab with ipilimumab in advanced metastatic cancer
Source: J Exp Med. 2024 Aug 27;221(10):e20240152. doi: 10.1084/jem.20240152 (PMC11349049; doi:10.1084/jem.20240152)
Supplement: Table S4 — shows comparison between patients with and without an on-treatment biopsy in the CD8-low group. [file JEM_20240152_TableS4.docx]

**Table S4. Comparison between patients with and without an on-treatment biopsy in the CD8-low group.**

|  | **Patients with**  **on-treatment biopsy**  **(N = 39)** | **Patients without**  **on-treatment biopsy**  **(N = 33)** |
| --- | --- | --- |
| CD8 Cells (%) at Screening |  |  |
| Median (IQR) | 1.0 (0 – 5.0) | 6.0 (3.0 – 11.0) |
| Tumor Type |  |  |
| Prostate (CRPC) | 6 (15.4) | 6 (18.2) |
| Head and Neck (HNCA) | 4 (10.3) | 2 (6.1) |
| Colorectal (CRCA) | 2 (5.1) | 5 (15.2) |
| Sarcoma (SARC) | 5 (12.8) | 2 (6.1) |
| Ovarian (OVCA) | 4 (10.3) | 1 (3.0) |
| Uterine (UTCA) | 2 (5.1) | 2 (6.1) |
| Breast (BRCA) | 1 (2.6) | 2 (6.1) |
| Hepatocellular cholangiocarcinoma (HECH) | 2 (5.1) | 1 (3.0) |
| Neuroendocrine (NEUC) | 2 (5.1) | 1 (3.0) |
| Renal (RNCA) | 1 (2.6) | 1 (3.0) |
| Thyroid (THYR) | 2 (5.1) | 1 (3.0) |
| Other | 8 (20.5) | 9 (27.3) |
| Prior Lines of Cancer Therapy |  |  |
| 0 | 2 (5.1) | 1 (3.0) |
| 1-2 | 12 (30.8) | 6 (18.2) |
| 3-4 | 10 (25.6) | 15 (45.5) |
| 5+ | 15 (38.5) | 11 (33.3) |
| Received Prior ICI Therapy, n (%) | 6 (15.4) | 9 (27.3) |
| Treatment duration (months), median (IQR) | 2.8 (1.4 – 5.3) | 0.7 (0.0 – 1.7) |
| Best response, n (%) |  |  |
| Complete response (CR) | 1 (2.6) | 0 |
| Partial response (PR) | 8 (20.5) | 5 (15.2) |
| Stable disease (SD) | 11 (28.2) | 6 (18.2) |
| SD $\geq$ 6 months | 3 | 1 |
| SD < 6 months | 8 | 5 |
| Progressive disease | 16 (41.0) | 10 (30.3) |
| No available post-treatment tumor assessment | 3 (7.7) | 12 (36.4) |
| Objective response rate | 23.1% (9 / 39) | 15.2% (5 / 33) |
| Disease control rate | 30.8% (12 / 39) | 18.2% (6 / 33) |

Abbreviations: CR = complete response; ICI = immune checkpoint inhibitor; IQR = interquartile range; PR = partial response; SD = stable disease.
