# Supplementary material for: Immunologic signatures of response and resistance to nivolumab with ipilimumab in advanced metastatic cancer
Source: J Exp Med. 2024 Aug 27;221(10):e20240152. doi: 10.1084/jem.20240152 (PMC11349049; doi:10.1084/jem.20240152)
Supplement: Table S5 — shows association of CD8 with clinical outcomes in the CD8-low group. [file JEM_20240152_TableS5.docx]

**Table S5. Association of CD8 with clinical outcomes in the CD8-low group.**

| **Explanatory Variable** | **Response Variable** | **Analysis Population** | **N** | **Odds Ratio (95% CI)** | **p-value** |
| --- | --- | --- | --- | --- | --- |
| Baseline percentage of CD8 cells | DCR | mITT Population | 72 | 0.94 (0.83 – 1.08) | 0.375 |
|  | ORR | mITT Population | 72 | 0.97 (0.84 – 1.12) | 0.676 |
| Maximum percentage of CD8 cells across all on-treatment biopsies | DCR | On-treatment Biopsy Population | 39 | 1.04 (0.99 – 1.10) | 0.136 |
|  | ORR | On-treatment Biopsy Population | 39 | 1.06 (1.00 – 1.12) | 0.055 |
| Change between baseline and maximum percentage of CD8 cells across all on-treatment biopsies | DCR | On-treatment Biopsy Population | 39 | 1.05 (0.99 – 1.11) | 0.119 |
|  | ORR | On-treatment Biopsy Population | 39 | 1.06 (1.00 – 1.13) | 0.063 |
| CD8 conversion from low to high (binary variable) | DCR | On-treatment Biopsy Population | 39 | 4.00 (0.95 – 16.79) | 0.058 |
|  | ORR | On-treatment Biopsy Population | 39 | 5.50 (1.11 – 27.37) | 0.037 |

Abbreviations: CI = confidence interval; DCR = disease control rate; N = sample size; ORR = objective response rate.

This table displays results from multiple logistic regression models that were fit to assess the relationship between the percentage of CD8 cells and clinical response (DCR or ORR) in the CD8-low group. Each regression model included an intercept term. P-values were calculated using a Wald test, which tests whether the coefficient for the CD8 variable is significantly different from zero.
