# Supplementary material for: Immunologic signatures of response and resistance to nivolumab with ipilimumab in advanced metastatic cancer
Source: J Exp Med. 2024 Aug 27;221(10):e20240152. doi: 10.1084/jem.20240152 (PMC11349049; doi:10.1084/jem.20240152)
Supplement: Table S6 — shows association of prior immune checkpoint inhibitor therapy use with clinical outcomes. [file JEM_20240152_TableS6.docx]

**Table S6. Association of prior immune checkpoint inhibitor therapy use with clinical outcomes.**

| **Response Variable** | **Treatment Group** | **Response Rate in Patients with Prior ICI Therapy** | **Response Rate in Patients without Prior ICI Therapy** | **Odds Ratio (95% CI)** | **p-value** |
| --- | --- | --- | --- | --- | --- |
| Objective Response Rate (ORR) | CD8-high | 0% (0/3) | 25% (1/4) | < 0.01 (NE, NE) | 0.998 |
|  | CD8-low | 6.7% (1/15) | 22.8% (13/57) | 0.24 (0.03 – 2.02) | 0.190 |
| Disease Control Rate (DCR) | CD8-high | 0% (0/3) | 25% (1/4) | < 0.01 (NE, NE) | 0.998 |
|  | CD8-low | 13.3% (2/15) | 28.1% (16/57) | 0.39 (0.08 – 1.95) | 0.253 |
| CD8 conversion from low to high (binary variable) | CD8-low | 16.7% (1/6) | 39.4% (13/33) | 0.31 (0.03 – 2.94) | 0.306 |

Abbreviations: CI = confidence interval; DCR = disease control rate; NE = not estimable; ICI = immune checkpoint inhibitor; ORR = objective response rate.

This table displays results from multiple logistic regression models that were fit to assess the relationship between prior ICI use and clinical response (DCR or ORR) or CD8 conversion from low (< 15%) to high ($\geq$ 15%). Each regression model included an intercept term. P-values were calculated using a Wald test, which tests whether the coefficient for the prior ICI variable is significantly different from zero.
