# Supplementary material for: Immunologic signatures of response and resistance to nivolumab with ipilimumab in advanced metastatic cancer
Source: J Exp Med. 2024 Aug 27;221(10):e20240152. doi: 10.1084/jem.20240152 (PMC11349049; doi:10.1084/jem.20240152)
Supplement: Table S7 — shows clinical outcomes and CD8 conversion by tumor type in the CD8-low group. [file JEM_20240152_TableS7.docx]

**Table S7. Clinical outcomes and CD8 conversion by tumor type in the CD8-low group.**

| **Tumor Type** | **No. with on-treatment biopsy / N** | **ORR** | **DCR** | **CD8 Conversion from Low to High** | **ORR within Patients with CD8 Conversion** |
| --- | --- | --- | --- | --- | --- |
| Prostate | 6 / 12 | 16.7% (2/12) | 33.3% (4/12) | 50.0% (3/6) | 33.3% (1/3) |
| Colorectal | 2 / 7 | 0% (0/7) | 14.3% (1/7) | 0% (0/2) | -- |
| Sarcoma | 5 / 7 | 0% (0/7) | 0% (0/7) | 60.0% (3/5) | 0% (0/3) |
| Head and Neck | 4 / 6 | 0% (0/6) | 0% (0/6) | 0% (0/5) | -- |
| Ovarian | 4 / 5 | 60.0% (3/5) | 60.0% (3/5) | 75.0% (3/4) | 66.7% (2/3) |
| Uterine | 2 / 4 | 50.0% (2/4) | 50.0% (2/4) | 50.0% (1/2) | 100% (1/1) |
| Breast | 1 / 3 | 0% (0/3) | 0% (0/3) | 0% (0/1) | -- |
| Hepatocellular Cholangiocarcinoma | 2 / 3 | 0% (0/3) | 0% (0/3) | 0% (0/2) | -- |
| Neuroendocrine | 2 / 3 | 66.7% (2/3) | 66.7% (2/3) | 50.0% (1/2) | 100% (1/1) |
| Thyroid | 2 / 3 | 33.3% (1/3) | 33.3% (1/3) | 0% (0/2) | -- |

Abbreviations: DCR = disease control rate; N = sample size; No. = number; ORR = objective response rate.

CD8 Conversion from Low to High is defined as having a tumor biopsy of CD8-low (< 15%) at screening and CD8-high ($\geq$ 15%) at any on-treatment biopsy.
