# Supplementary material for: Immunologic signatures of response and resistance to nivolumab with ipilimumab in advanced metastatic cancer
Source: J Exp Med. 2024 Aug 27;221(10):e20240152. doi: 10.1084/jem.20240152 (PMC11349049; doi:10.1084/jem.20240152)
Supplement: Table S8 — shows TRAEs with incidence ≥10% in any group. [file JEM_20240152_TableS8.docx]

**Table S8. Treatment-related adverse events with incidence** $\boldsymbol{\geq}$ **10% in any group.**

|  | **Nivolumab**  **(N = 7)** | | **Nivolumab + Ipilimumab**  **(N = 72)** | |
| --- | --- | --- | --- | --- |
| **MedDRA (v 25.0) Preferred Term, n (%)** | **Any Grade** | **Grade 3-4** | **Any Grade** | **Grade 3-4** |
| Fatigue | 0 | 0 | 28 (39) | 2 (3) |
| Diarrhea | 0 | 0 | 15 (21) | 1 (1) |
| Nausea | 0 | 0 | 12 (17) | 2 (3) |
| Pruritus | 1 (14) | 0 | 11 (15) | 0 |
| Rash | 0 | 0 | 11 (15) | 0 |
| Decreased appetite | 0 | 0 | 10 (14) | 1 (1) |
| Lipase increased | 0 | 0 | 10 (14) | 6 (8) |
| Anemia | 0 | 0 | 9 (13) | 2 (3) |
| Hypothyroidism | 1 (14) | 0 | 8 (11) | 0 |
| Vomiting | 1 (14) | 0 | 4 (6) | 1 (1) |
| Abdominal pain | 1 (14) | 0 | 3 (4) | 1 (1) |
| Hyperthyroidism | 1 (14) | 0 | 3 (4) | 0 |
| Arthralgia | 1 (14) | 0 | 2 (3) | 0 |
| Blood lactate dehydrogenase increased | 1 (14) | 0 | 1 (1) | 0 |
| Hypophysitis | 1 (14) | 0 | 1 (1) | 1 (1) |
