# Supplementary material for: Immunologic signatures of response and resistance to nivolumab with ipilimumab in advanced metastatic cancer
Source: J Exp Med. 2024 Aug 27;221(10):e20240152. doi: 10.1084/jem.20240152 (PMC11349049; doi:10.1084/jem.20240152)
Supplement: Table S9 — shows IRAEs. [file JEM_20240152_TableS9.docx]

**Table S9. Immune-related adverse events.**

|  | **Nivolumab**  **(N = 7)** | | **Nivolumab + Ipilimumab**  **(N = 72)** | |
| --- | --- | --- | --- | --- |
| **MedDRA (v 25.0) Preferred Term, n (%)** | **Any Grade** | **Grade 3** | **Any Grade** | **Grade 3** |
| Any immune-mediated adverse event | 2 (29) | 0 | 42 (58) | 7 (10) |
| Diarrhea | 0 | 0 | 15 (21) | 1 (1) |
| Pruritus | 1 (14) | 0 | 11 (15) | 0 |
| Rash | 0 | 0 | 11 (15) | 0 |
| Hypothyroidism | 1 (14) | 0 | 8 (11) | 0 |
| Aspartate aminotransferase increased | 0 | 0 | 7 (10) | 1 (1) |
| Alanine aminotransferase increased | 0 | 0 | 6 (8) | 1 (1) |
| Hyperthyroidism | 1 (14) | 0 | 3 (4) | 0 |
| Rash maculo-papular | 0 | 0 | 4 (6) | 1 (1) |
| Adrenal insufficiency | 0 | 0 | 3 (4) | 0 |
| Thyroiditis | 0 | 0 | 3 (4) | 2 (3) |
| Blood thyroid stimulating hormone increased | 0 | 0 | 2 (3) | 0 |
| Hypophysitis | 1 (14) | 0 | 1 (1) | 1 (1) |
| Autoimmune hepatitis | 0 | 0 | 1 (1) | 0 |
| Blood creatinine increased | 0 | 0 | 1 (1) | 0 |
| Colitis | 0 | 0 | 1 (1) | 1 (1) |
| Myositis | 0 | 0 | 1 (1) | 0 |
| Rash erythematous | 0 | 0 | 1 (1) | 0 |
| Rash macular | 0 | 0 | 1 (1) | 0 |
| Renal failure | 0 | 0 | 1 (1) | 0 |
| Rhabdomyolysis | 0 | 0 | 1 (1) | 1 (1) |

Abbreviations: MedDRA = Medical Dictionary for Regulatory Activities; n or N = number.

No grade 4 or grade 5 immune-related adverse events (IRAEs) were reported in either group.

IRAE terms were identified through manual medical review. All IRAEs had to be assessed by the investigator as being at least possibly related to either nivolumab or ipilimumab.
