# Supplementary material for: Immunologic signatures of response and resistance to nivolumab with ipilimumab in advanced metastatic cancer
Source: J Exp Med. 2024 Aug 27;221(10):e20240152. doi: 10.1084/jem.20240152 (PMC11349049; doi:10.1084/jem.20240152)
Supplement: Table S10 — shows treatment discontinuations owing to an AE in the nivolumab and ipilimumab group. [file JEM_20240152_TableS10.docx]

**Table S10. Treatment discontinuations owing to an adverse event in the nivolumab and ipilimumab group.**

| **MedDRA (v 25.0) Preferred Term** | **Toxicity Grade** | **Study Drug(s) Assessed as**  **Related per Investigator** |
| --- | --- | --- |
| Alanine aminotransferase increased | Grade 3 | Ipilimumab, Nivolumab |
| Anaemia | Grade 3 | Unrelated |
| Blood Creatinine Increased | Grade 2 | Unrelated |
| Dyspnoea | Grade 2 | Unrelated |
| Lipase increased | Grade 3 | Ipilimumab, Nivolumab |
| Lipase increased | Grade 3 | Unrelated |
| Papilloedema | Grade 3 | Ipilimumab, Nivolumab |
| Retinal vascular disorder | Grade 2 | Ipilimumab, Nivolumab |
| Rhabdomyolosis | Grade 3 | Ipilimumab, Nivolumab |

Abbreviations: MedDRA = Medical Dictionary for Regulatory Activities.

No treatment discontinuations owing to an adverse event were identified in the nivolumab group.
