# Supplementary material for: Immunologic signatures of response and resistance to nivolumab with ipilimumab in advanced metastatic cancer
Source: J Exp Med. 2024 Aug 27;221(10):e20240152. doi: 10.1084/jem.20240152 (PMC11349049; doi:10.1084/jem.20240152)
Supplement: Table S11 — shows sample size by assay, timepoint, and response in the CD8-low group. [file JEM_20240152_TableS11.docx]

**Table S11. Sample size by assay, time point, and response in the CD8-low group.**

| **Assay** | **Time Point** | **Number of Responders** | **Number of**  **Non-Responders** | **Number of CD8 Converters** | **Number of CD8**  **Non-converters** | **Total Patients** |
| --- | --- | --- | --- | --- | --- | --- |
| CD8 IHC | Baseline | 14 | 58 | 14 | 25 | 72 |
|  | On-treatment | 9 | 30 | 14 | 25 | 39 |
| RNAseq | Baseline | 7 | 25 | 7 | 14 | 32 |
|  | On-treatment | 6 | 17 | 11 | 12 | 23 |
| Whole exome sequencing (MSI & TMB) | Baseline | 8 | 32 | 9 | 16 | 40 |
| Vectra mIF imaging: Panel A | Baseline | 13 | 43 | 13 | 19 | 56 |
|  | On-treatment | 6 | 23 | 8 | 21 | 29 |
| Vectra mIF imaging: Panel B | Baseline | 14 | 46 | 13 | 22 | 60 |
|  | On-treatment | 6 | 24 | 9 | 21 | 30 |
| Vectra mIF imaging: Panel C | Baseline | 12 | 37 | 13 | 14 | 49 |
|  | On-treatment | 6 | 16 | 9 | 13 | 22 |
| X50 flow cytometry | Baseline | 14 | 56 | 14 | 24 | 70 |
| CyTOF | Baseline  (Cycle 1 Day 1) | 10 | 47 | 17 | 12 | 57 |
|  | Cycle 1 Day 8 | 11 | 46 | 21 | 12 | 57 |
|  | Cycle 2 Day 1 | 10 | 45 | 22 | 13 | 55 |
|  | Cycle 3 Day 1 | 9 | 31 | 16 | 12 | 40 |
| CITEseq | Baseline  (Cycle 1 Day 1) | 3 | 3 | 6 | 0 | 6 |
|  | Cycle 1 Day 8 | 3 | 3 | 6 | 0 | 6 |
| T cell receptor (TCR) sequencing | Baseline | 7 | 15 | 7 | 9 | 22 |
|  | On-treatment | 7 | 8 | 10 | 5 | 15 |
| Serum proteomics | Baseline  (Cycle 1 Day 1) | 14 | 53 | 13 | 23 | 67 |
|  | Cycle 1 Day 8 | 14 | 48 | 12 | 23 | 62 |
|  | Cycle 2 Day 1 | 13 | 43 | 13 | 23 | 56 |
|  | Cycle 3 Day 1 | 11 | 33 | 12 | 19 | 44 |
|  | Cycle 4 Day 1 | 12 | 16 | 10 | 13 | 28 |
| Circulating tumor DNA (ctDNA) | Baseline  (Cycle 1 Day 1) | 8 | 17 | 9 | 10 | 25 |
|  | Cycle 2 Day 1 | 7 | 16 | 9 | 10 | 23 |

Abbreviations: CITEseq = cellular indexing of transcriptomes and epitomes by sequencing; CyTOF = cytometry by time of flight; mIF = multiplex immunofluorescence; MSI = microsatellite instability; TMB = tumor mutational burden.
