# Supplementary material for: Immunologic signatures of response and resistance to nivolumab with ipilimumab in advanced metastatic cancer
Source: J Exp Med. 2024 Aug 27;221(10):e20240152. doi: 10.1084/jem.20240152 (PMC11349049; doi:10.1084/jem.20240152)
Supplement: Table S12 — shows the CyTOF antibody panel. [file JEM_20240152_TableS12.docx]

**Table S12. CyTOF antibody panel.**

| **Mass** | **Element** | **Target** | **Clone** | **Source** | **Cat #** | **Biology** | **Staining** |
| --- | --- | --- | --- | --- | --- | --- | --- |
| 89 | Y | CD45 | H130 | Fluidigm | 3089003B | Pan | Surface |
| 113 | In | CD8a | RPA-T8 | Fluidigm | 3146001B | T cell subset/NK | surface |
| 115 | In | Ki67 | B56 | Fluidigm | 3168007B | Proliferation | Intra-cellular |
| 140 | Ce | CD86 | IT2.2 | BioLegend | Custom | T cell costimulation | surface |
| 141 | Pr | CD3 | UCHT1 | Fluidigm | 3141019B | Pan T cells | surface |
| 142 | Nd | CD19 | HIB19 | Fluidigm | 3142001B | Pan B cells | surface |
| 143 | Nd | CD117  (c-kit) | 104D2 | Fluidigm | 3143001B | Mast cells primitive immune | surface |
| 144 | Nd | CD11b | IRCF44 | Fluidigm | 3144001B | Macrophage/monocyte | surface |
| 145 | Nd | CD4 | RPA-T4 | Fluidigm | 3145001B | T cell subset/monocyte | surface |
| 146 | Nd | CD69 | FN50 | BioLegend | Custom | T cell subset | surface |
| 147 | Sm | CD11c | BU15 | Fluidigm | 3147008B | DC/macrophage/monocyte | surface |
| 148 | Nd | CD14 | RMO52 | Fluidigm | 3148010B | Macrophage/monocyte | surface |
| 149 | Sm | CD1c (BDCA1) | L161 | BioLegend | Custom | DC | surface |
| 150 | Nd | FcER1 | AER-37 | Fluidigm | 3150027B | IgE Receptor (mast/basophil) | surface |
| 151 | Eu | CD123  (IL-3Ra) | 6H6 | Fluidigm | 3151001B | B cell sub/DC/Baso/pDC | surface |
| 152 | Sm | gdTCR | 11F2 | Fluidigm | 3152008B | gd T cell | surface |
| 153 | Eu | CD45RA | HI100 | Fluidigm | 3153001B | T cell naïve/memory | surface |
| 154 | Sm | CD366 (TIM3) | F38-2E2 | Fluidigm | 3154010B | Th1 polarization | surface |
| 155 | Gd | CD64 | 10.1 | BioLegend | Custom | FcgammaRI | surface |
| 156 | Gd | CD274 (PD-L1) | 29E.2A3 | Fluidigm | 3156026B | Checkpoint | surface |
| 157 | Gd | CD39 | A1 | BioLegend | Custom | T cell subset | surface |
| 158 | Gd | CD27 | L128 | Fluidigm | 3158010B | B/T cell memory | surface |
| 159 | Tb | CD141 | 1A4 | BioLegend | Custom | mDC | surface |
| 160 | Gd | Tbet | 4B10 | Fluidigm | 3160010B | Th1 polarization/NK | Intra-cellular |
| 161 | Dy | CD152 (CTLA-4) | 14D3 | Fluidigm | 3161004B | Checkpoint | Intra-cellular |
| 162 | Dy | Foxp3 | PCH101 | Fluidigm | 3162011A | Treg | Intra-cellular |
| 163 | Dy | CD33 | WM53 | Fluidigm | 3163023B | Pan myeloid | surface |
| 164 | Dy | CD45RO | UCHL1 | Fluidigm | 3164007B | T cell naïve/memory | surface |
| 165 | Ho | CD127  (IL-7Ra) | A019D5 | Fluidigm | 3165008B | T cell subset/Treg | surface |
| 166 | Er | CD154 (CD40L) | 24-31 | BioLegend | Custom | T cell activation | surface |
| 167 | Er | CCR7 (CD197) | G043H7 | Fluidigm | 3167009A | T cell subset (eff/memory) | surface |
| 168 | Er | ICOS (CD278) | C398.4A | Fluidigm | 3168024B | T cell activation | surface |
| 169 | Tm | CD25 | 2A3 | Fluidigm | 3169003B | Treg | surface |
| 170 | Er | TCR  Va24-Ja18  (iNKT cell) | 6B11 | Fluidigm | 3170015B | iNKT | surface |
| 171 | Yb | CD40 | 5C3 | BioLegend | Custom | APC | surface |
| 172 | Yb | CD38 | H1T2 | Fluidigm | 3172007B | B cell/NK/plasma | surface |
| 173 | Yb | CD192 (CCR2) | K036C2 | BioLegend | Custom | CCR2 Chemokine Receptor | surface |
| 174 | Yb | HLA-DR | L243 | Fluidigm | 3174001B | APC | surface |
| 175 | Lu | PD-1 (Nivo)/ anti-IgG4 | HP6025 | Southern Bio | Custom | Checkpoint | surface |
| 176 | Yb | CD56 | NCAM16.2 | Fluidigm | 3176088B | NK | surface |
| 209 | Bi | CD16 | 3G8 | Fluidigm | 3209002B | Fc Receptor/NK/ Neutrophil/Monocyte | surface |
