# Supplementary material for: Immunologic signatures of response and resistance to nivolumab with ipilimumab in advanced metastatic cancer
Source: J Exp Med. 2024 Aug 27;221(10):e20240152. doi: 10.1084/jem.20240152 (PMC11349049; doi:10.1084/jem.20240152)
Supplement: Table S13 — shows the T cell phenotyping antibody panel (X50). [file JEM_20240152_TableS13.docx]

**Table S13. T cell phenotyping antibody panel (X50).**

| **Fluorophore** | **Target** | **Clone** | **Source** | **Cat #** | **Category** | **Staining** |
| --- | --- | --- | --- | --- | --- | --- |
| BUV395 | CD45RA | HL100 | BD | 740298 | Differentiation | Surface |
| BUV496 | CD8a | RPA-T8 | BD | 612942 | Lineage | Surface |
| BUV563 | CD185 (CXCR5) | RF8B2 | BD | 741316 | Lineage | Surface |
| BUV615 | CD25 | 2A3 | BD | 612996 | Lineage/ activation | Surface |
| BUV661 | CD226 (DNAM-1) | DX11 | BD | 749934 | Activation | Surface |
| BUV737 | CD27 | L128 | BD | 612829 | Differentiation | Surface |
| BUV805 | CD4 | SK3 | BD | 612887 | Lineage | Surface |
| BV421 | CD197 (CCR7) | G043H7 | Biolegend | 353208 | Differentiation | Surface |
| BV480 | CD223 (LAG3) | T47-530 | BD | 746609 | Exhaustion | Surface |
| BV510 | Fixable Viability Stain (FVS) | n/a | BD | 564406 | Dump | Surface |
| BV510 | CD14 | M5E2 | BD | 740163 | Dump | Surface |
| BV510 | CD19 | SJ25C1 | BD | 562947 | Dump | Surface |
| BV510 | CD41a | HIP8 | BD | 563250 | Dump | Surface |
| BV570 | CD3 | UCHT1 | Biolegend | 300436 | Lineage | Surface |
| BV605 | CD137 (4-1BB) | 4B4-1 | BD | 745256 | Activation | Surface |
| BV650 | CD244 (2B4) | 2-69 | BD | custom | Activation/ exhaustion | Surface |
| BV711 | CD366 (Tim3) | 7D3 | BD | 565566 | Exhaustion | Surface |
| BV750 | CD39 | TU66 | BD | 747079 | Exhaustion | Surface |
| BV786 | CD28 | CD28.2 | BD | 740996 | Differentiation | Surface |
| BB515 | CD279 (PD-1) + anti-Human IgG4 | nivolumab, G17-4 | Selleck Chem; BD | A2002; custom | Activation/ exhaustion | Surface |
| BB660 | CD278 (ICOS) | DX29 | BD | custom | Activation | Surface |
| BB700 | CD127 (IL-7Ra) | HlL-7R-M21 | BD | 566398 | Differentiation | Surface |
| BB790 | CD38 | HIT2 | BD | custom | Differentiation/ activation | Surface |
| PE | TIGIT | MBSA43 | eBioscience | 12-9500-42 | Exhaustion | Surface |
| PE-eFluor610 | Eomes | WD1928 | eBioscience | 61-4877-42 | Differentiation/ exhaustion | Intra-cellular |
| PE-Cy5 | CD152 (CTLA-4) | BNI3 | BD | 555854 | Exhaustion | Intra-cellular |
| PE-Cy5.5 | FoxP3 | PCH101 | eBioscience | 35-4776-42 | Lineage | Intra-cellular |
| PE-Cy7 | T-bet | O4-46 | BD | custom | Lineage/activation | Intra-cellular |
| AF647 | TCF-1 (TCF7) | S33-966 | BD | 566693 | Differentiation | Intra-cellular |
| AF700 | Ki-67 | B56 | BD | 561277 | Proliferation | Intra-cellular |
| APC-Fire750 | KLRG1 | SA231A2 | Biolegend | 367718 | Exhaustion | Surface |
