# Supplementary material for: Immunologic signatures of response and resistance to nivolumab with ipilimumab in advanced metastatic cancer
Source: J Exp Med. 2024 Aug 27;221(10):e20240152. doi: 10.1084/jem.20240152 (PMC11349049; doi:10.1084/jem.20240152)
Supplement: Table S14 — shows multiplex imaging staining panels. [file JEM_20240152_TableS14.docx]

**Table S14. Multiplex imaging staining panels.**

| **Panel A** | | | | | |
| --- | --- | --- | --- | --- | --- |
| **Marker** | **Antibody clone** | **Source** | | **Dilution** | **Detection Dye (cycle)** |
| CD3 | BC33 | Biocare | | 1:200 | Opal 520 (1) |
| Ki67 | SP6 | Biocare | | 1:100 | Opal 540 (2) |
| CD56 | MRQ-42 | CellMarque | | 1:2 | Opal 570 (3) |
| FOXP3 | 236A/E7 | Biocare | | 1:2 | Opal 620 (4) |
| CD8 | C8/114B | CST | | 0.042 ug/mL | Opal 650 (5) |
| panCK | AE1/AE3 | DAKO | | 0.665 ug/mL | Opal 690 (6) |
| **Panel B** | | | | | |
| **Marker** | **Antibody clone** | | **Source** | **Dilution** | **Detection Dye (cycle)** |
| CD80 | EPR1157(2) | | Abcam | 1:500 | Opal 520 (1) |
| INOS | 13F5.1 | | Millipore | 1:2500 | Opal 540 (2) |
| CD68 | PG-M1 | | Dako | 0.15ug/mL | Opal 570 (3) |
| CD20 | E7B7T | | CST | 0.055ug/mL | Opal 620 (4) |
| PDL1 | 73-10 | | Abcam | 0.18ug/mL | Opal 650 (5) |
| panCK | AE1/AE3 | | DAKO | 0.665 ug/mL | Opal 690 (6) |
| **Panel C** | | | | | |
| **Marker** | **Antibody clone** | | **Source** | **Dilution** | **Detection Dye (cycle)** |
| PD1 | EPR4877(2) | | Abcam | 5ug/mL | Opal 520 (1) |
| CD68 | PG-M1 | | DAKO | 0.15ug/mL | Opal 540 (2) |
| CD163 | 10D6 | | Leica | 0.375ug/mL | Opal 540 (2) |
| TCF1/TCF7 | C63D9 | | CST | 0.985ug/mL | Opal 570 (3) |
| CD4 | EPR6855 | | Abcam | 0.7ug/mL | Opal 620 (4) |
| CD8 | C8/114B | | CST | 0.042ug/mL | Opal 650 (5) |
| TOX | E6I3Q | | CST | 0.644ug/mL | Opal 690 (6) |
