# Supplementary material for: Immunologic signatures of response and resistance to nivolumab with ipilimumab in advanced metastatic cancer
Source: J Exp Med. 2024 Aug 27;221(10):e20240152. doi: 10.1084/jem.20240152 (PMC11349049; doi:10.1084/jem.20240152)
Supplement: Protocol — contains the clinical study protocol and statistical analysis plan. [file JEM_20240152_DataS1.pdf]

Protocol Title: An Exploratory Study of Nivolumab with or without Ipilimumab According to the Percentage of Tumoral CD8 Cells in Participants with Advanced Metastatic Cancer

Protocol Number: PICI0025

Amendment Number: 4

Compound Number: Nivolumab, Ipilimumab

Short Title: Treatment with Nivolumab and Ipilimumab or Nivolumab Alone According to the Percentage of Tumoral CD8 Cells in Advanced Metastatic Cancer

Sponsor Name and Legal Registered Address:

Parker Institute for Cancer Immunotherapy
1 Letterman Drive
Suite D3500
San Francisco, CA 94129

Tel: 415-610-5466

Regulatory Agency Identifying Number(s):

IND NUMBER: 138520

EudraCT NUMBER: Not applicable

Approval Date: Final Protocol: 06 April 2018
Amendment 1: 27 July 2018
Amendment 2: 12 June 2019
Amendment 3: 08 July 2020
Amendment 4: 12 March 2021

CONFIDENTIAL

This document and its contents are the property of and confidential to Parker Institute for Cancer Immunotherapy. Any unauthorized copying or use of this document is prohibited.

SPONSOR APPROVAL PAGE

DocuSigned by:
Justin Fairchild
Signer Name: Justin Fairchild
Signing Reason: I approve this document
Signing Time: 3/12/2021 | 4:04:59 PM PST
949277DE9B084F27973F0D2BBB82C4F0

Date: 3/12/2021 | 4:05:09 PM PST

Justin Fairchild
Vice President, Clinical Development

INVESTIGATOR PROTOCOL AGREEMENT PAGE

I agree:

- To assume responsibility for the proper conduct of the study at this site.
- To conduct the study in compliance with this protocol, any future amendments, and with any other study conduct procedures provided by Parker Institute for Cancer Immunotherapy.
- Not to implement any changes to the protocol without written agreement from Parker Institute for Cancer Immunotherapy and prior review and written approval from the Institutional Review Board (IRB) or Independent Ethics Committee (IEC) except where necessary to eliminate an immediate hazard to patients.
- That I am thoroughly familiar with the appropriate use of the study drug(s), as described in this protocol and any other information provided by Parker Institute for Cancer Immunotherapy including, but not limited to, the current Investigator's Brochure (IB).
- That I am aware of, and will comply with, Good Clinical Practices (GCP) and all applicable regulatory requirements.
- To ensure that all persons assisting me with the study are adequately informed about the study drugs, the Parker Institute for Cancer Immunotherapy study protocol, and of their study-related duties and functions as described in the protocol.

Signature: ______________________________________ Date: __________________

Name (print): ______________________________________

Principal Investigator

Site Number: ___________

Amendment 1: 27 July 2018

Text revisions resulting from this amendment are incorporated in the synopsis and body of Protocol Amendment 1. Major changes to the protocol are summarized below.

Key Revisions in Amendment 1

Section # and Name	Description of Change
Eligibility Criteria	
5.1 Inclusion Criteria	Revised inclusion criterion #4 to clarify that participants must have a tumor that "is responsive to immunomodulation (ie, with US Prescribing Information [USPI])" and that "participants who have failed or refused available approved treatment options are eligible to participate."
5.1 Inclusion Criteria	Revised inclusion criterion #5b to indicate that the CTCAE version 5.0 is to be used to grade prior immune-related adverse events affecting eligibility and that participants must be off steroid therapy and/or other immunosuppressive therapy for ≥ 14 days prior to starting study intervention.
5.1 Inclusion Criteria	Added inclusion criterion #7a to clarify that "biopsies should be obtained from sites that do not pose significant risk to the participant based on the tumor site and procedure used. Biopsy sites/procedures including, but not limited to, the brain, open lung/mediastinum, pancreas, or endoscopic procedures extending beyond the esophagus, stomach, or bowel would be considered to pose a significant risk to the participant. Procedures to areas that are deemed by the Investigator to be of non-significant risk based on individual clinical scenarios will be permitted."
5.1 Inclusion Criteria	Revised inclusion criterion #13 to indicate that "creatinine clearance should be ≥ 30 mL/min as estimated by the Cockcroft-Gault equation."
5.1 Exclusion Criteria	Revised exclusion criterion #3 to remove reference to "such that absorption of oral medication is impaired."
5.1 Exclusion Criteria	Revised exclusion criterion #5c to clarify the participant must be off steroids and/or other immunosuppressive therapy, as treatment for irAEs, for ≥ 30 days from Cycle 1, Day 1.
Safety Assessments	
6.6 Dose Modifications (Escalation/Titration/Other)	Added text referencing the Appendix 8: Management Algorithms for treatment of AEs related to nivolumab and/or ipilimumab
8.2 Safety Assessments	Removed reference to adverse events of special interest (AESIs).
8.3.1 Time Period and Frequency for Collecting AE, SAE, and Other Reportable Safety Event Information	Removed reference to adverse events of special interest (AESIs).<br>Clarified which AEs and SAEs should be reported prior to the initiation of study intervention and how other medical occurrences should be handled during prior to the initiation of study intervention.<br>Added (by relocating text from Section 8.3.2) and revised a paragraph describing how to handle SAEs occurring after the end of the AE reporting period that are considered to be reasonably related to the study intervention or study participation.<br>Introduced the PICI SAE Reporting Form (SAERF) as the appropriate safety-related paper form for reporting SAEs to the Sponsor and removed reference to the paper case report form.
8.3.2 Follow-up Event Reporting	Removed reference to adverse events of special interest (AESIs).

	Clarified that all AEs and SAEs will be followed through the follow up phase of the study and that events will be followed until event resolution or death.<br>Removed reference to "pregnancies" in the 2nd paragraph as pregnancy is considered an SAE so it was unnecessary to include it.<br>Moved paragraph describing how to handle certain SAEs to Section 8.3.1.
10.5.3 Recording AEs and SAEs	Removed reference to adverse events of special interest (AESIs) in the section for "Follow-up of AEs and SAEs."
10.5.4 Reporting of AEs, SAEs, and Other Reportable Safety Events to the Sponsor	Removed references to the paper CRF and SAE paper form and added in references to the PICI SAERF.
10.5.5 Additional Reporting Considerations	Removed reference to adverse events of special interest (AESIs) in the section for "Abnormal Liver Function Tests."
10.8 Management Algorithms	Added Appendix 8 (Management Algorithms)
Study Design	
4.1 Overall Design	Added text to clarify that when the baseline biopsy is not adequate to obtain information on the percentage of tumoral CD8 cells for treatment assignment, a second baseline biopsy may be obtained if medically feasible and the participant is willing to undergo another tumor biopsy.
8.7.2 Enrollment Biomarkers	Added text to clarify that when the baseline biopsy is not adequate to obtain information on the percentage of tumoral CD8 cells for treatment assignment, a second baseline biopsy may be obtained if medically feasible and the participant is willing to undergo another tumor biopsy.
9.1 Sample Size Determination	Added text to clarify transformation in tumor group from CD8 low to CD8 high.
Study Intervention	
1.2 Schema	Revised Study Schema to include doses for nivolumab + ipilimumab following progressive disease after nivolumab monotherapy.
6.1 Study Intervention(s) Administered	Revised text of the bullets to include the dose and schedule of nivolumab monotherapy and nivolumab in combination with ipilimumab.<br>Moved text related to recording AEs and SAEs for up to 100 days post-last dose to Section 8.3.1.
6.2 Preparation/Handling/ Storage/Accountability	Clarified that dose preparations are based on the participant's weight and should be adjusted if the participant has a 10% change in comparison to their initial weight.<br>Clarified that further guidance and information for final disposition of unused study intervention is provided in the Pharmacy Manuals.
6.4 Study Intervention Compliance	Revised text to clarify that study intervention will be administered by "authorized" site personnel.
6.6.1 Dose Modifications with Nivolumab and Ipilimumab	Added text to clarify that "after administration first dose of ipilimumab, the Investigator may determine (based on clinical symptoms) the number of future doses of ipilimumab the participant will receive, for a maximum of 4 doses. Participants who stop ipilimumab dosing early due to toxicities, may start nivolumab maintenance (ie, 4 doses [12 weeks] of nivolumab following the first dose)."

	Removed text indicating that "holding of 1 agent and not the other agent is not allowed," and that both drugs should be held "according to the recommended dose modifications."
7.1 Discontinuation of Study Intervention	Revised text to clarify that participants in the nivolumab monotherapy arm can decide to enroll into the nivolumab and ipilimumab combination treatment arm at the time of progression.
Study Procedures	
1.3 Schedule of Activities	Revised Table 1 and Table 2 as follows:<br>• added a column for first follow up visit that includes assessments to be performed;<br>• revised table header to include only study days;<br>• clarified disease assessment schedule according to cycle;<br>• revised multiple procedure labels (Vital signs, Clinical chemistry, Stool collection);<br>• revised and/or added footnotes (a, b, f, g, h, i, and k) to clarify and/or elaborate on the study procedures and timing.
5.4 Screen Failures	Revised section to remove sentence indicating that "rescreened participants should be assigned the same participant number as for the initial rescreening."
Clarification of Document	
General Revisions	Document updated to address minor typographical errors and editorial changes for clarity.
Synopsis	Added cross-reference to Section 9.3.2 (ie, Data Review Team).
4.4 Treatment Beyond Progression	Revised text in the 3rd paragraph to remove reference to initial progression "post-surgery."
7.1.1 Temporary Discontinuation	Removed section as it was not applicable to this protocol.
8.2.3 Vital Signs	Rearranged the text in this section to improve flow.
8.2.5 Clinical Safety Laboratory Assessments	Added text that clarify that clinical laboratory tests will be performed "at the local institution" as described in the Schedule of Activities.<br>Revised text to indicate that any clinically relevant changes occurring during the study should be recorded on the "appropriate eCRF."
8.2.5.1 Local Laboratory Assessments	Revised Table 7 as follows:<br>• revised profile label for Clinical chemistry;<br>• added "(either indirect or direct)" following total bilirubin<br>• added row for Urinalysis
8.3.6 Disease-related Events and/or Disease-related Outcomes Not Qualifying as AEs or SAEs	Reworded section to clarify how and when an investigator should report an event that is not considered underlying progression of disease.
8.3.7 Trial-limiting Toxicity	Removed item #8 stating that "alopecia will not be considered TOX."
8.3.8 Adverse Events of Special Interest	Removed section as it was not applicable to this protocol.
8.3.8.1 Sponsor Contact Information	Removed reference to alternate Medical Monitor contact information in the study manual.

8.3.8.2 Safety Reporting Contacts	Clarified the process for using and submitting the safety-related paper forms.
8.4 Treatment of Overdose	Moved the definition of overdose to the start of the section for clarity.<br>Removed reference to classification of deviations in the Monitoring Plan and clarified that the PI is responsible for reporting protocol deviations of overdose to the IRB/IEC.
10.1.6 Administrative Structure	Removed reference to PK analyses and the applicable PK section as these are not applicable to this protocol.
10.1.8 Source Documentation	Added a reference to "clinical outcome assessment (COA)/patient-reported outcomes (PRO)" as types of source documents.
10.5.2 Additional Events Reported in the Same Manner as an SAE	Added a cross-reference to Section 8.4 (ie, Treatment of Overdose).
10.6.4.2 Female Participants who Become Pregnant	Removed reference to "or be withdrawn from the study" from the bullet describing outcome if a female participant becomes pregnant while participating.
11 References	Added month to the version dates for the Opdivo and Yervoy USPIs.

Amendment 2: 12 June 2019

Text revisions resulting from this amendment are incorporated in the synopsis and body of Protocol Amendment 2. Major changes to the protocol are summarized below.

Key Revisions in Amendment 2

Section # and Name	Description of Change
Eligibility Criteria	
5.1 Inclusion criteria	Clarification of language in criteria #4 to align the text with the study population diversity.<br>Added a list of tumor types that are included in the study.
5.1 Inclusion criteria	Removed statement for criteria #12b requiring no liver lesions to align with USPI.
5.1 Inclusion criteria	Aligned criteria #10 regarding AST and ALT elevations to USPI for Opdivo (3x ULN) and removed language differentiating individuals with liver lesions (12a).
5.1 Inclusion criteria	Aligned criteria #12 regarding total bilirubin elevations to USPI for Opdivo (1.5x ULN) and removed language differentiating individuals with liver lesions. Removed criteria #12b requiring no liver lesions for participants with Gilbert syndrome.
5.1 Inclusion criteria	Added language to criteria #13 to allow for other methods for calculating creatinine clearance beyond Cockcroft-Gault to align with site calculation methods
5.2 Exclusion criteria	Added language to criteria #8 to clarify conditions for investigators decision making regarding uncontrolled illness.
5.2 Exclusion criteria	Removed criteria #9 regarding cardiac health to align with drug manufacturer guidance.

5.2 Exclusion criteria	Clarification of language in criteria #12 regarding autoimmune diseases to align with drug manufacturer language.
5.2 Exclusion criteria	Added criteria #13 describing systemic treatment with corticosteroids or other immunosuppressive medication limits as well as use of inhaled or topical steroids in absence of active autoimmune disease.
5.2 Exclusion criteria	Added language to criteria #14a allowing for recent or concurrent unrelated study interventions to be administered so long as they are not anticipated to interfere with the study with permission from the Medical Monitor. As an exception to this requirement, administration of ^{89}Zr-Df-IAB22M2C in the context of a PICI-sponsored clinical trial does not require written agreement from the Medical Monitor.
Safety Assessments	
8.3.4 Regulatory Reporting Requirements for SAEs	Added partner companies to list of parties who may receive safety reports depending on country-specific regulatory requirements.
9.2 Populations for Analysis	Clarification safety population description to state it will include participants who have received at least one dose of study drug and provide clarification on analysis population definitions.
9.3.1.1 Safety Monitoring	Updated the safety monitoring assumption so that the "standard therapy" is 40% instead of 30% based on previous data for the drug combination. Table 10 was updated to reflect this change in the "standard therapy" TOX rate assumption.
9.3.1.3 Joint Operating Characteristics for Safety and Futility Monitoring	Updated Table 11 to reflect the change in safety monitoring assumption in Section 9.3.1.1.
9.3.2 Data Review Team	Removal of section. Given the well-established safety profile of nivolumab as a single agent, and in combination with ipilimumab, a formalized Data Review Team was deemed unnecessary. The Sponsor will continue to monitor patient safety closely via real-time review of SAEs as they are received, and periodic review of aggregate safety data. Ad hoc meetings will be held in the event that a potential safety concern is identified.<br>A note of this has been added to the Synopsis under Safety and Futility Monitoring.
9.4.1 Efficacy Analyses	Updated language to clarify definition of the study population and clarify which analysis populations will be used for each efficacy analysis.
9.4.2 Safety Analyses	Clarification of the study population definition to align with Section 9.2 and removal of language that was duplicated from Section 9.3.1.1.
Study Design	
1.1 Synopsis	Clarification of study population diversity during enrollment to enrich for CD8 high tumors.
4.1 Study Design	Clarification of timing for a second biopsy when participant is on treatment
8.7.1 Genetics	Removed language indicating use of Future Biomedical Research consent. This has been incorporated into the current informed consent.
8.7.3 CD8 and Exploratory Biomarkers	Added language to allow for biopsies that occur within a 30 day window of consent to be used for the enrollment biopsy. Additionally, if the biopsy is outside of the 30 day window, Medical Monitor approval will be needed. This is to allow participants to not have to undergo multiple biopsies in close succession if not necessary.

10.1.4 Informed Consent	Added language regarding re-consent policy when rescreening is beyond 30 days from C1D1 and if the Institution policy differs from that of Parker that Parker accepts this policy.
Study Intervention	
1.3 Schedule of Activities	Revised language for footer l to match Section 8.3.1 with collection of AEs and SAEs to 100 days after the last dose of study intervention.
6.1 Study Intervention(s) Administered	Added windows for administration in text to match with the Schedule of Activities administration windows and clarify that participants who see clinical benefit will move to nivolumab maintenance doses.
6.5.1 Permitted Therapy	Added Section 6.5.2 to the sections containing excluded medications.
6.5.2 Prohibited Therapy	Clarification of language for concurrent therapies to allow for use of hormonal therapy with Medical Monitor approval at entry.
6.5.2 Prohibited Therapy	Revised window for receiving live vaccines to 100 days post last dose of study intervention.
6.5.2 Prohibited Therapy	Provided clarification regarding herbal and natural remedies allowing for use of marijuana and derivatives for treatments of cancer or cancer treatment-related symptoms.
7.1 Discontinuation of Study Intervention	Clarification of language related to dose-limiting toxicities and dose reductions.
Study Procedures	
1.3 Schedule of Activities	Revised Table 1 and Table 2 as follows:<br>• Removed assessment at C1D15 as it is not required for sufficient safety assessment<br>• Split footnote for follow-up period between first visit information and subsequent collections<br>• Added in collection of Informed Consent during the screening period<br>• Revised language for footer l to match Section 8.3.1 with collection of AEs and SAEs to 100 days after the last dose of study intervention<br>• Added footnote to medical history indicating collection of prior cancer therapies and procedures<br>• Added footnote for vital signs that pulse oximetry will only be collected at baseline<br>• Added footnote to tumor markers indicating the types of markers that may be collected<br>• Clarification of footnote for the AE, SAE, and all concomitant medication collection period after consent and where trial limiting toxicities can be found in the protocol body<br>• Added assessment of subsequent cancer therapy to post-treatment collections<br>• Added language to clarify that a 2nd ,on treatment biopsy may be obtained for either arm if the results from the previous are not adequate to obtain the percentage of tumoral CD8 cells<br>• Added header to clarify what cycles are On-Study Evaluation and which are the nivolumab maintenance period
8 Study Assessments and Procedures	Clarification of language related to minimum timing between doses for Q3W and Q4W schedule.
8.1 Efficacy Assessments	Clarification of language relating to guidance on whether non-contrast CT vs MRI should be used for various anatomical locations in the case of a dye allergy.

8.1.1 Laboratory Assessments of Tumor Markers	Added sub-section indicating the types of tumor markers that may be collected and sent to local laboratories for analysis as part of standard of care.
8.2.3 Vital Signs	Removed requirement for participants to rest 10 minutes prior to recording vital signs.
8.2.3 Vital Signs	Revised window for measurement of vitals to be 0-20 minutes prior to infusion.
8.2.3 Vital Signs	Removed pulse oximetry from every collection and only to be collected at baseline.
8.2.5.1 Local Laboratory Assessments	Added in testing for thyroid stimulating hormone (TSH), triiodothyronine (T3), free triiodothyronine (FT3), free thyroxine (FT4), and lipase in Table 7. Clarified total bilirubin included either indirect or direct
8.3.1 Time Period and Frequency for Collecting AE, SAE, and Other Reportable Safety Event Information	Removed language indication collection period could go until initiation of ne systemic anticancer therapy. Collection will go from signing of consent until 100 days after the last dose regardless of a new systemic anticancer therapy. This also aligns to the footnote in the Schedule of Activities.
8.3.1.1 Events Requiring Expedited Reporting to the Sponsor	Added language that events must reported on a SAE Report Form within 24 of becoming aware of the event.
8.3.6 Disease-related Events and/or Disease-Related Outcomes Not Qualifying as AEs or SAEs	Added language clarifying that SAEs must be reported with 24 hours of becoming aware of the event.
8.3.8.1 Emergency Medical Contacts	Updated Primary Medical Monitor to Marko Spasic per Administrative Change Notification
10.6.3 Pregnancy Testing	Revised frequency of additional pregnancy testing to state they will occur every cycle and within 24 hours prior to dosing, aligning to what is stated in the Schedule of Activities.
Clarification of Document	
General Revisions	Document updated to address minor typographical errors and editorial changes for clarity.<br>Change usage of SAERF abbreviation to SAE Report Form throughout to reflect internal naming for the form.
1.2 Schema	Updated schema for study, separating out the two arms of the study.
8.3.7 Trial-limiting Toxicity	Update language to be more inclusive as to events that lead to trial-limiting toxicity
10.1.2 Institutional Review Board or Independent Ethics Committee	Clarified who the IRB of Record (MD Anderson) is for the study and who are the Relying IRBs (UCLA, UCSF, Stanford, DFCI, and MSKCC) as well as that Parker maintains information relating to these institutions in separate documentation.
10.6.2.1 Male Participants	Updated language for contraception guidance for male participants to indicate methods other than abstinence that are acceptable to use.

Amendment 3: 08 July 2020

Text revisions resulting from this amendment are incorporated in the synopsis and body of Protocol Amendment 3. Major changes to the protocol are summarized below.

Key Revisions in Amendment 3

Section # and Name	Description of Change
Eligibility Criteria	
5.1 Inclusion criteria	Added criteria specific to participants with advanced prostate cancer (criteria #17-21).
5.1 Inclusion criteria	Updated criterion #8 to differentiate those participants enrolled before Amendment 3 and those enrolled under Amendment 3.
5.2 Exclusion criteria	Updated criteria #1 to clarify that the use of anesthesia for reasons other than general anesthesia during biopsies, patient comfort and or safety, are to be excluded.
Safety Assessments	
6.6 Dose Modifications (Escalation/Titration/Other)	Update Management algorithms for the study interventions to be in line with the current Investigator's Brochures.
8.1.1 Laboratory Assessments of Tumor Markers	Added language requiring collection of PSA for participants enrolled under Amendment 3.
9.1 Sample Size Determination	Added information on arms containing advanced prostate cancer participants and Table 12: Credible intervals for CD8 Conversion.
9.2 Populations for Analysis	Updated populations in Table 13:<br>• Renamed Primary Analysis Population to Modified Intent-to-Treat (mITT) Population.<br>• Safety Population will be analyzed according to study intervention actually received.<br>• mITT Population will be analyzed according to the study intervention received.<br>• On-treatment Biopsy Population will be participants with at least 1 on-treatment biopsy with sufficient CD8 results.
9.4 Statistical Analyses	Updated language to indicate the statistical analysis plan complements the protocol and supersedes it in case of differences.
9.4.1 Efficacy Analyses	Clarification of language and indication that participants will be analyzed according to the study intervention to which the participants were assigned.<br>Added exploratory endpoint for PSA changes from baseline.
9.4.2 Safety Analyses	Clarified that participants will be analyzed according to the study intervention actually received. AEs will be described by grade and attribution for each study intervention.
10.8 Management Algorithms	Updated the Management Algorithms to align with the current Investigator's Brochures.
Study Design	
1.1 Synopsis	• Updated language to describe advanced prostate cancer participant arms

	• Updated Objective and endpoints table to align with Section 3
3 Objectives and Endpoints	• Updated Objectives and Endpoints to include specific language for advanced prostate cancer population<br>• Clarification of endpoint to be used for all populations
4.1.1 Study Design	Added additional requirements for biopsies for participants in the advanced prostate cancer arms
6.3.1 Intervention Assignment	Added language specifying the advanced metastatic cancer participants are not formally randomized to a treatment regimen but that participants entering with advanced prostate cancer, in the new cohorts, will be randomly allocated to 1 of 2 treatment cohorts.
Study Intervention	
4.3.2 Rationale for Nivolumab and Ipilimumab Combination Therapy Dose and Schedule (Advanced Metastatic Cancer)	Updated header to differentiate from new cohorts
4.3.2.1 Rationale for Nivolumab and Ipilimumab Combination Therapy Dose and Schedule (Advanced Prostate Cancer Cohorts A and B)	Added section of combination therapy to be used for advanced prostate participants in the CD8 low and CD8 high arms.
6.1 Study Intervention(s) Administered	Table 1: Clarified dosage levels for advanced metastatic cancer participants and added information on interventions specific to participants with advanced prostate cancer<br>Added the intervention specifics for the participants with advanced prostate cancer in both the CD8 high and CD8 low arms. Specified the 2 cohorts within the CD8 low arm and the different interventions that will be given in each.
6.5.2 Prohibited Therapy	Added clarification participants receiving androgen deprivation therapy and bone strengthening therapies will be allowed to participate
6.6.1.1 Dose Modifications and Toxicity Management for Adverse Events Associated with Nivolumab with or without Ipilimumab	Updated Table 7 to be toxicity management requirements instead of guidelines for participant enrolled under Amendment 3 only and added footnote abbreviations
6.6.1.2 Dose Modifications and Toxicity Management for Infusion-related Reaction Associated with Nivolumab with or without Ipilimumab	Updated Table 8 to contain current dose modification and toxicity management guidelines for infusion reactions.
6.6.1.4 Additional Safety Precautions (Advanced Prostate Cancer Cohort Only)	Added section regarding a safety pause and discontinuation criteria for the new advanced prostate cohorts.
7.2 Participant Discontinuation/Withdrawal from Study	• Updates the timing for follow-up assessments to specify radiographic progression or start of subsequent therapy.

	• Added allowance for collection of ad hoc scans for participants who discontinue for reasons other than radiographic disease progression or prior to alternative therapies.
Study Procedures	
1.3 Schedule of Activities	• Added schedules for advanced prostate cancer population CD8 high and low arms<br>• Clarified that cfDNA is to be collected preinfusion<br>• Updated disease assessment language to include all possible reasons for stopping collection as well as allowance of collection of ad hoc scans<br>• Decoupled disease assessment from cycles due to the change in collection language
8.2.5.1 Local Laboratory Assessments	Updated Table 9 TSH laboratory collections to specify additional collections (FT3 and FT4) will only occur when TSH is abnormal and removed collection of T3 collection.
8.7 Biomarkers	Added language specifying baseline biopsies will be adequate for CD8 ICH testing when they contain > 20% tumor nuclei and may not apply the same to on-treatment biopsies.
Clarification of Document	
General Revisions	Document updated to address minor typographical errors and editorial changes for clarity.
1.2 Schema	Updated schema for study:<br>• Added two arms for advanced prostate cancer<br>• Updated footnotes for all schema
2.2.3 Immune Checkpoint Inhibitor Therapies in the Treatment of Metastatic Castration-resistant Prostate Cancer	Added section specific to advanced prostate cancer.
11 References	Added new references associated with added background text.

Amendment 4: 12 March 2021

Text revisions resulting from this amendment are incorporated in the synopsis and body of Protocol Amendment 4. Major changes to the protocol are summarized below.

Key Revisions in Amendment 4

Section # and Name	Description of Change
Safety Assessments	
9.4 Statistical Analyses	Added new exploratory endpoints for ApricityCare™ patient reported outcomes (PRO) Research.

Study Intervention	
6.6.1.1 Dose Modifications and Toxicity Management for Adverse Events Associated with Nivolumab with or without Ipilimumab	Updated Table 7 from CTCAE version 4 to CTCAE version 5
Study Design	
3 Objectives and Endpoints	Updated Objectives and Endpoints to include specific language for ApricityCare™ PRO Research
Study Procedures	
1.3 Schedule of Activities	Added scheduled assessments for advanced prostate cancer patients who opt-in to ApricityCare™ PRO
8.8 Patient-Reported Outcomes Research	New Section added
Clarification of Document	
General Revisions	Document updated to address minor typographical errors and editorial changes for clarity.
10.8 Management Algorithms	Appendix 8 has been updated from CTCAE version 4.0 to CTCAE version 5.0

TABLE OF CONTENTS

Table of Contents 15
List of Tables 20
List of Figures 21
1 Protocol Summary 22
1.1 Synopsis 22
1.2 Schema 24
1.3 Schedule of Activities 29
2 Introduction 42
2.1 Study Rationale 42
2.2 Background 42
2.2.1 Immune Checkpoint Inhibitors 42
2.2.2 Biomarkers to Predict Benefit from Treatment with Immune Checkpoint Inhibitors 43
2.2.3 Immune Checkpoint Inhibitor Therapies in the Treatment of Advanced Prostate Cancer 46
2.3 Benefit/Risk Assessment 48
3 Objectives and Endpoints 50
4 Study Design 51
4.1 Overall Design 51
4.1.1 Advanced Prostate Cancer 52
4.2 Scientific Rationale for Study Design 53
4.3 Justification for Dose 53
4.3.1 Rationale for Nivolumab Monotherapy Dose and Schedule 53
4.3.2 Rationale for Nivolumab and Ipilimumab Combination Therapy Dose and Schedule (Advanced Metastatic Cancer) 55
4.3.2.1 Rationale for Nivolumab and Ipilimumab Combination Therapy Dose and Schedule (Advanced Prostate Cancer Cohorts A and B) . 55
4.4 Treatment Beyond Disease Progression 56
4.5 End of Study Definition 57
5 Study Population 57
5.1 Inclusion Criteria 57
5.2 Exclusion Criteria 60
5.3 Lifestyle Considerations 61

5.4 Screen Failures 61
6 Study Intervention 62
6.1 Study Intervention(s) Administered 62
6.2 Preparation/Handling/Storage/Accountability 63
6.3 Measures to Minimize Bias: Randomization and Blinding 64
6.3.1 Intervention Assignment 64
6.3.2 Blinding 64
6.4 Study Intervention Compliance 65
6.5 Concomitant Therapy 65
6.5.1 Permitted Therapy 65
6.5.2 Prohibited Therapy 65
6.6 Dose Modifications (Escalation/Titration/ Other) 66
6.6.1 Dose Modifications with Nivolumab and Ipilimumab 66
6.6.1.1 Dose Modifications and Toxicity Management for Adverse Events Associated with Nivolumab with or without Ipilimumab 67
6.6.1.2 Dose Modifications and Toxicity Management for Infusion-related Reaction Associated with Nivolumab with or without Ipilimumab .73
6.6.1.3 Dose Delays and Interruptions for Nivolumab with or without Ipilimumab 75
6.6.1.4 Additional Safety Precautions (Advanced Prostate Cancer Cohort Only) 75
6.7 Intervention After the End of the Study 77
7 Discontinuations of Study Intervention and Participant Discontinuation/Withdrawal 77
7.1 Discontinuation of Study Intervention 77
7.2 Participant Discontinuation/Withdrawal from Study 78
7.3 Lost to Follow-up 78
8 Study Assessments and Procedures 79
8.1 Efficacy Assessments 79
8.1.1 Laboratory Assessments of Tumor Markers 80
8.2 Safety Assessments 81
8.2.1 Medical History and Demographic Data 81
8.2.2 Physical Examinations 81
8.2.3 Vital Signs 81
8.2.4 Electrocardiograms 82
8.2.5 Clinical Safety Laboratory Assessments 82

8.2.5.1 Local Laboratory Assessments 82
8.3 Adverse Events and Serious Adverse Events..83
8.3.1 Time Period and Frequency for Collecting AE, SAE, and Other Reportable Safety Event Information..83
8.3.1.1 Events Requiring Expedited Reporting to the Sponsor 84
8.3.2 Follow-up Event Reporting 84
8.3.3 Method of Eliciting Adverse Event Information 85
8.3.4 Regulatory Reporting Requirements for SAEs..85
8.3.5 Pregnancy 86
8.3.6 Disease-related Events and/or Disease-related Outcomes Not Qualifying as AEs or SAEs..86
8.3.7 Trial-limiting Toxicity...86
8.3.8 Sponsor Contact Information...87
8.3.8.1 Emergency Medical Contacts..87
8.3.8.2 Safety Reporting Contacts..87
8.4 Treatment of Overdose 88
8.5 Pharmacokinetics..88
8.6 Anti-drug Antibodies 88
8.7 Biomarkers 88
8.7.1 Genetics 90
8.7.2 Enrollment Biomarkers 90
8.7.3 CD8 and Exploratory Biomarkers..91
8.7.4 Sample Collection for Long-term Future Biomedical Research 91
8.7.4.1 Overview of Long-term Future Biomedical Research....................91
8.7.4.2 Sample Collection...92
8.7.4.3 Withdrawal from Long-term Sample Storage................................92
8.7.4.4 Protection of Data Privacy and Data Generation 93
8.8 Patient-Reported Outcomes Research 93
8.8.1 Medical Disclaimer 94
8.8.2 Health Data Protection 94
8.9 Medical Resource Utilization and Health Economics 95
9 Statistical Considerations...95
9.1 Sample Size Determination..95
9.2 Populations for Analysis 97
9.3 Interim Analyses..97

9.3.1 Safety and Futility Monitoring....97
9.3.1.1 Safety Monitoring....97
9.3.1.2 Futility Monitoring....98
9.3.1.3 Joint Operating Characteristics for Safety and Futility Monitoring....99
9.4 Statistical Analyses....100
9.4.1 Efficacy Analyses....100
9.4.2 Safety Analyses....101
9.4.3 Other Analyses....101
9.4.3.1 Biomarker Analysis....101
9.4.3.2 Patient-Reported Outcomes Analysis....101
10 Supporting Documentation and Operational Considerations....102
10.1 Appendix 1: Regulatory, Ethical and Study Oversight Considerations....102
10.1.1 Compliance with Laws and Regulations....102
10.1.2 Institutional Review Board or Independent Ethics Committee....102
10.1.3 Financial Disclosure....103
10.1.4 Informed Consent....103
10.1.5 Data Protection....104
10.1.6 Administrative Structure....104
10.1.7 Data Quality Assurance....105
10.1.8 Source Documentation....105
10.1.9 Study and Site Closure....106
10.1.10 Site Inspections....107
10.1.11 Retention of Records....107
10.1.12 Publication Policy and Protection of Trade Secrets....107
10.2 Appendix 2: Eastern Cooperative Oncology Group (ECOG) Performance Status....108
10.3 Appendix 3: RECIST Criteria (Version 1.1)....109
10.3.1 Measurability of Tumor at Baseline....109
10.3.2 Tumor Response Evaluation....109
10.3.2.1 Baseline Documentation of Target and Nontarget Lesions....109
10.3.2.2 Evaluation of Target Lesions....110
10.3.2.3 Evaluation of Nontarget Lesions....110
10.3.2.4 New Lesions....111
10.3.3 Evaluation of Overall Response....111
10.4 Appendix 4: Clinical Laboratory Tests....113

10.5 Appendix 5: Adverse Events: Definitions and Procedures for Recording, Evaluating, Follow-up, and Reporting 114
10.5.1 Definitions 114
10.5.1.1 Definition of AE 114
10.5.1.2 Definition of SAE 115
10.5.1.3 Definition of Unexpected AE 116
10.5.1.4 Definition of Treatment-emergent AE 117
10.5.2 Additional Events Reported in the Same Manner as an SAE 117
10.5.3 Recording AEs and SAEs 117
10.5.4 Reporting of AEs, SAEs, and Other Reportable Safety Events to the Sponsor 120
10.5.5 Additional Reporting Considerations 121
10.6 Appendix 6: Contraceptive Guidance and Collection of Pregnancy Information 125
10.6.1 Definitions 125
10.6.1.1 Woman of Childbearing Potential (WOCBP) 125
10.6.2 Contraception Guidance 125
10.6.2.1 Male Participants 125
10.6.2.2 Female Participants 126
10.6.3 Pregnancy Testing 127
10.6.4 Collection of Pregnancy Information 127
10.6.4.1 Male Participants with Partners who Become Pregnant 127
10.6.4.2 Female Participants who Become Pregnant 127
10.7 Appendix 7: Genetics 129
10.8 Appendix 8: Management Algorithms 130
10.9 Appendix 9: List of Abbreviations 138
10.10 Appendix 10: Protocol Amendment History 141

LIST OF TABLES

Table 1: Schedule of Assessments Advanced Metastatic Cancer (Nivolumab Only Arm: Nivolumab 360 mg Q3W and Maintenance 480 mg Q4W)....30

Table 2: Schedule of Assessments Advanced Metastatic Cancer (Nivolumab and Ipilimumab Arm: Nivolumab Q3W and Ipilimumab Q3W [Doses 1 & 2], then Q6W [Doses 3 & 4], Followed by Single-agent Nivolumab Q4W)....33

Table 3: Schedule of Assessments Advanced Prostate Cancer (Nivolumab Only Arm: Nivolumab 360 mg Q3W and Maintenance 480 mg Q4W)....36

Table 4: Schedule of Assessments Advanced Prostate Cancer (Nivolumab and Ipilimumab Arm: Nivolumab Q3W and Ipilimumab Q6W [Cycles 1 and 2], Followed by Single-agent Nivolumab Q4W)....39

Table 5: Objectives and Corresponding Endpoints....50

Table 6: Study Intervention....62

Table 7: Dose Modification and Toxicity Management Guidelines for Adverse Events Associated with Nivolumab with or without Ipilimumab....68

Table 8: Dose Modification and Toxicity Management Guidelines for Infusion-related Reaction Associated with Nivolumab with or without Ipilimumab....74

Table 9: Toxicity Criteria Requiring Permanent Treatment Discontinuation of Both Nivolumab and Ipilimumab (Amendment 3 Advanced Prostate Cohort Only)....76

Table 10: Laboratory Tests Sent to the Study Site's Local Laboratory for Analysis....83

Table 11: Possible Subgroup Sizes....96

Table 12: Credible Intervals for CD8 Conversion....97

Table 13: Populations for Analysis....97

Table 14: Rules for Safety and Futility Monitoring per Arm....98

Table 15: Operating Characteristics Under Varying Toxicity and Response Rates....99

LIST OF FIGURES

Figure 1: Study Schema: Advanced Metastatic Cancer Monotherapy Arm (CD8+ ≥ 15%) 25

Figure 2: Study Schema: Advanced Metastatic Cancer Combination Arm (CD8+ < 15%). 26

Figure 3: Study Schema: Advanced Prostate Cancer Monotherapy Arm (CD8+ ≥ 15% [CD8 High Arm]) 27

Figure 4: Study Schema: Advanced Prostate Cancer Combination Arm (CD8+ < 15% [CD8 Low Arm]) 28

Figure 5: Tumor-infiltrating CD8 T-cell Expression in Responders and Non-responders to PD-1 Blockade 44

Figure 6: Tumor-infiltrating CD8 T-cell Expression by Best Overall Response 45

Figure 7: Percentage of Tumoral CD8 T Cells by Immunohistochemistry in Different Tumor Types 46

1 PROTOCOL SUMMARY

1.1 SYNOPSIS

Protocol Title:

An Exploratory Study of Nivolumab with or without Ipilimumab According to the Percentage of Tumoral CD8 Cells in Participants with Advanced Metastatic Cancer

Short Title:

Treatment with Nivolumab and Ipilimumab or Nivolumab Alone According to the Percentage of Tumoral CD8 Cells in Advanced Metastatic Cancer

Rationale:

The aim of this study is to provide a prospective classification of CD8 high (immunologically "hot") versus CD8 low (immunologically "cold") tumors at the time of treatment, based on the percentage of CD8 cells in a tumor biopsy, and to address the predictive value of the CD8 biomarker for selecting patients for treatment with nivolumab with or without ipilimumab.

Key Objectives and Endpoints:

Objectives	Endpoints
Primary	
• To determine the clinical benefit rate (CBR) of nivolumab with or without ipilimumab in participants with advanced metastatic cancer, including advanced prostate cancer.<br>• To assess the proportion of participants in the nivolumab plus ipilimumab arm whose tumors will change from CD8 low to CD8 high as measured by a change in the percentage of tumoral CD8 cells.	• CBR is the proportion of participants who show clinical benefit, defined as CR, PR, or SD for ≥ 6 months as best response by RECIST v1.1.<br>• Change in the percentage of CD8 cells in on-treatment biopsies from baseline.
Secondary	
• To determine the safety and tolerability of nivolumab with or without ipilimumab in participants with advanced metastatic cancer, including advanced prostate cancer.<br>• To determine the ORR of nivolumab with or without ipilimumab in participants with advanced metastatic cancer, including advanced prostate cancer.<br>• To assess the association of percentage of CD8 infiltration in tumor samples with clinical outcomes (ORR, PFS, and OS).	• Incidence and severity of AEs based on CTCAE v5.0.<br>• ORR: Defined as CR or PR as best response by RECIST v1.1 assessment.<br>• PFS: Defined as the time from initiation of study therapy to date of first documented progression of disease or date of death due to any cause.<br>• OS: Defined as the time from initiation of study therapy until death due to any cause.

AE = adverse event; CBR = clinical benefit rate; CR = complete response; CTCAE = Common Terminology Criteria for Adverse Events; ORR = objective response rate; OS = overall survival; PFS = progression-free survival; PR = partial response; RECIST = Response Evaluation Criteria in Solid Tumors; SD = stable disease.

Overall Design:

This is an open-label, exploratory study to evaluate nivolumab with or without ipilimumab based on percentage of tumoral CD8 cells at the time of treatment in participants with varying advanced solid tumors. Participants who have a tumor with ≥ 15% CD8 cells (classified as CD8 high) will receive nivolumab monotherapy, and participants who have a tumor with < 15% CD8 cells (classified as CD8 low) will receive ipilimumab in combination with nivolumab.

Number of Participants:

A total of up to approximately 200 participants will be treated. Beginning with Amendment 2, enrollment will be limited to tumor types known to be responsive to immunotherapy, have high prevalence (> 20%) CD8 ≥ 15% tumors, and/or have been observed in the study to have tumors transition from CD8 low to CD8 high following initiation of immunotherapy. Beginning with Amendment 3, a total of approximately 20 participants with advanced prostate cancer and tumoral CD8 < 15% will be randomly allocated to 1 of 2 cohorts using combinations of ipilimumab and nivolumab. Advanced prostate cancer participants with tumoral CD8 ≥ 15% will be enrolled in the nivolumab monotherapy arm. Ongoing monitoring for safety and futility will be implemented based on the method of Thall and colleagues (Thall et al, 1995) separately in the CD8 high and CD8 low tumor groups. Refer to Section 9.1.

Intervention Groups and Duration:

Advanced Metastatic Cancer: Single-agent nivolumab will be administered at 360 mg intravenously (IV) every 3 weeks (Q3W). Participants who continue to show clinical benefit after the first disease assessment will receive nivolumab 480 mg IV every 4 weeks (Q4W) until progressive disease (PD) or intolerable toxicity. At PD, participants will be allowed to crossover to the CD8 low arm of ipilimumab (1 mg/kg) and nivolumab (360 mg).

For nivolumab and ipilimumab combination therapy, nivolumab will be administered at 360 mg IV Q3W, and ipilimumab will be administered at 1 mg/kg IV Q3W for the first 2 doses and then every 6 weeks for the 3rd and 4th doses, followed by nivolumab 480 mg IV Q4W until PD or intolerable toxicity. After receipt of the first dose of ipilimumab, the Investigator may determine (based on clinical symptoms) the number of future doses of ipilimumab the participant will receive, for a maximum of 4 doses. Participants who stop ipilimumab dosing early due to toxicities, may start nivolumab maintenance (ie, 4 doses [12 weeks] of nivolumab following the first dose).

Advanced Prostate Cancer (Amendment 3): Single-agent nivolumab administered at 360 mg intravenously (IV) every 3 weeks (Q3W). Participants who continue to show clinical benefit after the first disease assessment will receive nivolumab 480 mg IV every 4 weeks (Q4W) until progressive disease (PD) or intolerable toxicity. At PD, participants will be allowed to crossover

to the advanced prostate cancer CD8 low arm of ipilimumab (3 mg/kg) and nivolumab (1 mg/kg).

For nivolumab and ipilimumab combination therapy, CD8 low arm will be randomly allocated into 1 of 2 cohorts, using different doses of ipilimumab administered in 6-week cycles. Prostate Cohort A will receive nivolumab 1 mg/kg Q3W and ipilimumab 3 mg/kg every 6 weeks (Q6W) for 2 cycles, then nivolumab maintenance 480 mg Q4W until PD or intolerable toxicity. Prostate Cohort B will receive nivolumab 1 mg/kg Q3W and ipilimumab 5 mg/kg Q6W for 2 cycles, then nivolumab maintenance 480 mg Q4W until PD or intolerable toxicity.

Safety and Futility Monitoring:

Monitoring will be ongoing during enrollment in each group to assess the stopping rules as defined in the protocol (see Section 9.3.1). There is no independent Data Safety Monitoring Committee.

1.2 SCHEMA

The study schema is depicted in Figure 1 and Figure 2 for advanced metastatic cancer and in Figure 3 and Figure 4 for advanced prostate cancer.

Figure 1: Study Schema: Advanced Metastatic Cancer Monotherapy Arm (CD8+ ≥ 15%)

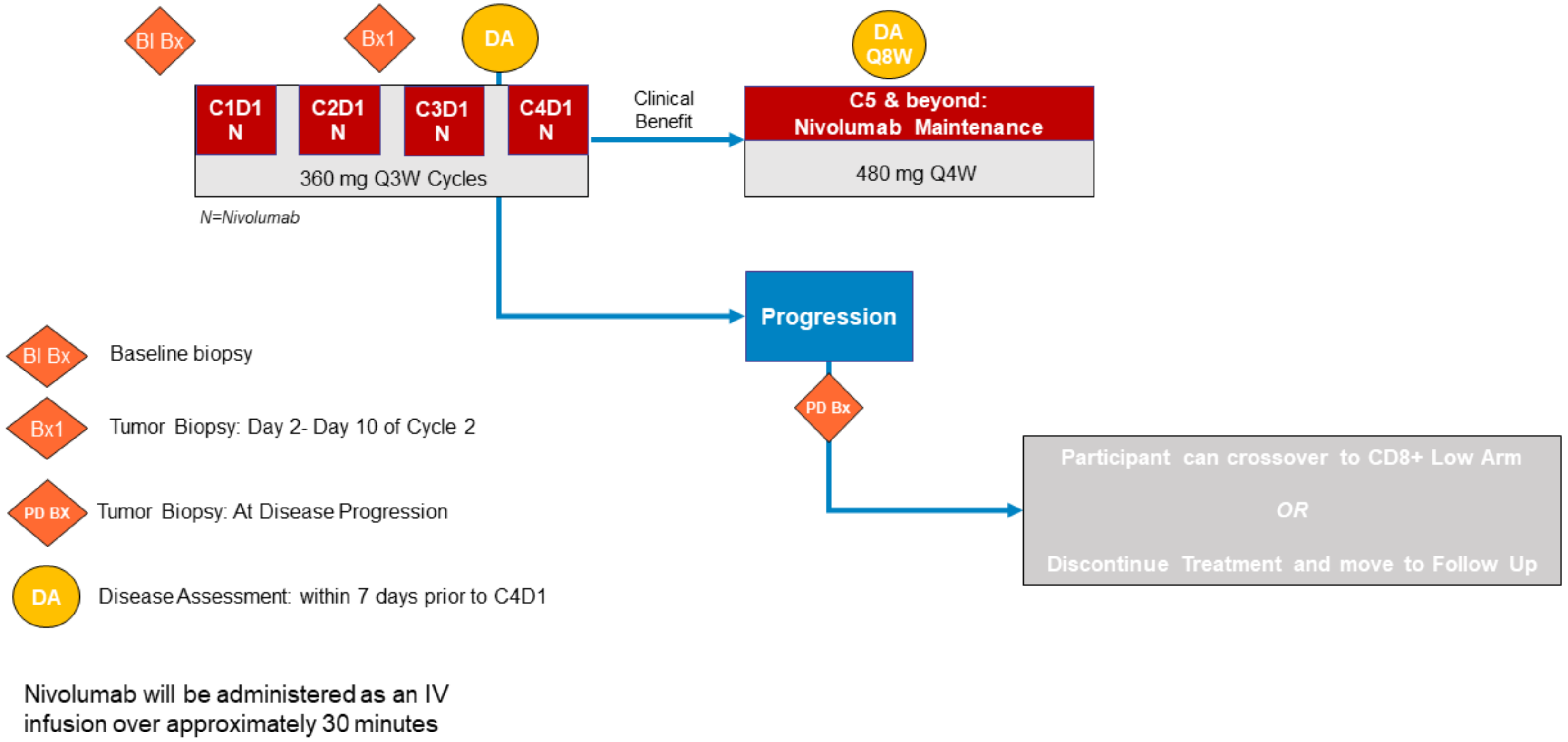

Bl = baseline; Bx = tumor biopsy; DA = disease assessment; IV = intravenous(ly); N = nivolumab; PD = progressive disease; Q3W = every 3 weeks; Q4W = every 4 weeks; Q6W = every 6 weeks; Q8W = every 8 weeks.

[a] Single-agent nivolumab will be administered at 360 mg IV Q3W. Participants who continue to show clinical benefit after the first disease assessment will receive nivolumab 480 mg IV Q4W until PD or intolerable toxicity. At PD (any time following initiation of study therapy), participants will be allowed to cross-over to the Advanced Metastatic Cancer Combination Arm. All decisions to cross-over must be discussed with the Sponsor.

[b] Blood collection prior to administration of each dose and at time of progression.

[c] For participants who cross-over to the CD8 low arm, nivolumab will be administered at 360 mg IV Q3W, and ipilimumab will be administered at 1 mg/kg IV Q3W for the first 2 doses and then Q6W for the 3rd and 4th doses, followed by single-agent nivolumab 480 mg IV Q4W until PD or intolerable toxicity.

Figure 2: Study Schema: Advanced Metastatic Cancer Combination Arm (CD8+ < 15%)

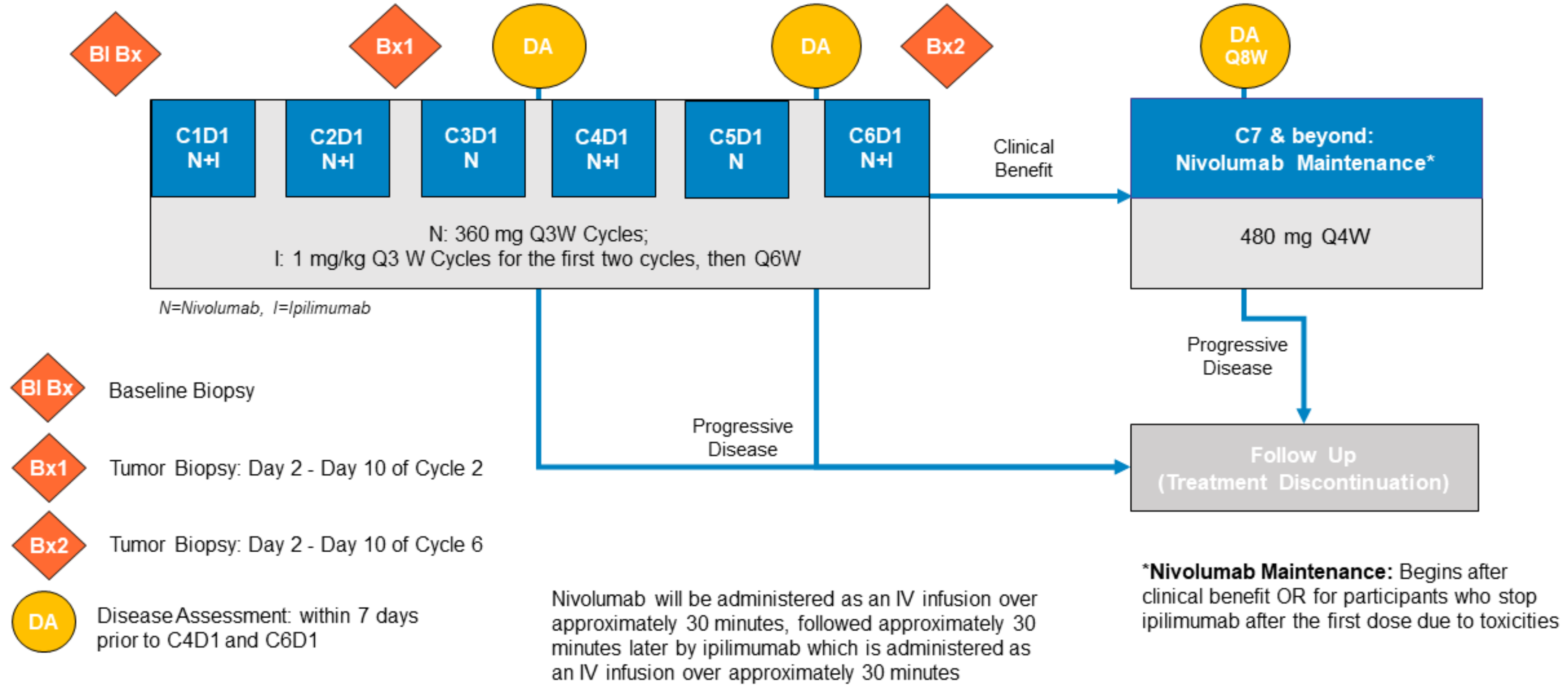

Bl = baseline; Bx = tumor biopsy; DA = disease assessment; I = ipilimumab; IV = intravenous(ly); N = nivolumab; PD = progressive disease; Q3W = every 3 weeks; Q4W = every 4 weeks; Q6W = every 6 weeks; Q8W = every 8 weeks.

[a] For nivolumab and ipilimumab combination therapy, nivolumab will be administered at 360 mg IV Q3W, and ipilimumab will be administered at 1 mg/kg IV Q3W for the first 2 doses and then Q6W for the 3rd and 4th doses, followed by single-agent nivolumab 480 mg IV Q4W until PD or intolerable toxicity.

[b] Blood collection prior to administration of each dose and at time of progression.

Figure 3: Study Schema: Advanced Prostate Cancer Monotherapy Arm (CD8+ ≥ 15% [CD8 High Arm])

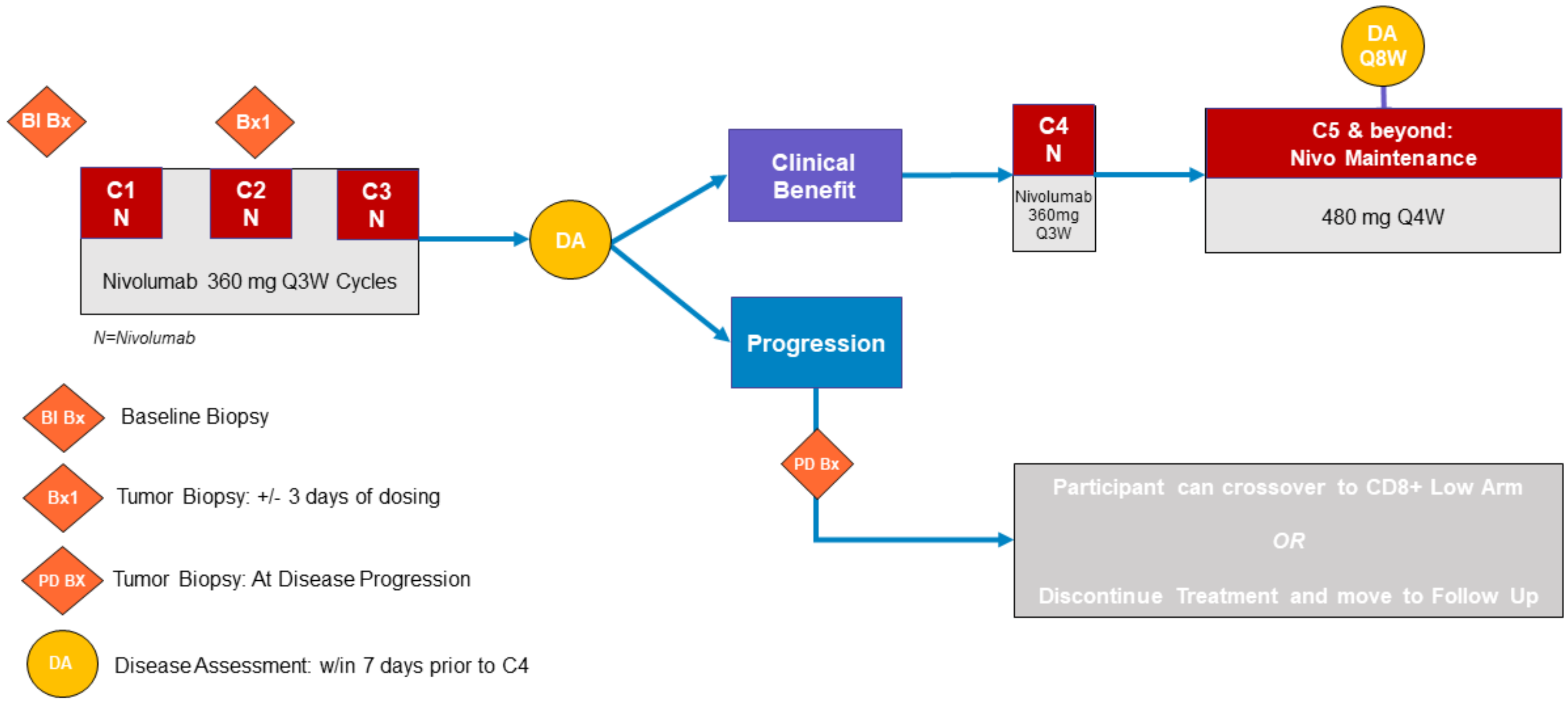

Bl = baseline; Bx =tumor biopsy; DA = disease assessment; IV = intravenous(ly); N = nivolumab; PD = progressive disease; Q3W = every 3 weeks; Q4W = every 4 weeks; Q6W = every 6 weeks; Q8W = every 8 weeks.

[a] Single-agent nivolumab will be administered at 360 mg IV Q3W. Participants who continue to show clinical benefit after the first disease assessment will receive nivolumab 480 mg IV Q4W until PD or intolerable toxicity. At PD (anytime following initiation of study therapy), participants will be allowed to cross-over to the Advanced Prostate Cancer Combination Therapy Arm. All decisions to cross-over must be discussed with the Sponsor.

[b] Blood collection prior to administration of each dose and at time of progression.

[c] For participants who cross-over to the CD8 low arm, nivolumab will be administered at 1 mg/kg IV Q3W, and ipilimumab will be administered at 3 mg/kg IV Q6W for 2 cycles (Prostate Cohort A), followed by nivolumab maintenance 480 mg IV Q4W until PD or intolerable toxicity.

Figure 4: Study Schema: Advanced Prostate Cancer Combination Arm (CD8+ < 15% [CD8 Low Arm])

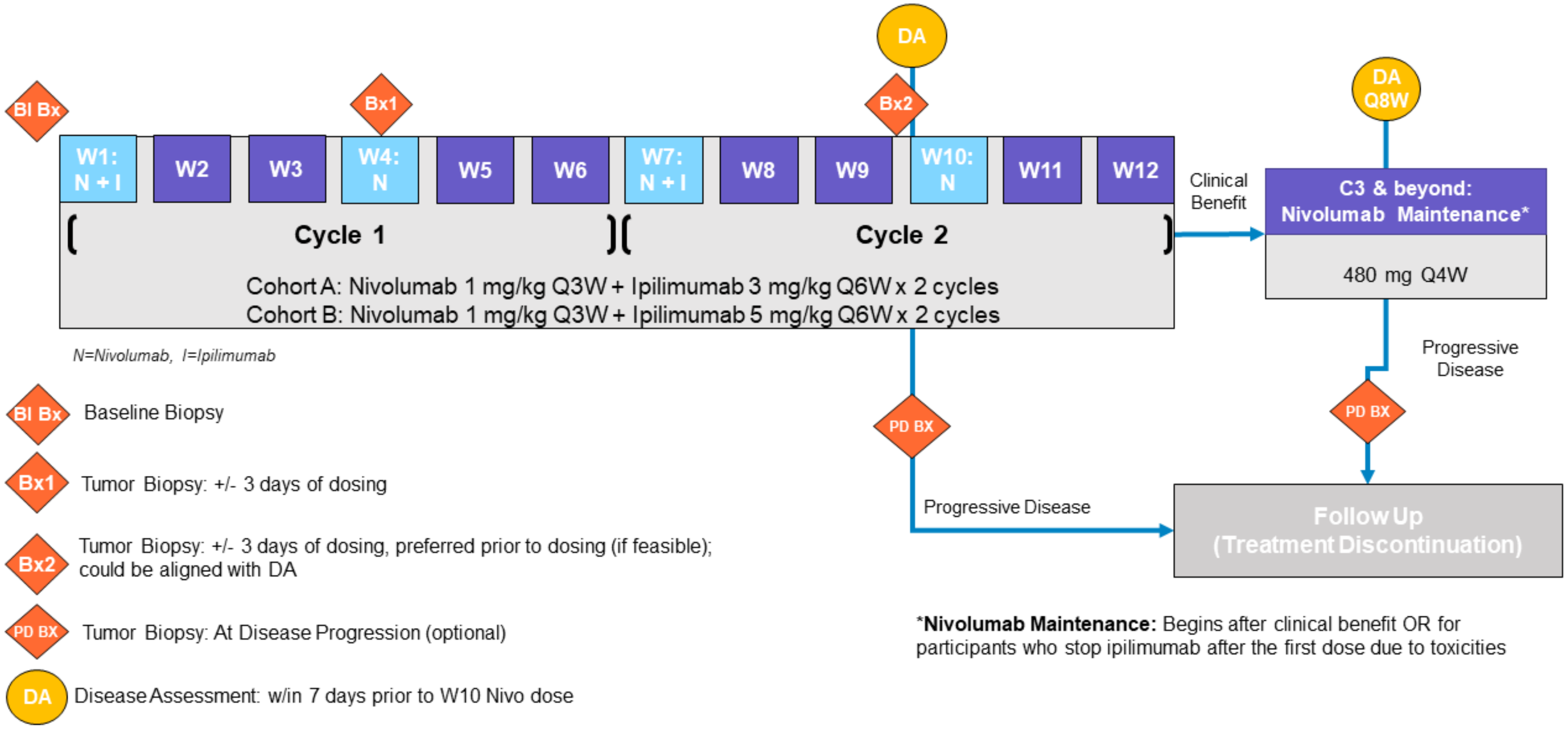

Bl = baseline; Bx = tumor biopsy; DA = disease assessment; I = ipilimumab; IV = intravenous(ly); N = nivolumab; PD = progressive disease; Q3W = every 3 weeks; Q4W = every 4 weeks; Q6W = every 6 weeks; Q8W = every 8 weeks.

[a] Nivolumab and ipilimumab combination therapy will be administered in 2 cohorts. For Prostate Cohort A, nivolumab will be administered at 1 mg/kg IV Q3W, and ipilimumab will be administered at 3 mg/kg IV Q6W for 2 cycles, followed by nivolumab maintenance 480 mg IV Q4W until PD or intolerable toxicity. For Prostate Cohort B, nivolumab will be administered at 1 mg/kg IV Q3W, and ipilimumab will be administered at 5 mg/kg IV Q6W for 2 cycles, followed by nivolumab maintenance 480 mg IV Q4W until PD or intolerable toxicity.

1.3 SCHEDULE OF ACTIVITIES

The Schedule of Activities (SOAs) for the nivolumab only arm and nivolumab plus ipilimumab combination arm are shown in Table 1 and Table 2 for advanced metastatic cancer and in Table 3 and Table 4 for advanced prostate cancer.

Table 1: Schedule of Assessments Advanced Metastatic Cancer (Nivolumab Only Arm: Nivolumab 360 mg Q3W and Maintenance 480 mg Q4W)

Tests & Procedures	Screening[a]	On-Treatment Evaluations									End of Treatment	Follow up[b]	
		C1		C2	C3	C4	C5	C6	C7	C8			
Day	0	D1	D8	D1	D1	D1	D1	D1	D1	D1	15-30 days after last dose	First visit (90 days after last dose)[b]	Q3M for 2 years[c]
Window (days)	-28 to 0	--	± 3	± 3	± 3	± 3	± 3	± 3	± 3	± 3	± 7	± 7	± 7
Informed consent[a]	X												
Medical, cancer history[d]	X												
Physical examination	X			X	X	X	X	X	X	X	X	X	
Pregnancy test (serum or urine)[e]	X[e]			X	X	X	X	X	X	X	X		
ECOG performance status	X			X	X	X	X	X	X	X	X	X	
Vital signs (see Section 8.2.3)[f]	X	X		X	X	X	X	X	X	X	X	X	
Body weight	X	X		X	X	X	X	X	X	X			
Hematology (see Table 10)	X		X	X	X	X	X	X	X	X	X	X	
Clinical chemistry (see Table 10)	X		X	X	X	X	X	X	X	X	X	X	
Urinalysis	X		X	X	X	X	X	X	X	X	X	X	
12-lead ECG	X												
Tumor markers[g]	X			X	X	X	X	X	X	X	X		
cfDNA (blood)[h]	X			X							X		
Circulating soluble analytes[i]	X	X	X	X	X	X					X		
Blood immune biomarkers[i]	X	X	X	X	X	X					X		
Stool collection[j]	X			X									
Tumor biopsy[k]	X[l]	As early as possible after the 2nd dose of nivolumab monotherapy[k] and at PD											
Concomitant medications[m]	X	X	X	X	X	X	X	X	X	X	X	X	
Adverse events[m]	X	X	X	X	X	X	X	X	X	X	X	X	
Nivolumab administration[n]		X		X	X	X	X[o]	X	X	X			
Follow-up for overall survival[c]												X	X

Tests & Procedures	Screening[a]	On-Treatment Evaluations									End of Treatment	Follow up[b]	
		C1		C2	C3	C4	C5	C6	C7	C8			
Day	0	D1	D8	D1	D1	D1	D1	D1	D1	D1	15-30 days after last dose	First visit (90 days after last dose)[b]	Q3M for 2 years[c]
Window (days)	-28 to 0	--	± 3	± 3	± 3	± 3	± 3	± 3	± 3	± 3	± 7	± 7	± 7
Assess subsequent cancer therapy											X	X	X
Disease assessment[p]	X	Cycle 4 Day 1, then beginning at Cycle 6 Day 1, repeat every 8 weeks (± 7 days) until radiographic progression or the start of subsequent therapy, whichever comes first											

cfDNA = cell-free deoxyribonucleic acid; Cx = Cycle x; ECG = electrocardiogram; ECOG = Eastern Cooperative Oncology Group; EOT = end of treatment; PD = progressive disease; Q3M = every 3 months; Q3W = every 3 weeks; Q4W = every 4 weeks; Q6W = every 6 weeks; Q12W = every 12 weeks.

[a] Screening evaluations are to be conducted within 28 days of the first dose of protocol therapy. Some screening procedures may occur on Cycle 1 Day 1 as appropriate. Informed consent must be obtained prior to any study-specific procedures and may be obtained prior to the 28-day screening window.

[b] Ninety (90) days after the last dose there is a visit with assessments ***if no alternative therapy has started***. If the participant has started alternative therapy within that 90 days after the last dose , the first follow up visit will be contact by telephone only.

[c] For a maximum of 2 years from the initiation of study intervention, participants will be contacted by telephone every 3 months to determine their survival status.

[d] Cancer history will include stage, date of diagnoses, and prior cancer therapies and procedures.

[e] Pregnancy tests (serum or urine) will be performed at Screening (within 24 hours of first dose), on Day 1 of every cycle thereafter (within 24 hours of dose), and EOT.

[f] Pulse oximetry will be collected with vital signs only at baseline.

[g] Tumor markers, (eg, CA19-9, PSA, etc) will be collected if performed as part of standard of care.

[h] cfDNA (blood) will be assessed at Screening, preinfusion Cycle 2 Day 1, and EOT.

[i] Circulating soluble analytes and blood immune biomarkers will be assessed at Screening, preinfusion Cycle 1 Days 1 and 8, Cycles 2-4 Day 1, and EOT.

[j] Stool collection will be assessed at Screening and at Cycle 2 Day 1, if possible. If this timing is not possible, any stool sample collected while on treatment would be acceptable.

[k] Participants will undergo 3 tumor biopsies: mandatory baseline biopsy (for treatment assignment based on percent of tumoral CD8 cells), as early as possible after the 2nd dose of nivolumab (Day 2 – Day 10 of Cycle 2; however, any on treatment biopsy after Day 1 of Cycle 2 will be accepted, if medically feasible), and at PD (if medically feasible) prior to the optional addition of ipilimumab. Regardless of dosing, participants should continue to follow assessments as timed.

[l] If the results of the baseline screening biopsy are not adequate to obtain information on the percentage of tumoral CD8 cells for treatment assignment, a 2nd baseline biopsy may be obtained, if it is medically feasible and the participant is willing to undergo another tumor biopsy. Additionally, if the results of the on treatment biopsy are not adequate to obtain information on the percentage of tumoral CD8 cells, a 2nd on treatment biopsy may be obtained, if it is medically feasible and the participant is willing to undergo another tumor biopsy.

[m] All AEs, SAEs, and all concomitant medications will be collected from-the time the participant signs informed consent until 100 days after the last dose of study intervention. Prior to initiation of study intervention, only AEs and SAEs that are related to a protocol mandated intervention, including those that occur

prior to assignment of study procedures (eg, screening invasive procedures, such as biopsies) should be reported. Trial-limiting toxicities can be found in Section 8.3.7.

[n] Nivolumab monotherapy will be administered at 360 mg IV Q3W for 4 cycles. Participants who continue to show clinical benefit after the first disease assessment will receive nivolumab at 480 mg IV Q4W until PD or intolerable toxicity (starting at Cycle 5).

[o] Beginning at Cycle 5, nivolumab maintenance (nivolumab at 480 mg IV Q4W) will be administered until PD or intolerable toxicity.

[p] Scans will be performed within 7 days prior to Day 1 of Cycle 4, Cycle 6, and every ***even*** cycle thereafter until death, disease progression, initiation of another systemic anticancer therapy, loss to follow-up, withdrawal of consent, or study termination, whichever occurs first (See Section 8.1). In addition, ad hoc scans, as part of standard of care, will be collected for participants who have discontinued treatment for reasons other than radiographic progression and prior to any alternate therapies.

Table 2: Schedule of Assessments Advanced Metastatic Cancer (Nivolumab and Ipilimumab Arm: Nivolumab Q3W and Ipilimumab Q3W [Doses 1 & 2], then Q6W [Doses 3 & 4], Followed by Single-agent Nivolumab Q4W)

Tests & Procedures	Screening[a]	On-Treatment Evaluations											End of Treatment	Follow-up	
		C1		C2	C3	C4	C5	C6	C7	C8	C9	C10			
Day	0	D1	D8	D1	D1	D1	D1	D1	D1	D1	D1	D1	15-30 days after last dose	First visit (90 days after last dose)[b]	Q3M for 2 years[c]
Window (days)	-28 to 0	--	± 3	± 3	± 3	± 3	± 3	± 3	± 3	± 3	± 3	± 3	± 7	± 7	± 7
Informed consent[a]	X														
Medical, cancer history[d]	X														
Physical examination	X			X	X	X	X	X	X	X	X	X	X	X	
Pregnancy test (serum or urine)[e]	X[e]			X	X	X	X	X	X	X	X	X	X		
ECOG performance status	X			X	X	X	X	X	X	X	X	X	X	X	
Vital signs (see Section 8.2.3)[f]	X	X		X	X	X	X	X	X	X	X	X	X	X	
Body weight	X	X		X	X	X	X	X	X	X	X	X			
Hematology (see Table 10)	X		X	X	X	X	X	X	X	X	X	X	X	X	
Clinical chemistry (see Table 10)	X		X	X	X	X	X	X	X	X	X	X	X	X	
Urinalysis	X		X	X	X	X	X	X	X	X	X	X	X	X	
12-lead ECG	X														
Tumor markers[g]	X			X	X	X	X	X	X	X	X	X	X		
cfDNA (blood)[h]	X			X									X		
Circulating soluble analytes[i]	X	X	X	X	X	X							X		
Blood immune biomarkers[i]	X	X	X	X	X	X							X		
Stool collection[j]	X			X											
Tumor biopsy[k]	X[l]	As early as possible after the 2nd dose[k] of ipilimumab and after the 4th dose of ipilimumab													
Concomitant medications[m]	X	X	X	X	X	X	X	X	X	X	X	X	X	X	
Adverse events[m]	X	X	X	X	X	X	X	X	X	X	X	X	X	X	
Nivolumab administration[n]		X		X	X	X	X	X	X[o]	X	X	X			
Ipilimumab administration[n]		X		X		X		X							

Tests & Procedures	Screening[a]	On-Treatment Evaluations											End of Treatment	Follow-up	
		C1		C2	C3	C4	C5	C6	C7	C8	C9	C10			
Day	0	D1	D8	D1	D1	D1	D1	D1	D1	D1	D1	D1	15-30 days after last dose	First visit (90 days after last dose)[b]	Q3M for 2 years[c]
Window (days)	-28 to 0	--	± 3	± 3	± 3	± 3	± 3	± 3	± 3	± 3	± 3	± 3	± 7	± 7	± 7
Follow-up for overall survival[c]														X	X
Assess subsequent cancer therapy													X	X	X
Disease assessment[p]	X	Cycle 4 Day 1, then beginning at Cycle 6 Day 1, repeat every 8 weeks (± 7 days) until radiographic progression or the start of subsequent therapy, whichever comes first													

cfDNA = cell-free deoxyribonucleic acid; Cx = Cycle x; ECG = electrocardiogram; ECOG = Eastern Cooperative Oncology Group; Q3M = every 3 months; Q3W = every 3 weeks; Q4W = every 4 weeks; Q6W = every 6 weeks; Q12W = every 12 weeks.

[a] Screening evaluations are to be conducted within 28 days of the first dose of protocol therapy. Some screening procedures may occur on Cycle 1 Day 1 as appropriate. Informed consent must be obtained prior to any study-specific procedures and may be obtained prior to the 28-day screening window.

[b] Ninety (90) days after the last dose there is a visit with assessments ***if no alternative therapy has started***. If the participant has started alternative therapy within that 90 days after the last dose, the first follow up visit will be contact by telephone only.

[c] For a maximum of 2 years from the initiation of study intervention, participants will be contacted by telephone every 3 months to determine their survival status.

[d] Cancer history will include stage, date of diagnoses, and prior cancer therapies and procedures.

[e] Pregnancy tests (serum or urine) will be performed at Screening (within 24 hours of first dose), on Day 1 of every cycle thereafter (within 24 hours of dose), and EOT.

[f] Pulse oximetry will be collected with vital signs only at baseline.

[g] Tumor markers (eg, CA19-9, PSA, etc) will be collected if performed as part of standard of care.

[h] cfDNA (blood) will be assessed at Screening, preinfusion Cycle 2 Day 1, and EOT.

[i] Circulating soluble analytes and blood immune biomarkers will be assessed at Screening, preinfusion Cycle 1 Days 1 and 8, Cycles 2-4 Day 1, and EOT.

[j] Stool collection will be assessed at Screening and at Cycle 2 Day 1, if possible. If this timing is not possible, any stool sample collected while on treatment would be acceptable.

[k] Participants will undergo 3 tumor biopsies: mandatory baseline biopsy (for treatment assignment based on percent of tumoral CD8 cells), as early as possible after the 2nd dose of ipilimumab and after the 4th dose of ipilimumab (Day 2 – Day 10 of Cycle 2 and Cycle 6, respectively; however, any on treatment biopsy after Day 1 of Cycle 2 and Cycle 6 will be accepted, if medically feasible). Regardless of dosing, participants should continue to follow assessments as timed.

[l] If the results of the baseline screening biopsy are not adequate to obtain information on the percentage of tumoral CD8 cells for treatment assignment, a 2nd baseline biopsy may be obtained, if it is medically feasible and the participant is willing to undergo another tumor biopsy. Additionally, if the results of the on treatment biopsy are not adequate to obtain information on the percentage of tumoral CD8 cells, a 2nd on treatment biopsy may be obtained, if it is medically feasible and the participant is willing to undergo another tumor biopsy.

[m] All AEs, SAEs, and all concomitant medications will be collected from-the time the participant signs informed consent until 100 days after the last dose of study intervention. Prior to initiation of study intervention, only AEs and SAEs that are related to a protocol mandated intervention, including those that occur

prior to assignment of study procedures (eg, screening invasive procedures, such as biopsies) should be reported. Trial-limiting toxicities can be found in Section 8.3.7.

[n] Nivolumab monotherapy will be administered at 360 mg IV Q3W for 4 cycles. Participants who continue to show clinical benefit after the first disease assessment will receive nivolumab at 480 mg IV Q4W until PD or intolerable toxicity (starting at Cycle 5).

[o] Beginning at Cycle 7, nivolumab maintenance (nivolumab at 480 mg IV Q4W) will be administered until PD or intolerable toxicity.

[p] Scans will be performed within 7 days prior to Day 1 of Cycle 4, Cycle 6, and every ***even*** cycle thereafter until death, disease progression, initiation of another systemic anticancer therapy, loss to follow-up, withdrawal of consent, or study termination, whichever occurs first (See Section 8.1). In addition, ad hoc scans, as part of standard of care, will be collected for participants who have discontinued treatment for reasons other than radiographic progression and prior to any alternate therapies.

Table 3: Schedule of Assessments Advanced Prostate Cancer (Nivolumab Only Arm: Nivolumab 360 mg Q3W and Maintenance 480 mg Q4W)

Tests & Procedures	Screening[a]	On-Treatment Evaluations				End of Treatment	Follow up[b]	
		C1		C2-4	C5 Onwards			
Day	0	D1	D8	D1	D1	15-30 days after last dose	First visit (90 days after last dose)[b]	Q3M for 2 years[c]
Window (days)	-28 to 0	--	± 3	± 3	± 3	± 7	± 7	± 7
Informed consent[a]	X							
Medical, cancer history[d]	X							
Physical examination	X			X	X	X	X	
ECOG performance status	X			X	X	X	X	
Vital signs (see Section 8.2.3)[e]	X	X		X	X	X	X	
Body weight	X	X		X	X			
Hematology (see Table 10)	X		X	X	X	X	X	
Clinical chemistry (see Table 10)	X		X	X	X	X	X	
Urinalysis	X		X	X	X	X	X	
12-lead ECG	X							
PSA[f]	X	X	PSA will be collected every cycle throughout treatment			X		
cfDNA (blood)[g]	X			X[g]		X		
Circulating soluble analytes[h]	X	X	X	X		X		
Blood immune biomarkers[h]	X	X	X	X		X		
Stool collection[i]	X			X[i]				
Tumor biopsy[j]	X[k]			X[j]		X[j]		
Concomitant medications[l]	X	X	X	X	X	X	X	
Adverse events[l]	X	X	X	X	X	X	X	
Nivolumab administration[m]		X		X	X[n]			
Follow-up for overall survival[c]							X	X

Tests & Procedures	Screening[a]	On-Treatment Evaluations				End of Treatment	Follow up[b]	
		C1		C2-4	C5 Onwards			
Day	0	D1	D8	D1	D1	15-30 days after last dose	First visit (90 days after last dose)[b]	Q3M for 2 years[c]
Window (days)	-28 to 0	--	± 3	± 3	± 3	± 7	± 7	± 7
Assess subsequent cancer therapy						X	X	X
Disease assessment[o]	X	Cycle 4 Day 1, then beginning at Cycle 6 Day 1, repeat every 8 weeks (± 7 days) until radiographic progression or the start of subsequent therapy, whichever comes first						
ApricityCare ™ PRO[p]	X	Participant's daily assessment via ApricityCare™ digital smartphone application[p]<br>Study Staff to review participant's data via ApricityOncology™ as per Apricity Health Training Materials[p]						

cfDNA = cell-free deoxyribonucleic acid; Cx = Cycle x; ECG = electrocardiogram; ECOG = Eastern Cooperative Oncology Group; EOT = end of treatment; PD = progressive disease; PRO = patient-reported outcomes; Q3M = every 3 months; Q3W = every 3 weeks; Q4W = every 4 weeks.

[a] Screening evaluations are to be conducted within 28 days of the first dose of protocol therapy. Some screening procedures may occur on Cycle 1 Day 1 as appropriate. Informed consent must be obtained prior to any study-specific procedures and may be obtained prior to the 28-day screening window.

[b] Ninety (90) days after the last dose there is a visit with assessments ***if no alternative therapy has started***. If the participant has started alternative therapy within that 90 days after the last dose, the first follow up visit will be contact by telephone only.

[c] For a maximum of 2 years from the initiation of study intervention, participants will be contacted by telephone every 3 months to determine their survival status.

[d] Cancer history will include stage, date of diagnoses, and prior cancer therapies and procedures.

[e] Pulse oximetry will be collected with vital signs only at baseline.

[f] PSA will be collected during each cycle during treatment. Any PSA assessments collected outside of the schedule, as part of standard of care, will be accepted.

[g] cfDNA (blood) will be assessed at Screening, preinfusion Cycle 2 Day 1, and EOT.

[h] Circulating soluble analytes and blood immune biomarkers will be assessed at Screening, preinfusion Cycle 1 Days 1 and 8, Cycles 2-4 Day 1, and EOT.

[i] Stool collection will be assessed at Screening and at Cycle 2 Day 1, if possible. If this timing is not possible, any stool sample collected while on treatment would be acceptable.

[j] Participants will undergo up to 3 tumor biopsies: mandatory baseline biopsy (for treatment assignment based on percent of tumoral CD8 cells), as early as possible around the 2nd dose of nivolumab (± 3 days of Cycle 2 Day 1; however, any on treatment biopsy around Day 1 of Cycle 2 will be accepted, if medically feasible), and at PD (if medically feasible) prior to the optional addition of ipilimumab. Regardless of dosing (eg, treatment hold), participants should continue to follow assessments as timed.

[k] If the results of the baseline screening biopsy are not adequate to obtain information on the percentage of tumoral CD8 cells for treatment assignment, a 2nd baseline biopsy may be obtained, if it is medically feasible and the participant is willing to undergo another tumor biopsy. Additionally, if the results of the on treatment biopsy are not adequate to obtain information on the percentage of tumoral CD8 cells, a 2nd on treatment biopsy may be obtained, if it is medically feasible and the participant is willing to undergo another tumor biopsy.

[l] All AEs, SAEs, and all concomitant medications will be collected from-the time the participant signs informed consent until 100 days after the last dose of study intervention. Prior to initiation of study intervention, only AEs and SAEs that are related to a protocol mandated intervention, including those that occur prior to assignment of study procedures (eg, screening invasive procedures, such as biopsies) should be reported.

[m] Nivolumab monotherapy will be administered at 360 mg IV Q3W for 4 cycles. Participants who continue to show clinical benefit after the first disease assessment will receive nivolumab at 480 mg IV Q4W until PD or intolerable toxicity (starting at Cycle 5).

[n] Beginning at Cycle 5, nivolumab maintenance (nivolumab at 480 mg IV Q4W) will be administered until PD or intolerable toxicity.

[o] Scans will be performed within 7 days prior to Day 1 of Cycle 4, Cycle 6 and every 8 weeks thereafter until death, disease progression, initiation of another systemic anticancer therapy, loss to follow-up, withdrawal of consent, or study termination, whichever occurs first (See Section 8.1). In addition, ad hoc scans, as part of standard of care, will be collected for participants who have discontinued treatment for reasons other than radiographic progression and prior to any alternate therapies.

[p] Optional procedure for participants who consent to participate in the digital PRO using a smartphone application, from consent up to 100 days after the last dose of study intervention. Study Staff to review participant's data via ApricityOncology™, a clinical dashboard website at regular intervals per training materials.

Table 4: Schedule of Assessments Advanced Prostate Cancer (Nivolumab and Ipilimumab Arm: Nivolumab Q3W and Ipilimumab Q6W [Cycles 1 and 2], Followed by Single-agent Nivolumab Q4W)

Tests & Procedures	Screening[a]	On-Treatment Evaluations						End of Treatment	Follow-up (After Date Off Treatment)	
		C1			C2		C3 Onward			
Day	0	D1	D8	D22	D1	D22	D1	15-30 days after last dose	First visit (90 days after last dose)[b]	Q3M for 2 years[c]
Window (days)	-28 to 0	--	± 3	± 3	± 3	± 3	± 3	± 7	± 7	± 7
Informed consent[a]	X									
Medical, cancer history[d]	X									
Physical examination	X			X	X	X	X	X	X	
ECOG performance status	X			X	X	X	X	X	X	
Vital signs (see Section 8.2.3)[e]	X	X		X	X	X	X	X	X	
Body weight	X	X		X	X	X	X			
Hematology (see Table 10)	X		X	X	X	X	X	X	X	
Clinical chemistry (see Table 10)	X		X	X	X	X	X	X	X	
Urinalysis	X		X	X	X	X	X	X	X	
12-lead ECG	X									
PSA[f]	X	X	PSA will be collected every cycle throughout treatment					X		
cfDNA (blood)[g]	X			X				X		
Circulating soluble analytes[h]	X	X	X	X	X	X		X		
Blood immune biomarkers[h]	X	X	X	X	X	X		X		
Stool collection[i]	X			X[i]						
Tumor biopsy[j]	X[k]			X[j]		X[j]		X (optional)		
Concomitant medications	X	X	X	X	X	X	X	X	X	
Adverse events[l]	X	X	X	X	X	X	X	X	X	
Nivolumab administration[m]		X		X	X	X	X[n]			
Ipilimumab administration[m]		X			X					
Follow-up for overall survival[c]									X	X

Tests & Procedures	Screening[a]	On-Treatment Evaluations						End of Treatment	Follow-up (After Date Off Treatment)	
		C1			C2		C3 Onward			
Day	0	D1	D8	D22	D1	D22	D1	15-30 days after last dose	First visit (90 days after last dose)[b]	Q3M for 2 years[c]
Window (days)	-28 to 0	--	± 3	± 3	± 3	± 3	± 3	± 7	± 7	± 7
Assess subsequent cancer therapy								X	X	X
Disease assessment[o]	X	Beginning at Cycle 2 Day 22, repeat every 8 weeks (± 7 days) until radiographic progression or the start of subsequent therapy, whichever comes first								
ApricityCare™ PRO[p]	X	Participant's daily assessment via ApricityCare™ digital smartphone application[p]<br>Study Staff to review participant's data via ApricityOncology™ as per Apricity Health Training Materials[p]								

cfDNA = cell-free deoxyribonucleic acid; Cx = Cycle x; ECG = electrocardiogram; ECOG = Eastern Cooperative Oncology Group; PRO = patient-reported outcomes; Q3M = every 3 months; Q3W = every 3 weeks; Q4W = every 4 weeks; Q6W = every 6 weeks.

[a] Screening evaluations are to be conducted within 28 days of the first dose of protocol therapy. Some screening procedures may occur on Cycle 1 Day 1 as appropriate. Informed consent must be obtained prior to any study-specific procedures and may be obtained prior to the 28-day screening window.

[b] Ninety (90) days after the last dose there is a visit with assessments ***if no alternative therapy has started***. If the participant has started alternative therapy within that 90 days after the last dose, the first follow up visit will be contact by telephone only.

[c] For a maximum of 2 years from the initiation of study intervention, participants will be contacted by telephone every 3 months to determine their survival status.

[d] Cancer history will include stage, date of diagnoses, and prior cancer therapies and procedures.

[e] Pulse oximetry will be collected with vital signs only at baseline.

[f] PSA will be collected during each cycle during treatment. Any PSA assessments collected outside of the schedule, as part of standard of care, will be accepted.

[g] cfDNA (blood) will be assessed at Screening, preinfusion Cycle 1 Day 22 and EOT.

[h] Circulating soluble analytes and blood immune biomarkers will be assessed at Screening, preinfusion Cycle 1 Days 1, 8, and 22 and Cycle 2 Days 1 and 22, and EOT.

[i] Stool collection will be assessed at Screening and at Cycle 1 Day 22, if possible. If this timing is not possible, any stool sample collected while on treatment would be acceptable.

[j] Participants will undergo up to 4 tumor biopsies: mandatory baseline biopsy (for treatment assignment based on percent of tumoral CD8 cells), ± 3 days of the 2nd dose of nivolumab and within 3 days of the 4th dose of nivolumab (Day 22 of Cycles 1 and 2, respectively; however, any on treatment biopsy after Day 22 of Cycles 1 and 2 will be accepted, if medically feasible), biopsy may occur prior to dosing. Regardless of dosing, participants should continue to follow assessments as timed. Additionally, an optional tumor biopsy at the time of progression may be obtained.

[k] If the results of the baseline screening biopsy are not adequate to obtain information on the percentage of tumoral CD8 cells for treatment assignment, a 2nd baseline biopsy may be obtained, if it is medically feasible and the participant is willing to undergo another tumor biopsy. Additionally, if the results of the on treatment biopsy are not adequate to obtain information on the percentage of tumoral CD8 cells, a 2nd on treatment biopsy may be obtained, if it is medically feasible and the participant is willing to undergo another tumor biopsy.

[l] All AEs, SAEs, and all concomitant medications will be collected from-the time the participant signs informed consent until 100 days after the last dose of study intervention. Prior to initiation of study intervention, only AEs and SAEs that are related to a protocol mandated intervention, including those that occur prior to assignment of study procedures (eg, screening invasive procedures, such as biopsies) should be reported. Trial-limiting toxicities can be found in Section 8.3.7.

[m] Nivolumab will be administered in combination with ipilimumab. Two cohorts will receive combination therapy. Prostate Cohort A will receive nivolumab **1 mg/kg Q3W (4 doses over 2 cycles)** and ipilimumab **3 mg/kg Q6W (2 doses over 2 cycles)**. Participants will then receive nivolumab at **480 mg IV Q4W** until PD or intolerable toxicity (starting at Cycle 3). Prostate Cohort B will receive nivolumab **1 mg/kg Q3W (4 doses over 2 cycles)** and ipilimumab **5 mg/kg Q6W (2 doses over 2 cycles**). Participants will then receive nivolumab at **480 mg IV Q4W** until PD or intolerable toxicity (starting at Cycle 3).

[n] Beginning at Cycle 3, nivolumab maintenance (nivolumab at 480 mg IV Q4W) will be administered until PD or intolerable toxicity.

[o] Scans will be performed within 7 days prior to Day 22 of Cycle 2, then every 8 weeks thereafter until death, disease progression, initiation of another systemic anticancer therapy, loss to follow-up, withdrawal of consent, or study termination, whichever occurs first (See Section 8.1). In addition, ad hoc scans, as part of standard of care, will be collected for participants who have discontinued treatment for reasons other than radiographic progression and prior to any alternate therapies.

[p] Optional procedure for participants who consent to participate in the digital PRO using a smartphone application, from consent up to 100 days after the last dose of study intervention. Study Staff to review participant's data via ApricityOncology™, a clinical dashboard website at regular intervals per training materials.

2 INTRODUCTION

2.1 STUDY RATIONALE

The aim of this study is to provide a prospective classification of CD8 high (immunologically "hot") versus CD8 low (immunologically "cold") tumors at the time of treatment, based on the percentage of CD8 cells in a tumor biopsy, and to address the predictive value of the CD8 biomarker for selecting patients for treatment with nivolumab with or without ipilimumab.

2.2 BACKGROUND

Immuno-oncology is a promising new area for cancer therapeutics. The immune system is capable of exquisite adaptation and selective targeting, a process that is now being harnessed and directed towards advanced cancer. Immuno-oncology therapies manipulate the immune response against cancer in several ways. Vaccines have been developed with the goal of priming the cellular and humoral immune response towards specific cancer antigens. Other therapies target the specific mechanisms that cancer cells use to avoid detection by the host immune system. These evasion mechanisms take advantage of immune "checkpoints" that regulate the cellular immune response. Many cancer types hijack these mechanisms thereby rendering the immune system unable to mount an effective antitumor immune response.

2.2.1 Immune Checkpoint Inhibitors

Immune checkpoint inhibitors, such as ipilimumab and nivolumab, have resulted in remarkable clinical benefits, including durable clinical responses and improved survival, in several tumor types. However, despite this important advancement, the benefit of immuno-oncology is limited to a minority of patients and only in a few indications. For example, following treatment with a programmed cell death-1 (PD-1) antibody, 31% to 34% of advanced melanoma patients (Weber et al, 2015; Robert et al, 2015), 19% to 20% of non-small cell lung cancer (NSCLC) patients (Borghaei et al, 2015; Brahmer et al, 2015; Herbst et al, 2016), and 25% of renal cell carcinoma (RCC) patients (Motzer et al, 2015) have durable objective (partial or complete) responses and improved overall survival (OS) compared with conventional therapies. Ipilimumab, a human monoclonal antibody (mAb) that blocks cytotoxic T-lymphocyte-associated antigen-4 (CTLA-4) on T cells (approved in 2011), achieves durable clinical responses in ~10% of patients with advanced melanoma and 5-year OS rates of ~18% (Hodi et al, 2010; Schadendorf et al, 2015; Maio et al, 2015). Both nivolumab and pembrolizumab, which are anti-PD-1 mAbs (approved in 2014), achieve clinical responses in 31% to 34% of advanced melanoma patients, and most responses are durable (Weber et al, 2015; Robert et al, 2015). While the activity observed with single-agent anti-PD-1 mAbs is significant, combined treatment with ipilimumab and nivolumab (approved in 2015) results in clinical

responses of 55% to 60% in metastatic melanoma patients (Ribas et al, 2017; Larkin et al, 2015). Similarly, nivolumab plus ipilimumab also showed significantly higher ORR compared with sunitinib (42% vs 27%) in previously untreated advanced RCC patients (Motzer et al, 2018). However, the improvement in clinical activity achieved with nivolumab in combination with ipilimumab is accompanied by increased side effects; Grade 3 or 4 events occurred in 59% of patients in the nivolumab plus ipilimumab group, in 21% of those in the nivolumab group, and in 28% in the ipilimumab group (Wolchok et al, 2017). Thus, there is a critical need for biomarkers that can predict which cancer patients will benefit from ipilimumab and nivolumab combination therapy.

2.2.2 Biomarkers to Predict Benefit from Treatment with Immune Checkpoint Inhibitors

Certain tumors are primed and ready to respond to treatment with an anti-PD-1 antibody, and others are not. Cancer patients who benefit from anti-PD-1 treatment are characterized as having “hot” tumors, whereas those who do not respond to anti-PD-1 treatment are characterized as having “cold” tumors. Several biomarkers have been proposed to classify a tumor as “hot” versus “cold,” including programmed cell death ligand-1 (PD-L1) expression, tumor mutation burden (Rizvi et al, 2015), interferon-gamma expression (Ayers et al, 2017), tumor immune signature (Chen et al, 2016), fecal microbiome profile (Gopalakrishnan et al, 2018), and the extent of the CD8 T-cell infiltrate (Hegde et al, 2016). These biomarkers have an association with the CD8 T-cell infiltrate (Maby et al, 2015; Thompson et al, 2017; Roy and Trinchieri, 2017). Several clinical studies have shown that clinical response to anti-PD-1 treatment is correlated with the pre-treatment number or the percentage of tumoral CD8 cells (Tumeh et al, 2014; Chen et al, 2016; Johnson et al, 2016). Therefore, larger numbers of tumoral CD8 cells may be a useful biomarker to identify “hot” (CD8 high) tumors.

Furthermore, an increase in tumoral CD8 cells has been observed after anti-PD-1 treatment, and this increase is associated with improved clinical outcomes in melanoma and squamous cell carcinoma of the head and neck (SCCHN; Chen et al, 2016; Concha-Benavente et al, 2017). Following anti-PD-1 treatment, a significant increase in intratumoral CD8 cells was seen in responders but not in non-responders (Figure 5).

Figure 5: Tumor-infiltrating CD8 T-cell Expression in Responders and Non-responders to PD-1 Blockade

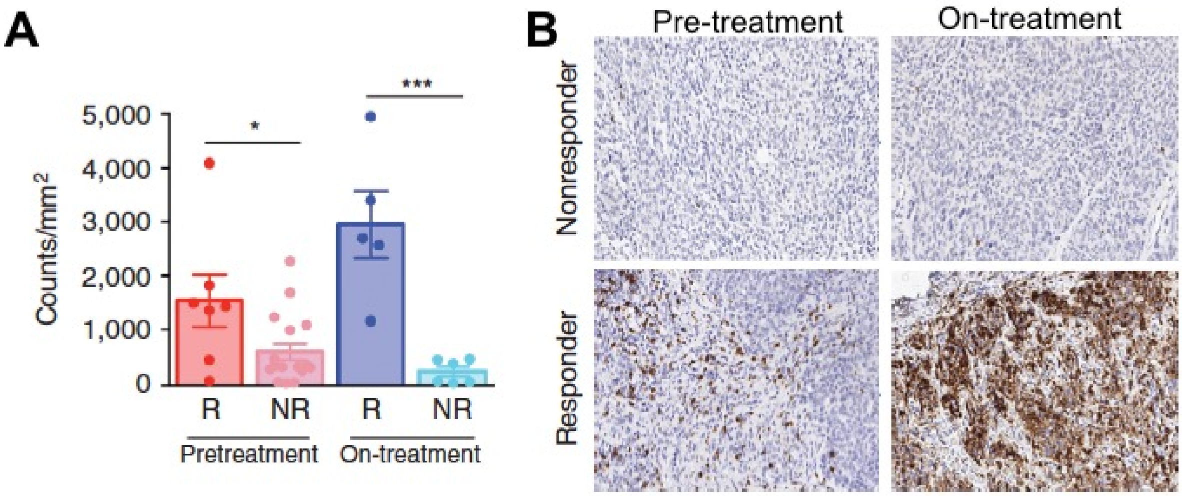

Tumor samples were stained by immunohistochemistry with pooled data in panel A and representative images in panel B. Adapted from Chen et al. 2016.

Treatment with ipilimumab monotherapy also results in an increase in CD4 and CD8 T cells in the tumor (Gao et al, 2017; Kvistborg et al, 2014). In a preclinical tumor model, anti-CTLA-4 treatment of tumor-bearing animals resulted in a significant increase in intravascular CXCL9 and E-selectin expression, which are involved in mediating CD8 T-cell extravasation and entry into the tumor (Wilson et al, 2016). Therefore, adding ipilimumab to anti-PD-1 treatment could further promote the trafficking of CD8 T cells into the tumor microenvironment. This hypothesis is supported by recent finding in a small subset of participants from the CheckMate 038 study in which the combination of ipilimumab and nivolumab significantly induced higher percentage of tumoral CD8 cells compared with nivolumab alone (Figure 6; Ribas et al, 2017). In addition, the combination of ipilimumab and nivolumab yielded a higher response rate compared with nivolumab alone (Larkin et al, 2015).

Figure 6: Tumor-infiltrating CD8 T-cell Expression by Best Overall Response

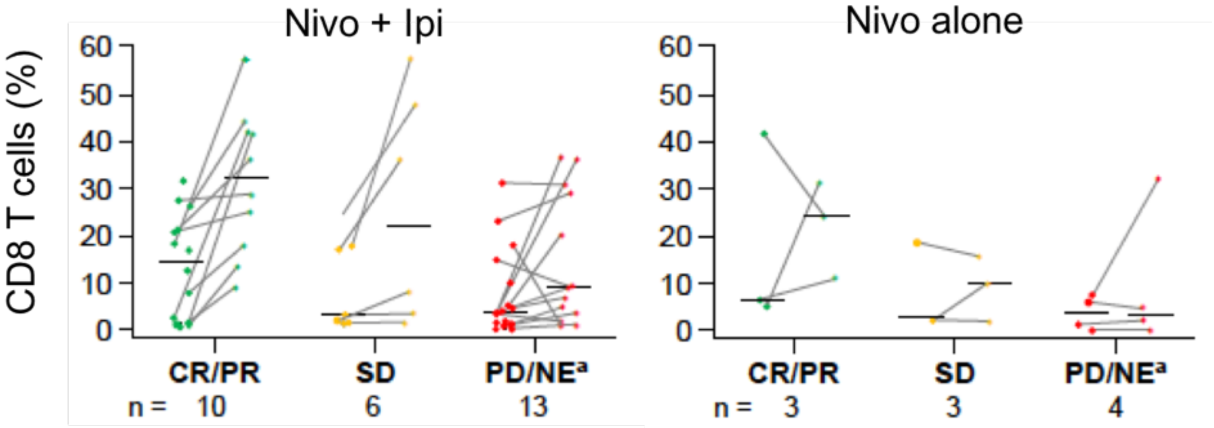

CR = clinical response; Ipi = ipilimumab; NE = not evaluable; Nivo = nivolumab; PD = progressive disease; PR = partial response; SD = stable disease.
Biomarker study on Phase 1b CheckMate 038 study and tumor biopsies were assessed by immunohistochemistry.
Adapted from Ribas oral presentation at 2017 AACR.

In summary, both pre-treatment tumoral CD8 cells and recruitment of CD8 T cells in response to immune checkpoint inhibitors are associated with improved clinical outcomes in patients treated with anti-PD-1 therapy. These data suggest that a rational approach to convert "CD8 low tumors into "CD8 high tumors is through combination treatment approaches with anti-PD-1 and anti-CTLA-4 mAbs that can recruit more CD8 T cells to the tumor (Haanen, 2017).

A prospective study with larger sample size is warranted to confirm that the percentage of tumoral CD8 cells at the time of treatment can determine responsiveness to anti-PD-1 therapy and whether combination therapy can convert tumors from CD8 low to CD8 high. We propose a prospective clinical study in which patients with varying advanced solid tumors will be assigned to 1 of 2 treatment arms based on the percentage of tumoral CD8 cells at the time of treatment. Participants who have a tumor with ≥ 15% CD8 cells (classified as CD8 high) will receive nivolumab monotherapy, and participants who have a tumor with < 15% CD8 cells (classified as CD8 low) will receive ipilimumab in combination with nivolumab.

It is well recognized that immune infiltration varies across tumor types, and different tumor types may display a range of therapeutic sensitivities. Several ongoing studies are evaluating nivolumab treatment in various tumor types (BMS Nivolumab Studies). To better assess the feasibility of using percent of tumoral CD8 cells as a biomarker for selecting checkpoint inhibitor therapy and to gain more insight regarding the design of the trial, we examined CD8 cells in a set of 195 tumor samples from 10 different tumor types (Figure 7). For tumor types in which anti-PD-1 treatment has been approved, the classification of CD8 high tumor is consistent with the clinical benefit reported. The 15% cutoff was selected based on data from retrospective

studies and evaluation of responses to checkpoint inhibitors. This study will advance the field by providing a prospective classification of CD8 high versus CD8 low tumors at the time of treatment, based on response to nivolumab, and address the predictive value of the CD8 biomarker for selecting patients who can benefit from nivolumab with or without ipilimumab.

Figure 7: Percentage of Tumoral CD8 T Cells by Immunohistochemistry in Different Tumor Types

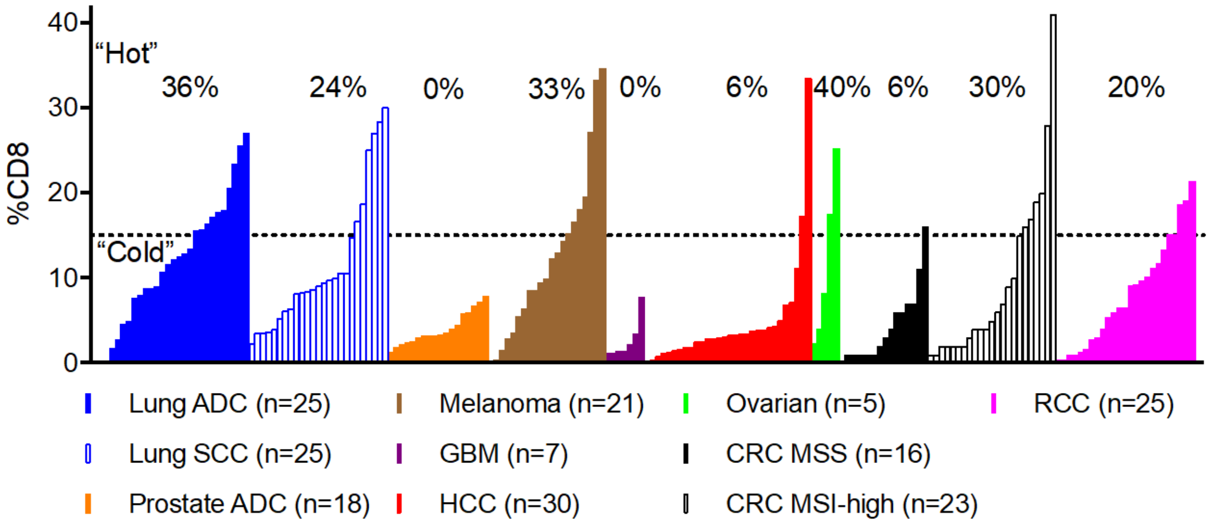

ADC = adenocarcinoma; CRC = colorectal cancer; GBM = glioblastoma multiforme; HCC = hepatocellular carcinoma; IHC = immunohistochemistry; MSI = microsatellite instable; MSS = microsatellite stable; RCC = renal cell carcinoma; SCC = squamous cell carcinoma.

IHC is performed on formalin-fixed paraffin-embedded (FFPE) tumor samples obtained from a cohort of untreated cancer patients. Tumor tissues were fixed in 10% formalin, embedded in paraffin, and transversely sectioned; 4-µm sections were used for the IHC study. Sections were stained with mouse anti-human monoclonal antibodies against CD8 (Thermo Scientific, MS-457-S). Quantitative analysis of IHC staining was conducted using the image analysis software ImageScope-Aperio/Leica. Five random areas (1 mm^2 each) were selected, and a customized software algorithm for each immunologic marker was used to determine the percentage of positive cells per high-power field. The data are expressed as a percentage score (total number of positive cells divided by the total number of cells and multiplied by 100). IHC staining was interpreted in conjunction with hematoxylin and eosin stained sections, which facilitated the identification of malignant cells and thus directed IHC quantification.

2.2.3 Immune Checkpoint Inhibitor Therapies in the Treatment of Advanced Prostate Cancer

Immune checkpoint therapies targeting the CTLA-4 and/or PD-(L)1 pathway have demonstrated a survival benefit in multiple malignancies. Single agent ipilimumab and nivolumab have been studied in multiple clinical and preclinical studies across many tumor types; however, both have had limited clinical efficacy as monotherapies in patients with advanced prostate cancer (APC).

Clinical activity has been observed in subsets of APC patients in a number of clinical trials, including a phase 1/2 study in participants with metastatic castration-resistant prostate cancer

(mCRPC) that explored ipilimumab as monotherapy and in combination with radiotherapy, based on the preclinical evidence of synergistic antitumor activity between anti-CTLA-4 antibody and radiotherapy. While evaluating adverse events, prostate-specific antigen (PSA) decline, and tumor response, it was found that ipilimumab every 3 weeks for 4 doses at 3, 5, or 10 mg/kg ± radiotherapy suggested clinical antitumor activity with disease control and manageable AEs (Slovin et al., 2013).

Data have shown that ipilimumab dosing up to 10 mg/kg in mCRPC (with/without stereotactic body radiation therapy) has a manageable safety profile. Metastatic castration-resistant prostate cancer participants who had received docetaxel were evaluated in a randomized clinical trial with ipilimumab versus placebo (NCT00861614). Although the study failed to meet the primary endpoint of improving overall survival, it demonstrated improved PFS and PSA response in those participants administered ipilimumab every 3 weeks for up to 4 doses compared to placebo (Kwon et al., 2014). Post-study analyses suggested that ipilimumab improved survival in patients without visceral metastasis, who had lower levels of alkaline phosphatase and had elevated hemoglobin levels at baseline (Drake et al., 2014).

In a double-blind clinical trial evaluating 598 APC patients, chemotherapy-naive participants were randomized 2:1 to receive ipilimumab at 10 mg/kg (n = 399) or placebo (n = 199) every 3 weeks for up to 4 doses, with ipilimumab at 10 mg/kg or placebo maintenance given to non-progressing patients every 3 months(NCT01057810). Although ipilimumab alone did not improve overall survival in those advanced prostate cancer patients, increases in progression-free survival and prostate-specific antigen response rates were observed, suggesting antitumor activity in a subset of patients with chemotherapy-naive metastatic castration-resistant prostate cancer without visceral metastases. (Beer et al., 2017).

In these 2 large phase 3 trials (Kwon et al., 2014; Beer et al., 2017), ipilimumab (anti-CTLA-4; 10 mg/kg Q4W) failed to improve survival in men with mCRPC, which may be attributed to the immunologically “cold” prostate tumor microenvironment, characterized by low tumor mutational burden, paucity of T cells, and high frequency of immunosuppressive cells. Interestingly, in tumors from patients in a presurgical clinical trial ipilimumab induced intratumoral T cell infiltration; however, this was countered by increased expression of the immune checkpoint, PD-L1, within the prostate tumor microenvironment (Gao et al., 2017).

This led to the design and accrual of the CheckMate 650 clinical trial (NCT02985957), which recently demonstrated promising durable antitumor responses in a subset of patients with mCRPC treated with the combination of nivolumab plus ipilimumab using the approved melanoma dosing and schedule (nivolumab 1 mg/kg and ipilimumab 3 mg/kg Q3W up to 4 cycles, then nivolumab 480 mg Q4W) (Sharma et al., 2019).

The safety profile of ipilimumab in CRPC has been well characterized as a monotherapy at doses up to 10 mg/kg (Kwon et al., 2014; Beer et al., 2017) or in combination with radiation, at doses up to 5 mg/kg with vaccines (Madan et al., 2012) and in combination with nivolumab at ipilimumab doses of 1 and 3 mg/kg (CheckMate-650, Sharma et al., 2019). While side effects are manageable, data from other tumor types, such as NSCLC (CheckMate-227, Hellmann et al., 2019; CheckMate-568, Ready et al., 2019), suggest that less frequent dosing of ipilimumab may improve tolerability when administered in combination with nivolumab.

Checkpoint inhibitor treatment benefit can be associated with the presence of CD8 T cells in the tumor, and CTLA-4 treatment has been shown to promote T cell infiltration, thus hypothesizing that the combination of ipilimumab and nivolumab may promote tumoral T cell infiltration and antitumor immunity.

Preclinical and clinical data support that ipilimumab promotes T cell infiltration in the tumor, yet the necessary dosing and scheduling of treatments remain unclear. Dose-finding studies for single-agent ipilimumab have been studied in multiple clinical and preclinical studies. Clinical responses are consistently observed at ipilimumab levels of ≥ 3 mg/kg, and analysis from melanoma clinical studies suggest that tumor responses improve from 3 to 10 mg/kg. The hypothesis that ipilimumab triggers long-lasting antitumor immunity suggests that providing it earlier during the course of prostate cancer may provide a more meaningful benefit (Kwek et al., 2012; Madan et al., 2009).

Preliminary evidence from advanced prostate cancer participants enrolled in the ipilimumab and nivolumab arm of PICI0025 has demonstrated clinical activity in tumors with a low CD8 levels in the tumor at baseline, including an increase in the T cell infiltration. We postulate that a low dose of ipilimumab in such tumors may provide a moderate CD8 infiltration (NCT03651271) and that higher doses of ipilimumab may result in a higher number of participants having increased CD8 T cell infiltration, and that the combination of nivolumab and ipilimumab may result in improved antitumor immunity (NCT03651271).

2.3 BENEFIT/RISK ASSESSMENT

Nivolumab has demonstrated durable responses exceeding 6 months as monotherapy and in combination with ipilimumab in several tumor types, including NSCLC, melanoma, RCC, and SCCHN. In confirmatory trials, nivolumab as monotherapy demonstrated a statistically significant improvement in OS compared with the current standard of care in participants with previously treated advanced or metastatic NSCLC, unresectable or metastatic melanoma, previously treated advanced RCC, or previously treated recurrent or metastatic SCCHN. Nivolumab in combination with ipilimumab improved progression-free survival (PFS) and objective response rate (ORR) over ipilimumab alone in participants with unresectable or

metastatic melanoma (Ribas et al, 2017; Larkin et al, 2015). Similarly, nivolumab plus ipilimumab demonstrated significantly higher OS and ORR compared with sunitinib in intermediate- and poor-risk patients with previously untreated advanced RCC (Motzer et al, 2018).

The overall safety experience with nivolumab, as monotherapy or in combination with other therapeutics, is based on experience in approximately 20,000 patients treated to date (Nivolumab Investigator's Brochure, 2020). For monotherapy, the safety profile is similar across tumor types. There is no pattern in the incidence, severity, or causality of adverse events (AEs) to nivolumab dose level. In phase 3 controlled studies, the safety profile of nivolumab monotherapy is acceptable in the context of the observed clinical efficacy and manageable using established safety guidelines. Clinically relevant AEs typical of stimulation of the immune system were infrequent and manageable by delaying or stopping nivolumab treatment and using timely immunosuppressive therapy or other supportive care.

In several ongoing clinical trials, the safety of nivolumab in combination with other therapeutics such as ipilimumab, cytotoxic chemotherapy, anti-angiogenics, and targeted therapies is being explored. Most studies are ongoing, and, as such, the safety profile of nivolumab combinations continues to evolve. The most advanced combination under development is nivolumab in combination with ipilimumab, which is approved in patients with unresectable or metastatic melanoma and being studied in multiple tumor types. Results to date suggest that the safety profile of nivolumab and ipilimumab combination therapy is consistent with the mechanisms of action of nivolumab and ipilimumab. The nature of the AEs is similar to that observed with either agent used as monotherapy; however, both frequency and severity of most AEs are increased with the combination.

Overall, these findings support a favorable benefit-risk profile for nivolumab with or without ipilimumab across various tumor types. More detailed information about the known and expected benefits and risks and reasonably expected AEs of nivolumab and ipilimumab may be found in the respective Investigator's Brochures (IB; Ipilimumab IB, 2020; Nivolumab IB, 2020).

3 OBJECTIVES AND ENDPOINTS

The study objectives and endpoints are listed in Table 5.

Table 5: Objectives and Corresponding Endpoints

Objectives	Endpoints
Primary	
• To determine the clinical benefit rate (CBR) of nivolumab with or without ipilimumab in participants with advanced metastatic cancer, including advanced prostate cancer.<br>• To assess the proportion of participants in the nivolumab plus ipilimumab arm whose tumors will change from CD8 low to CD8 high as measured by a change in the percentage of tumoral CD8 cells.	• CBR is the proportion of participants who show clinical benefit, defined as CR, PR, or SD for ≥ 6 months as best response by RECIST v1.1.<br>• Change in the percentage of CD8 cells in on-treatment biopsies from baseline.
Secondary	
• To determine the safety and tolerability of nivolumab with or without ipilimumab in participants with advanced metastatic cancer, including advanced prostate cancer.<br>• To determine the ORR of nivolumab with or without ipilimumab in participants with advanced metastatic cancer, including advanced prostate cancer.<br>• To assess the association of percentage of CD8 infiltration in tumor samples with clinical outcomes (ORR, PFS, and OS).	• Incidence and severity of AEs based on CTCAE v5.0.<br>• ORR: Defined as CR or PR as best response by RECIST v1.1 assessment.<br>• PFS: Defined as the time from initiation of study therapy to date of first documented progression of disease or date of death due to any cause.<br>• OS: Defined as the time from initiation of study therapy until death due to any cause.
Exploratory	
• To evaluate tumor immune biomarkers and their association with treatment outcomes.<br>• To evaluate changes in prostate-specific antigen (PSA) (advanced prostate cancer).<br>• To assess feasibility of implementing the ApricityCare™ digital application (app) for participants being treated with immune checkpoint inhibitor(s) and the use by study staff of ApricityOncology™ website.	• Clinical responses based on a composite biomarker derived from nucleic acids, epigenetics, protein, immune cell characteristics, or other factors (CD8 and other biomarkers).<br>• Additional biomarkers of primary resistance to nivolumab or ipilimumab combined with nivolumab beyond the percentage of tumoral CD8 cells based on additional immune evaluation of baseline and on-treatment biopsies or blood samples.<br>• Potential relationship of T-cell phenotypic characteristics and immune characteristics in the tumor microenvironment in participants on nivolumab monotherapy and nivolumab in combination with ipilimumab with clinical outcomes (clinical benefit endpoints and AEs with an interest in immune-related AEs).<br>• Within each study intervention arm, explore the potential relationship of clinical response/resistance/AE with:<br>o mRNA quantity and expression in tumor and/or PBMCs

Objectives	Endpoints
	o Germline DNA and/or tumor genomics<br>o Multiparameter cytometry of tumor and/or PBMCs<br>o Immune markers in PBMCs<br>o Blood circulating analytes<br>o TCR repertoire in tumor and peripheral blood<br>o Baseline and on-treatment microbiome<br>• Identify molecular (genomic, metabolic, and/or proteomic) biomarkers that may be indicative of clinical response/resistance, safety, pharmacodynamic activity, and/or mechanism of action of nivolumab and/or ipilimumab in combination with nivolumab.<br>• Change in PSA from baseline (advanced prostate cancer).<br>• Frequency of ApricityCare™ app usage by participants and frequency of ApricityOncology website access by study staff.

AE = adverse event; CBR = clinical benefit rate; CR = complete response; CTCAE = Common Terminology Criteria for Adverse Events; DNA = deoxyribonucleic acid; mRNA = messenger ribonucleic acid; ORR = objective response rate; OS = overall survival; PBMC = peripheral blood mononuclear cell; PD = progressive disease; PFS = progression-free survival; PR = partial response; PSA = prostate-specific antigen; RECIST = Response Evaluation Criteria in Solid Tumors; SD = stable disease; TCR = T-cell receptor.

4 STUDY DESIGN

4.1 OVERALL DESIGN

After consenting to participate in this clinical trial, participants will undergo tumor biopsies. Core needle or incisional biopsies will be required. Fine needle aspiration is not acceptable. The tumor tissue will be sent to University of Texas MD Anderson Cancer Center (MDACC) Clinical Laboratory Improvement Amendment (CLIA)- and College of American Pathologists (CAP)-certified Immunohistochemistry and Image Analysis Laboratories to determine the percentage of tumoral CD8 cells. The test for CD8 expression is a laboratory developed test used to determine participant eligibility. This test is not a Food and Drug Administration (FDA)-approved device and its use is investigational. Participants will be assigned immunotherapy according to the percentage of CD8 cells in their tumor biopsies:

- Participants with ≥ 15% CD8 cells in their tumor biopsies (ie, CD8 high tumors) will be treated with single-agent nivolumab
- Participants with < 15% CD8 cells in their tumor biopsies (ie, CD8 low tumors) will be treated with nivolumab in combination with ipilimumab

Participants receiving single-agent nivolumab will be monitored for response and safety. Participants will undergo 3 tumor biopsy procedures to assess immune markers:

1. Prior to beginning protocol therapy (ie, baseline biopsy, mandatory)
    a. If the results of the baseline biopsy are not adequate to obtain information on the percentage of tumoral CD8 cells for treatment assignment, a second baseline biopsy may be obtained, if it is medically feasible and the participant is willing to undergo another tumor biopsy
2. During treatment (ie, on-treatment biopsy; mandatory if medically feasible) at up to 2 time points:
    a. After receiving the second dose of nivolumab
    b. If the results of the on-treatment biopsy are not adequate to obtain information on the percentage of tumoral CD8 cells, a second on treatment biopsy may be obtained, if it is medically feasible and the participant is willing to undergo another tumor biopsy
3. At the time of progressive disease (PD) prior to the addition of ipilimumab to nivolumab (if medically feasible, in participants who elect to receive combination therapy)

Participants receiving nivolumab in combination with ipilimumab will also be monitored for response and safety. Participants will undergo 3 tumor biopsy procedures to assess immune markers:

1. Prior to beginning protocol therapy (ie, baseline biopsy, mandatory)
    a. If the results of the baseline biopsy are not adequate to obtain information on the percentage of tumoral CD8 cells for treatment assignment, a second baseline biopsy may be obtained, if it is medically feasible and the participant is willing to undergo another tumor biopsy
2. During treatment (ie, on-treatment biopsy, mandatory if medically feasible) at 2 time points:
    a. After receiving the second dose of ipilimumab
    b. After receiving the 4th dose of ipilimumab. Participants who respond to treatment will continue to receive single-agent nivolumab.
    c. If the results of either on-treatment biopsy are not adequate to obtain information on the percentage of tumoral CD8 cells, an additional on-treatment biopsy may be obtained, if it is medically feasible and the participant is willing to undergo another tumor biopsy. An optional tumor biopsy sample may be collected upon disease progression.

4.1.1 Advanced Prostate Cancer

1. Participants with advanced prostate cancer who are receiving nivolumab in combination with ipilimumab will be monitored for response and safety. Participants will undergo

on-treatment tumor biopsy procedures to assess immune markers according to the schedule:

a. Within ± 3 days of the second dose of nivolumab (mandatory if medically feasible)
b. A second biopsy, if medically feasible, should be obtained at Cycle 2 Day 22 (± 3 days of dose administration). An optional tumor biopsy sample may be collected upon disease progression.

The study design schema for advanced metastatic cancer are shown in Figure 1 and Figure 2 and for advanced prostate cancer are shown in Figure 3 and Figure 4.

4.2 SCIENTIFIC RATIONALE FOR STUDY DESIGN

The study population rationale is provided in Section 2, and the analysis groups are described in Section 9.

4.3 JUSTIFICATION FOR DOSE

4.3.1 Rationale for Nivolumab Monotherapy Dose and Schedule

Single-agent nivolumab will be administered at 360 mg intravenously (IV) every 3 weeks (Q3W). Participants who continue to show clinical benefit after the first disease assessment will receive nivolumab 480 mg IV every 4 weeks (Q4W) until PD or intolerable toxicity. At PD, participants will be allowed to add ipilimumab.

Nivolumab 360 mg Q3W Dosing Regimen

The nivolumab 360 mg Q3W dosing regimen was selected based on results from population pharmacokinetic (PPK) and exposure-response analysis modeling and simulation approaches such that they are predicted to provide approximately equivalent exposures (time-averaged steady-state concentration [C_{avgss}]) as nivolumab 3 mg/kg every 2 weeks (Q2W). The model predicted that, following administration of nivolumab 360 mg Q3W, C_{avgss} is expected to be similar to those following nivolumab 3 mg/kg or 240 mg Q2W, while minimum steady-state concentration (C_{minss}) is predicted to be 6% lower and is not considered to be clinically relevant. Following nivolumab 360 mg Q3W, maximum steady-state concentration (C_{maxss}) is predicted to be approximately ~23% greater relative to that following nivolumab 3 mg/kg Q2W dosing; however, the range of nivolumab exposures (median and 90% prediction intervals) following administration of 360 mg Q3W across the 35 to 160 kg weight range is predicted to be maintained well below the corresponding exposures observed with the well-tolerated 10 mg/kg nivolumab Q2W dosing regimen and is not considered to put participants at increased risk. Additionally, the nivolumab Q3W dosing regimen was selected for both treatment arms to allow consistency in timing for obtaining on-treatment biopsies.

Nivolumab 480 Q4W Dosing Regimen

The nivolumab dose of 480 mg Q4W was selected based on clinical data and modeling and simulation approaches using PPK and exposure-response analyses examining relationships between nivolumab exposures and efficacy (eg, OS, objective response) and safety responses, using data from studies in multiple tumor types (melanoma, NSCLC, and RCC) where body weight-normalized dosing (mg/kg) has been used. The PPK analyses have shown that exposure to nivolumab increased dose proportionally over the dose range of 0.1 to 10 mg/kg administered Q2W, and no clinically meaningful differences in pharmacokinetics (PK) across ethnicities and tumor types were observed. Nivolumab clearance and volume of distribution were found to increase as body weight increases but less than proportionally with increasing weight, indicating that mg/kg dosing represents an over-adjustment for the effect of body weight on nivolumab PK.

Using the PPK and exposure-response models, nivolumab exposures and probabilities of efficacy responses and risks of AEs were predicted following nivolumab 480 mg Q4W and compared with those following nivolumab 3 mg/kg Q2W. The overall distributions of nivolumab C_{avgss} are comparable following administration with either nivolumab 3 mg/kg Q2W or nivolumab 480 mg Q4W. Nivolumab 480 mg Q4W is predicted to result in approximately 43% greater C_{maxss} compared with nivolumab 3 mg/kg Q2W; however, these exposures are predicted to be lower than the exposure ranges observed at doses up to nivolumab 10 mg/kg Q2W used in the nivolumab clinical program. Although the C_{maxss} of nivolumab is expected to be greater following nivolumab 480 mg Q4W compared with nivolumab 3 mg/kg Q2W, the predicted C_{maxss} following nivolumab 480 mg Q4W is predicted to be well below the median C_{maxss} achieved following administration of nivolumab 10 mg/kg Q2W, a safe and tolerable dose level.

Exposure-safety analysis demonstrated that the exposure margins for safety are maintained following nivolumab 480 mg Q4W, and the predicted risks of AE resulting in discontinuation of study drug, AE ≥ Grade 3, and immune-mediated AE ≥ Grade 2, are predicted to be similar following nivolumab 480 mg Q4W relative to nivolumab 3 mg/kg Q2W across tumor types. Safety analyses using available data following nivolumab 3 mg/kg Q2W and 10 mg/kg Q2W administration indicated there were no differences in AE profiles across body weight groups. Finally, initial evidence demonstrated that, following administration of nivolumab 480 mg Q4W, nivolumab has been shown to be well tolerated.

At nivolumab 480 mg Q4W, C_{minss} is predicted to be approximately 16% lower compared with nivolumab 3 mg/kg Q2W. While these exposures are predicted to be lower, they are predicted to be on the flat part of the exposure-response curves and are not predicted to affect efficacy. Exposure-efficacy analyses of multiple PK measures and efficacy endpoints (eg, OS, objective response) have indicated that, following administration of nivolumab 480 mg Q4W, efficacy is predicted to be similar to that following administration of nivolumab 3 mg/kg Q2W across

multiple tumor types. Based on these data, nivolumab 480 mg Q4W is expected to have similar efficacy and safety profiles to nivolumab 240 mg Q2W or nivolumab 3 mg/kg Q2W.

The less frequent dosing regimens (Q3W and Q4W vs Q2W) are designed to afford greater convenience to the target patient population and to allow combination of nivolumab with other agents. Refer to the nivolumab IB for details (Nivolumab IB, 2020).

4.3.2 Rationale for Nivolumab and Ipilimumab Combination Therapy Dose and Schedule (Advanced Metastatic Cancer)

For nivolumab and ipilimumab combination therapy in advanced metastatic cancer, nivolumab will be administered at 360 mg IV Q3W, and ipilimumab will be administered at 1 mg/kg IV Q3W for the first 2 doses and then every 6 weeks for the 3rd and 4th doses, followed by nivolumab 480 mg IV Q4W until PD or intolerable toxicity. The rationale for the nivolumab 360 mg Q3W and 480 mg Q4W dosing regimens is provided above (Section 4.3.1).

4.3.2.1 Rationale for Nivolumab and Ipilimumab Combination Therapy Dose and Schedule (Advanced Prostate Cancer Cohorts A and B)

For nivolumab and ipilimumab combination therapy in advanced prostate cancer, there will be 2 treatment cohorts, both of which will follow 6-week cycles as opposed to 3-week cycles.

Prostate Cohort A:

- nivolumab will be administered at **1 mg/kg IV Q3W** (4 doses over 2 cycles)
- ipilimumab will be administered at **3 mg/kg IV Q6W** (2 doses over 2 cycles)
- following the first 2 cycles, nivolumab will be administered at **480 mg IV Q4W** until PD or intolerable toxicity

Prostate Cohort B:

- nivolumab will be administered at **1 mg/kg IV Q3W** (4 doses over 2 cycles)
- ipilimumab will be administered at **5 mg/kg IV Q6W** (2 doses over 2 cycles)
- following the first 2 cycles, nivolumab will be administered at **480 mg IV Q4W** until PD or intolerable toxicity

Given data suggesting a dose response in earlier studies of ipilimumab 0.3, 3 and 10 mg/kg (Wolchok et al., 2010), and building on data observed in the current study with lower doses of ipilimumab, it is proposed that increased CTLA-4 inhibition in combination with anti-PD-1 may result in greater influx of CD8 T cells into an otherwise immune-excluded APC microenvironment.

The safety profile of ipilimumab has been well established in thousands of patients, both as a monotherapy at doses up to 10 mg/kg every 3 weeks, and in combination with nivolumab at various doses and schedules. In the phase 1 study CA209004 (NCT01024231), ascending doses of nivolumab (0.3, 1, or 3 mg/kg) have been studied concomitantly with ascending doses of ipilimumab (1 or 3 mg/kg) in subjects with unresectable or metastatic melanoma (Callahan et al., 2018; Wolchok et al., 2013). The combination of nivolumab 1 mg/kg + ipilimumab 3 mg/kg was assessed as the maximum tolerated dose (MTD) and taken forward for further evaluation, and was subsequently approved for treatment of metastatic melanoma (Opdivo USPI, 2020). Nivolumab 3 mg/kg + ipilimumab 3 mg/kg was found to exceed the MTD, but combinations of nivolumab 1 mg/kg with higher doses of ipilimumab have not been tested to date.

The disinhibition of T cell function induced by combined checkpoint blockade can lead to a distinct constellation of inflammatory side effects, collectively termed immune-related adverse events (irAEs). Ipilimumab, when given in combination with PD(L)-1 therapy, can induce a subset of adverse events, predominantly gastrointestinal, cutaneous, and hepatic, which in some cases can be severe. As such, irAE treatment guidelines have been established and widely implemented, and in most cases are effective in controlling these events (see Appendix 8; Nivolumab IB, 2020; Nivolumab IB Addendum, 2020).

In order to further mitigate the frequency and severity of these irAEs, only two doses of ipilimumab will be administered, and the doses will be given less frequently, on a 6 week schedule.

4.4 TREATMENT BEYOND DISEASE PROGRESSION

Accumulating evidence indicates that a minority of patients treated with immunotherapy may derive clinical benefit from continued treatment despite initial evidence of disease progression (Wolchok et al, 2009). Therefore, participants receiving nivolumab with or without ipilimumab in this study will be permitted to continue study intervention beyond initial Investigator-assessed disease progression (according to Response Evaluation Criteria in Solid Tumors version 1.1 [RECIST v1.1]) if they meet the 2 criteria listed below:

- Investigator-assessed clinical benefit, and
- Participant is tolerating study intervention

The assessment of clinical benefit should take into account whether the participant is clinically deteriorating and unlikely to receive further benefit from continued treatment.

All decisions to continue treatment beyond initial progression must be discussed with the Sponsor and documented in the study records.

Participants should discontinue study intervention upon evidence of further progression, defined as an additional ≥ 10% increase in tumor burden from the time of initial progression (including all target lesions and new measurable lesions).

New lesions are considered measurable at the time of initial progression if the diameters are ≥ 10 mm (except for pathological lymph nodes, which must have a short axis of ≥ 15 mm). Any new lesion considered non-measurable at the time of initial progression may become measurable and therefore included in the tumor burden measurement if the diameter increases to ≥ 10 mm (except for pathological lymph nodes, which must have an increase in short axis to ≥ 15 mm).

For statistical analyses that include the Investigator-assessed progression date, participants who continue treatment beyond initial Investigator-assessed disease progression (according to RECIST v1.1) will be considered to have Investigator-assessed PD at the time of the initial progression event.

4.5 END OF STUDY DEFINITION

A participant is considered to have completed the study if he/she has completed all phases of the study, including the last visit or the last scheduled procedure required for statistical analysis or safety follow-up.

The end of this study is defined as the date when the last visit of the last participant occurs or the date at which the last data point required for statistical analysis or safety follow-up is received from the last participant, whichever occurs later.

The total length of the study, from screening of the first participant to the end of the study, is expected to be approximately 4 years.

5 STUDY POPULATION

Prospective requests for approval of protocol deviations to recruitment and enrollment criteria, also known as waivers or exemptions, are not allowed.

5.1 INCLUSION CRITERIA

Participants are eligible to be included in the study only if all the following criteria apply:

General Inclusion Criteria

1. Participant must be ≥ 18 years of age inclusive, at the time of signing the informed consent.
2. Male or female participants of child-producing potential must agree to use contraception (as detailed in Appendix 6) or avoidance of pregnancy measures during the study and for 7 and 5 months, respectively, after the last dose.
3. Females of childbearing potential must have a negative serum or urine pregnancy test.

Cancer-specific Inclusion Criteria

4. Histologically or cytologically confirmed cancer that is metastatic, unresectable, or recurrent. Under Amendment 2 only, enrollment will be limited to tumor types known to be responsive to immunotherapy, have high prevalence (> 20%) of CD8 > 15% tumors, and/or have been observed in the study to have tumors transition from CD8 low to CD8 high following initiation of immunotherapy.
    a. Participants with these tumor types will be eligible to participate regardless of screening CD8 results (either ≥ 15% or < 15%).
    b. Tumor types for investigation under Amendment 2:
        i. Melanoma, Gastric, Small cell lung cancer, NSCLC, HCC, Cutaneous squamous cell carcinoma, RCC, Cervical, Gastroesophageal junction cancer, Urothelial bladder cancer, Sarcoma (soft tissue only), Neuroendocrine, SCCHN, Ovarian, Gynecologic malignancies, Merkel Cell, Prostate, Microsatellite instability high Colorectal, Anal (squamous cell only)
5. Participants who have received prior immunotherapy, including prior anti-PD-1 or anti-PD-L1 therapies, will be allowed to participate in this study.
    a. Participants who received prior anti-PD-1 or anti-PD-L1 may participate only if their prior anti-PD-1 or anti-PD-L1 monotherapy or combination therapy were NOT the last treatment prior to participation on this study**.**
    b. Participants who had prior immunotherapies and experienced Grade 1-2 immune-related adverse event (irAE) must have documentation that their irAEs are ≤ Grade 1 or baseline using current Common Terminology Criteria for Adverse Events v5.0 (CTCAE v5.0) **and** participants must be off steroid therapy and/or other immunosuppressive therapy, as treatment for irAEs, for ≥ 14 days from Cycle 1, Day 1.
    c. Participants who experienced Grade 3 irAEs consisting of laboratory abnormalities that were asymptomatic and have now resolved to ≤ Grade 1 or baseline **and** participants who have been off steroid and/or other immunosuppressive therapy, as treatment for irAEs, for ≥ 30 days from Cycle 1, Day 1.
6. Concurrent malignancies are permitted if any one of the following applies:
    a. Previously treated malignancy for which all treatment of that malignancy was completed at least 2 years before enrollment and no evidence of disease exists, or
    b. With agreement from the Sponsor and Principal Investigator (PI), participants who have a concurrent malignancy that is clinically stable and does not require tumor-directed treatment are eligible to participate if the risk of the prior malignancy interfering with either safety or efficacy endpoints is very low, or
    c. With agreement from the Sponsor and PI, other malignancies may be permitted if the risk of the prior malignancy interfering with either safety or efficacy end points is very low.

7. Provide newly obtained core needle or incisional biopsy of a non-bone tumor lesion not previously irradiated. Fine needle aspiration is not acceptable.
    a. Biopsies should be obtained from sites that do not pose significant risk to the participant based on the tumor site and the procedure used. Biopsy sites/procedures including, but not limited to, the brain, open lung/mediastinum, pancreas, or endoscopic procedures extending beyond the esophagus, stomach, or bowel would be considered to pose a significant risk to the participant. Procedures to areas that are deemed by the Investigator to be of non-significant risk based on individual clinical scenarios will be permitted.
8. Measurable disease as defined by RECIST v1.1.
    a. With the exception of prostate cancer participants enrolled under Amendment 3 who must have measurable disease, participants who do not have measurable disease by RECIST criteria but whose disease can be objectively measured through tumor markers or another disease-specific standard are considered eligible.
9. Eastern Cooperative Oncology Group (ECOG) performance status of 0 or 1.
10. Aspartate aminotransferase (AST) and alanine aminotransferase (ALT) ≤ 3 x upper limit of normal (ULN).
    a. Participants with hepatocellular carcinoma (HCC) may be eligible provided they have AST and ALT that are ≤ 5.0 x ULN.
11. Hemoglobin ≥ 9 g/dL.
12. Total bilirubin ≤ 1.5 × ULN.
    a. Participants with HCC are eligible provided they have total bilirubin < 3.0 x ULN and are considered Child-Pugh Class A or Child-Pugh Class B7 (Child-Pugh Class B with a total Child-Pugh score not to exceed 7).
    b. Participants with Gilbert syndrome must have ≤ 3 x ULN.
13. Creatinine clearance should be ≥ 30 mL/min as estimated by the Cockcroft-Gault equation or other appropriate calculation.
14. Absolute neutrophil count ≥ 1.0 x 10^9/L.
15. Platelets count ≥ 75 x 10^9/L.

Informed Consent

16. Participants must be capable of giving signed informed consent as described in Appendix 1, which includes compliance with the requirements and restrictions listed in the informed consent form (ICF) and in this protocol.

Advanced Prostate Cancer Specific Inclusion Criteria (Amendment 3 only)

17. Evidence of stage IV prostate cancer (as defined by American Joint Committee of Cancer criteria) on previous bone, CT and/or MRI scan.

18. Ongoing androgen deprivation therapy (ADT) with a gonadotropin-releasing hormone (GnRH) analogue or a surgical/medical castration with testosterone level of ≤ 1.73 nmol/L (< 50 ng/dL).
19. Participants with skeletal system symptoms who are already on medications (eg, bisphosphonates and/or RANK ligand inhibitors) to strengthen bones are allowed if they were started > 28 days before the first dose of study treatment.
20. Participants must have measurable disease per RECIST v 1.1.
21. Have received and progressed on prior secondary androgen receptor signaling inhibitor therapy (eg, abiraterone, enzalutamide, apalutamide). Progression is defined by one or more of the following 3 criteria:
    a. Prostate-specific antigen (PSA) ≥ 1.0 ng/mL and rising PSA by at least 2 consecutive measurements a minimum of 1-week apart.
    b. Soft tissue progression as defined RECIST v 1.1 (Eisenhauer et al., 2009).
    c. Bone disease progression as defined by 2 new bone lesions (as per Prostate Cancer Clinical Trials Working Group 3 [PCWG3; Scher et al., 2016]).

5.2 EXCLUSION CRITERIA

Participants are excluded from the study if any of the following criteria apply:

General Exclusion Criteria

1. Participants who had a medical condition that required a surgical procedure and were subject to general anesthesia within 4 weeks prior to beginning protocol therapy are excluded except the use of general anesthesia during biopsy procedures, indicated for patient comfort and or safety will be permitted.
2. Pregnant or breastfeeding.
3. Significant gastrointestinal disorder(s) (eg, active Crohn’s disease or ulcerative colitis or a history of extensive gastric resection and/or small intestinal resection).
4. Has interstitial lung disease or active, noninfectious pneumonitis.
5. Has a transplanted organ or has undergone allogeneic bone marrow transplant.
6. Has received a live vaccine within 30 days prior to first dose.
7. Known hypersensitivity to a component of protocol therapy.
    a. Participants with known hypersensitivity to ipilimumab and/or nivolumab are excluded.
8. Uncontrolled concurrent illness including, but not limited to, ongoing or active infection, clinically significant non-healing or healing wounds, symptomatic congestive heart failure, unstable angina pectoris, cardiac arrhythmia, significant pulmonary disease (shortness of breath at rest or on mild exertion), uncontrolled infection, or psychiatric

illness/social situations that, in the opinion of the investigator, would pose a safety concern or limit compliance with study requirements.

Cancer-specific Exclusion Criteria

9. Participants who experienced any ≥ Grade 3 symptomatic irAE on a prior immunotherapy study will be excluded from this study regardless of resolution of the irAE.
10. Any known, untreated, brain metastases. Treated participants must be stable 4 weeks after completion of treatment for brain metastases, and image-documented stability is required. Participants must have no clinical symptoms from brain metastases and have not required systemic corticosteroids > 10 mg/day prednisone or equivalent for ≥ 2 weeks prior to first dose of study intervention.
11. Participants with an active, known or suspected autoimmune disease. Participants with type I diabetes mellitus, hypothyroidism only requiring hormone replacement, skin disorders (such as vitiligo, psoriasis, or alopecia) not requiring systemic treatment, or conditions not expected to recur in the absence of an external trigger are permitted to enroll.
12. Participants with a condition requiring systemic treatment with either corticosteroids (> 10 mg daily prednisone equivalent) or other immunosuppressive medications within 14 days of start of study treatment. Inhaled or topical steroids, and adrenal replacement steroid doses > 10 mg daily prednisone equivalent, are permitted in the absence of active autoimmune disease.

Treatment-specific Exclusion Criteria

13. Anticancer chemotherapy, radiotherapy, immunotherapy, or investigational agents within 14 days of first dose of study intervention, provided that all treatment-related AEs have resolved.
    a. Recent or concurrent non-therapeutic investigational agents that are not anticipated to interfere with study intervention, may be permitted with written agreement from the Medical Monitor. As an exception to this requirement, administration of ^{89}Zr-Df-IAB22M2C in the context of a PICI-sponsored clinical trial does not require written agreement from the Medical Monitor.

5.3 LIFESTYLE CONSIDERATIONS

No restrictions are required.

5.4 SCREEN FAILURES

Screen failures are defined as participants who consent to participate in the clinical study but are not subsequently entered in the study. A minimal set of screen failure information is required to ensure transparent reporting of screen failure participants to meet the Consolidated Standards of Reporting Trials (CONSORT) publishing requirements and to respond to queries from regulatory

authorities. Minimal information includes demography, screen failure details, eligibility criteria, and any serious adverse event (SAE).

Individuals who do not meet the criteria for participation in this study (screen failure) may be rescreened.

6 STUDY INTERVENTION

Study intervention is defined as any investigational intervention(s), marketed product(s), or placebo intended to be administered to a study participant according to the study protocol.

6.1 STUDY INTERVENTION(S) ADMINISTERED

The study interventions to be administered in this study are summarized in Table 6.

Table 6: Study Intervention

Study Intervention Name	Dosage Formulation	Unit Dose Strength(s)	Dosage Level(s)	Route of Administration	Sourcing
**Advanced Metastatic Cancer**					
Nivolumab	Aqueous solution	10 mg/mL	360 mg (administered with ipilimumab)<br>480 mg (maintenance)	Intravenous	Bristol-Myers Squibb
Ipilimumab	Aqueous solution	5 mg/mL	1 mg/kg (administered with nivolumab)	Intravenous	Bristol-Myers Squibb
**Advanced Prostate Cancer**					
Nivolumab	Aqueous solution	10 mg/mL	1 mg/kg (administered with ipilimumab)<br>480 mg (maintenance)	Intravenous	Bristol-Myers Squibb
Ipilimumab	Aqueous solution	5 mg/mL	3 or 5 mg/kg (administered with nivolumab)	Intravenous	Bristol-Myers Squibb

Participants with ≥ 15% CD8 cells in their tumor biopsy enrolled on the CD8 high arm will be treated with single-agent nivolumab until disease progression. Additionally, participants enrolled on the CD8 high arm in this study could, at disease progression, cross-over to the CD8 low arm; participants with advanced prostate cancer enrolled under Amendment 3 who cross-over to the CD8 low arm will be placed in Prostate Cohort A.

Nivolumab monotherapy and nivolumab in combination with ipilimumab will be administered as described below.

- ***Nivolumab monotherapy (Advanced Metastatic Cancer and Advanced Prostate Cancer):*** Nivolumab will be administered at 360 mg as an IV infusion over approximately 30 minutes Q3W ± 3 days (see Section 4.3.1). Participants who

continue to show clinical benefit after the first disease assessment will receive nivolumab maintenance 480 mg IV Q4W ± 3 days until PD or intolerable toxicity (starting at Cycle 5).

- ***Nivolumab in combination with ipilimumab (Advanced Metastatic Cancer):*** Nivolumab will be administered at 360 mg as an IV infusion over approximately 30 minutes Q3W ± 3 days (see Section 4.3.2), followed approximately 30 minutes later by ipilimumab, which will be administered at 1 mg/kg as an IV infusion over approximately 30 minutes (Momtaz et al, 2015). Ipilimumab will be administered Q3W ± 3 days for the first 2 doses and then every 6 weeks for the 3rd and 4th doses, followed by nivolumab maintenance 480 mg IV Q4W ± 3 days until PD or intolerable toxicity (starting at Cycle 7). Each of the subsequent doses will be administered as nivolumab maintenance 480 mg IV Q4W ± 3 days over approximately 30 minutes.
- ***Nivolumab in combination with ipilimumab (Advanced Prostate Cancer):*** Participants in the < 15% CD8 cells (CD8 low) arm will receive either one of the following combinations of nivolumab and ipilimumab:
    - **Prostate Cohort A**: Nivolumab will be administered at **1 mg/kg** as an IV infusion over approximately 30 minutes **Q3W** (see Section 4.3.2), followed approximately 30 minutes later by ipilimumab, which will be administered at **3 mg/kg** as an IV infusion over approximately 30 minutes (Momtaz et al, 2015) **Q6W for two 6 week cycles**. After completion of 2 cycles, nivolumab maintenance **480 mg IV Q4W** ± 3 days will be administered until PD or intolerable toxicity.
    - **Prostate Cohort B**: Nivolumab will be administered at **1 mg/kg** as an IV infusion over approximately 30 minutes **Q3W** (see Section 4.3.2), followed approximately 30 minutes later by ipilimumab, which will be administered at **5 mg/kg** as an IV infusion over approximately 30 minutes (Momtaz et al, 2015) **Q6W for two 6 week cycles**. After completion of 2 cycles, nivolumab maintenance **480 mg IV Q4W** ± 3 days will be administered until PD or intolerable toxicity.

For all study interventions, either a peripheral IV or central port or line is acceptable for infusion. Administration of study intervention will be performed in a monitored setting in which there is immediate access to trained personnel and adequate equipment and medicine to manage potentially serious reactions.

6.2 PREPARATION/HANDLING/STORAGE/ACCOUNTABILITY

Participant weight will be assessed at Screening and Day 1 of each cycle. Dose preparations are based on the participant's weight and should be adjusted if the participant has a 10% change in comparison to their initial weight on Cycle 1, Day 1.

The Investigator or designee must confirm that appropriate temperature conditions have been maintained during transit for all study intervention received and any discrepancies are reported and resolved before use of the study intervention.

Only participants enrolled in the study may receive study intervention, and only authorized site staff may supply or administer study intervention. All study intervention must be stored in a secure, environmentally controlled, and monitored (manual or automated) area in accordance with the labeled storage conditions with access limited to the Investigator and authorized site staff.

The Investigator, institution, or the head of the medical institution (where applicable) is responsible for study intervention accountability, reconciliation, and record maintenance (ie, receipt, reconciliation, and final disposition records).

Further guidance and information for the final disposition of unused study interventions are provided in the Pharmacy Manuals.

6.3 MEASURES TO MINIMIZE BIAS: RANDOMIZATION AND BLINDING

6.3.1 Intervention Assignment

The advanced metastatic cancer portion of this study is not randomized. Treatment assignment will be based on the percentage of tumoral CD8 cells at the initial biopsy. Participants who have a tumor with ≥ 15% CD8 cells will receive nivolumab monotherapy, and participants who have a tumor with < 15% CD8 cells will receive ipilimumab in combination with nivolumab.

Approximately 20 advanced prostate cancer participants who have a tumor with < 15% CD8 cells will be randomly allocated to receive nivolumab 1 mg/kg in combination with ipilimumab 3 mg/kg (Prostate Cohort A) or nivolumab 1 mg/kg in combination with ipilimumab 5 mg/kg (Prostate Cohort B) in a 1:1 ratio. All advanced prostate cancer participants who have a tumor with ≥ 15% CD8 cells will receive nivolumab monotherapy. Participants who are randomly allocated but do not receive study intervention for any reason may be replaced. Randomization will be performed by the Sponsor.

Study intervention will be administered by site personnel and tracked using drug accountability records.

6.3.2 Blinding

This is an open-label trial; therefore, the Sponsor, Investigator, and participant will know the study intervention administered.

6.4 STUDY INTERVENTION COMPLIANCE

Study intervention will be administered by authorized site personnel and tracked using drug accountability records. No additional measures of compliance will be instituted.

6.5 CONCOMITANT THERAPY

Any medication or vaccine (including over-the-counter or prescription medicines, vitamins, and/or herbal supplements) that the participant is receiving at the time of enrollment or receives during the study must be recorded along with:

- Reason for use
- Dates of administration including start and end dates
- Dosage information including dose, administration route, and frequency

The Medical Monitor should be contacted if there are any questions regarding concomitant or prior therapy.

6.5.1 Permitted Therapy

Concomitant medications or treatments (eg, acetaminophen/paracetamol, diphenhydramine) may be prescribed if considered necessary for adequate prophylactic or supportive care except for those medications identified as "excluded" in Section 5.2 and Section 6.5.2.

6.5.2 Prohibited Therapy

The medications listed below are prohibited during the study. The Sponsor must be notified if a participant receives any of these during the study.

- Any concurrent investigational anticancer therapy.
- Immunosuppressive medications, including chronic systemic steroids at physiologic doses (equivalent to a dose of 10 mg oral prednisone) 14 days before the first dose (except for participants who require hormone replacement therapy [HRT]). A temporary course of steroids (eg, contrast allergy, chronic obstructive pulmonary disease) may be permitted, depending on the duration and dose, after discussion and agreement with the Medical Monitor.
- Any concurrent chemotherapy, radiotherapy (except palliative radiotherapy), immunotherapy, or biologic treatment. Concurrent use of hormones for noncancer-related conditions is permitted. Participants receiving cancer related hormonal therapy at study entry may continue with Medical Monitor approval. Ongoing androgen deprivation therapy and bone strengthening therapies (such as bisphosphonates and RANKL inhibitors) are permissible under the conditions in the advanced prostate cancer specific inclusion criteria.

- Any live attenuated vaccine therapies used for the prevention of infectious diseases (for up to 30 days prior to the first dose and 100 days after the last dose of study intervention).
- Any botanical preparation (eg herbal supplements or traditional Chinese medicines) intended to treat the disease under study. Use of marijuana and its derivatives for treatment of symptoms related to cancer or to cancer treatment are permitted.

6.6 DOSE MODIFICATIONS (ESCALATION/TITRATION/ OTHER)

Immuno-oncology agents are associated with AEs that can differ in severity and duration from AEs caused by other therapeutic classes. Early recognition and management of AEs associated with immuno-oncology agents may mitigate severe toxicity. Management algorithms (see Appendix 8) have been developed to assist Investigators in assessing and managing the following groups of drug-related AEs:

Management algorithms for AEs related to nivolumab and/or ipilimumab:

- Immune-mediated colitis
- Immune-mediated hepatitis
- Immune-mediated endocrinopathies (hypophysitis, adrenal insufficiency, hypothyroidism and hyperthyroidism, Type 1 diabetes mellitus)
- Immune-mediated skin adverse reactions
- Immune-mediated encephalitis
- Immune-mediated pneumonitis
- Immune-mediated nephritis and renal dysfunction
- Immune-mediated myocarditis

Based on the available characterization of the mechanism of action, and preliminary data from ongoing studies, nivolumab with or without ipilimumab may cause AEs similar to, but independent of, concurrent therapy, may exacerbate the frequency or severity, or may have non-overlapping toxicities. The anticipated important safety risks are outlined below. Refer to the Investigator's Brochure of nivolumab and the Investigator's Brochure of ipilimumab for complete summaries of safety information.

6.6.1 Dose Modifications with Nivolumab and Ipilimumab

The Investigator may attribute each AE to the combination of nivolumab and ipilimumab or to nivolumab alone. Study participants may not have any dose modifications of nivolumab or

ipilimumab doses in this study. After administration of the first dose ipilimumab, the Investigator may determine (based on clinical symptoms) the number of future doses of ipilimumab the participant will receive, for a maximum of 4 doses (advanced metastatic cancer) or for a maximum of 2 doses (advanced prostate cancer). If the ipilimumab is stopped and/or course has been completed, a repeat dose of ipilimumab at a later date is not permitted. Participants who stop ipilimumab dosing early due to toxicities may start nivolumab maintenance. If toxicity does not resolve or the criteria for resuming study intervention are not met, the participant must be discontinued from nivolumab monotherapy or nivolumab and ipilimumab combination therapy. Appropriate documentation is required regarding the drug to which the Investigator is attributing the AE. If, in the opinion of the Investigator, the toxicity is related to the combination of 2 agents, then both drugs should be held.

Specific anticipated or potential toxicities associated with the administration of nivolumab with or without ipilimumab, as well as the measures taken intended to avoid or minimize such toxicity in this trial, are described in the following sections.

6.6.1.1 Dose Modifications and Toxicity Management for Adverse Events Associated with Nivolumab with or without Ipilimumab

AEs associated with nivolumab with or without ipilimumab exposure may represent an immunologic etiology. These irAEs may occur shortly after the first dose or several months after the last dose of treatment and may affect more than one body system simultaneously. Therefore, early recognition and initiation of treatment is critical to reduce complications. Based on existing clinical study data, drug-related AEs have included pulmonary toxicity, renal toxicity (including acute renal failure), endocrine abnormalities, gastrointestinal toxicity, dermatologic toxicity (including rash), and hepatotoxicity. For nivolumab monotherapy and combination therapy, the majority of these AEs have been managed successfully with supportive care and, in more severe cases, a combination of dose delay, permanent discontinuation, and/or use of corticosteroids or HRT (endocrinopathies). For suspected irAEs, ensure adequate evaluation to confirm etiology or exclude other causes. Additional procedures or tests such as bronchoscopy, endoscopy, and skin biopsy may be included as part of the evaluation.

For Amendments 1 and 2, dose modification and toxicity management guidelines for irAEs associated with nivolumab with or without ipilimumab are provided in Table 7. For Amendment 3 (advanced prostate cohorts), requirements for the discontinuation of study treatment for irAEs associated with nivolumab with or without ipilimumab are provided in Table 9.

Table 7: Dose Modification and Toxicity Management Guidelines for Adverse Events Associated with Nivolumab with or without Ipilimumab

Drug-Related Adverse Event (AE) per CTCAE V5	Severity	Action Taken	Clarifications, Exceptions, and Resume Criteria
**Gastrointestinal**			
Colitis or Diarrhea	Grade 2	Delay dose	Dosing may resume when AE resolves to baseline
	Grade 3	Nivolumab monotherapy: Delay dose	Dosing may resume when AE resolves to baseline
		When administered with ipilimumab: Permanently Discontinue Ipilimumab	Nivolumab monotherapy may be resumed when AE resolves to baseline. If Grade 3 diarrhea or colitis recurs while on nivolumab monotherapy, permanently discontinue
	Grade 4	Permanently discontinue	
**Renal**			
Serum Creatinine Increased	Grade 2 or 3	Delay dose	Dosing may resume when AE resolves to Grade ≤ 1 or baseline value
	Grade 4	Permanently discontinue	
**Pulmonary**			
Pneumonitis	Grade 2	Delay dose	Dosing may resume after pneumonitis has resolved to ≤ Grade 1.
	Grade 3 or 4	Permanently discontinue	

Drug-Related Adverse Event (AE) per CTCAE V5	Severity	Action Taken	Clarifications, Exceptions, and Resume Criteria
**Hepatic**			
Aspartate aminotransferase (AST), alanine aminotransferase (ALT), or total bilirubin (T.bili) increased	AST or ALT > 3x and ≤ 5 x upper limit of normal (ULN) or T.Bili >1.5x and ≤ 3 x ULN, regardless of baseline value	Delay dose	Dosing may resume when laboratory values return to baseline.
	AST or ALT > 5 x ULN or T. bili > 3 x ULN, regardless of baseline value	Delay dose or Permanently discontinue	In most cases of AST or ALT > 5 x ULN, study treatment will be permanently discontinued. If the investigator determines a possible favorable benefit/risk ratio that warrants continuation of study treatment, a discussion between the investigator and the Medical Monitor/ designee must occur and a written agreement from Medical Monitor prior to resuming therapy.
	Concurrent AST or ALT > 3 x ULN and T.bili > 2 x ULN, regardless of baseline value	Permanently discontinue	
**Endocrinopathy**			
Adrenal Insufficiency	Grade 2 adrenal insufficiency	Delay dose	Dosing may resume with lab confirmation of hormone replacement treatment.
	Grade 3 or 4 adrenal insufficiency or adrenal crisis	Delay dose or permanently discontinue	Mandatory discussion with and approval from the Medical Monitor needed prior to resuming therapy. If adrenal insufficiency resolves or is adequately controlled with physiologic hormone replacement, participant may not require discontinuation of study drug.
Hyperglycemia	Hyperglycemia requiring initiation or change in daily management (Grade 2 or 3)	Delay dose	Dosing may resume if hyperglycemia resolves to Grade ≤ 1 or baseline value, or is adequately controlled with glucose-controlling agents.
	Grade 4	Delay dose or permanently discontinue	Mandatory discussion with and approval from the Medical Monitor needed prior to resuming therapy. If hyperglycemia resolves, or is adequately controlled with glucose-controlling agents, participant may not require discontinuation of study drug.

Drug-Related Adverse Event (AE) per CTCAE V5	Severity	Action Taken	Clarifications, Exceptions, and Resume Criteria
Hypophysitis/Hypopituitarism	Symptomatic Grade 1-3 that is also associated with corresponding abnormal lab and/or pituitary scan	Delay dose	Dosing may resume if endocrinopathy resolves to be asymptomatic, or is adequately controlled with only physiologic hormone replacement.
	Grade 4	Delay dose or permanently discontinue	Mandatory discussion with and approval from the Medical Monitor needed prior to resuming therapy. If endocrinopathy resolves or is adequately controlled with physiologic hormone replacement, participant may not require discontinuation of study drug.
Hyperthyroidism or Hypothyroidism	Grade 2 or 3	Delay dose	Dosing may resume if endocrinopathy resolves to be asymptomatic, or is adequately controlled with only physiologic hormone replacement or other medical management.
	Grade 4	Delay dose or permanently discontinue	Mandatory discussion with and approval from the Medical Monitor needed prior to resuming therapy. If endocrinopathy resolves or is adequately controlled with physiologic hormone replacement or other medical management, participant may not require discontinuation of study drug.
**Skin**			
Rash	Grade 2 rash covering >30% body surface area or Grade 3 rash	Delay dose	Dosing may resume when rash reduces to ≤ 10% body surface area
	Suspected Stevens-Johnson syndrome (SJS), toxic epidermal necrolysis (TEN) or drug reaction with eosinophilia and systemic symptoms (DRESS)	Delay dose	Dosing may resume if SJS, TEN, or DRESS is ruled out and rash reduces to is ≤ 10% body surface area
**Neurological**			
Guillain-Barre Syndrome (GBS)	Any Grade	Permanently discontinue	
Myasthenia Gravis (MG)	Any Grade	Permanently discontinue	

Drug-Related Adverse Event (AE) per CTCAE V5	Severity	Action Taken	Clarifications, Exceptions, and Resume Criteria
Encephalitis	Any Grade encephalitis	Delay dose	Differential Diagnosis workup is mandatory to rule out secondary causes (infection, tumor, etc). Dosing may resume with Medical Monitor discussion and written agreement.
	Any Grade drug-related encephalitis	Permanently discontinue	
Myelitis	Any Grade myelitis	Delay dose	After workup for differential diagnosis, (i.e. infection, tumor-related), if myelitis is not drug related, then dosing may resume when AE resolves
	Any Grade drug-related myelitis	Permanently discontinue	
Neurological (other than GBS, MG, encephalitis, or myelitis)	Grade 2	Delay dose	Dosing may resume when AE resolves to baseline
	Grade 3 or 4	Permanently discontinue	
**Myocarditis**			
Myocarditis	Symptoms induced from mild to moderate activity or exertion	Delay dose	Dosing may resume after myocarditis has resolved
	Severe or life threatening, with symptoms at rest or with minimal activity or exertion, and/or where intervention indicated.	Permanently discontinue	
**Other Clinical AE**			
Pancreatitis: Amylase or Lipase increased	Grade 3 with symptoms	Delay dose	Note: Grade 3 increased amylase or lipase without signs or symptoms of pancreatitis does not require dose delay. Dosing may resume when patient becomes asymptomatic.
	Grade 4	Permanently discontinue	
Uveitis	Grade 2 uveitis	Delay dose	Dosing may resume if uveitis responds to topical therapy (eye drops) and after uveitis resolves to Grade ≤ 1 or baseline. If patient requires oral steroids for uveitis, then permanently discontinue study drug.
	Grade 3 or 4 uveitis	Permanently discontinue	

Drug-Related Adverse Event (AE) per CTCAE V5	Severity	Action Taken	Clarifications, Exceptions, and Resume Criteria
Other Drug-Related AE **(not listed above)**	Grade 2 non-skin AE, except fatigue	Delay dose	Dosing may resume when AE resolves to Grade ≤ 1 or baseline value.
	Grade 3 AE - First occurrence lasting ≤ 7 days	Delay dose	Dosing may resume when AE resolves to Grade ≤ 1 or baseline value.
	Grade 3 AE- First occurrence lasting > 7 days	Permanently discontinue	
	Recurrence of Grade 3 AE of any duration	Permanently discontinue	
	Grade 4 or Life-threatening adverse reaction	Permanently discontinue	
**Other Lab abnormalities**			
Other Drug-Related lab abnormality **(not listed above)**	Grade 3	Delay dose	Exceptions:<br>No delay required for: Grade 3 lymphopenia<br><br>Permanent Discontinuation for: Grade 3 thrombocytopenia > 7 days or associated with bleeding.
	Grade 4	Permanently discontinue	Exceptions: The following events do not require discontinuation of study drug:<br>• Grade 4 neutropenia ≤ 7 days<br>• Grade 4 lymphopenia or leukopenia<br>• Grade 4 isolated electrolyte imbalances/abnormalities that are not associated with clinical sequelae and are responding to supplementation/appropriate management within 72 hours of their onset
**Infusion Reactions (manifested by fever, chills, rigors, headache, rash, pruritus, arthralgia, hypotension, hypertension, bronchospasm, or other allergic-like reactions.)**			
Hypersensitivity reaction or infusion reaction	Grade 3 or 4	Permanently discontinue	Refer to Section 6.6.1.2 on Treatment of Related Infusion Reactions

ALT = alanine aminotransferase; AST = aspartate aminotransferase; DRESS = drug reaction with eosinophilia and systemic symptoms; GBS = Guillain-Barre Syndrome; HCC = hepatocellular carcinoma; MG = Myasthenia Gravis; SJS = Stevens-Johnson syndrome; T.bili = total bilirubin; TEN = toxic epidermal necrosis; ULN = upper limit of normal

[a] Toxicity was graded per National Cancer Institute Common Terminology Criteria for Adverse Events, version 5.0 (NCI CTCAE v5.0).

[b] Resume treatment when adverse reaction improves to Grade 0 or 1.

[c] Resume treatment when AST/ALT returns to baseline.

6.6.1.2 Dose Modifications and Toxicity Management for Infusion-related Reaction Associated with Nivolumab with or without Ipilimumab

Interrupt or slow the rate of infusion in participants with mild or moderate infusion reactions. Discontinue nivolumab monotherapy or nivolumab and ipilimumab combination therapy in participants with severe or life-threatening infusion reactions.

Dose modification and toxicity management guidelines for infusion-related reaction associated with or without ipilimumab are provided in Table 8.

Table 8: Dose Modification and Toxicity Management Guidelines for Infusion-related Reaction Associated with Nivolumab with or without Ipilimumab

Grade (NCI CTCAE v 5.0)	Treatment	Premedication at Subsequent Dosing
**Grade 1**<br>Mild reaction; infusion interruption not indicated; intervention not indicated	Remain at bedside and monitor participant until recovery from symptoms.	The following prophylactic premedications are recommended for future infusions:<br>diphenhydramine 50 mg (or equivalent) and/or acetaminophen/paracetamol 325 to 1000 mg at least 30 minutes before additional nivolumab and/or ipilimumab administrations
**Grade 2**<br>Requires therapy or infusion interruption but responds promptly to symptomatic treatment (eg, antihistamines, NSAIDs, narcotics, IV fluids); prophylactic medications indicated for ≤24 hours	**Stop Infusion**<br>Begin an IV infusion of normal saline, and treat the participant with diphenhydramine 50 mg IV (or equivalent) and/or acetaminophen/paracetamol 325 to 1000 mg; remain at bedside and monitor participant until resolution of symptoms. Corticosteroid and/or bronchodilator therapy may also be administered as appropriate. If the infusion is interrupted, then restart the infusion at 50% of the original infusion rate when symptoms resolve; if no further complications ensue after 30 minutes, the rate may be increased to 100% of the original infusion rate. Monitor participant closely. If symptoms recur, then no **further study** medication will be administered at that visit.	For future infusions, the following prophylactic premedications are recommended:<br>diphenhydramine 50 mg (or equivalent) and/or acetaminophen/paracetamol 325 to 1000 mg should be administered at least 30 minutes before nivolumab and/or ipilimumab infusions. If necessary, corticosteroids (up to 25 mg of hydrocortisone or equivalent) may be used.
**Grades 3 or 4**<br>Grade 3:<br>Prolonged (ie, not rapidly responsive to symptomatic medication and/or brief interruption of infusion); recurrence of symptoms following initial improvement; hospitalization indicated for other clinical sequelae (eg, renal impairment, pulmonary infiltrates)<br>Grade 4:<br>Life-threatening consequences; urgent intervention indicated	**Stop Infusion**<br>Begin an IV infusion of normal saline and treat the participant as follows: Recommend bronchodilators, epinephrine 0.2 to 1 mg of a 1:1000 solution for subcutaneous administration or 0.1 to 0.25 mg of a 1:10,000 solution injected slowly for IV administration, and/or diphenhydramine 50 mg IV with methylprednisolone 100 mg IV (or equivalent), as needed. Participant should be monitored until the Investigator is comfortable that the symptoms will not recur. Remain at bedside and monitor participant until recovery of the symptoms. Study drug will be permanently discontinued.<br>Investigators should follow their institutional guidelines for the treatment of anaphylaxis.	No subsequent dosing
In case of late-occurring hypersensitivity symptoms (eg, appearance of a localized or generalized pruritus within 1 week after treatment), symptomatic treatment may be given (eg, oral antihistamine or corticosteroids).		
Appropriate resuscitation equipment should be available at the bedside and a physician readily available during the period of drug administration.<br>For further information, please refer to the Common Terminology Criteria for Adverse Events v5.0 (CTCAE) at http://ctep.cancer.gov		

CTCAE = Common Terminology Criteria for Adverse Events; NCI = National Cancer Institute; NSAIDs = nonsteroidal anti-inflammatory drugs; IV = intravenous

6.6.1.3 Dose Delays and Interruptions for Nivolumab with or without Ipilimumab

Dose delays and interruptions are permitted for toxicity reasons (see Section 6.6.1.1). Dose delays and interruptions for reasons other than toxicity, such as surgical procedures, may be allowed with Medical Monitor approval. The acceptable length of interruption will depend on agreement between Investigator and Medical Monitor.

6.6.1.4 Additional Safety Precautions (Advanced Prostate Cancer Cohort Only)

The safety profile for nivolumab (1 mg/kg) in combination with ipilimumab (3 mg/kg) is well established in melanoma and HCC at more frequent ipilimumab administration (Q3W vs Q6W) than proposed for this cohort (Opdivo USPI, 2020). Hence, concurrent enrollment will be pursued with participants randomly allocated to regimens containing either ipilimumab 3 mg/kg or 5 mg/kg Q6W for up to 2 doses in combination with nivolumab 1 mg/kg. As the combination regimen with ipilimumab 5 mg/kg has not been extensively studied, in addition to adverse events being reviewed by the Sponsor on an ongoing basis and investigator meetings convening approximately every 2 weeks to discuss and review safety events, a safety pause is included.

Enrollment will pause until all participants have been followed for at least 6 weeks. During the pause, new participants may be consented and screened, but not initiated on study intervention. The safety pause will apply to both cohorts to preserve random allocation; however, the safety assessment as follows will apply to the ipilimumab 5 mg/kg cohort specifically. The initial 5 participants enrolled in the ipilimumab 5 mg/kg cohort will be observed for the occurrence of treatment-related adverse events (TRAEs) necessitating discontinuation of both agents (AEs requiring discontinuation are included in Table 9) from the beginning of treatment through completion of the first cycle (ie, 6 weeks).

If 3 or fewer of the first 5 participants in the ipilimumab 5 mg/kg cohort experience a TRAE leading to discontinuation of both agents, enrollment for that specific cohort will continue as planned until a total of approximately 10 participants are enrolled. If 4 or more of the first 5 participants of the ipilimumab 5 mg/kg cohort experience a TRAE leading to discontinuation of both agents, enrollment into the cohort will be suspended, and an ad hoc safety review (conducted by Investigators and Sponsor representatives) will be performed to assess the available data and provide a recommendation, which may include, but is not limited to, modification or discontinuation of the cohort, and will be documented in the study records. Only TRAEs leading to treatment discontinuation of both agents occurring up until the 5th participant in each cohort completes Cycle 1 will be included—dose delays for adverse events not otherwise meeting criteria at that time will not be counted. The stopping rule was chosen as this corresponds to a > 80% chance that the toxicity rate in the cohort is > 40%, assuming a beta (0.8, 1.2) prior.

Should the ipilimumab 5 mg/kg cohort demonstrate 6 or greater treatment-related adverse events requiring discontinuation of therapy at any time, enrollment will be suspended until an ad hoc safety review convenes and provides a recommendation, as described above.

Table 9: Toxicity Criteria Requiring Permanent Treatment Discontinuation of Both Nivolumab and Ipilimumab (Amendment 3 Advanced Prostate Cohort Only)

Participants should be monitored for the occurrence of any of the following AEs that are considered by the Investigator to be possibly, probably, or definitely related to ipilimumab, nivolumab, or the immunotherapy combination.

Treatment with all study intervention should be monitored for the occurrence of any of the following TRAEs, and if any of these events occur, action should be taken as follows:

- Any Grade 2 drug-related uveitis, eye pain or blurred vision that does not respond to topical therapy and does not improve to Grade 1 severity within the re-treatment period OR requires systemic treatment.
- Any Grade 3 non-skin, drug-related AE lasting > 7 days, or recurs with the following exceptions for laboratory abnormalities, diarrhea, colitis, neurologic toxicity, drug-related uveitis, pneumonitis, bronchospasm, hypersensitivity reactions, infusion reactions, and endocrinopathies:
  - Grade 3 drug-related diarrhea, colitis, neurologic toxicity, uveitis, pneumonitis, bronchospasm, myocarditis, hypersensitivity reaction, or infusion reaction of any duration requires discontinuation.
  - Grade 3 drug-related endocrinopathies, adequately controlled with only physiologic hormone replacement do not require discontinuation. Adrenal insufficiency requires discontinuation regardless of control with hormone replacement.
  - Grade 3 drug-related laboratory abnormalities do not require treatment discontinuation except:
    - Grade 3 drug-related thrombocytopenia > 7 days or associated with bleeding requires discontinuation.
    - Any drug-related liver function test abnormality that meets the following criteria require discontinuation:
      - Grade ≥ 3 drug-related AST, ALT or total bilirubin requires discontinuation.
        - In most cases of Grade 3 AST, ALT evaluation study drug(s) will be permanently discontinued. If the investigator determines a possible favorable benefit/risk ratio that warrants continuation of study drug(s), a discussion between the investigator and the Medical Monitor (or designee) must occur.
      - Concurrent AST or ALT > 3x ULN and total bilirubin > 2x ULN.
- Any Grade 4 drug-related adverse event or laboratory abnormality (including but not limited to creatinine, AST, ALT, or total bilirubin), except for the following events which do not require discontinuation:
  - Grade 4 neutropenia ≤ 7 days.
  - Grade 4 lymphopenia or leukopenia or asymptomatic amylase or lipase.
  - Isolated Grade 4 electrolyte imbalances/abnormalities that are not associated with clinical sequelae and are corrected with supplementation/appropriate management within 72 hours of their onset.
  - Grade 4 drug-related endocrinopathy adverse events, such as, hyper- or hypothyroidism, or glucose intolerance, which resolve or are adequately controlled with physiologic hormone replacement (corticosteroids, thyroid hormones) or glucose-controlling agents, respectively, may not require discontinuation after discussion with and approval from the Medical Monitor (or designee).
- Any event that leads to delay in dosing lasting > 12 weeks from the previous dose requires discontinuation, with the following exceptions:
  - Dosing delays to allow for prolonged steroid tapers to manage drug-related adverse events are allowed.
  - Dosing delays lasting > 6 weeks from the previous dose that occur for non-drug-related reasons may be allowed if approved by the Medical Monitor (or designee).

• Prior to re-initiating treatment in a participant with a dosing delay lasting > 6 weeks, the Medical Monitor (or designee) must be consulted. Tumor assessments should continue as per protocol even if dosing is delayed. Periodic study visits to assess safety and laboratory studies should also continue per SOA or more frequently if clinically indicated during such dosing delays.<br>• Any adverse event, laboratory abnormality, or intercurrent illness which, in the judgment of the Investigator, presents a substantial clinical risk to the participant with continued nivolumab dosing.

AE = adverse event; ALT = alanine aminotransferase; AST = aspartate aminotransferase; SOA = Schedule of Assessments; TRAEs = treatment-related adverse event; ULN = upper limit of normal

6.7 INTERVENTION AFTER THE END OF THE STUDY

There will be no intervention following the end of the study.

7 DISCONTINUATIONS OF STUDY INTERVENTION AND PARTICIPANT DISCONTINUATION/WITHDRAWAL

7.1 DISCONTINUATION OF STUDY INTERVENTION

Participants must discontinue study intervention if they experience any of the following:

- Intolerable toxicity related to study intervention, including the development of an AE determined by the Investigator to be unacceptable even with the participant's potential response to intervention due to the severity of the event
- Any medical condition that may jeopardize participant safety if he or she continues the study intervention
- Use of another anticancer therapy
- Symptomatic deterioration attributed to disease progression that per assessment by the Investigator necessitates discontinuation of study intervention.
- Radiographic disease progression per RECIST v1.1, unless the participant in the nivolumab monotherapy arm decides to enroll into the nivolumab and ipilimumab combination treatment arm (see Appendix 3). Refer to Section 4.4 for description of treatment beyond progression.
- Pregnancy (see Appendix 6)

Participants have the right to voluntarily withdraw from study intervention at any time for any reason. In addition, the Investigator has the right to withdraw a participant from study intervention at any time. Reasons for withdrawal from study intervention may include, but are not limited to, the following:

- Investigator or Sponsor determines it is in the best interest of the participant
- Participant noncompliance

The primary reason for study intervention discontinuation should be documented on the appropriate electronic case report form (eCRF) page.

The visit at which disease assessment shows PD may be used as the end of treatment visit. Participants who discontinue study intervention for any reason other than PD or loss of clinical benefit are to continue assessments as outlined in the Schedule of Activities (SOAs; see Section 1.3).

7.2 PARTICIPANT DISCONTINUATION/WITHDRAWAL FROM STUDY

When study intervention is discontinued, participants should have an end of treatment/discontinuation assessment and continue follow-up assessments until radiographic progression or the start of subsequent therapy, whichever comes first, as outlined in the SOAs (see Section 1.3). In addition, ad hoc scans, as part of standard of care, will be collected for participants who have discontinued treatment for reasons other than radiographic progression and prior to any alternate therapies. Information on survival follow-up and new anticancer therapy will be collected for all participants via telephone calls, participant medical records, and/or clinic visits, until any of the following occurs:

- Death
- Lost to follow-up
- Study termination by the Sponsor
- Participant requests to be withdrawn from follow-up
- Investigator requests that the participant is withdrawn from follow-up

If a participant requests to be withdrawn from the study, the request must be documented in the source documents and signed by the Investigator. The primary reason for withdrawal from study should be documented on the appropriate eCRF page. If the participant withdraws from study, the Sponsor may retain and continue to use any data collected before such withdrawal of consent. In addition, the study staff may use a public information source (eg, county records) to obtain information about survival status only. However, participants who withdraw consent will not be followed for any reason after consent has been withdrawn. Participants who receive study intervention and subsequently withdraw from the study will not be replaced.

7.3 LOST TO FOLLOW-UP

A participant will be considered lost to follow-up if he or she repeatedly fails to return for scheduled visits and is unable to be contacted by the study site.

The following actions must be taken if a participant fails to return to the clinic for a required study visit:

- The site must attempt to contact the participant and reschedule the missed visit as soon as possible and counsel the participant on the importance of maintaining the assigned visit schedule and ascertain whether or not the participant wishes to and/or should continue in the study.
- Before a participant is deemed lost to follow-up, the Investigator or designee will make every effort to regain contact with the participant (where possible, 3 telephone calls and, if necessary, a certified letter to the participant's last known mailing address or local equivalent methods). These contact attempts should be documented in the participant's medical record or study file.
- Should the participant continue to be unreachable, he/she will be considered to have withdrawn from the study.

8 STUDY ASSESSMENTS AND PROCEDURES

Please see Section 1.3 for the SOAs to be performed during the study. All activities must be performed and documented for each participant in the order of SOAs. Participants will be closely monitored for safety and tolerability throughout the study. Participants should be assessed for toxicity prior to each dose; dosing will occur only if the clinical assessment and local laboratory test values are acceptable as deemed by the PI.

If the timing of a protocol-mandated study visit coincides with a holiday, weekend, or other administrative disruption that precludes the visit, the visit should be scheduled on the nearest following feasible date. The time between doses must not be less than minus 3 days from the normal schedule of the last dose received (doses must not be administered < 25 days for a Q4W schedule or < 18 days apart for a Q3W).

Collection of any non-safety-related data or participant samples may be terminated by the Sponsor at any time if further collection of such data or samples is also not related to the study's primary or secondary objective. The decision to discontinue any data collection will be communicated to the sites and Institutional Review Board (IRB)/Independent Ethics Committee (IEC) by means of a memorandum and will not require a protocol amendment.

8.1 EFFICACY ASSESSMENTS

Participants will undergo tumor assessments as designated in the SOAs (see Section 1.3) regardless of dose delays, until loss of clinical benefit as determined by the Investigator (unless the participant withdraws consent or the Sponsor terminates the study). All participants who discontinue study intervention for reasons other than radiographic progression (eg, AEs) will

continue tumor assessments until death, disease progression, initiation of another systemic anticancer therapy, loss to follow-up, withdrawal of consent, or study termination, whichever occurs first. At the Investigator's discretion, tumor assessments may be repeated at any time if PD is suspected.

Measurable and evaluable lesions should be assessed and documented at screening. Tumor assessments performed as standard of care prior to obtaining informed consent and within 28 days prior to treatment do not have to be repeated at screening.

Screening assessments must include computed tomography (CT) scans (with IV contrast unless contraindicated and oral contrast as appropriate per institutional standards). A spiral CT scan of the chest may be obtained but is not a requirement. If a CT scan with contrast is contraindicated (ie, in participants with contrast allergy or impaired renal clearance), a non-contrast CT scan of the chest may be performed and magnetic resonance imaging (MRI) scans of the abdomen, pelvis, and head (as applicable) should be performed as applicable.

If a CT scan for a tumor assessment is performed in a positron emission tomography (PET)/CT scanner, the CT acquisition must be consistent with the standards for a full contrast diagnostic CT scan.

Bone scans (technetium-99m [TC-99m]) or sodium fluoride (NaF) PET should be performed at screening if clinically indicated. If bone metastases are present at screening and cannot be seen on CT or MRI scans, or if clinically indicated, TC-99m and NaF-PET bone scans should be repeated when complete response (CR) is identified in target disease or when progression in bone is suspected.

CT scans of the head, neck, or extremities should also be performed if clinically indicated and repeated throughout the study if there is evidence of disease at screening.

All measurable and evaluable lesions should be re-assessed at each subsequent tumor evaluation. The same radiographic procedures used to assess disease sites at screening should be used for subsequent tumor assessments (eg, same contrast protocol for CT scans).

Response will be assessed by the Investigator using RECIST v1.1 (see Appendix 3). Assessments should be performed by the same evaluator, if possible, to ensure internal consistency across visits. Results must be reviewed by the Investigator before dosing at the next cycle.

8.1.1 Laboratory Assessments of Tumor Markers

Results for tumor markers (eg, CA19-9, PSA, etc) will be collected if performed as part of standard of care.

PSA is required for participants in the prostate cohorts under Amendment 3 (see Table 3 and Table 4).

8.2 SAFETY ASSESSMENTS

Safety assessments will consist of monitoring and recording AEs, including SAEs, performing protocol-specified safety laboratory assessments, measuring protocol specify vital signs, and conducting other protocol-specified tests that are deemed critical to the safety evaluation of the study. Planned timing for all safety assessments is provided in the SOAs (Section 1.3).

Certain types of events require immediate reporting to the Sponsor, as described in Section 8.3.1.1.

8.2.1 Medical History and Demographic Data

Medical history, including clinically significant diseases, surgeries, cancer history (including stage, date of diagnoses, and prior cancer therapies and procedures), reproductive status, smoking history, use of alcohol, and drugs of abuse, will be recorded at baseline. In addition, all medications (eg, prescription drugs, over-the-counter drugs, vaccines, herbal or homeopathic remedies, nutritional supplements) used by the participant within 28 days prior to study intervention will be recorded. Demographic data may include age, sex, and race/ethnicity.

8.2.2 Physical Examinations

A complete physical examination should be performed at screening and should include an evaluation of the head, eyes, ears, nose, and throat, and the cardiovascular, dermatological, musculoskeletal, respiratory, gastrointestinal, genitourinary, and neurological systems. Any abnormality identified at baseline should be recorded on the Medical History eCRF page.

ECOG Performance Status (see Appendix 2) should be assessed per the SOAs in Section 1.3.

At subsequent visits (or as clinically indicated), limited, symptom-directed physical examinations should be performed. Changes from baseline abnormalities should be recorded in participant notes. New or worsened clinically significant abnormalities should be recorded as AEs on the Adverse Event eCRF page.

8.2.3 Vital Signs

Vital signs should include measurements of respiratory rate, pulse rate, systolic and diastolic blood pressure, pulse oximetry (only at baseline), and temperature as outlined in the SOA (Section 1.3). Vital signs collected at the screening visit should be recorded on the eCRF. For each visit thereafter, only vital signs obtained prior to the first drug infusion on Day 1 or during an AE (eg, temperature or event of fever) should be recorded on the eCRF. All vital signs collected per protocol should be documented in the participant’s medical record.

Vital signs should be measured on Day 1 of every cycle prior to administration of study intervention. Vital signs may be measured, if medically indicated, at other time points.

Vital signs should be measured within 0-20 minutes prior to the first infusion and, if clinically indicated, during or after the infusions (see Section 6).

8.2.4 Electrocardiograms

A single ECG recording will be obtained at Baseline, as outlined in the SOAs (Section 1.3), and may be obtained at unscheduled time points as indicated.

All ECG recordings must be performed using an institutionally approved ECG. Lead placement should be as consistent as possible. ECG recordings must be performed after the participant has been resting in a supine position for at least 10 minutes. All ECGs are to be obtained prior to other procedures scheduled at that same time (eg, vital sign measurements, blood draws) and should not be obtained within 3 hours after any meal. Circumstances that may induce changes in heart rate, including environmental distractions (eg, television, radio, conversation), should be avoided during the pre-ECG resting period and during ECG recording.

For safety monitoring purposes, the Investigator must review, sign, and date all ECG tracings. Paper copies of ECG tracings will be kept as part of the participant's permanent study file at the site. The following should be recorded on the appropriate eCRF page: heart rate, RR interval, QRS interval, PR duration, uncorrected QT interval, and QTcF based on the machine readings of the individual ECG tracings. Any morphologic waveform changes or other ECG abnormalities must be documented on the eCRF page.

8.2.5 Clinical Safety Laboratory Assessments

Clinical laboratory tests will be performed at the local institution as described in the SOAs (Section 1.3).

The Investigator must review the laboratory report, document this review, and record any clinically relevant changes occurring during the study on the appropriate eCRF. The laboratory reports must be filed with the source documents. Clinically significant abnormal laboratory findings are those that are not associated with the underlying disease, unless judged by the Investigator to be more severe than expected for the participant's condition.

In the event of a Grade 3 or Grade 4 laboratory toxicity, the test for the abnormal laboratory value should be repeated until the event is resolved to ≥ Grade 1 or baseline.

8.2.5.1 Local Laboratory Assessments

Samples for the laboratory test in Table 10 will be sent to the study site local laboratory for analysis:

Table 10: Laboratory Tests Sent to the Study Site's Local Laboratory for Analysis

Profile	Laboratory Test
Hematology	white blood cell count<br>hemoglobin<br>hematocrit<br>platelet count<br>differential count (neutrophils, lymphocytes)
Clinical Chemistry (Serum or Plasma)	sodium<br>potassium<br>magnesium<br>glucose<br>BUN or urea<br>creatinine<br>total bilirubin (including either indirect or direct)<br>alkaline phosphatase<br>ALT<br>AST<br>LDH<br>TSH, FT3, and FT4 - screening<br>TSH, with reflexive FT3 and FT4 if TSH is abnormal - on treatment<br>Lipase
Urinalysis	

ALT = alanine aminotransferase; AST = aspartate aminotransferase; BUN = blood urea nitrogen; FT3 = free triiodothyronine; FT4 = free thyroxine; LDH = lactate dehydrogenase; TSH = thyroid stimulating hormone

8.3 ADVERSE EVENTS AND SERIOUS ADVERSE EVENTS

The definitions of an AE or SAE can be found in Appendix 5.

Investigators will seek information on AE at each participant contact. AEs reported by the participant (or, when appropriate, by a caregiver, surrogate, or the participant's legally authorized representative) or noted by study personnel will be recorded in the participant's medical record and on the Adverse Event eCRF page.

The Investigator and any qualified designees are responsible for detecting, documenting, and recording events that meet the definition of an AE or SAE and remain responsible for following up AEs that are serious, considered related to the study intervention or study procedures, or that caused the participant to discontinue the study intervention (see Appendix 5).

8.3.1 Time Period and Frequency for Collecting AE, SAE, and Other Reportable Safety Event Information

All AEs and SAEs will be collected from the time the participant signs informed consent until 100 days after the last dose of study intervention.

Prior to initiation of study intervention, only AEs and SAEs that are related to a protocol-mandated intervention, including those that occur prior to assignment of study procedures (eg, screening invasive procedures, such as biopsies) should be reported. After obtaining informed consent, but prior to initiation of study intervention, other medical occurrences will be recorded as medical history.

If the Investigator learns of any SAE, including a death, at any time after the end of the AE reporting period, and he/she considers the event to be reasonably related to the study intervention or study participation, the Investigator must promptly notify the Sponsor or its designee. The Investigator should report these events directly to the Sponsor or its designee, either by faxing or emailing the Serious Adverse Event Report Form (SAE Report Form).

The method of recording and reporting of AE and SAE are provided in Appendix 5. The procedure for submitting SAE reports is provided in Section 8.3.8.2.

The method of reporting all deaths are provided in Appendix 5.

8.3.1.1 Events Requiring Expedited Reporting to the Sponsor

Certain events require immediate reporting to allow the Sponsor to take appropriate measures to address potential new risks in a clinical trial. The Investigator must report such events to the Sponsor immediately; under no circumstances should reporting take place more than 24 hours after the Investigator becomes aware of the event. The following is a list of events that the Investigator must report to the Sponsor on a SAE Report Form within 24 hours of becoming aware of the event, regardless of relationship to study intervention:

- All SAEs (defined in Appendix 5)
- Pregnancy (see Section 8.3.5 for details on reporting requirements)
- Occurrence of overdose (see Section 8.4 for details on reporting requirements)

8.3.2 Follow-up Event Reporting

The Investigator must report new significant follow-up information for these events to the Sponsor immediately (ie, no more than 24 hours after becoming aware of the information). New significant information includes the following:

- New signs or symptoms or a change in the diagnosis
- Significant new diagnostic test results
- Change in causality based on new information
- Change in the event’s outcome, including recovery
- Additional narrative information on the clinical course of the event

After the initial AE/SAE report, the Investigator is required to proactively follow each participant at subsequent visits/contacts. All AEs and SAEs will be followed through the follow up phase of the study. Events will be followed until event resolution or death, the participant is lost to follow-up (as defined in Section 7.3), or the participant withdraws consent. Further information on follow-up procedures is provided in Appendix 5.

For SAEs, the Sponsor or a designee may follow up by telephone, fax, electronic mail, and/or a monitoring visit to obtain additional case details and outcome information (eg, from hospital discharge summaries, consultant reports, autopsy reports) in order to perform an independent medical assessment of the reported case.

8.3.3 Method of Eliciting Adverse Event Information

A consistent methodology of non-directive questioning should be adopted for eliciting AE information at all participant evaluation time points. Open-ended and non-leading verbal questioning of the participant is the preferred method to inquire about AE occurrences. Examples of non-directive questions include: “How have you felt since your last clinic visit?”, “Have you had any new or changed health problems since you were last here?”

8.3.4 Regulatory Reporting Requirements for SAEs

Prompt notification by the Investigator to the Sponsor of an SAE is essential so that legal obligations and ethical responsibilities towards the safety of participants and the safety of a study intervention under clinical investigation are met (see Section 8.3.1.1). Investigators must also comply with local requirements for reporting SAEs to the IRB/IEC or other local health authorities.

The Sponsor has a legal responsibility to notify both the local regulatory authority and other regulatory agencies about the safety of a study intervention under clinical investigation. The Sponsor will comply with country-specific regulatory requirements relating to safety reporting to the regulatory authority, IRB/IEC, partner companies, and Investigators.

Expectedness will be assessed using the Investigator’s Brochure(s) as reference documents. Reporting requirements will also be based on the Investigator's assessment of causality and seriousness, with allowance for upgrading by the Sponsor as needed.

Investigator safety reports must be prepared for suspected unexpected serious adverse reactions (SUSAR) according to local regulatory requirements and Sponsor policy and forwarded to Investigators as necessary.

An Investigator who receives an Investigator safety report describing an SAE or other specific safety information (eg, summary or listing of SAEs) from the Sponsor will review and then file it

along with the Investigator's Brochure and will notify the IRB/IEC, if appropriate, according to local requirements.

8.3.5 Pregnancy

Details of all pregnancies in female participants and female partners of male participants will be collected after the start of study intervention and until 100 days after the last dose. Female participants, as well as female partners of male participants, of childbearing potential will be instructed to immediately inform the Investigator if they become pregnant during the study or within 100 days after the last dose of study intervention. If a pregnancy is reported, the Investigator should inform the Sponsor within 24 hours of becoming aware of the pregnancy and should follow the procedures outlined in Appendix 6. Abnormal pregnancy outcomes that meet serious criteria (eg, spontaneous abortion, fetal death, stillbirth, congenital anomalies, ectopic pregnancy) are considered SAEs.

A clinical trial specific Pregnancy Reporting form should be completed and submitted immediately to the Sponsor or its designee, either by faxing or by scanning and emailing the form using the fax number or email address provided to Investigators (see Section 8.3.8.2). Pregnancy should not be recorded on the Adverse Event eCRF page. The Investigator should discontinue study intervention and counsel the participant, discussing the risks of the pregnancy and the possible effects on the fetus. Monitoring of the participant should continue until conclusion of the pregnancy. Any SAEs associated with the pregnancy (eg, an event in the fetus, an event in the mother during or after the pregnancy, or a congenital anomaly/birth defect in the child) should be reported on the SAE Report Form. In addition, the Investigator will submit a clinical trial specific Pregnancy Reporting form when updated information on the course and outcome of the pregnancy becomes available.

8.3.6 Disease-related Events and/or Disease-related Outcomes Not Qualifying as AEs or SAEs

Progression of the cancer under study, as judged by the Investigator, is not considered a reportable event. Upon further review by the Investigator, if the event is not considered underlying progression of disease, it must be reported as an SAE within 24 hours or becoming aware of the event to the Parker Institute for Cancer Immunotherapy Pharmacovigilance Group as described in Section 8.3.8.2.

8.3.7 Trial-limiting Toxicity

Trial-limiting toxicity (TOX) is defined as the occurrence of any adverse event leading to treatment discontinuation that is possibly, probably, or definitely related to nivolumab, ipilimumab, or ipilimumab in combination with nivolumab during the initial 12 weeks of

treatment, unless there is a clear alternative explanation. Additional discontinuation criteria regarding the advanced prostate cohorts can be found in Section 6.6.1.4.

8.3.8 Sponsor Contact Information

8.3.8.1 Emergency Medical Contacts

Parker Institute for Cancer Immunotherapy Medical Monitor Contact Information:

Primary Medical Monitor: Marko Spasic, MD

Parker Institute for Cancer Immunotherapy
1 Letterman Drive, Ste. D3500
San Francisco, CA 94080
mspasic@parkerici.org

Telephone No.: (916) 303-0815 (Cell; United States)

Alternate Medical Monitor: Joyson Karakunnel, MD, MSc, FACP

Parker Institute for Cancer Immunotherapy
1 Letterman Drive, Ste. D3500
San Francisco, CA 94080
jkarakunnel@parkerici.org

Telephone No.: (415) 539-3165 (Office; United States)

8.3.8.2 Safety Reporting Contacts

The following contact information should be used when submitting safety-related paper forms (SAE Report Form and Pregnancy Report Form) as described in Appendix 5 for SAEs and other reportable safety events. These forms should be completed and submitted to the Sponsor immediately (ie, no more than 24 hours after becoming aware of the event), by faxing to the Parker Institute for Cancer Immunotherapy Pharmacovigilance Group at: 415-610-5471 within 24 hours of event awareness. If technical issues arise, the form may be scanned and emailed to safety@parkerici.org and please contact the Parker Institute for Cancer Immunotherapy Pharmacovigilance group immediately at 415-930-4414.

Parker Institute for Cancer Immunotherapy Pharmacovigilance Group:

- Pharmacovigilance Fax Number: 415-610-5471
- Pharmacovigilance email: safety@parkerici.org
- Pharmacovigilance Telephone Number: 415-930-4414

8.4 TREATMENT OF OVERDOSE

Overdose is defined as any dose higher than the dose specified to be administered in accordance with the protocol.

The Investigator must immediately notify the Parker Institute for Cancer Immunotherapy Pharmacovigilance Group of any occurrence of overdose with study intervention.

All overdoses should be reported as an SAE with the safety criteria of "**other important medical event**." Details of signs and symptoms, clinical management, and outcome should be reported, if applicable. Overdoses should also be captured as protocol deviations.

The PI has the obligation to report the deviations to the IRB/IEC.

8.5 PHARMACOKINETICS

Pharmacokinetic parameters are not evaluated in this study.

8.6 ANTI-DRUG ANTIBODIES

Antibodies to nivolumab and/or ipilimumab are not evaluated in this study.

8.7 BIOMARKERS

The percentage of tumoral CD8 cells will be determined from tumor tissue using immunohistochemistry (IHC) by the Immunohistochemistry and Image Analysis Laboratories in the Department of Pathology at MDACC's CLIA- and CAP-certified laboratory. The test for CD8 expression is a laboratory developed test used to determine participant eligibility. This test is not an FDA-approved device and its use is investigational. Tumor biopsies with ≥ 15% CD8 cells in their tumor biopsies will be classified as CD8 high tumors, and those with < 15% CD8 cells in their tumor biopsies will be classified as CD8 low tumors. The percentage of tumoral CD8 cells at baseline will determine immunotherapy treatment (nivolumab with or without ipilimumab) to evaluate if the percentage of CD8 cells can predict clinical benefit to nivolumab. On treatment, the percentage of tumoral CD8 cells in tumor biopsies will be evaluated to determine if nivolumab in combination with ipilimumab converts CD8 low tumors to CD8 high tumors.

For baseline biopsy samples, the tissue is deemed "adequate" for CD8 IHC testing when the pathologist determines that the section contains > 20% tumor nuclei (ie, 20% of the total nuclei visualized in the section are from tumor cells). However, the sample adequacy metric when applied to on-treatment biopsy samples may inadvertently exclude from analysis cases in which an active immune response results in the destruction of tumor cells and, consequently, a decrease in the percentage of tumor nuclei in the sample (a "tumor-free" sample). Therefore, the definition of sample adequacy for on-treatment biopsy samples is being clarified such that CD8 IHC testing

will be performed and reported for samples with features indicative of a treated tumor bed consisting of viable components of an effective, anti-tumor fibroinflammatory response. For these cases, the pathologist will note in the CLIA report the presence or absence of tumor cells as detected and will note histological features indicative of an immune response (eg, giant cells, fibrohistiocytic characteristics, etc.).

The exploratory biomarker objectives of this study are to identify biomarkers associated with anti-PD-1 and/or anti-PD-1 combined with anti-CTLA-4 study intervention by assessing tumor tissue and circulating soluble factors, including, but not limited to, deoxyribonucleic acid (DNA), ribonucleic acid (RNA), enzymes, growth factors, cytokines, antibodies, and immune cells in tissue and blood. An aim of this study is to evaluate if a composite biomarker (CD8 with a second biomarker) is able to predict clinical benefit to checkpoint inhibitor treatment. Additionally, microbiome profiles may be evaluated from stool samples. Evaluation of baseline levels and/or changes with study intervention may be performed to determine association with clinical outcomes, including clinical response and resistance, as well as study intervention tolerability.

- Collection of samples for biomarker research is part of this study. The following samples for biomarker research are required and will be collected from all participants in this study as specified in the SOAs (Section 1.3):
  - Blood
  - Peripheral blood mononuclear cells (PBMCs)
  - DNA
  - Tumor tissue biopsy (at intervals specified in Section 4.1)
  - Stool
- Samples may be tested for genetic analysis on tumor and blood samples, including, but not limited to, assays on circulating free DNA, DNA from tumor, blood and/or immune cells and T-cell receptor sequencing may be performed. This research may evaluate whether genetic variations correspond with outcomes of treatment. If genetic variation is found to predict efficacy or AEs, the data might inform optimal use of therapies in cancer participants. Circulating soluble analytes may be assessed that may include but are not limited to immune cytokines, growth factors, antibodies, and/or markers associated with immune characteristics and activation or cancer. Additionally, tumor and blood samples will be collected before and on study intervention for immune cell profiling, which may include immune cell phenotyping, enumerations, and/or activation state. Both genome-wide and targeted messenger RNA expression profiling and sequencing in tumor and/or blood may be performed to define gene signatures that correlate with treatment outcomes. Epigenetic analyses may also be performed as these are important biomarkers for some cancers. Stool samples at baseline and on treatment may be evaluated for microbiome profiling to determine if there is any association with treatment outcome.

- Other samples may be used for research, including future research to develop methods, assays, prognostics and/or companion diagnostics related to immuno-oncology treatment, disease process, pathways associated with disease state, and/or mechanisms of action of checkpoint inhibitor treatment.

8.7.1 Genetics

Sample collection, storage, and shipment instructions for planned genetic analysis samples will be provided in the Laboratory Manual. Samples should be collected for planned analysis of associations between genetic variants in germline/tumor DNA and clinical outcomes to study inventions(s). Blood for planned genetic analysis will be collected for DNA as described in the SOAs in Section 1.3. If a documented law or regulation prohibits (or local IRB/IEC does not approve) sample collection for these purposes, then such samples should not be collected at the corresponding sites. Additional DNA extracted from planned genetic analysis samples will be stored for future biomedical research.

In the event of DNA extraction failure, a replacement genetic blood sample may be requested from the participant. Signed informed consent will be required to obtain a replacement sample unless it was included in the original consent.

See Appendix 7: for Information regarding genetic research.

8.7.2 Enrollment Biomarkers

Core needle or incisional tumor biopsy samples (3-4 cores if medically feasible) are required at screening for CD8 IHC testing and classification of CD8 high vs CD8 low tumors. Fine needle aspiration samples are not acceptable. This test is required for enrollment. The test for CD8 expression is a laboratory developed test used to determine participant eligibility. This test is not an FDA-approved device and its use is investigational. Sample collection, storage, and shipment instructions are provided in the Laboratory Manual.

Immunohistochemistry will be performed in the CLIA-certified Immunohistochemistry and Image Analysis Laboratories in the Department of Pathology at MDACC using a Leica Bond autostainer and an anti-CD8 antibody (Lab Vision, Thermo-Fisher; clone: C8/144B; dilution 1:20) with 3,3'-diaminobenzidine chromogen, counterstained with hematoxylin.

CD8 IHC slides will be scanned at ×20 magnification (Leica Biosystems). Image analysis software (Aperio ImageScope) will be applied to quantify the number and percentage of IHC-positive lymphocytes within designated areas. Given the size of CD8-postive immune cells, a modified version of the nuclear v9 algorithm has been devised as a basis for detecting immune marker positivity. This algorithm has been used to quantify CD8-positive cells in different tumor types, including melanoma and Merkel cell carcinoma (Chen et al, 2016; Feldmeyer et al, 2016).

To quantify the levels of CD8 in a tumor, areas will be marked by a pathologist manually (ie, hand drawn around the tumor foci for needle core biopsies and other uniquely shaped fragments of tissue) or by applying 1-mm^2 square boxes to quantify larger fragments of tumor. The Aperio image analysis software will quantify (1) the percentage of CD8-positive cells and (2) the number of CD8-positive cells in a given area (the latter reported as CD8+ cells/mm^2). Tumors with ≥ 15% CD8 cells will be classified as CD8 high tumors for participant treatment with nivolumab and < 15% CD8 cells will be classified as CD8 low tumors for participant treatment with nivolumab and ipilimumab.

If the results of the screening biopsy are not adequate to obtain information on the percentage of tumoral CD8 cells for treatment assignment, a second pre-treatment biopsy may be obtained, if it is medically feasible and the participant is willing to undergo another tumor biopsy.

8.7.3 CD8 and Exploratory Biomarkers

A fresh biopsy is required at screening for enrollment CD8 IHC analysis and other exploratory analysis. If the participant had a previous biopsy within 30 days of consent this may be used as the enrollment biopsy. However, the sample must provide one or two FFPE blocks for IHC analysis. Slides are not allowed. If the archival biopsy is beyond 30 days of consent, this will require Medical Monitor approval. Additional on-treatment tumor biopsies should occur if medically feasible per the SOA (see Section 1.3). Core needle or incisional tumor biopsy samples are required. Furthermore, every attempt should be made for the on-treatment biopsies to be taken from the same lesion site. Fine needle aspiration is not allowed.

Blood will be collected for exploratory biomarkers. Blood may be used for whole blood, PBMCs, plasma, and/or serum preparation and nucleic acid extraction. These blood samples will be collected as described in the SOAs in Section 1.3.

Stool samples will be collected for microbiome analysis, which may include metabolites as outlined in the SOAs in Section 1.3.

Tissue, blood, and stool sample collection, storage, and shipment instructions are provided in the Laboratory Manual.

8.7.4 Sample Collection for Long-term Future Biomedical Research

8.7.4.1 Overview of Long-term Future Biomedical Research

Participants in this clinical trial will be asked to consent to provide biological samples for long-term future biomedical research. The objective of collecting/retaining specimens for future biomedical research is to explore and identify biomarkers that inform the scientific understanding of disease and/or their therapeutic treatments. The overarching goal is to use such information to understand disease, safety, and potential treatments for future participants. Such

research is for biomarker testing and hypothesis testing to address emergent questions not described elsewhere in the protocol (as part of the main trial).

This research may include genetic and genomic analyses (DNA), gene expression profiling (RNA), proteomics, microbiome, metabolomics (serum, plasma, stool), and/or the measurement of other analytes, depending on which specimens are consented for future biomedical research.

The collection and submission of biological samples to be stored for long-term future biomedical research must be detailed in the IRB/IEC-approved ICF. Participants who do not wish to participate in the future biomedical research may still participate in the study.

8.7.4.2 Sample Collection

The following samples will be collected and stored in accordance with applicable law for long-term research purposes, including, but not limited to, research on biomarkers related to immunotherapies, such as anti-PD-1 and/or anti-CTLA-4 therapy, and diseases such as cancer or inflammatory disorders.

- Blood, including but not limited to PBMCs, DNA, plasma, and/or serum
- RNA
- Tumor tissue
- Stool samples

These samples may be sent to one or more laboratories.

For sampling procedures, storage conditions, and shipment instructions, see the Laboratory Manual.

8.7.4.3 Withdrawal from Long-term Sample Storage

Participants have the right to withdraw their consent for the future biomedical research of his/her specimens at any time for any reason and request that their specimens be destroyed. If the participant wishes to withdraw consent for this testing, the Investigator must inform the Medical Monitor in writing. Any analyses in progress at the time of request for withdrawal or already performed prior to the request being received by the Sponsor will continue to be used as part of the overall research trial data and results. No new analyses would be generated after the request is received. In the event that the medical records for the main trial are no longer available (eg, if the Investigator is no longer required by regulatory authorities to retain the main trial records) or the specimens have been completely anonymized, there will no longer be a link between the participant's personal information and their specimens. In this situation, the request for specimen withdrawal cannot be processed.

8.7.4.4 Protection of Data Privacy and Data Generation

Participant specimens and associated data will be labeled with a unique participant identification number. Participant medical information associated with the long-term storage of specimens is confidential and may be disclosed to third parties only as permitted by the ICF signed by the participant or as permitted or required by law.

Given the complexity and research nature of the exploratory analyses, data derived from long-term stored specimens will generally not be provided to study investigators or participants unless required by law.

Data generated from specimens that are stored long term must be available for inspection upon request by representatives of national or local health authorities and Sponsor monitors, representatives, and collaborators as appropriate and as described in Section 10.1.10.

8.8 PATIENT-REPORTED OUTCOMES RESEARCH

Participants enrolled in the prostate cancer cohort may be offered the option to use ApricityCare™, a patient-reported outcome (PRO) digital application, during their participation in this study. Study staff will have an opportunity to review participant's data at the ApricityOncology™, a clinical dashboard website.

The ApricityCare™ smartphone app allows participants (or their designee) to self-report adverse events in between clinic visits. The app also provides educational information and videos about potential AEs participants may experience. ApricityCare™ was initially developed for the identification and management of irAEs in participants receiving standard-of-care immunotherapy for cancer.

Inclusion of the technology in the current PICI0025 AMADEUS prostate cancer cohort will generate important data on the feasibility of incorporating this approach to PRO collection in a clinical research setting. Participation in this research is optional for study participants, and the use of ApricityCare™ will not impact the participant's treatment and is not a substitute for clinical evaluation. The data collected via this app for participants as well as the ApricityOncology app for the study team is for exploratory research purposes only and will not be incorporated in the PICI0025 electronic data capture system. In the event of AEs or SAEs that meet reporting criteria per Section 8.3, providers and study team members will continue to follow reporting procedures per Section 10.5. The de-identified data collected via ApricityCare™ and ApricityOncology may be used by Sponsor's researchers and its affiliates, Apricity Health, and/or shared with other researchers and/or institutions for use in future research.

The clinician-facing portion of this application will produce recommendations for irAE management as advisory to clinicians and investigators. The management guidelines will use algorithms and the toxicity management guidelines from Table 7. These do not constitute Medical Advice and will not be presented directly to participants.

8.8.1 Medical Disclaimer

The ApricityCare™ app does not make any medical diagnosis, does not provide any treatment, and does not substitute medical service or communication with a healthcare provider. The software used in this patient-reported study is intended for research purposes only.

All content found on the ApricityCare™ app, including text, images, audio, or other formats, are only for research. If Medical Assistance is needed, participants are instructed via the ApricityCare™ app to call 911 and alert their health care provider.

8.8.2 Health Data Protection

Participant's medical information obtained by the AMADEUS study and this optional procedure is confidential. It may be disclosed to third parties only as permitted by the ICF (or separate authorization for use and disclosure of personal health information) signed by the participant or as permitted or required by law.

Apricity will provide training to site staff. PICI's monitors will not have access to the data collected by ApricityCare™ and will not monitor, interfere with or evaluate Apricity's data collection or procedures.

Participants will be registered and receive instructions and training to use the application via the site-level research team. Consenting participants will be requested to download a software application into their smartphone devices. Devices will not be provided. Participants are expected to start using the app daily from the time of informed consent until the safety period to track daily routine and self-monitoring of symptoms. During the participant's end of treatment visit, they will be asked to survey their experience using the ApricityCare™ app.

Throughout the study, the research staff are encouraged to follow the study procedures, in addition to those included in the SOA of this protocol and are not limited to:

- On-boarding to register and train participants in the use of the ApricityCare™ app.
- Reviewing participant’s data via ApricityOncology™, a clinical dashboard website at regular intervals per training materials.

Definitions	
ApricityCare™	A smartphone digital application designed for collection of patient-reported assessments.
ApricityOncology™	A clinical dashboard website available for study staff to review participant's data.

8.9 MEDICAL RESOURCE UTILIZATION AND HEALTH ECONOMICS

This section is not applicable.

9 STATISTICAL CONSIDERATIONS

9.1 SAMPLE SIZE DETERMINATION

A total of up to approximately 200 participants with varying tumor types will be enrolled. Enrollment to initial biopsy may be about 10% higher (ie, approximately 220 participants) to account for unexpected challenges obtaining CD8 infiltration information. The selection of 200 participants is to have sufficient numbers in the CD8 high and CD8 low tumor groups in larger tumor populations to report subgroup response rates, the transformation from CD8 low to high, as well as to predict response from marker and clinical information. With 200 participants, approximately 40 and 160 participants are estimated for the CD8 high and CD8 low tumor groups, respectively (Figure 7). Clinical benefit rate (CBR) within tumor groups is expected to range from 10% to 30% (Nivolumab IB). An average CBR of 20% is assumed to provide approximately 32 participants with clinical benefit in the CD8 low tumor group. With 160 participants, a 95% credible interval of CBR with 32 responders would be (14.2%, 26.5%) assuming a beta(0.4, 1.6) prior. Similarly, if there are 30 participants in the largest disease subgroup, a 95% credible interval of response would be (8.3%, 35.2%) assuming 6 responders and a similar prior. There is no prior for the expected proportion of participants who will convert from CD8 low to CD8 high on treatment as this is a first look for this endpoint. The 95% credible interval for 16/160 (10%) participants converting would be (5.9%, 15.1%) and for 80/160 (50%) the credible interval would be (42.3%, 57.7%). Similarly, 8 of 40 participants are in the CD8 high group expected to exhibit benefit, resulting in a 95% credible interval of (9.5%, 33.2%). The selection of 200 participants allows a minimum reasonable number for estimating CBR in the CD8 high tumor group. For secondary analyses, if there are 20 responders in any CD8 status and/or tumor site group, it would be reasonable to perform multivariate logistic regression with 2 variables to predict response. Therefore, 200 participants is the smallest reasonable sample size for the initial exploration of efficacy prediction in this population.

Additional widths of credible intervals of CBR for possible subgroups sizes are presented in Table 11.

Approximately 20 advanced prostate cancer participants with CD8 low tumors will be randomly allocated by the Sponsor into one of two cohorts (10 participants per cohort) to evaluate different dose levels of ipilimumab in combination with nivolumab. We hypothesize that higher doses of ipilimumab will demonstrate a larger proportion of participants who convert from CD8 low to CD8 high. There is no prior for the expected proportion of participants who will convert from CD8 low to CD8 high at the various dose levels of ipilimumab. With 10 participants enrolled per cohort, Table 12 provides 95% credible intervals for possible CD8 conversion rates in each cohort. For example, a 95% credible interval assuming 4/10 (40%) participants converting in a cohort would be (13.7%, 70.1%).

Table 11: Possible Subgroup Sizes

Subgroup Size	Clinical Benefit Rate	Expected Success	95% Credible Interval
160	10	16	(6.0, 15.2)
125	10	12	(5.3, 15.5)
100	10	10	(5.1, 16.7)
75	10	8	(5.0, 18.7)
50	10	5	(3.7, 19.9)
40	10	4	(3.2, 21.2)
30	10	3	(2.6, 23.2)
160	20	32	(14.2, 26.5)
125	20	25	(13.5, 27.4)
100	20	20	(12.9, 28.3)
75	20	15	(11.9, 29.6)
50	20	10	(10.4, 31.8)
40	20	8	(9.5, 33.2)
30	20	6	(8.3, 35.2)
160	30	48	(23.1, 37.1)
125	30	38	(22.6, 38.5)
100	30	30	(21.4, 39.0)
75	30	22	(19.6, 39.6)
50	30	15	(18.1, 42.6)
40	30	12	(16.9, 44.0)
30	30	9	(15.2, 46.0)

Table 12: Credible Intervals for CD8 Conversion

Subgroup Size	CD8 Conversion Rate	Expected Successes	95% Credible Interval
10	20	2	(2.8, 48.2)
10	30	3	(7.5, 60.0)
10	40	4	(13.7, 70.1)
10	50	5	(21.2, 78.8)

9.2 POPULATIONS FOR ANALYSIS

For purposes of analysis, the following populations are defined (Table 13):

Table 13: Populations for Analysis

Population	Description
Safety Population	All participants who received at least one dose of study drug. Participants will be analyzed according to the study intervention actually received.
Modified Intent-to-Treat (mITT) Population	All participants with sufficient biopsy results to be assigned to a treatment arm and who received at least 1 dose of study drug. Participants will be analyzed according to the study intervention to which they were assigned.
On-treatment Biopsy Population	All participants with at least 1 on-treatment biopsy with sufficient CD8 results will be included in the analyses of changes in CD8 counts

9.3 INTERIM ANALYSES

9.3.1 Safety and Futility Monitoring

Ongoing monitoring for safety and futility will be implemented based on the method of Thall and colleagues (Thall et al, 1995) separately in the CD8 high and CD8 low tumor groups for advanced metastatic cancer and advanced prostate cancer participants. Monitoring will be ongoing during enrollment in each group, once a participant receives treatment and their first treatment biopsy is performed. Screening and accrual to each group will continue until sufficient information is available to assess the stopping rules as defined below. Stopping rules and operating characteristics were calculated using Multc Lean v2.1.

9.3.1.1 Safety Monitoring

For trial monitoring and decisions about future trials, TOX criteria are defined in Section 8.3.7. A participant who voluntarily leaves the trial for any reason, including toxicity that does not meet the TOX criteria, will not count as having a TOX. Denote the probability of TOX by θ_T.

The stopping rule for advanced metastatic cancer arms is given by the following probability statement: Pr ($\theta_T > 0.40$ | data) > 0.975. That is, the trial will be stopped if, at any time during the study, it is determined that there is > 97.5% chance that the TOX rate is > 40%. The stopping rules assume a constant TOX rate for "standard therapy" of 0.40 (Larkin et al, 2015; Wolchok et al, 2017) and a prior θ_T ~ beta (0.8, 1.2) for the current study. The stopping boundaries for this toxicity rule are to terminate the trial if the number of participants with TOX compared to the number of participants having received treatment exceeds the limits in Table 14.

Table 14: Rules for Safety and Futility Monitoring per Arm

If there are this many (or more) participants evaluable for response	Stop the arm if there are this many (or more) participants with a TOX event:	Stop the arm if there are this many (or fewer) participants who meet the definition of success defined above:
20	13	1
40	23	3
60	32	6
80	41	9
100	50	12
120	59	15
140	68	19

9.3.1.2 Futility Monitoring

For trial monitoring, "success" will be defined separately by tumor group. For participants with CD8 low tumors, participants will be counted as having success if they meet the definition of CBR *or* if the tumor converts from CD8 low to CD8 high expression. For participants in the CD8 high tumor group, participants will be counted as success if they meet the definition of CBR. Denote the probability of success by θ_S. We assume θ_S ~ beta (0.4, 1.6). The stopping rule is given by the following probability statement: Pr($\theta_S < 0.20$ | data) > 0.975. That is, participant enrollment will be stopped if, at any analysis time, it is determined that there is > 97.5% chance that the CBR is < 20%, a constant rate that is at the average expected response rate for these participants. The stopping boundaries for this futility rule, shown in Table 14, are designed to terminate enrollment on the arm if the number of overall responses compared with the number of participants enrolled who are evaluable does not meet the requirements of this table. A participant is evaluable for benefit either on the date of disease assessment or the date the participant goes off study without benefit or if the participant does not receive the scheduled disease assessment within 3 weeks without associated scheduling delay. Monitoring will be carried out during enrollment in each group. Participants who withdraw from the study after

receiving treatment but before undergoing on-treatment biopsies or who miss the evaluation window without associated treatment delay will be counted as not benefitting and therefore evaluable for analysis. CBR is defined as best response of CR or partial response (PR) any time or stable disease (SD) lasting 6 months. For futility monitoring purposes, confirmation of response (CR or PR) is not required. Additionally, a participant who has been on study between 12 weeks and 6 months and has SD at the time of data collection will be counted as a success for that futility analysis. If that participant later progresses and no longer meets the SD definition, their updated information will be included in the monitoring analysis (Table 14).

9.3.1.3 Joint Operating Characteristics for Safety and Futility Monitoring

The rules above will apply independently to each CD8 high or CD8 low tumor group. It is estimated that there will be 40 participants with CD8 high tumors and 160 participants with CD8 low tumors in presenting the operating characteristics below. The probability of stopping at 20 participants provides the probability of stopping early in the CD8 high tumor group, assuming only 40 participants are entered. Table 15 shows the operating characteristics under varying toxicity and response rates.

Table 15: Operating Characteristics Under Varying Toxicity and Response Rates

True Overall Toxicity Rate	True Overall Response Rate	Probability of Stopping at 20 Participants	Probability of Stopping Before 160 Participants	Median (25th %ile, 75th %ile) for "Cold" Cohort
0.10	0.05	0.74	>0.99	20 (20, 40)
	0.10	0.39	0.96	40 (20, 80)
	0.20	0.07	0.12	160 (160, 160)
	0.30	0.01	0.01	160 (160, 160)
	0.40	0.001	0.001	160 (160, 160)
0.20	0.05	0.74	>0.99	20 (20, 40)
	0.10	0.39	0.96	40 (20, 80)
	0.20	0.07	0.12	160 (160, 160)
	0.30	0.01	0.01	160 (160, 160)
	0.40	0.001	0.001	160 (160, 160)
0.30	0.05	0.74	>0.99	20 (20, 40)
	0.10	0.39	0.96	40 (20, 80)
	0.20	0.07	0.12	160 (160, 160)
	0.30	0.01	0.01	160 (160, 160)
	0.40	0.002	0.002	160 (160, 160)
**0.40**	0.05	0.74	>0.99	20 (20, 40)

True Overall Toxicity Rate	True Overall Response Rate	Probability of Stopping at 20 Participants	Probability of Stopping Before 160 Participants	Median (25th %ile, 75th %ile) for “Cold” Cohort
	0.10	0.40	0.96	40 (20, 80)
	**0.20**	**0.09**	**0.19**	160 (160, 160)
	0.30	0.03	0.09	160 (160, 160)
	0.40	0.02	0.08	160 (160, 160)
0.50	0.05	0.77	>0.99	20 (20, 20)
	0.10	0.47	0.99	40 (20, 60)
	0.20	0.19	0.78	80 (40, 140)
	0.30	0.14	0.75	80 (40, 140)
	0.40	0.13	0.75	80 (40, 160)

9.4 STATISTICAL ANALYSES

The study will use an adaptive approach, with on-treatment data guiding trial adaptation and success or futility of each combination in each study subpopulation.

The statistical analysis plan will be developed and finalized before database lock and will describe the study populations to be included in the analyses and procedures for accounting for missing, unused, and spurious data. The statistical analysis plan will serve as a complement to the protocol and supersedes it in case of differences. This section is a summary of the planned statistical analyses of the primary and secondary endpoints.

9.4.1 Efficacy Analyses

The efficacy analysis will be based on the modified Intent-to-Treat (mITT) Population, which comprises all enrolled participants with sufficient biopsy results to be assigned to a treatment arm and who received at least one dose of study drug, unless otherwise specified. Participants will be analyzed according to the study intervention to which they were assigned.

The primary endpoint of CBR will be reported among all participants within each study intervention group with a 95% credible interval assuming a beta (0.4, 1.6) prior. Per RECIST, to be assigned a best overall response of CR or PR, changes in tumor measurements must be confirmed by a repeat assessment that should be performed no less than 4 weeks after the criteria for response are first met. Participants who do not have a disease assessment will be counted as not having clinical benefit. In addition, CBR and similar credible intervals will be reported for each tumor site and study intervention subgroup that has at least 10 participants.

The proportion of participants whose tumors change from CD8 low to CD8 high expression will be reported with a 95% credible interval with no prior as this is the first look.

For secondary endpoints, baseline CD8 counts will be explored for association with CBR using logistic regression models and with OS using Cox regression models. Multivariate models will be explored with up to 1 variable for every 10 events. For CBR prediction, an event is an objective response (CR or PR or SD as defined above). For association with survival, an event is a participant death.

Among the On-treatment Biopsy Population, both the baseline and on-treatment biopsies will be associated with response and survival using similar logistic and Cox regression models described above, assuming there are sufficient events to carry out the regression analyses. The association of CD8 changes from baseline to on treatment with clinical outcomes will also be explored.

The exploratory endpoint of changes in PSA (absolute and percent) from baseline will be analyzed by cohort for advanced prostate cancer participants.

9.4.2 Safety Analyses

The safety analysis will be based on the Safety Population, which comprises all participants who receive at least one dose of study drug. Participants will be analyzed according to the study intervention actually received.

Safety and tolerability will be assessed through AEs, clinical laboratory parameters, vital signs, and ECGs.

AEs will be described by grade and attribution for each study intervention.

9.4.3 Other Analyses

Biomarker exploratory analyses will be performed when possible and included in the clinical study report or a separate research report.

9.4.3.1 Biomarker Analysis

Descriptive statistics will be used to evaluate the exploratory biomarkers. Biomarker analysis will be further described in a biomarker analysis plan.

9.4.3.2 Patient-Reported Outcomes Analysis

An exploratory analysis may be performed with the patient-reported dataset collected via ApricityCare™, including but not limited to the frequency with which participants complete the daily symptom survey in ApricityCare™, and the frequency of Apricity website access by investigative sites.

10 SUPPORTING DOCUMENTATION AND OPERATIONAL CONSIDERATIONS

10.1 APPENDIX 1: REGULATORY, ETHICAL AND STUDY OVERSIGHT CONSIDERATIONS

10.1.1 Compliance with Laws and Regulations

This study will be conducted in full conformance with the International Council for Harmonisation (ICH) E6 guideline for Good Clinical Practice (GCP) and the consensus ethical principles derived from international guidelines, including the Declaration of Helsinki and Council for International Organizations of Medical Sciences International Ethical Guidelines, and applicable laws and regulations. The study will comply with the requirements of the ICH E2A guideline (Clinical Safety Data Management: Definitions and Standards for Expedited Reporting). Studies conducted in the United States or under a U.S. Investigational New Drug (IND) application will comply with U.S. FDA regulations and applicable local, state, and federal laws. Studies conducted in the European Union or European Economic Area will comply with the E.U. Clinical Trial Directive (2001/20/EC).

10.1.2 Institutional Review Board or Independent Ethics Committee

The protocol, protocol amendments, ICF(s), Investigator's Brochure, any information to be given to the participant, and relevant supporting information must be submitted to the IRB/IEC and reviewed and approved by the IRB/IEC before the study is initiated. In addition, any participant recruitment materials (eg, advertisements) must be approved by the IRB/IEC.

Any amendments to the protocol will require IRB/IEC approval before implementation of changes made to the study design, except for changes necessary to eliminate an immediate hazard to study participants or changes that involve logistical or administrative aspects only (eg, change in Medical Monitor or contact information).

The Investigator will be responsible for the following:

- Providing written summaries of the status of the study to the IRB/IEC annually or more frequently in accordance with the requirements, policies, and procedures established by the IRB/IEC
- Notifying the IRB/IEC of SAEs or other significant safety findings as required by IRB/IEC procedures
- Promptly documenting and reporting any deviations that might have an impact on participant safety and data integrity to the Sponsor and to the IRB/IEC in accordance with established requirements, policies and procedures

- Providing oversight of the conduct of the study at the site and adherence to requirements of 21 CFR, ICH guidelines, the IRB/IEC, European regulation 536/2014 for clinical studies (if applicable), and all other applicable local regulations.

The IRB of Record is MD Anderson and the Relying IRBs are UCLA, UCSF, Stanford, DFCI, and MSKCC._Parker maintains separate documentation of the institution's address, FWA number and expiration date, and the named Institutional Official and IRB Contact, as well as their email and phone numbers and will provide that to the sites.

10.1.3 Financial Disclosure

Investigators and Subinvestigators will provide the Sponsor with sufficient, accurate financial information as requested to allow the Sponsor to submit complete and accurate financial certification or disclosure statements to the appropriate health authorities. Investigators are responsible for providing information on financial interests during the course of the study and for 1 year after completion of the study (see definition of end of study in Section 4.5).

10.1.4 Informed Consent

The Investigator or his/her representative will explain the nature of the study to the participant or his/her legally authorized representative and answer all questions regarding the study.

Participants must be informed that their participation is voluntary. Participants or their legally authorized representative will be required to sign a statement of informed consent that meets the requirements of 21 CFR 50, local regulations, ICH guidelines, Health Insurance Portability and Accountability Act (HIPAA) requirements, where applicable, and the IRB/IEC or study center.

The medical record must include a statement that written informed consent was obtained before the participant was enrolled in the study and the date the written consent was obtained. The authorized person obtaining the informed consent must also sign the ICF.

If applicable, the ICF will contain separate sections for any optional procedures. The Investigator or authorized designee will explain to each participant the objectives, methods, and potential risks associated with each optional procedure. Participants will be told that they are free to refuse to participate and may withdraw their consent at any time for any reason. A separate, specific signature will be required to document a participant's agreement to participate in optional procedures. Participants who decline to participate will not provide a separate signature.

Participants must be re-consented to the most current version of the ICF(s) (or to a significant new information/findings addendum in accordance with applicable laws and IRB/IEC policy) during their participation in the study. The medical record should document the re-consent process and that written informed consent was obtained using the updated/revised ICF for continued participation in the study. A participant who is rescreened is not required to sign

another ICF if the rescreening is beyond 30 days from C1D1 unless the institution's policy is to perform re-consent. In that case, PICI accepts the institution's policy on re-consent.

A copy of the ICF(s) must be provided to the participant or to the participant's legally authorized representative. All signed and dated ICFs must remain in the participant's study file or in the site study file and must be available for verification by study monitors at any time.

The final revised IRB/IEC-approved ICFs must be provided to the Sponsor for the purpose of health authority submission.

A participant who is rescreened is not required to sign another ICF if the rescreening occurs within 30 days from the previous ICF signature date.

The ICF will contain a separate section that addresses the use of remaining mandatory samples for optional exploratory research. The Investigator or authorized designee will explain to each participant the objectives of the exploratory research. Participants will be told that they are free to refuse to participate and may withdraw their consent at any time and for any reason during the storage period. A separate signature will be required to document a participant's agreement to allow any remaining specimens to be used for exploratory research. Participants who decline to participate in this optional research will not provide this separate signature.

10.1.5 Data Protection

The Sponsor maintains confidentiality standards by assigning a unique participant identification number to each participant enrolled in the study. This means that participant names are not included in data sets that are transmitted to any Sponsor location.

Participant medical information obtained by this study is confidential and may be disclosed to third parties only as permitted by the ICF (or separate authorization for use and disclosure of personal health information) signed by the participant or as permitted or required by law.

Medical information may be given to a participant's personal physician or other appropriate medical personnel responsible for the participant's welfare, for treatment purposes.

Data generated by this study must be available for inspection upon request by representatives of national and local health authorities, Sponsor monitors, representatives and collaborators, and the IRB/IEC for each study site, as appropriate.

10.1.6 Administrative Structure

This trial will be sponsored and managed by the Parker Institute for Cancer Immunotherapy. The Sponsor will provide clinical operations management, data management, and medical monitoring.

Central facilities will be used for certain study assessments throughout the study (eg, specified laboratory test and biomarker analyses), as specified in Section 8.2.5 and Section 8.7, respectively. Accredited local laboratories will be used for routine monitoring; local laboratory ranges will be collected.

10.1.7 Data Quality Assurance

All participant data relating to the study will be collected via the electronic data collection (EDC) system on an eCRF unless transmitted to the Sponsor or designee electronically (eg, central laboratory data, biomarker, and other biological sample data). Sites will be responsible for data entry into the EDC system and will receive training for appropriate eCRF completion. The Investigator is responsible for verifying that data entries are accurate and correct by electronically signing and dating the eCRF.

The Investigator must maintain accurate documentation (source data, see Section 10.1.8) that supports the information entered in the eCRF. The Investigator must permit study-related monitoring, audits, IRB/IEC review, and regulatory agency inspections and provide direct access to source data documents.

The Sponsor or designee is responsible for the data management of this study, including quality checking of the data. Study monitors will perform ongoing source data verification to confirm that data entered on the eCRF by authorized site personnel are accurate, complete, and verifiable from source documents; that the safety and rights of participants are being protected; and that the study is being conducted in accordance with the currently approved protocol and any other study agreements, ICH GCP, and all applicable regulatory requirements.

At the end of the study, the Investigator will receive participant data for his/her site in a readable format on a compact disc (or other readable digital format) that must be kept with the study records. Acknowledgement of receipt of the compact disc is required.

10.1.8 Source Documentation

Source documents provide evidence for the existence of the participant and substantiate the integrity of the data collected. Source documents are filed at the Investigator's site. Data entered on the eCRF that are transcribed from source documents must be consistent with the source documents or the discrepancies must be explained. The Investigator may need to request previous medical records or transfer records, depending on the study. Also, current medical records must be available.

Source documents (paper or electronic) are those in which participant data are recorded and documented for the first time. They include, but are not limited to, hospital records, clinical and office charts, laboratory notes, memoranda, clinical outcome assessment (COA)/PRO, evaluation checklists, pharmacy dispensing records, recorded data from automated instruments, copies of

transcriptions that are certified after verification as being accurate and complete, microfiche, photographic negatives, microfilm or magnetic media, X-rays, participant files, and records kept at pharmacies, laboratories, and medico-technical departments involved in a clinical trial.

When clinical observations are entered directly into a study site's computerized medical record system (ie, in lieu of original hardcopy records), the electronic record can serve as the source document if the system has been validated in accordance with health authority requirements pertaining to computerized systems used in clinical research. An acceptable computerized data collection system allows preservation of the original entry of data. If original data are modified, the system should maintain a viewable audit trail that shows the original data as well as the reason for the change, name of the person making the change, and date of the change.

Source documents that are required to verify the validity and completeness of data entered on the eCRFs must not be obliterated or destroyed and must be retained as described in Section 10.1.11.

10.1.9 Study and Site Closure

The Sponsor or designee reserves the right to close the study site or terminate the study at any time for any reason at the sole discretion of the Sponsor. Reasons for terminating the study may include, but are not limited to, the following:

- Discontinuation of further study intervention development
- The incidence or severity of AEs in this or other studies indicates the potential hazard to participants
- Participant enrollment is unsatisfactory

The Sponsor will notify the Investigator if the Sponsor decides to discontinue the study.

Reasons for the early closure of a study site by the Sponsor or Investigator may include, but are not limited to, the following:

- Poor protocol adherence
- Inaccurate or incomplete data recording
- Failure of the Investigator to comply with the protocol, the requirements of the IRB/IEC or local health authorities, the Sponsor's procedures, or GCP guidelines
- No study activity (ie, all participants have completed the study and all obligations have been fulfilled)

10.1.10 Site Inspections

Site visits will be conducted by the Sponsor or an authorized representative for inspection of study data, participants' medical records, and eCRFs. The Investigator will permit national and local health authorities; Sponsor monitors, representatives, and collaborators; and the IRBs/IECs to inspect facilities and records relevant to this study.

10.1.11 Retention of Records

Records and documents pertaining to the conduct of this study and distribution of the investigational medicinal product, including signed eCRFs, electronic or paper participant-reported outcomes data (if applicable), signed ICFs, laboratory test results, and medication inventory records, must be retained by the Investigator for the maximum period required by applicable regulations of relevant national or local health authorities. No records may be disposed of without the written approval of the Sponsor. The Sponsor will notify the Investigator when the records are no longer needed. Following notification from the Sponsor, the documents may be destroyed, subject to local regulations.

Written notification should be provided to the Sponsor prior to transferring any records to another party or moving them to another location.

10.1.12 Publication Policy and Protection of Trade Secrets

Regardless of the outcome of a trial, the Sponsor is dedicated to openly providing information on the trial to healthcare professionals and to the public, both at scientific congresses and in peer-reviewed journals. The Sponsor will comply with all requirements for publication of study results.

The Investigator must agree to submit all manuscripts or abstracts to the Sponsor prior to submission for publication or presentation. This allows the Sponsor to protect proprietary information and to provide comments based on information from other studies that may not yet be available to the Investigator.

The Sponsor will comply with the requirements for publication of study results. In accordance with standard editorial and ethical practice, the Sponsor will generally support publication of multicenter trials only in their entirety and not as individual center data. In this case, a coordinating Investigator will be designated in accordance with the Parker Institute for Cancer Immunotherapy publication policy. Authorship will be based on overall scientific contribution and participant enrollment.

10.2 APPENDIX 2: EASTERN COOPERATIVE ONCOLOGY GROUP (ECOG) PERFORMANCE STATUS

Grade	ECOG Performance Status
0	Fully active, able to carry on all pre-disease performance without restriction
1	Restricted in physically strenuous activity but ambulatory and able to carry out work of a light or sedentary nature, eg, light house work, office work
2	Ambulatory and capable of all self-care but unable to carry out any work activities; up and about more than 50% of waking hours
3	Capable of only limited self-care; confined to bed or chair more than 50% of waking hours
4	Completely disabled; cannot carry on any self-care; totally confined to bed or chair
5	Dead

Oken MM, Creech RH, Tormey DC, Horton J, Davis TE, McFadden ET, Carbone PP: Toxicity and response criteria of the Eastern Cooperative Oncology Group. Am J Clin Oncol 5:649-655, 1982.

10.3 APPENDIX 3: RECIST CRITERIA (VERSION 1.1)

Tumor response will be assessed according to RECIST v1.1 (Eisenhauer et al, 2009), as described below.

10.3.1 Measurability of Tumor at Baseline

At baseline, tumor lesions/lymph nodes will be categorized as measurable or nonmeasurable as follows:

- **Measurable**

  Tumor lesions: Must be accurately measured in ≥ 1 dimension (longest diameter in the plane of measurement to be recorded with a minimum size of:

  - 10 mm by CT scan (CT scan slice thickness no greater than 5 mm)
  - 10 mm caliper measurement by clinical examination (lesions that cannot be accurately measured with calipers should be recorded as nonmeasurable)
  - 20 mm by chest X-ray

  Malignant lymph nodes: To be considered pathologically enlarged and measurable, a lymph node must be ≥ 15 mm in short axis when assessed by CT scan (CT scan slice thickness recommended to be no greater than 5 mm).

- **Nonmeasurable**

  - All other lesions (or disease sites), including small lesions (longest diameter < 10 mm or pathological lymph nodes with ≥ 10 to < 15 mm short axis)
  - Lesions considered truly nonmeasurable include the following: leptomeningeal disease, ascites, pleural/pericardial effusion, inflammatory breast disease, lymphangitic involvement of skin or lung, and abdominal masses/abdominal organomegaly identified by physical examination that is not measurable by reproducible imaging techniques

10.3.2 Tumor Response Evaluation

10.3.2.1 Baseline Documentation of Target and Nontarget Lesions

- **Target lesions**

  - When > 1 measurable lesion is present at baseline, all lesions up to a maximum of 5 lesions total (and a maximum of 2 lesions per organ) representative of all involved organs should be identified as target lesions

- It may be the case that, on occasion, the largest lesion that can be measured reproducibly should be selected

- **Nontarget lesions**
  - All other lesions (or disease sites), including pathological lymph nodes, should be identified as nontarget lesions
  - It is possible to record multiple nontarget lesions involving the same organ as a single item (eg, “multiple enlarged pelvic lymph nodes” or “multiple liver metastases”)

10.3.2.2 Evaluation of Target Lesions

Target lesions will be evaluated and response recorded as defined in Appendix Table 1.

Appendix Table 1: Response Based on Evaluation of Target Lesions at Each Assessment

Complete response	Disappearance of all target lesions; if a pathological lymph node, reduction in the shortest axis to < 10 mm [a]
Partial response [b]	≥ 30% decrease in the sum of the diameters of target lesions relative to the baseline sum diameters [c]
Stable disease [b,d]	Neither a sufficient reduction to qualify as a partial response nor a sufficient increase to qualify as progression [c]
Progressive disease [b]	≥ 20% increase in the sum diameters relative to the smallest sum diameters recorded (including the baseline sum diameters) in conjunction with an increase of at least 5 mm in that smallest sum diameters, or the appearance of 1 or more new lesions [c,e]

[a] For each pathological lymph node considered a target lesion, the node must have a short axis measuring < 10 mm to be considered as a complete response. In such cases, the sum diameters may not be zero (a normal lymph node can have a short axis of < 10 mm).
[b] For each pathological lymph node considered a target lesion, the measurement of the short axis of the node is to be included in the sum diameters when determining partial response, stable disease, and progression.
[c] In this study, the “baseline sum diameter” is calculated based on the lesion measurements obtained at screening.
[d] Duration of stable disease is measured from the date of the first dose of study treatment until criteria for progressive disease are met based on the smallest sum diameters recorded (including the baseline sum diameters).
[e] The finding of a new lesion should be unequivocal and not possibly attributable to a difference in imaging modality or scanning technique. Post-baseline, fluorodeoxyglucose positron emission tomography (FDG-PET) may be useful in assessing new lesions apparent on computerized tomography (CT) scan.

10.3.2.3 Evaluation of Nontarget Lesions

Nontarget lesions will be evaluated and response recorded as defined in Appendix Table 2.

Appendix Table 2: Response Based on Evaluation of Nontarget Lesions at Each Assessment

Complete response	Disappearance of all non-target lesions; all lymph nodes must be nonpathological in size (ie, < 10 mm on the short axis)
Not complete response or not progressive disease	Persistence of 1 or more non-target lesions
Progressive disease	Unequivocal progression [a] of any existing non-target lesion or the appearance of 1 or more new lesions [b]

[a] The participant should stop study intervention, even in the presence of a partial response or stable disease, based on assessment of target lesions.

[b] The finding of a new lesion should be unequivocal and not possibly attributable to a difference in imaging modality or scanning technique. Post-baseline, fluorodeoxyglucose positron emission tomography (FDG-PET) may be useful in assessing new lesions apparent on computerized tomography (CT) scan.

10.3.2.4 New Lesions

The appearance of new malignant lesions denotes disease progression; therefore, some comments on detection of new lesions are important. There are no specific criteria for the identification of new radiographic lesions; however, the finding of a new lesion should be unequivocal, ie, not attributable to differences in scanning technique, change in imaging modality, or findings thought to represent something other than tumor (eg, some “new” bone lesions may be simply healing or flare-up of pre-existing lesions). This is particularly important when the participant’s baseline lesions show PR or CR. For example, necrosis of a liver lesion may be reported on a CT scan as a “new” cystic lesion, which it is not.

10.3.3 Evaluation of Overall Response

Overall response based on the evaluation of target and nontarget lesions will be determined as shown in Appendix Table 3.

Appendix Table 3: Evaluation of Overall Response at Each Assessment

Target Lesions	Nontarget Lesions	New Lesions	Overall Response
Complete response	Complete response	No	Complete response
No target lesion[a]	Complete response	No	Complete response
Complete response	Not evaluable[b]	No	Partial response
Complete response	Not complete response/ non-progressive disease	No	Partial response
Partial response	Non-progressive disease and not evaluable[b]	No	Partial response
Stable disease	Non-progressive disease and not evaluable[b]	No	Stable disease

Target Lesions	Nontarget Lesions	New Lesions	Overall Response
Not all evaluated	Non-progressive disease	No	Not evaluable
No target lesion[a]	Not all evaluated	No	Not evaluable
No target lesion[a]	Non-complete response/ non-progressive disease	No	Non-complete response/ non-progressive disease
Progressive disease	Any	Yes or no	Progressive disease
Any	Progressive disease	Yes or no	Progressive disease
Any	Any	Yes	Progressive disease
No target lesion[a]	Unequivocal progressive disease	Yes or No	Progressive disease
No target lesion[a]	Any	Yes	Progressive disease

[a] Defined as no target lesions at baseline.
[b] Not evaluable is defined as either when no or only a subset of lesion measurements are made at an assessment.

10.4 APPENDIX 4: CLINICAL LABORATORY TESTS

- Protocol-specific requirements for inclusion or exclusion of participants are detailed in Section 5 of the protocol.
- Additional tests may be performed at any time during the study as determined necessary by the Investigator or required by local regulations.
- Investigators must document their review of each laboratory safety report.

10.5 APPENDIX 5: ADVERSE EVENTS: DEFINITIONS AND PROCEDURES FOR RECORDING, EVALUATING, FOLLOW-UP, AND REPORTING

10.5.1 Definitions

10.5.1.1 Definition of AE

**AE Definition**
• An AE is any untoward medical occurrence in a participant, temporally associated with the use of study intervention, whether or not considered related to the study intervention.<br>• NOTE: An AE can therefore be any unfavorable and unintended sign (including an abnormal laboratory finding), symptom, or disease (new or exacerbated) temporally associated with the use of a study intervention.

**Events Meeting the AE Definition**
• Any abnormality or deterioration in a laboratory test results (hematology, clinical chemistry, or urinalysis) or other safety assessments (eg, ECG, radiological scans, vital signs measurements), including those that worsen from baseline, or are considered clinically significant in the medical and scientific judgment of the Investigator.<br>• Exacerbation of a chronic or intermittent pre-existing condition including either an increase in frequency and/or intensity of the condition.<br>• A new condition detected or diagnosed after study intervention administration even though it may have been present before the start of the study.<br>• Signs, symptoms, or the clinical sequelae of a suspected drug-drug interaction.<br>• Signs, symptoms, or the clinical sequelae of a suspected overdose of either study intervention or a concomitant medication.<br>• Serious events that are related to a protocol-mandated intervention, including those that occur prior to assignment of study procedures (eg, screening invasive procedures, such as biopsies, discontinuation of study intervention).<br>• Any new cancer (that is not a condition of the study).<br>• Note: Progression of the cancer under study is not a reportable event. Refer to Section 8.3.6 for additional details.

**Events NOT Meeting the AE Definition**
• Medical or surgical procedure (eg, endoscopy, appendectomy): the condition that leads to the procedure is the AE.<br>• Situations in which an untoward medical occurrence did not occur (social and/or convenience admission to a hospital).<br>• Anticipated day-to-day fluctuations of pre-existing disease(s) or condition(s) present or detected at the start of the study that do not worsen.<br>• Surgery planned prior to informed consent to treat a pre-existing condition that has not worsened.

10.5.1.2 Definition of SAE

If an event is not an AE per definition above, then it cannot be an SAE even if serious conditions are met.

**An SAE is defined as any AE that suggests a significant hazard, contraindication, side effect, or untoward medical occurrence that, at any dose:**
**a. Results in death**
**b. Is life-threatening**<br>The term "life-threatening" in the definition of "serious" refers to an event in which the participant was at risk of death at the time of the event. It does not refer to an event, which hypothetically might have caused death, if it were more severe.
**c. Requires inpatient hospitalization or prolongation of existing hospitalization**<br>In general, hospitalization signifies that the participant has been detained at the hospital or emergency ward for observation and/or treatment that would not have been appropriate in the physician's office or outpatient setting. Hospitalization is defined as an inpatient admission, regardless of length of stay, even if the hospitalization is a precautionary measure for continued observation.<br>Note: Hospitalizations for the following reasons are not considered SAEs in this study:<br>• a visit to the emergency room or other hospital department for < 24 hours that does not result in admission (unless considered an important medical or life-threatening event)<br>• elective surgery, planned prior to signing consent<br>• admissions as per protocol for a planned medical/surgical procedure<br>• routine health assessment requiring admission for baseline/trending of health status (eg, routine colonoscopy)

• medical/surgical admission other than to remedy ill health and planned prior to entry into the study. Appropriate documentation is required in these cases<br>• admission encountered for another life circumstance that carries no bearing on health status and requires no medical/surgical intervention (eg, lack of housing, economic inadequacy, caregiver respite, family circumstances, administrative reason).
**d. Results in persistent or significant disability/incapacity**<br>• The term disability means a substantial disruption of a person's ability to conduct normal life functions.<br>• This definition is not intended to include experiences of relatively minor medical significance such as uncomplicated headache, nausea, vomiting, diarrhea, influenza, and accidental trauma (eg, sprained ankle), which may interfere with or prevent everyday life functions but do not constitute a substantial disruption.
**e. Is a congenital anomaly/birth defect**<br>In offspring of a participant exposed to the study intervention regardless of timing as determined for study intervention based on product half-life. Any spontaneous abortion should be reported in the same fashion (as the Sponsor considers spontaneous abortions to be medically significant).
**f. Other important medical events:**<br>• Medical or scientific judgment should be exercised in deciding whether SAE reporting is appropriate in other situations such as important medical events that may not be immediately life-threatening or result in death or hospitalization but may jeopardize the participant or may require medical or surgical intervention to prevent one of the other outcomes listed in the above definition. These events should usually be considered serious.<br>Examples of such events include invasive or malignant cancers, intensive treatment in an emergency room or at home for allergic bronchospasm, blood dyscrasias or convulsions that do not result in hospitalization, or development of drug dependency or drug abuse.

10.5.1.3 Definition of Unexpected AE

Unexpected AE Definition
• Any AE, the specificity or severity of which is not consistent with the current Investigator Brochure. Expected means that the event has previously been observed with the study intervention and is identified and/or described in the current IB. It does not mean that the event is expected with the underlying disease(s), co-morbidities, or concomitant medications.

10.5.1.4 Definition of Treatment-emergent AE

Treatment-emergent AE Definition
• Any unfavorable and unintended diagnosis, symptom, sign (including an abnormal laboratory finding that is considered to be clinically significant), syndrome, or disease that either occurs during the study, having been absent at baseline, or, if present at baseline, appears to have worsened in severity or frequency, whether or not the event is considered related to the study intervention.

10.5.2 Additional Events Reported in the Same Manner as an SAE

Additional Events Reported in the Same Manner as an SAE
• In addition to the SAE criteria in Section 10.5.1.2, AEs meeting any of the below criteria, although not serious per ICH definition, are reportable to the Sponsor in the same timeframe as SAEs to meet certain local requirements. Therefore, these events are considered serious by the Sponsor for collection purposes.<br>o A new cancer (not the indicated condition of the study)<br>o An overdose or is an associated event that meets safety criterion with an overdose (as specified in Section 8.4).<br>o Pregnancy (as specified in Section 8.3.5)

10.5.3 Recording AEs and SAEs

AE and SAE Recording
• When an AE/SAE occurs, it is the responsibility of the Investigator to review all documentation (eg, hospital progress notes, laboratory, and diagnostics reports) related to the event. Only a single AE term should be recorded for the event.<br>• The Investigator will record all relevant AE/SAE information in the eCRF.<br>• It is **not** acceptable for the Investigator to send photocopies of the participant's medical records to the Sponsor in lieu of completion of the Adverse Event/Serious Adverse Event eCRF page.<br>• There may be instances when copies of medical records for certain cases are requested by the Sponsor. In this case, all participant identifiers, with the exception of the participant number, will be redacted on the copies of the medical records before submission to the Sponsor.

• The Investigator will attempt to establish a diagnosis of the event based on signs, symptoms, and/or other clinical information. In such cases, the diagnosis (not the individual signs/symptoms) will be documented as the AE/SAE.
**Assessment of Severity**
• The terms "severe" and "serious" are not synonymous. An event is defined as "serious" when it meets at least 1 of the predefined outcomes as described in the definition of an SAE, NOT when it is rated as severe. Severity (intensity) and seriousness need to be independently assessed for each AE recorded on the eCRF.<br>• The Investigator will assess the intensity for each AE and SAE (and other reportable safety events) according to the NCI CTCAE, version 5.0, which can be found at: http://ctep.cancer.gov/protocolDevelopment/electronic_applications/ctc.htm. The following grading will be used for assessing intensity for AEs not specifically listed in the NCI CTCAE:<br>o Grade 1: Mild; asymptomatic or mild symptoms; clinical or diagnostic observations only; intervention not indicated.<br>o Grade 2: Moderate; minimal, local or noninvasive intervention indicated; limiting age-appropriate instrumental activities of daily living.<br>o Grade 3: Severe or medically significant but not immediately life-threatening; hospitalization or prolongation of hospitalization indicated; disabling; limiting self-care activities of daily living.<br>o Grade 4: Life threatening consequences; urgent intervention indicated.<br>o Grade 5: Death related to AE.<br>• Any AE that changes CTCAE grade over the course of a given episode (ie, persistent AE) will have each change of grade recorded on the Adverse Event eCRF.
**Assessment of Causality**
• The Investigator is obligated to assess the relationship between study intervention and each occurrence of each AE/SAE.<br>• A "reasonable possibility" of a relationship conveys that there are facts, evidence, and/or arguments to suggest a causal relationship, rather than a relationship cannot be ruled out.<br>• The Investigator will use their clinical judgment, knowledge of the participant, the circumstances surrounding the event, and an evaluation of any potential alternative causes to determine the relationship.<br>• The following guidance will be considered and investigated:<br>o Temporal relationship of the event onset to study intervention administration

o Course of the event, with special consideration of the effects of dose reduction, discontinuation of study intervention, or reintroduction of study intervention (as applicable)<br>o Known association of the event with the study intervention or with similar treatments<br>o Known association of the event with the disease under study<br>o Presence of risk factors in the participant or use of concomitant medications known to increase the occurrence of the event<br>o Presence of non-treatment-related factors that are known to be associated with the occurrence of the event<br>• The Investigator will also consult the Investigator's Brochure (IB) and/or Product Information, for marketed products, in his/her assessment.<br>• For each AE/SAE, the Investigator must document in the medical notes that he/she has reviewed the AE/SAE and has provided an assessment of causality.<br>• There may be situations in which an SAE has occurred and the Investigator has minimal information to include in the initial report to the Sponsor. However, it is very important that the Investigator always make an assessment of causality for every event before the initial transmission of the SAE data to the Sponsor.<br>• The Investigator may change his/her opinion of causality in light of follow-up information and send an SAE follow-up report with the updated causality assessment.<br>• The causality assessment is one of the criteria used when determining regulatory reporting requirements.<br>• For studies in which multiple agents are administered as part of a combination regimen, the Investigator may attribute each AE causality to the combination regimen or to a single agent of the combination. In general, causality attribution should be assigned to the combination regimen (ie, to all agents in the regimen). However, causality attribution may be assigned to a single agent if, in the Investigator's opinion, there are sufficient data to support full attribution of the AE to the single agent.
Is the AE suspected to be caused by the study intervention on the basis of facts, evidence, science-based rationales, and clinical judgment?<br>• Yes: There is a plausible temporal relationship between the onset of the AE and administration of the study intervention, and the AE cannot be readily explained by the participant's clinical state, intercurrent illness, or concomitant therapies; and/or the AE follows a known pattern of response to the study intervention; and/or the AE abates or resolves upon discontinuation of the study intervention or dose reduction and, if applicable, reappears upon re-challenge.<br>• No: An AE will be considered related, unless it fulfills the following criteria: Evidence exists that the AE has an etiology other than the study intervention (eg, pre-existing medical condition, underlying disease, intercurrent illness, or concomitant medication);

and/or the AE has no plausible temporal relationship to administration of the study intervention (eg, cancer diagnosed 2 days after first dose of study intervention).

**Follow-up of AEs and SAEs**
• The Investigator is obligated to perform or arrange for the conduct of supplemental measurements and/or evaluations as medically indicated or as requested by the Sponsor to elucidate the nature and/or causality of the AE or SAE as fully as possible. This may include additional laboratory tests or investigations, histopathological examinations, or consultation with other health care professionals.<br>• New or updated information will be recorded on the eCRF.<br>• The Investigator will submit any updated data related to SAEs to the Sponsor within 24 hours of receipt of the information.

10.5.4 Reporting of AEs, SAEs, and Other Reportable Safety Events to the Sponsor

**Reporting of AEs, SAEs, and Other Reportable Safety Events to the Sponsor via an Electronic Data Collection Tool**
• The primary mechanism for reporting AEs to the Sponsor will be via electronic data collection (EDC) tool.<br>• Electronic reporting procedures can be found in the EDC data entry guidelines (or equivalent).<br>• If the electronic system is unavailable for more than 24 hours, then the site will use the paper AE reporting tool (see next section).<br>• Reference Section 8.3.1– Time Period and Frequency for Collecting AE, SAE, and Other Reportable Safety Event Information for reporting time requirements<br>• After the study is completed at a given site, the EDC tool will be taken off-line to prevent the entry of new data or changes to existing data.<br>• The site will report all SAE data to the Parker Institute for Cancer Immunotherapy Pharmacovigilance Group within 24 hours of site awareness.<br>• Contacts for SAE and other reportable serious safety event reporting can be found in Section 8.3.8.2.

SAE and Other Reportable Safety Event Reporting to the Sponsor
• In the instance where an SAE occurs, a facsimile transmission of the SAE Report Form and/or Pregnancy form is the preferred method to transmit this information to the Parker Institute for Cancer Immunotherapy Pharmacovigilance Group.<br>• In rare circumstances and in the absence of facsimile equipment, notification by telephone is acceptable with a copy of the SAE Report Form sent via email.<br>• Initial notification via telephone does not replace the need for the Investigator to complete and sign the SAE Report Form within the designated reporting time frames.<br>• Contacts for SAE and other reportable safety event reporting can be found in Section 8.3.8.2.

10.5.5 Additional Reporting Considerations

AE and SAE Recording for Special Circumstances
**Diagnosis versus Signs and Symptoms**<br>• A diagnosis (if known) or cause of death should be recorded on the Adverse Event eCRF rather than individual signs and symptoms (eg, record only liver failure or hepatitis rather than jaundice, asterixis, and elevated transaminases).<br>• If a constellation of signs and/or symptoms cannot be medically characterized as a single diagnosis or syndrome at the time of reporting, each individual event should be recorded separately on the Adverse Event eCRF page.<br>• If a diagnosis is subsequently established, all previously reported AEs based on signs and symptoms should be nullified and replaced by one AE report based on the single diagnosis, with a starting date that corresponds to the starting date of the first symptom of the eventual diagnosis.
**Adverse Events That Are Secondary to Other Events**<br>• In general, AEs that are secondary to other events (eg, cascade events or clinical sequelae) should be identified by their primary cause, with the exception of severe or serious secondary events. A medically significant secondary AE that is separated in time from the initiating event should be recorded as an independent event on the Adverse Event eCRF page. For example:<br>o If vomiting results in mild dehydration with no additional treatment in a healthy adult, only vomiting should be reported on the eCRF.<br>o If vomiting results in severe dehydration, both events should be reported separately on the eCRF.

o If a severe gastrointestinal hemorrhage leads to renal failure, both events should be reported separately on the eCRF.<br>o If dizziness leads to a fall and consequent fracture, all 3 events should be reported separately on the eCRF.<br>o If neutropenia is accompanied by an infection, both events should be reported separately on the eCRF.<br>• All AEs should be recorded separately on the Adverse Event eCRF page if it is unclear as to whether the events are associated.
**Persistent or Recurrent Adverse Events**<br>• A persistent AE is one that extends continuously, without resolution, between participant evaluation time points. Such events should only be recorded once on the Adverse Event eCRF page. The initial severity (intensity or grade) of the event will be recorded at the time the event is first reported.<br>• If a persistent AE becomes more severe, the most extreme severity should also be recorded on the Adverse Event eCRF page, and details regarding any increases or decreases in severity will be captured on the Adverse Event Grade Changes eCRF.<br>• If the event becomes serious, it should be reported to the Sponsor as an SAE, and the Adverse Event eCRF page should be updated by changing the event from "non-serious" to "serious," providing the date that the event became serious and completing all data fields related to SAEs.<br>• A recurrent AE is one that resolves between participant evaluation time points and subsequently recurs. Each recurrence of an AE should be recorded as a separate event on the Adverse Event eCRF page.
**Abnormal Laboratory Values**<br>• A clinical laboratory test value must be reported as an AE if it meets any of the following criteria:<br>o is accompanied by clinical symptoms<br>o results in a change in study intervention (eg, dose modification, treatment interruption, or treatment discontinuation)<br>o results in a medical intervention or change in concomitant medication<br>o is clinically significant in the Investigator's judgment

Abnormal Vital Sign Values

- Medical and scientific judgment should be exercised in deciding whether an isolated vital sign abnormality should be classified as an AE.
- If a clinically significant vital sign abnormality is a sign of a disease or syndrome (eg, high blood pressure), only the diagnosis (ie, hypertension) should be recorded on the Adverse Event eCRF page.
- Observations of the same clinically significant vital sign abnormality from visit to visit should only be recorded once on the Adverse Event eCRF page (see above for details on recording persistent AEs).

Abnormal Liver Function Tests

- The finding of an elevated ALT or AST (> 3 x baseline value) in combination with either an elevated total bilirubin (> 2 x ULN) or clinical jaundice in the absence of cholestasis or other causes of hyperbilirubinemia is considered to be an indicator of severe liver injury (as defined by Hy's Law). Therefore, Investigators must report as an AE the occurrence of either of the following:
    - Treatment-emergent ALT or AST > 3 x baseline value in combination with total bilirubin > 2 x ULN (of which ≥ 35% is direct bilirubin)
    - Treatment-emergent ALT or AST > 3 x baseline value in combination with clinical jaundice
- The most appropriate diagnosis or, if a diagnosis cannot be established, the abnormal laboratory values should be recorded on the Adverse Event eCRF page and reported to the Sponsor immediately as an SAE.

Lack of Efficacy or Worsening of Underlying Disease

- Events that are clearly consistent with the expected pattern of progression of the underlying disease should not be recorded as AEs. These data will be captured as efficacy assessment data only. In most cases, the expected pattern of progression will be based on RECIST. In rare cases, the determination of clinical progression will be based on symptomatic deterioration. However, every effort should be made to document progression through use of objective criteria. If there is any uncertainty as to whether an event is due to disease progression, it should be reported as an AE.

Deaths

- All deaths that occur during the protocol-specified AE reporting period, regardless of relationship to study intervention, must be recorded on the Death eCRF page and immediately reported to the Sponsor (see Section 8.3.1), unless the death is attributed to progression of disease.
- Death should be considered an outcome and not a distinct event. The event or condition that caused or contributed to the fatal outcome should be recorded as the single medical

concept on the Adverse Event eCRF page. Generally, only one such event should be reported.<br>• If the cause of death is unknown and cannot be ascertained at the time of reporting, “unexplained death” should be recorded on the Adverse Event eCRF page.<br>• If the cause of death later becomes available (eg, after autopsy), “unexplained death” should be replaced by the established cause of death.<br>• The term “sudden death” should not be used unless combined with the presumed cause of death (eg, “sudden cardiac death”).<br>• If the death is attributed to progression of underlying disease, “underlying disease" should be recorded on the Death eCRF page; no SAE form is necessary.<br>• Deaths that occur after the AE reporting period should be reported as described in Section 8.3.2.
**Pre-existing Medical Conditions**<br>• A pre-existing medical condition is one that is present at the baseline visit for this study. Such conditions should be recorded on the General Medical History and Baseline Conditions eCRF page.<br>• A pre-existing medical condition should be recorded as an AE only if the frequency, severity, or character of the condition worsens during the study. When recording such events on the Adverse Event eCRF page, it is important to convey the concept that the pre-existing condition has changed by including applicable descriptors (eg, “more frequent headaches”).
**Adverse Events Associated with Overdose or Error in Drug Administration**<br>• An overdose is the accidental or intentional use of a drug in an amount higher than the dose being studied. An overdose or incorrect administration of study intervention is not itself an AE, but it may result in an AE. All AEs associated with an overdose or incorrect administration of study intervention should be recorded on the Adverse Event eCRF page.<br>• If the associated AE fulfills seriousness criteria, the event should be reported to the Sponsor as a separate SAE.

10.6 APPENDIX 6: CONTRACEPTIVE GUIDANCE AND COLLECTION OF PREGNANCY INFORMATION

10.6.1 Definitions

10.6.1.1 Woman of Childbearing Potential (WOCBP)

A woman is considered fertile following menarche and until becoming post-menopausal unless permanently sterile (see below).

Women in the following categories **are not considered WOCBP**

1. Premenarchal
2. Premenopausal female with 1 of the following:
    - Documented hysterectomy
    - Documented bilateral salpingectomy
    - Documented bilateral oophorectomy

    Note: Documentation can come from the site personnel's review of the participant's medical records, medical examination, or medical history interview.
3. Postmenopausal female
    - A postmenopausal state is defined as no menses for 12 months without an alternative medical cause. A high follicle-stimulating hormone level in the postmenopausal range may be used to confirm a postmenopausal state in women not using hormonal contraception or HRT. However, in the absence of 12 months of amenorrhea, a single follicle-stimulating hormone measurement is insufficient.
    - Females on HRT and whose menopausal status is in doubt will be required to use one of the non-estrogen hormonal highly effective contraception methods if they wish to continue their HRT during the study. Otherwise, they must discontinue HRT to allow confirmation of postmenopausal status before study enrollment.

10.6.2 Contraception Guidance

10.6.2.1 Male Participants

Male participants with female partners of childbearing potential are eligible to participate if they agree to ONE of the following:

- Are abstinent from penile-vaginal intercourse as their usual and preferred lifestyle (abstinent on a long-term and persistent basis) and agree to remain abstinent
- Agree to use a male condom plus partner use of a contraceptive method with a failure rate of < 1% per year as described in Appendix Table 4 when having penile-vaginal intercourse with a WOCBP who is not currently pregnant.

In addition, male participants must refrain from donating sperm for the duration of the study and for 7 months after the last dose of study intervention.

Male participants with a pregnant or breastfeeding partner must agree to remain abstinent from penile vaginal intercourse or use a male condom during each episode of penile penetration during the protocol-defined time frame and for 7 months after the last dose of study intervention.

10.6.2.2 Female Participants

Female participants of childbearing potential are eligible to participate if they agree to use a highly effective method of contraception consistently and correctly as described in Appendix Table 4.

Appendix Table 4: Highly Effective Contraceptive Methods

**Highly Effective Contraceptive Methods That Are User Dependent** [a]<br>*Failure rate of < 1% per year when used consistently and correctly.*
Combined (estrogen and progestogen containing) hormonal contraception associated with inhibition of ovulation[b]<br>• Oral<br>• Intravaginal<br>• Transdermal
Progestogen only hormonal contraception associated with inhibition of ovulation<br>• Oral<br>• Injectable
**Highly Effective Methods That Are User Independent**[a]
Implantable progestogen-only hormonal contraception associated with inhibition of ovulation[b]<br>• Intrauterine device (IUD)<br>• Intrauterine hormone-releasing system (IUS)<br>• Bilateral tubal occlusion
**Vasectomized partner**<br>A vasectomized partner is a highly effective contraception method provided that the partner is the sole male sexual partner of the WOCBP and the absence of sperm has been confirmed. If not, an additional highly effective method of contraception should be used.
**Sexual abstinence**

Sexual abstinence is considered a highly effective method only if defined as refraining from heterosexual intercourse during the entire period of risk associated with the study intervention. The reliability of sexual abstinence needs to be evaluated in relation to the duration of the study and the preferred and usual lifestyle of the participant.
NOTES:<br>a) Typical use failure rates may differ from those when used consistently and correctly. Use should be consistent with local regulations regarding the use of contraceptive methods for participants participating in clinical studies.<br>b) Hormonal contraception may be susceptible to interaction with the study intervention, which may reduce the efficacy of the contraceptive method. In this case, 2 highly effective methods of contraception should be utilized during the treatment period and for at least 5 months after the last dose of study intervention.

10.6.3 Pregnancy Testing

- WOCBP should only be included after a confirmed menstrual period and a negative highly sensitive urine or serum pregnancy test within 24 hours of first dose.
- Additional pregnancy testing should be performed every cycle during the treatment period and within 24 hours prior to dosing.
- Pregnancy testing will be performed whenever a menstrual cycle is missed or when pregnancy is otherwise suspected.

10.6.4 Collection of Pregnancy Information

10.6.4.1 Male Participants with Partners who Become Pregnant

- The Investigator will attempt to collect pregnancy information on any male participant's female partner who becomes pregnant while the male participant is in this study. This applies only to male participants who receive study intervention.
- After obtaining the necessary signed informed consent from the pregnant female partner directly, the Investigator will record pregnancy information on the appropriate form and submit it to the Sponsor within 24 hours of learning of the partner's pregnancy. The female partner will also be followed to determine the outcome of the pregnancy. Information on the status of the mother and child will be forwarded to the Sponsor. Generally, the follow-up will be no longer than 6 to 8 weeks following the estimated delivery date. Any termination of the pregnancy will be reported regardless of fetal status (presence or absence of anomalies) or indication for the procedure.

10.6.4.2 Female Participants who Become Pregnant

- The Investigator will collect pregnancy information on any female participant who becomes pregnant while participating in this study. Information will be recorded on the appropriate form and submitted to the Sponsor within 24 hours of learning of a participant's pregnancy. The participant will be followed to determine the outcome of

the pregnancy. The Investigator will collect follow-up information on the participant and the neonate and the information will be forwarded to the Sponsor. Generally, follow-up will not be required for longer than 6 to 8 weeks beyond the estimated delivery date. Any termination of pregnancy will be reported, regardless of fetal status (presence or absence of anomalies) or indication for the procedure.

- While pregnancy itself is not considered to be an AE or SAE, any pregnancy complication or elective termination of a pregnancy will be reported as an AE or SAE. A spontaneous abortion is always considered to be an SAE and will be reported as such. Any post-study pregnancy-related SAE considered reasonably related to the study intervention by the Investigator will be reported to the Sponsor as described in Section 8.3.5. While the Investigator is not obligated to actively seek this information in former study participants, he or she may learn of an SAE through spontaneous reporting.
- Any female participant who becomes pregnant while participating in the study will discontinue study intervention.

10.7 APPENDIX 7: GENETICS

Use/Analysis of DNA

- Genetic variation may impact a participant's response to study intervention, susceptibility to, and severity and progression of disease. Variable response to study intervention may be due to genetic determinants that impact immune response, drug absorption, distribution, metabolism, and excretion; mechanism of action of the drug; disease etiology; and/or molecular subtype of the disease being treated. Therefore, where local regulations and IRB/IEC allow, a blood and tumor sample will be collected for DNA analysis from consenting participants.
- DNA samples will be used for research related to checkpoint inhibitor therapy or cancer and/or immune disorders and related diseases or evaluation of new assay technologies. They may also be used to develop tests/assays including diagnostic tests related to checkpoint inhibitor therapies and cancer. Genetic research may consist of the analysis of one or more candidate genes or the analysis of genetic markers throughout the genome or analysis of the entire genome (as appropriate).
- DNA samples may be analyzed for whole genome sequencing and relationship to clinical outcomes in response to study intervention. Additional analyses may be conducted if it is hypothesized that this may help further understand the clinical data.
- The samples may be analyzed as part of a multi-study assessment of genetic factors involved in the response to checkpoint inhibitor therapy or study interventions of this class to understand study disease or related conditions.
- The results of genetic analyses may be reported in the clinical study report or in a separate study summary.
- The Sponsor will store the DNA samples in a secure storage space with adequate measures to protect confidentiality.
- The samples will be retained while research on checkpoint inhibitor therapy continues in accordance with applicable law.

10.8 APPENDIX 8: MANAGEMENT ALGORITHMS

GI Adverse Event Management Algorithm

Rule out non-inflammatory causes. If non-inflammatory cause is identified, treat accordingly and continue I-O therapy.
Opiates/narcotics may mask symptoms of perforation. Infliximab should not be used in cases of perforation or sepsis.

Grade of Diarrhea/ Colitis (NCI CTCAE v5)	Management	Follow-up
**Grade 1**<br>Diarrhea: < 4 stools/day over baseline<br>Colitis: asymptomatic	• Continue I-O therapy per protocol<br>• Symptomatic treatment	• Close monitoring for worsening symptoms<br>• Educate patient to report worsening immediately<br><br>If worsens:<br>• Treat as Grade 2 or Grade 3-4
**Grade 2**<br>Diarrhea: 4-6 stools per day over baseline;<br>IV fluids indicated < 24 hrs;<br>Limiting instrumental ADL<br>Colitis: abdominal pain; blood in stool	• Delay I-O therapy per protocol<br>• Symptomatic treatment	If improves to Grade 1:<br>• Resume I-O therapy per protocol<br>If persists > 5-7 days or recurs:<br>• 0.5-1 mg/kg/day methylprednisolone or oral equivalent<br>• When symptoms improve to Grade 1, taper steroids over at least 1 month, consider prophylactic antibiotics for opportunistic infections, and resume I-O therapy per protocol.<br>If worsens or persists > 3-5 days with oral steroids:<br>• Treat as Grade 3-4
**Grade 3-4**<br>Diarrhea (G3): ≥ 7 stools per day over baseline;<br>IV fluids ≥24 hrs;<br>limiting self care ADL<br>Colitis (G3): severe abdominal pain, peritoneal signs<br>G4: life-threatening, perforation	• Discontinue or delay I-O therapy per protocol*<br>• 1 to 2 mg/kg/day methylprednisolone IV or IV equivalent<br>• Add prophylactic antibiotics for opportunistic infections<br>• Consider lower endoscopy	If improves:<br>• Continue steroids until grade 1, then taper over at least 1 month<br>If persists > 3-5 days, or recurs after improvement:<br>• Add infliximab 5 mg/kg (if no contraindication) or other immunosuppressants.<br>• Consider testing CMV for steroid refractory colitis<br>Note: Infliximab should not be used in cases of perforation or sepsis

Patients on IV steroids may be switched to an equivalent dose of oral corticosteroids (eg, prednisone) at start of tapering or earlier, after sustained clinical improvement is observed. Lower bioavailability of oral corticosteroids should be taken into account when switching to the equivalent dose of oral corticosteroids.

* Discontinue for Grade 4 diarrhea or colitis. For Grade 3 diarrhea or colitis, 1) Nivolumab monotherapy: Nivolumab can be delayed. 2) Nivolumab+ Ipilimumab combination: Ipilimumab should be discontinued while nivolumab can be delayed. Nivolumab monotherapy can be resumed when symptoms improve to Grade 1. Please refer to protocol for dose delay and discontinue criteria for other combinations.

Hepatic Adverse Event Management Algorithm

Rule out non-inflammatory causes. If non-inflammatory cause, treat accordingly and continue I-O therapy.
Consider imaging for obstruction.

Liver Function Test	Management	Follow-up
AST or ALT > ULN to 3.0 x ULN<br>and/or<br>T. bili > ULN to 1.5 x ULN	• Continue I-O therapy per protocol<br>• Consider increasing frequency of monitoring	• Continue LFT monitoring per protocol<br><br>If worsens:<br>• Treat as below
AST or ALT > 3.0 to ≤ 5 x ULN<br>and/or<br>T. bili > 1.5 to ≤ 3 x ULN	• Delay I-O therapy per protocol<br>• Increase frequency of monitoring to every 3 days	If returns to baseline:<br>•Resume routine monitoring, resume I-O therapy per protocol<br><br>If elevations persist > 5-7 days or worsen:<br>• 0.5-1 mg/kg/day methylprednisolone or oral equivalent and when and AST ALT returns to <3X ULN and bili <1.5X ULN, taper steroids over at least 1 month, consider prophylactic antibiotics for opportunistic infections, and resume I-O therapy per protocol
AST or ALT > 5 x ULN<br>or<br>T.bili >3 x ULN	• Delay or Discontinue I-O therapy per protocol^<br>• Increase frequency of monitoring to every 1-2 days<br>• 1 to 2 mg/kg/day methylprednisolone IV or IV equivalent*<br>• Add prophylactic antibiotics for opportunistic infections<br>• Consult gastroenterologist or hepatologist	If AST and ALT returns to <5X ULN and bili <3X ULN,<br>• Taper steroids over at least 1 month<br><br>If does not improve in >3-5 days, worsens, or rebounds:<br>• Add mycophenolate mofetil 1 g BID<br>• If no response within an additional 3-5 days, consider tacrolimus or other immunosuppressants per local guidelines<br>Note: Avoid infliximab due to potential risk of liver failure

Patients on IV steroids may be switched to an equivalent dose of oral corticosteroids (e.g. prednisone) at start of tapering or earlier, after sustained clinical improvement is observed. Lower bioavailability of oral corticosteroids should be taken into account when switching to the equivalent dose of oral corticosteroids.

^ Please refer to protocol dose delay and discontinue criteria for specific details.

*The recommended starting dose for AST or ALT > 20 x ULN or bilirubin >10 x ULN is 2 mg/kg/day methylprednisolone IV.

Endocrinopathy Adverse Event Management Algorithm

Rule out non-inflammatory causes. If non-inflammatory cause, treat accordingly and continue I-O therapy.
Consider visual field testing, endocrinology consultation, and imaging.

Endocrinopathy	Management	Follow-up
Asymptomatic TSH abnormality<br>or<br>Hyperglycemia requiring no medical intervention	• Continue I-O therapy per protocol<br>• If TSH < 0.5 x LLN, or TSH > 2 x ULN, or consistently out of range in 2 subsequent measurements:<br>o include fT4 at subsequent cycles as clinically indicated;<br>o consider endocrinology consult	• Monitor TSH as per protocol<br>• Monitor blood sugar<br>• If symptomatic or blood sugar requiring initiation of treatment or change in management, manage as below
Symptomatic endocrinopathy<br>or<br>hyperglycemia requiring initiation of treatment or change in daily management or work up	• Evaluate endocrine function; consider endocrinology consult<br>• Consider pituitary scan<br>Symptomatic with abnormal lab/pituitary scan:<br>• Delay I-O therapy per protocol<br>• 1-2 mg/kg/day methylprednisolone IV or PO equivalent for hypophysitis<br>• Initiate appropriate hormone/medical therapy<br>No abnormal lab/pituitary MRI scan but symptoms persist:<br>• Continue IO therapy. Repeat labs in 1-3 weeks /MRI in 1 month<br>Hyperglycemia:<br>Screen for Diabetic Ketoacidosis (DKA). If negative, continue IO and treat diabetes. If DKA hold IO, admit, manage DKA. Steroids not recommended for DKA/hyperglycemia	If improves (with or without hormone replacement):<br>• Taper steroids over at least 1 month and consider prophylactic antibiotics for opportunistic infections<br>• Resume I-O therapy per protocol<br>• Patients with adrenal insufficiency may need to continue steroids with mineralocorticoid component
Suspicion of adrenal crisis (eg, severe dehydration, hypotension, shock out of proportion to current illness)	• Delay or discontinue I-O therapy per protocol<br>• Rule out sepsis<br>• Stress dose of IV steroids with mineralocorticoid activity<br>• IV fluids<br>• Consult endocrinologist	• Patients with adrenal insufficiency may need to continue steroids with mineralocorticoid component<br>• If adrenal crisis resolves and patient is adequately controlled with physiologic hormone replacement, patient can resume IO therapy per protocol.

Patients on IV steroids may be switched to an equivalent dose of oral corticosteroids (eg, prednisone) at start of tapering or earlier, after sustained clinical improvement is observed. Lower bioavailability of oral corticosteroids should be taken into account when switching to the equivalent dose of oral corticosteroids.

Skin Adverse Event Management Algorithm

Rule out non-inflammatory causes. If non-inflammatory cause, treat accordingly and continue I-O therapy.

Rash	Management	Follow-up
Covering ≤ 30% BSA*	• Symptomatic therapy (eg, antihistamines, topical steroids)<br>• Continue I-O therapy per protocol	<u>If persists > 1-2 weeks or recurs</u>:<br>• Consider skin biopsy<br>• Delay I-O therapy per protocol<br>• Consider 0.5-1.0 mg/kg/day methylprednisolone IV or oral equivalent. Once improving , taper steroids over at least 1 month, consider prophylactic antibiotics for opportunistic infections, and resume I-O therapy per protocol<br><u>If worsens</u>:<br>• Treat as >30% BSA
Covering > 30% BSA (with or without symptoms)<br><br>Life threatening consequences*^	• Delay or discontinue I-O therapy per protocol<br>• Consider skin biopsy<br>• Dermatology consult<br>• 1.0 -2.0 mg/kg/day IV methylprednisolone IV or oral equivalent	<u>If improves to < 10% BSA:</u><br>• Taper steroids over at least 1 month and add prophylactic antibiotics for opportunistic infections<br>• Resume I-O therapy per protocol

Patients on IV steroids may be switched to an equivalent dose of oral corticosteroids (e.g. prednisone) at start of tapering or earlier, after sustained clinical improvement is observed. Lower bioavailability of oral corticosteroids should be taken into account when switching to the equivalent dose of oral corticosteroids.

*Refer to NCI CTCAE v5 for term-specific grading criteria.

^If Steven-Johnson Syndrome (SJS),toxic epidermal necrosis (TEN), Drug Reaction with Eosinophilia and Systemic Symptoms (DRESS) is suspected, withhold I-O therapy and refer patient for specialized care for assessment and treatment. If SJS, TEN, or DRESS is diagnosed, permanently discontinue I-O therapy.

Neurological Adverse Event Management Algorithm

Rule out non-inflammatory causes. If non-inflammatory cause, treat accordingly and continue I-O therapy.

Grade of Neurological Toxicity (NCI CTCAE v5)	Management	Follow-up
**Grade 1**<br>Asymptomatic or mild symptoms;<br>Intervention not indicated	• Continue I-O therapy per protocol<br>• Discontinue I-O for select AEs^	• Continue to monitor per protocol.<br><u>If worsens:</u><br>• Treat as Grade 2 or Grade 3-4
**Grade 2**<br>Moderate symptoms;<br>Limiting instrumental ADL	• Delay I-O therapy per protocol<br>• Discontinue I-O for select AEs^<br>• Consider neurology consult<br>• Treat symptoms per local guidelines<br>• Consider 0.5 to 1 mg/kg/day methylprednisolone IV or PO equivalent	<u>If returns to baseline</u>:<br>• Resume I-O therapy per protocol when improved to baseline If worsens:<br>• Treat as Grade 3-4
**Grade 3-4**<br>Severe symptoms;<br>Limiting self-care ADL;<br>Life-threatening	• Discontinue I-O therapy per protocol<br>• Obtain neurology consult<br>• Treat symptoms per local guidelines<br>• 1-2 mg/kg/day IV methylprednisolone IV or IV equivalent<br>• Add prophylactic antibiotics for opportunistic infections	<u>If improves to Grade 2</u>:<br>• Taper steroids over at least 1 month<br><u>If worsens or atypical presentation</u>:<br>• Consider IVIG or other immunosuppressive therapies per local guidelines

Patients on IV steroids may be switched to an equivalent dose of oral corticosteroids (eg. prednisone) at start of tapering or earlier, after sustained clinical improvement is observed. Lower bioavailability of oral corticosteroids should be taken into account when switching to the equivalent dose of oral corticosteroids.

^Discontinue for any grade myasthenia gravis, Guillain-Barre syndrome, treatment-related myelitis, or encephalitis.

Pulmonary Adverse Event Management Algorithm

Rule out non-inflammatory causes. If non-inflammatory cause, treat accordingly and continue I-O therapy.
Evaluate with imaging and pulmonary consultation.

Grade of Pneumonitis (NCI CTCAE v5)	Management	Follow-up
**Grade 1**<br>Radiographic changes only	• Consider delay of I-O therapy<br>• Monitor for symptoms every 2-3 days<br>• Consider Pulmonary and ID consults	• Re-image at least every 3 weeks<br><br>If worsens:<br>• Treat as Grade 2 or 3-4
**Grade 2**<br>Mild to moderate new symptoms	• Delay I-O therapy per protocol<br>• Pulmonary and ID consults<br>• Monitor symptoms daily, consider hospitalization<br>• 1 mg/kg/day methyl prednisolone IV or oral equivalent<br>• Consider bronchoscopy, lung biopsy	If improves to Grade 1:<br>• Resume I-O therapy per protocol<br>If persists > 5-7 days or recur:<br>• 0.5-1 mg/kg/day methylprednisolone or oral equivalent<br>• When symptoms improve to Grade 1, taper steroids over at least 1 month, consider prophylactic antibiotics for opportunistic infections, and resume I-O therapy per protocol.<br>If worsens or persists > 3-5 days with oral steroids:<br>• Treat as Grade 3-4
**Grade 3-4**<br>Severe new symptoms; New/worsening hypoxia; Life-threatening	• Discontinue I-O therapy per protocol<br>• Hospitalize<br>• Pulmonary and ID consults<br>• 2-4 mg/kg/day methyl prednisolone IV or IV equivalent<br>• Add prophylactic antibiotics for opportunistic infections<br>• Consider bronchoscopy, lung biopsy	If improves to baseline:<br>• Taper steroids over at least 6 weeks<br><br>If not improving after 48 hours or worsening:<br>• Add additional immunosuppression

Patients on IV steroids may be switched to an equivalent dose of oral corticosteroids (eg, prednisone) at start of tapering or earlier, after sustained clinical improvement is observed. Lower bioavailability of oral corticosteroids should be taken into account when switching to the equivalent dose of oral corticosteroids.

Renal Adverse Event Management Algorithm

Rule out non-inflammatory causes. If non-inflammatory cause, treat accordingly and continue I-O therapy.

Grade of Creatinine Elevation (NCI CTCAE v5)	Management	Follow-up
**Grade 1**<br>Creatinine > ULN to ≤ 1.5x ULN	• Continue I-O therapy per protocol<br>• Monitor creatinine weekly	If returns to baseline:<br>•Resume routine creatinine monitoring per protocol<br>If worsens:<br>• Treat as Grade 2 to Grade 4
**Grade 2-3**<br>Creatinine > 1.5x baseline to ≤ 6x ULN	• Delay I-O therapy per protocol<br>• Monitor creatinine every 2-3 days<br>• 0.5 to 1 mg/kg/day methylprednisolone IV or oral equivalent<br>• Consider renal biopsy with nephrology consult	If returns to Grade 1:<br>•Taper steroids over at least 1 month, consider prophylactic antibiotics for opportunistic infections, and resume I-O therapy and routine creatinine monitoring per protocol<br>If elevations persist > 7 days or worsen:<br>•Treat as Grade 4
**Grade 4**<br>Creatinine > 6x ULN	• Discontinue I-O therapy per protocol<br>• Monitor creatinine daily<br>• 1-2 mg/kg/day methylprednisolone IV or IV equivalent<br>• Consult nephrologist<br>• Consider renal biopsy	If returns to Grade 1: Taper steroids over at least 1 month and add prophylactic antibiotics for opportunistic infections<br><br>If no improvement or worsening, add additional immunosuppression.

Patients on IV steroids may be switched to an equivalent dose of oral corticosteroids (eg, prednisone) at start of tapering or earlier, after sustained clinical improvement is observed. Lower bioavailability of oral corticosteroids should be taken into account when switching to the equivalent dose of oral corticosteroids.

Myocarditis Adverse Event Management Algorithm

Rule out non-inflammatory causes. If non-inflammatory cause, treat accordingly and continue I-O therapy.

Myocarditis	Management	Follow-up
Symptoms with mild to moderate activity or exertion	• Delay I-O therapy; hospitalization with cardiac monitoring<br>• Urgent cardiology consultation for evaluation and management<br>o Troponin and BNP<br>o ECG ± continuous cardiac monitoring<br>o Echocardiogram<br>o Cardiac MRI<br>• Prompt initiation of 2 mg/kg/day methylprednisolone IV or equivalent	• If worsens, intensify treatment according to grade<br>• Upon recovery, taper steroids over at least 1 month with close monitoring of troponin and BNP. as well as for new symptoms<br>• Repeat cardiac MRI for post treatment assessment and cardiology follow-up<br>• Retreatment may be considered after recovery and completion of steroid taper
Severe with symptoms at rest or with minimal activity or exertion; intervention indicated<br><br>Life-threatening consequences; urgent intervention indicated (e.g., continuous IV therapy or mechanical hemodynamic support)	• Permanently discontinue I-O therapy<br>• Hospitalize to intensive cardiac monitoring<br>• Cardiac evaluation to include:<br>o Troponin and B-type natriuretic peptide monitoring<br>o ECG ± continuous cardiac monitoring<br>o Echocardiogram<br>o Cardiac MRI<br>o Myocardial biopsy if feasible<br>• Immediate initiation of 2 mg/kg/day methylprednisolone IV or 1 g IV bolus<br>• Consider adding a second immunosuppressive agent<br>Additionally, for life threatening myocarditis:<br>• Hospitalize/transfer to institution with expertise in intensive cardiac monitoring<br>• Consider anti-thymocyte globulin as second agent given its immediate effect	• If no improvement, consider additional immunosuppression<br>• Upon recovery, taper steroids over at least 1 month with close monitoring of troponin and BNP as well as for new symptoms<br>• Repeat cardiac MRI for post treatment assessments and cardiology follow-up

Patients on IV steroids may be switched to an equivalent dose of oral corticosteroids (eg, prednisone) at start of tapering or earlier, after sustained clinical improvement is observed. Lower bioavailability of oral corticosteroids should be taken into account when switching to the equivalent dose of oral corticosteroids.
Prophylactic antibiotics should be considered in the setting of ongoing immunosuppression.

10.9 APPENDIX 9: LIST OF ABBREVIATIONS

Abbreviation	Definition
AE	adverse event
ALT	alanine aminotransferase
APC	advanced prostate cancer
AST	aspartate aminotransferase
CAP	College of American Pathologists
C_{avgss}	time-averaged steady-state concentration
CBR	clinical benefit rate
CLIA	Clinical Laboratory Improvement Amendment
C_{maxss}	maximum steady-state concentration
C_{minss}	minimum steady-state concentration
COA	clinical outcome assessment
CR	complete response
CRF	case report form
CT	computed tomography
CTCAE	Common Terminology Criteria for Adverse Events
CTLA-4	cytotoxic T-lymphocyte-associated antigen-4
DNA	deoxyribonucleic acid
DRESS	drug reaction with eosinophilia and systemic symptoms
ECG	electrocardiogram
ECOG	Eastern Cooperative Oncology Group
eCRF	electronic case report form
EDC	electronic data collection
FDA	Food and Drug Administration
FFPE	formalin-fixed paraffin-embedded
FT3	free triiodothyronine
FT4	free thyroxine
GBS	Guillain-Barre Syndrome
GCP	Good Clinical Practice
HCC	hepatocellular carcinoma
HIPAA	Health Insurance Portability and Accountability Act
HRT	hormone replacement therapy
IB	Investigator's Brochure
ICF	informed consent form

Abbreviation	Definition
ICH	International Council for Harmonisation
IEC	Independent Ethics Committee
IHC	immunohistochemistry
irAE	immune-related adverse event
IRB	Institutional Review Board
IV	intravenous(ly)
mAb	monoclonal antibody
mCRPC	metastatic castration-resistant prostate cancer
MDACC	MD Anderson Cancer Center
MG	Myasthenia Gravis
mITT	modified Intent-to-Treat
MRI	magnetic resonance imaging
MSI	microsatellite instable
MSS	microsatellite stable
MTD	maximum tolerated dose
NAF	sodium fluoride
NSCLC	non-small cell lung cancer
ORR	objective response rate
OS	overall survival
PBMC	peripheral blood mononuclear cell
PCWG3	Prostate Cancer Clinical Trials Working Group 3
PD	progressive disease
PD-1	programmed cell death-1
PD-L1	programmed cell death ligand-1
PET	positron emission tomography
PFS	progression-free survival
PI	Principal Investigator
PK	pharmacokinetic(s)
PPK	population pharmacokinetic
PR	partial response
PRO	patient-reported outcomes
Q2W	every 2 weeks
Q3W	every 3 weeks
Q4W	every 4 weeks

Abbreviation	Definition
RCC	renal cell carcinoma
RECIST v1.1	Response Evaluation Criteria in Solid Tumors version 1.1
RNA	ribonucleic acid
SAE	serious adverse event
SCCHN	squamous cell carcinoma of the head and neck
SD	stable disease
SOA	Schedule of Activities
T.bili	total bilirubin
TC-99m	technetium-99m
TOX	trial-limiting toxicity
TRAE(s)	treatment-related adverse event(s)
TSH	thyroid stimulating hormone
ULN	upper limit of normal
USPI	US Prescribing Information
WOCBP	woman of childbearing potential

10.10 APPENDIX 10: PROTOCOL AMENDMENT HISTORY

The Protocol Amendment Summary of Changes Table for the current amendment is located directly before the Table of Contents (TOC).

DOCUMENT HISTORY	
**Documents**	**Date of Issue**
Amendment 4	12 Mar 2021
Amendment 3	08 Jul 2020
Amendment 2	12 Jun 2019
Amendment 1	27 July 2018
Original Protocol	06 Apr 2018

11 REFERENCES

Ayers M, Lunceford J, Nebozhyn M, Murphy E, Loboda A, Kaufman DR, et al. IFN-γ-related mRNA profile predicts clinical response to PD-1 blockade. J Clin Invest. 2017;127:2930-40.

Beer TM, Kwon ED, Drake CG, et al. Randomized, Double-Blind, Phase III Trial of Ipilimumab Versus Placebo in Asymptomatic or Minimally Symptomatic Patients With Metastatic Chemotherapy-Naive Castration-Resistant Prostate Cancer. J Clin Oncol. 2017;35(1):40-47.

Bristol-Myers Squibb (BMS). Nivolumab Studies. Available at: https://www.bmsstudyconnect.com/us/en/health-studies/

Borghaei H, Paz-Ares L, Horn L, Spigel DR, Steins M, Ready NE, et al. Nivolumab versus Docetaxel in Advanced Nonsquamous Non-Small-Cell Lung Cancer. N Engl J Med. 2015;373:1627–39.

Brahmer J, Reckamp KL, Baas P, Crino L, Eberhardt WE, Poddubskaya E, et al. Nivolumab versus Docetaxel in Advanced Squamous-Cell Non-Small-Cell Lung Cancer. N Engl J Med. 2015;373:123–35.

Callahan MK, Kluger H, Postow MA, Segal NH, Lesokhin A, Atkins MB, et al. Nivolumab Plus Ipilimumab in Patients With Advanced Melanoma: Updated Survival, Response, and Safety Data in a Phase I Dose-Escalation Study. J Clin Oncol 2018;36(4):391–8.

Chen PL, Roh W, Reuben A, Cooper ZA, Spencer CN, Prieto PA, et al. Analysis of immune signatures in longitudinal tumor samples yields insight into biomarkers of response and mechanisms of resistance to immune checkpoint blockade. Cancer Discov. 2016;6:827-37.

Concha-Benavente F, Gillison ML, Blumenschein GR, Harrington K, Fayette J, et al. Characterization of potential predictive biomarkers of response to nivolumab in CheckMate 141 in patients with squamous cell carcinoma of the head and neck (SCCHN). J Clin Oncol. 2017;35(15_suppl):6050 (abstract 6050).

Drake CG, Kwon ED, Fizazi K, et al. Results of subset analyses on overall survival (OS) from study CA184-043: Ipilimumab (Ipi) versus placebo (Pbo) in post-docetaxel metastatic castration-resistant prostate cancer (mCRPC). JCO. 2014;32(4_suppl):2-2.

Eisenhauer EA, Therasse P, Bogaerts J, et al. New response evaluation criteria in solid tumours: revised RECIST guidelines (version 1.1). Eur J Cancer. 2009;45:228-47.

Feldmeyer L, Hudgens CW, Ray-Lyons G, Nagarajan P, Aung PP, Curry JL, et al. Density, distribution, and composition of immune infiltrates correlate with survival in Merkel cell carcinoma. Clin Cancer Res. 2016;22:5553-63.

Gao J, Ward JF, Pettaway CA, Shi LZ, Subudhi SK, Vence LM, et al. VISTA is an inhibitory immune checkpoint that is increased after ipilimumab therapy in patients with prostate cancer. Nat Med. 2017;23:551-7.

Gopalakrishnan V, Spencer CN, Nezi L, Reuben A, Andrews MC, Karpinets TV, et al. Gut microbiome modulates response to anti-PD-1 immunotherapy in melanoma patients. Science. 2018;359:97-103.

Haanen JBAG. Converting cold into hot tumors by combining immunotherapies. Cell. 2017;170:1055-6.

Hegde PS, Karanikas V, Evers S. The where, the when, and the how of immune monitoring for cancer immunotherapies in the era of checkpoint inhibition. Clin Cancer Res. 2016;22:1865-74.

Hellmann MD, Paz-Ares L, Bernabe Caro R, Zurawski B, Kim S-W, Carcereny Costa E, et al. Nivolumab plus Ipilimumab in Advanced Non–Small-Cell Lung Cancer. New England Journal of Medicine 2019;381(21):2020–31.

Herbst RS, Baas P, Kim DW, Felip E, Perez-Gracia JL, Han JY, et al. Pembrolizumab versus docetaxel for previously treated, PD-L1-positive, advanced non-small-cell lung cancer (KEYNOTE-010): a randomised controlled trial. Lancet. 2016;387:1540-50.

Hodi FS, O'Day SJ, McDermott DF, Weber RW, Sosman JA, Haanen JB, et al. Improved survival with ipilimumab in patients with metastatic melanoma. N Engl J Med. 2010;363:711-23.

Ipilimumab Investigator Brochure version 23, dated 11-Mar-2020. Bristol-Myers Squibb Research and Development, 2020. Document Control No. 930017531.

Johnson DB, Estrada MV, Salgado R, Sanchez V, Doxie DB, Opalenik SR, et al. Melanoma-specific MHC-II expression represents a tumour-autonomous phenotype and predicts response to anti-PD-1/PD-L1 therapy. Nat Comm. 2016;7:10582.

Kvistborg P, Philips D, Kelderman S, Hageman L, Ottensmeier C, Joseph-Pietras D, et al. Anti-CTLA-4 therapy broadens the melanoma-reactive CD8+ T cell response. Sci Transl Med. 2014;6:254ra128.

Kwek SS, Cha E, Fong L. Unmasking the immune recognition of prostate cancer with CTLA4 blockade. Nat Rev Cancer. 2012;12(4):289-297.

Kwon ED, Drake CG, Scher HI, Fizazi K, Bossi A, van den Eertwegh AJM, et al. Ipilimumab versus placebo after radiotherapy in patients with metastatic castration-resistant prostate cancer that had progressed after docetaxel chemotherapy (CA184-043): a multicentre, randomised, double-blind, phase 3 trial. Lancet Oncol 2014;15(7):700–12.

Larkin J, Chiarion-Sileni V, Gonzalez R, Grob JJ, Cowey CL, Lao CD, et al. Combined nivolumab and ipilimumab or monotherapy in untreated melanoma. N Engl J Med. 2015;373:23-34.

Maby P, Galon J, Latouche JB. Frameshift mutations, neoantigens and tumor-specific CD8(+) T cells in microsatellite unstable colorectal cancers. Oncoimmunology. 2015;5(5):e1115943.

Madan RA, Arlen PM, Mohebtash M, Hodge JW, Gulley JL. Prostvac-VF: a vector-based vaccine targeting PSA in prostate cancer. Expert Opin Investig Drugs. 2009;18(7):1001-1011.

Madan RA, Mohebtash M, Arlen PM, et al. Ipilimumab and a poxviral vaccine targeting prostate-specific antigen in metastatic castration-resistant prostate cancer: a phase 1 dose-escalation trial. Lancet Oncol. 2012;13(5):501-508.

Maio M, Grob JJ, Aamdal S, Bondarenko I, Robert C, Thomas L, et al. Five-year survival rates for treatment-naïve patients with advanced melanoma who received ipilimumab plus dacarbazine in a phase III trial. J Clin Oncol. 2015;33:1191-6.

Momtaz P, Park V, Panageas KS, Postow MA, Callahan M, Wolchok JD, et al. Safety of infusing ipilimumab over 30 minutes. J Clin Oncol. 2015;33:3454-8.

Motzer RJ, Escudier B, McDermott DF, George S, Hammers HJ, Srinivas S, et al. Nivolumab versus everolimus in advanced renal-cell carcinoma. N Engl J Med. 2015;373:1803-13.

Motzer RJ, Tannir NM, McDermott DF, Aren Frontera O, Melichar B, Choueiri TK, et al. Nivolumab plus ipilimumab versus sunitinib in advanced renal-cell carcinoma. N Engl J Med. 2018. [Epub ahead of print]

NCT00861614: A Randomized, Double-Blind, Phase 3 Trial Comparing Ipilimumab vs. Placebo Following Radiotherapy in Subjects With Castration Resistant Prostate Cancer That Have Received Prior Treatment With Docetaxel [Internet]. clinicaltrials.gov; 2016 [cited 2020 May 31]. Available from: https://clinicaltrials.gov/ct2/show/NCT00861614.

NCT01024231: Dose-escalation Study of Combination BMS-936558 (MDX-1106) and Ipilimumab in Subjects With Unresectable Stage III or Stage IV Malignant Melanoma - Full Text View - ClinicalTrials.gov [Internet]. [cited 2020 Jun 5];Available from: https://clinicaltrials.gov/ct2/show/NCT01024231.

NCT01057810: Randomized, Double-Blind, Phase 3 Trial to Compare the Efficacy of Ipilimumab vs Placebo in Asymptomatic or Minimally Symptomatic Patients With Metastatic Chemotherapy-Naïve Castration Resistant Prostate Cancer [Internet]. clinicaltrials.gov; 2016 [cited 2020 May 31]. Available from: https://clinicaltrials.gov/ct2/show/NCT01057810.

NCT02985957: A Phase 2 Trial of Nivolumab Plus Ipilimumab, Ipilimumab Alone, or Cabazitaxel in Men With Metastatic Castration-Resistant Prostate Cancer - Full Text View - ClinicalTrials.gov [Internet]. [cited 2020 May 13]; Available from: https://clinicaltrials.gov/ct2/show/NCT02985957.

NCT03651271: An Exploratory Study of Nivolumab With or Without Ipilimumab According to the Percentage of Tumoral CD8 Cells in Participants With Advanced Metastatic Cancer - Full Text View - ClinicalTrials.gov [Internet]. [cited 2020 May 13]; Available from: https://clinicaltrials.gov/ct2/show/NCT03651271.

Nivolumab Investigator Brochure version 19, dated 29-Jun-2020. Bristol-Myers Squibb Research and Development, 2020. Document Control No. 930038243.

Nivolumab Investigator Brochure version 19 Addendum, dated 28-Sep-2020. Bristol-Myers Squibb Research and Development, 2020. Document Control No. 930160015.

Postow MA, Goldman DA, Shoushtari AN, Warner AB, Callahan MK, Momtaz P, et al. A phase II study to evaluate the need for > two doses of nivolumab + ipilimumab combination (combo) immunotherapy. JCO 2020;38(15_suppl):10003–10003.

Opdivo (nivolumab) US Prescribing Information (USPI), March 2020; Bristol-Myers Squibb Company, Princeton, NJ.

Ready N, Hellmann MD, Awad MM, Otterson GA, Gutierrez M, Gainor JF, et al. First-Line Nivolumab Plus Ipilimumab in Advanced Non-Small-Cell Lung Cancer (CheckMate 568): Outcomes by Programmed Death Ligand 1 and Tumor Mutational Burden as Biomarkers. J Clin Oncol 2019;37(12):992–1000.

Ribas A, Marin-Algarra S, Bhatia S, Hwu WJ, Slingluff, Jr CL, Sharfman WH, et al. Immunomodulatory effects of nivolumab and ipilimumab in combination or nivolumab monotherapy in advanced melanoma patients: CheckMate 038. AACR 2017.

Rizvi NA, Hellmann MD, Snyder A, Kvistborg P, Makarov V, Havel JJ, et al. Mutational landscape determines sensitivity to PD-1 blockade in non-small cell lung cancer. Science. 2015;348:124-8.

Robert C, Schachter J, Long GV, Arance A, Grob JJ, Mortier L, et al. Pembrolizumab versus Ipilimumab in Advanced Melanoma. N Engl J Med. 2015;372:2521-32.

Roy S, Trinchieri G. Microbiota: a key orchestrator of cancer therapy. Nat Rev Cancer. 2017;17:271-85.

Schadendorf D, Hodi FS, Robert C, Weber JS, Margolin K, Hamid O, et al. Pooled analysis of long-term survival data from phase II and phase III trials of ipilimumab in unresectable or metastatic melanoma. J Clin Oncol. 2015;33:1889-94.

Scher HI, Morris MJ, Stadler WM, Higano C, Basch E, Fizazi K, et al. Trial design and objectives for castration-resistant prostate cancer: Updated recommendations from the Prostate Cancer Clinical Trials Working Group 3. J Clin Oncol 2016;34(12):1402–18.

Sharma P, Pachynski RK, Narayan V, Flechon A, Gravis G, Galsky MD, et al. Initial results from a phase II study of nivolumab (NIVO) plus ipilimumab (IPI) for the treatment of metastatic castration-resistant prostate cancer (mCRPC; CheckMate 650). JCO 2019;37(7_suppl):142–142.

Slovin SF, Higano CS, Hamid O, et al. Ipilimumab alone or in combination with radiotherapy in metastatic castration-resistant prostate cancer: results from an open-label, multicenter phase I/II study. Ann Oncol. 2013;24(7):1813-1821.

Thall PF, Simon RM, Estey EH. Bayesian sequential monitoring designs for single-arm clinical trials with multiple outcomes. Stat Med. 1995;14:357-79.

Thompson E, Zahurak M, Murphy A, Cornish T, Cuka N, Abdelfatah E, et al. Patterns of PD-L1 expression and CD8 T cell infiltration in gastric adenocarcinomas and associated immune stroma. Gut. 2017;66:794-801.

Tumeh PC, Harview CL, Yearley JH, Shintaku IP, Taylor EJM, Robert L, et al. PD-1 blockade induces responses by inhibiting adaptive immune resistance. Nature. 2014;515:568-83.

Weber JS, D'Angelo SP, Minor D, Hodi FS, Gutzmer R, Neyns B, et al. Nivolumab versus chemotherapy in patients with advanced melanoma who progressed after anti-CTLA-4 treatment (CheckMate 037): a randomised, controlled, open-label, phase 3 trial. Lancet Oncol. 2015;16:375-84.

Wilson AL, Woods AN, Engelhard VH. Immunosuppression limits CD8+ T cell entry into subcutaneous B16 melanoma. J Immunol. 2016;196 (1 Supplement):212.6.

Wolchok JD, Hoos A, O'Day S, Weber JS, Hamid O, Lebbe C, et al. Guidelines for the evaluation of immune therapy activity in solid tumors: immune-related response criteria. Clin Cancer Res. 2009;15:7412-9.

Wolchok JD, Neyns B, Linette G, Negrier S, Lutzky J, Thomas L, et al. Ipilimumab monotherapy in patients with pretreated advanced melanoma: a randomised, double-blind, multicentre, phase 2, dose-ranging study. The Lancet Oncology. 2010;11(2):155-164.

Wolchok JD, Kluger H, Callahan MK, Postow MA, Rizvi NA, Lesokhin AM, et al. Safety and clinical activity of combined PD-1 (nivolumab) and CTLA-4 (ipilimumab) blockade in advanced melanoma patients. N Engl J Med 2013;369(2):122–33.

Wolchok JD, Chiarion-Sileni V, Gonzalez R, Rutkowski P, Grob JJ, Cowey CL, et al. Overall survival with combined nivolumab and ipilimumab in advanced melanoma. N Engl J Med. 2017;377:1345-56.

Yervoy (ipilimumab) US Prescribing Information (USPI), March 2020; Bristol-Myers Squibb Company, Princeton, NJ.

Certificate Of Completion

Envelope Id: A98E0FD249AC4B9BBD0DD15B80D54E33		Status: Completed
Subject: Please DocuSign: PICI0025 Amendment 4 Final (12-Mar-2021).pdf		
Source Envelope:		
Document Pages: 146	Signatures: 1	Envelope Originator:
Certificate Pages: 5	Initials: 0	Leonardo Nissola
AutoNav: Enabled		1 Letterman Drive
EnvelopeId Stamping: Disabled		San Francisco, CA 94129
Time Zone: (UTC-08:00) Pacific Time (US & Canada)		lnissola@parkerici.org
		IP Address: 24.5.29.64

Record Tracking

Status: Original<br>3/12/2021 2:42:37 PM	Holder: Leonardo Nissola<br>lnissola@parkerici.org	Location: DocuSign

Signer Events	Signature	Timestamp
Justin Fairchild<br>jfairchild@parkerici.org<br>VP, Clinical Development<br>Parker Institute for Cancer Immunotherapy<br>Security Level: Email, Account Authentication (Required)	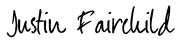<br>Signature Adoption: Pre-selected Style<br>Signature ID:<br>949277DE-9B08-4F27-973F-0D2BBB82C4F0<br>Using IP Address: 73.4.15.209<br><br>With Signing Authentication via DocuSign password<br>With Signing Reasons (on each tab):<br>I approve this document	Sent: 3/12/2021 2:45:03 PM<br>Viewed: 3/12/2021 4:04:43 PM<br>Signed: 3/12/2021 4:05:09 PM

Electronic Record and Signature Disclosure:
Accepted: 5/15/2019 9:13:01 AM
ID: b1573b3d-089b-41c9-8b03-72c198ebb255

In Person Signer Events	Signature	Timestamp

Editor Delivery Events	Status	Timestamp

Agent Delivery Events	Status	Timestamp

Intermediary Delivery Events	Status	Timestamp

Certified Delivery Events	Status	Timestamp

Carbon Copy Events	Status	Timestamp

Witness Events	Signature	Timestamp

Notary Events	Signature	Timestamp

Envelope Summary Events	Status	Timestamps
Envelope Sent	Hashed/Encrypted	3/12/2021 2:45:03 PM
Certified Delivered	Security Checked	3/12/2021 4:04:43 PM
Signing Complete	Security Checked	3/12/2021 4:05:09 PM
Completed	Security Checked	3/12/2021 4:05:09 PM

Payment Events	Status	Timestamps

Electronic Record and Signature Disclosure

ELECTRONIC RECORD AND SIGNATURE DISCLOSURE

From time to time, Parker Institute for Cancer Immunotherapy (we, us or Company) may be required by law to provide to you certain written notices or disclosures. Described below are the terms and conditions for providing to you such notices and disclosures electronically through the DocuSign system. Please read the information below carefully and thoroughly, and if you can access this information electronically to your satisfaction and agree to this Electronic Record and Signature Disclosure (ERSD), please confirm your agreement by selecting the check-box next to 'I agree to use electronic records and signatures' before clicking 'CONTINUE' within the DocuSign system.

Getting paper copies

At any time, you may request from us a paper copy of any record provided or made available electronically to you by us. You will have the ability to download and print documents we send to you through the DocuSign system during and immediately after the signing session and, if you elect to create a DocuSign account, you may access the documents for a limited period of time (usually 30 days) after such documents are first sent to you. After such time, if you wish for us to send you paper copies of any such documents from our office to you, you will be charged a $0.00 per-page fee. You may request delivery of such paper copies from us by following the procedure described below.

Withdrawing your consent

If you decide to receive notices and disclosures from us electronically, you may at any time change your mind and tell us that thereafter you want to receive required notices and disclosures only in paper format. How you must inform us of your decision to receive future notices and disclosure in paper format and withdraw your consent to receive notices and disclosures electronically is described below.

Consequences of changing your mind

If you elect to receive required notices and disclosures only in paper format, it will slow the speed at which we can complete certain steps in transactions with you and delivering services to you because we will need first to send the required notices or disclosures to you in paper format, and then wait until we receive back from you your acknowledgment of your receipt of such paper notices or disclosures. Further, you will no longer be able to use the DocuSign system to receive required notices and consents electronically from us or to sign electronically documents from us.

All notices and disclosures will be sent to you electronically

Unless you tell us otherwise in accordance with the procedures described herein, we will provide electronically to you through the DocuSign system all required notices, disclosures, authorizations, acknowledgements, and other documents that are required to be provided or made available to you during the course of our relationship with you. To reduce the chance of you inadvertently not receiving any notice or disclosure, we prefer to provide all of the required notices and disclosures to you by the same method and to the same address that you have given us. Thus, you can receive all the disclosures and notices electronically or in paper format through the paper mail delivery system. If you do not agree with this process, please let us know as described below. Please also see the paragraph immediately above that describes the consequences of your electing not to receive delivery of the notices and disclosures electronically from us.

How to contact Parker Institute for Cancer Immunotherapy:

You may contact us to let us know of your changes as to how we may contact you electronically, to request paper copies of certain information from us, and to withdraw your prior consent to receive notices and disclosures electronically as follows:
To contact us by email send messages to: pici-support@parkerici.org

To advise Parker Institute for Cancer Immunotherapy of your new email address

To let us know of a change in your email address where we should send notices and disclosures electronically to you, you must send an email message to us at pici-support@parkerici.org and in the body of such request you must state: your previous email address, your new email address. We do not require any other information from you to change your email address.

If you created a DocuSign account, you may update it with your new email address through your account preferences.

To request paper copies from Parker Institute for Cancer Immunotherapy

To request delivery from us of paper copies of the notices and disclosures previously provided by us to you electronically, you must send us an email to sbolisetty@parkerici.org and in the body of such request you must state your email address, full name, mailing address, and telephone number. We will bill you for any fees at that time, if any.

To withdraw your consent with Parker Institute for Cancer Immunotherapy

To inform us that you no longer wish to receive future notices and disclosures in electronic format you may:

i. decline to sign a document from within your signing session, and on the subsequent page, select the check-box indicating you wish to withdraw your consent, or you may;

ii. send us an email to pici-support@parkerici.org and in the body of such request you must state your email, full name, mailing address, and telephone number. We do not need any other information from you to withdraw consent.. The consequences of your withdrawing consent for online documents will be that transactions may take a longer time to process..

Required hardware and software

The minimum system requirements for using the DocuSign system may change over time. The current system requirements are found here: https://support.docusign.com/guides/signer-guide-signing-system-requirements.

Acknowledging your access and consent to receive and sign documents electronically

To confirm to us that you can access this information electronically, which will be similar to other electronic notices and disclosures that we will provide to you, please confirm that you have read this ERSD, and (i) that you are able to print on paper or electronically save this ERSD for your future reference and access; or (ii) that you are able to email this ERSD to an email address where you will be able to print on paper or save it for your future reference and access. Further, if you consent to receiving notices and disclosures exclusively in electronic format as described herein, then select the check-box next to 'I agree to use electronic records and signatures' before clicking 'CONTINUE' within the DocuSign system.

By selecting the check-box next to 'I agree to use electronic records and signatures', you confirm that:

- You can access and read this Electronic Record and Signature Disclosure; and
- You can print on paper this Electronic Record and Signature Disclosure, or save or send this Electronic Record and Disclosure to a location where you can print it, for future reference and access; and
- Until or unless you notify Parker Institute for Cancer Immunotherapy as described above, you consent to receive exclusively through electronic means all notices, disclosures, authorizations, acknowledgements, and other documents that are required to be provided or made available to you by Parker Institute for Cancer Immunotherapy during the course of your relationship with Parker Institute for Cancer Immunotherapy.

Statistical Analysis Plan for

Protocol Number: PICI0025

Protocol Title: An Exploratory Study of Nivolumab with or without Ipilimumab According to the Percentage of Tumoral CD8 Cells in Participants with Advanced Metastatic Cancer

**IND Number:**	138520
**Sponsor:**	Parker Institute for Cancer Immunotherapy<br>1 Letterman Drive<br>Suite D3500<br>San Francisco, CA 94129
**SAP Version Number:**	1.0
**Date Final:**	10 January 2022

-CONFIDENTIAL-

This document and its contents are the property of and confidential to Parker Institute for Cancer Immunotherapy. Any unauthorized copying or use of this document is prohibited.

SPONSOR APPROVAL PAGE

DocuSigned by:

Christopher Cabanski

Signer Name: Christopher Cabanski
Signing Reason: I approve this document
Signing Time: 1/10/2022 | 11:36:01 AM PST
73CCABA749D14634B5D6314D83F02F85

Christopher Cabanski, PhD
Senior Director, Biometrics

DocuSigned by:

Justin Fairchild

Signer Name: Justin Fairchild
Signing Reason: I approve this document
Signing Time: 1/10/2022 | 1:27:58 PM PST
949277DE9B084F27973F0D2BBB82C4F0

Justin Fairchild, MPH
VP, Clinical Development

1 TABLE OF CONTENTS

1 TABLE OF CONTENTS 3
List of Tables 4
2 INTRODUCTION 5
3 STUDY DESIGN 5
3.1 Protocol Synopsis 6
3.2 Study Objectives and Endpoints 6
3.3 Determination of Sample Size 8
3.4 Analysis Timing 8
4 STUDY CONDUCT 8
4.1 Intervention Assignment 8
4.2 Blinding 9
4.3 Safety Monitoring for Advanced Metastatic Cancer 9
4.4 Safety Monitoring for Advanced Prostate Cancer 9
5 STATISTICAL ANALYSIS 10
5.1 Populations for Analysis 10
5.2 Analysis of Study Conduct 10
5.3 Analysis of Baseline Characteristics 11
5.4 Efficacy Analysis 11
5.4.1 Primary Efficacy Endpoints 11
5.4.2 Secondary Efficacy Endpoints 12
5.4.2.1 Objective Response Rate 12
5.4.2.2 Progression-Free Survival 12
5.4.2.3 Overall Survival 13
5.4.2.4 Association of CD8 with clinical outcomes 13
5.4.3 Exploratory Efficacy Endpoints 14
5.4.3.1 Prostate-specific Antigen Analysis (Advanced Prostate Cancer) 14
5.4.4 Subgroup Analyses 14
5.5 Biomarker Analysis 14
5.6 Safety Analysis 15
5.6.1 Exposure to Study Medication 15
5.6.2 Adverse Events 15
5.6.3 Laboratory Data 16

5.6.4 Vital Signs....16
5.6.5 Physical Examinations....16
5.7 Patient-Reported Outcomes Analysis....16
5.8 Missing Data....16
5.8.1 Missing and Partial Missing Adverse Event Dates....16
5.8.2 Missing and Partial Missing Death Dates....17
5.9 Interim Analyses....17
6 DIFFERENCES COMPARED TO PROTOCOL....18
7 REFERENCES....19
8 APPENDICES....20
8.1 Protocol Synopsis....20

LIST OF TABLES

Table 1: Objectives and Corresponding Endpoints....6
Table 2: Populations for Analysis....10
Table 3: Logistic Regression Model Parameters....14

2 INTRODUCTION

The aim of this study is to provide a prospective classification of CD8 high (immunologically "hot") versus CD8 low (immunologically "cold") tumors at the time of treatment, based on the percentage of CD8 cells in a tumor biopsy, and to address the predictive value of the CD8 biomarker for selecting patients for treatment with nivolumab with or without ipilimumab.

The purpose of this document is to provide details of the planned clinical analyses for PICI0025. The analyses specified in this document supersede the high-level analysis plan described in the protocol. Statistical analyses will be performed consistent with the principles of the ICH Guidance for Industry E9 Statistical Principles for Clinical Trials.

3 STUDY DESIGN

This is an open-label, exploratory study to evaluate nivolumab with or without ipilimumab, with treatment assigned based on the percentage of tumoral CD8 cells at the time of treatment in participants with varying advanced solid tumors.

After consenting to participate in this clinical trial, participants will undergo a tumor biopsy. The tumor tissue will be sent to a Clinical Laboratory Improvement Amendment (CLIA)- and College of American Pathologists (CAP)-certified Immunohistochemistry and Image Analysis Laboratory to determine the percentage of tumoral CD8 cells. Participants will be assigned an immunotherapy regimen according to the percentage of CD8 cells in their tumor biopsies.

There are two distinct cohorts enrolled in this study.

- Advanced Metastatic Cancer: enrolled participants with varying advanced solid tumors.
    - Participants with ≥ 15% CD8 cells in their tumor biopsies (ie, CD8 high tumors) will be treated with nivolumab 360 mg every 3 weeks (Q3W) for 4 cycles, followed by nivolumab maintenance 480 mg every 4 weeks (Q4W). At the time of disease progression, participants may crossover to the CD8 low arm or discontinue treatment.
    - Participants with < 15% CD8 cells in their tumor biopsies (ie, CD8 low tumors) will be treated with nivolumab 360 mg Q3W and ipilimumab 1 mg/kg Q3W for 2 doses and then every 6 weeks (Q6W) for 2 doses, followed by nivolumab maintenance 480 mg Q4W.
- Advanced Prostate Cancer (Protocol Amendment 3): enrolled participants with advanced prostate cancer.
    - Participants with ≥ 15% CD8 cells in their tumor biopsies will be treated with nivolumab 360 mg Q3W for 4 cycles, followed by nivolumab maintenance

480 mg Q4W. At the time of disease progression, participants may crossover to the advanced prostate cohort CD8 low arm of ipilimumab 3 mg/kg and nivolumab 1 mg/kg or discontinue treatment.

- Participants with < 15% CD8 cells in their tumor biopsies (ie, CD8 low tumors) will be randomly allocated into 1 of 2 treatment arms using different doses of ipilimumab.
  - Prostate Cohort A: nivolumab 1 mg/kg Q3W and ipilimumab 3 mg/kg Q6W for 2 cycles, then nivolumab maintenance 480 mg Q4W until disease progression or intolerable toxicity.
  - Prostate Cohort B: nivolumab 1 mg/kg Q3W and ipilimumab 5 mg/kg Q6W for 2 cycles, then nivolumab maintenance 480 mg Q4W until disease progression or intolerable toxicity.

Up to 200 participants with advanced metastatic cancer could be enrolled. A total of 20 participants with advanced prostate cancer and tumoral CD8 < 15% could be enrolled into the Advanced Prostate Cancer portion of the study (10 per treatment arm).

3.1 Protocol Synopsis

The Protocol Synopsis is included in Section 8.1.

3.2 Study Objectives and Endpoints

The study objectives and endpoints are listed in Table 1.

Table 1: Objectives and Corresponding Endpoints

**Objectives**	**Endpoints**
Primary	
• To determine the clinical benefit rate (CBR) of nivolumab with or without ipilimumab in participants with advanced metastatic cancer, including advanced prostate cancer.<br>• To assess the proportion of participants in the nivolumab plus ipilimumab arm whose tumors will change from CD8 low to CD8 high as measured by a change in the percentage of tumoral CD8 cells.	• CBR is the proportion of participants who show clinical benefit, defined as CR, PR, or SD for ≥ 6 months as best response by RECIST v1.1.<br>• Change in the percentage of CD8 cells in on-treatment biopsies from baseline.
Secondary	
• To determine the safety and tolerability of nivolumab with or without ipilimumab in participants with advanced metastatic cancer, including advanced prostate cancer.	• Incidence and severity of AEs based on CTCAE v5.0.<br>• ORR: Defined as CR or PR as best response by RECIST v1.1 assessment.

Objectives	Endpoints
• To determine the ORR of nivolumab with or without ipilimumab in participants with advanced metastatic cancer, including advanced prostate cancer.<br>• To assess the association of percentage of CD8 infiltration in tumor samples with clinical outcomes (ORR, PFS, and OS).	• PFS: Defined as the time from initiation of study therapy to date of first documented progression of disease or date of death due to any cause.<br>• OS: Defined as the time from initiation of study therapy until death due to any cause.
Exploratory	
• To evaluate tumor immune biomarkers and their association with treatment outcomes.<br>• To evaluate changes in prostate-specific antigen (PSA) (advanced prostate cancer).<br>• To assess feasibility of implementing the ApricityCare™ digital application (app) for participants being treated with immune checkpoint inhibitor(s) and the use by study staff of ApricityOncology™ website.	• Clinical responses based on a composite biomarker derived from nucleic acids, epigenetics, protein, immune cell characteristics, or other factors (CD8 and other biomarkers).<br>• Additional biomarkers of primary resistance to nivolumab or ipilimumab combined with nivolumab beyond the percentage of tumoral CD8 cells based on additional immune evaluation of baseline and on-treatment biopsies or blood samples.<br>• Potential relationship of T-cell phenotypic characteristics and immune characteristics in the tumor microenvironment in participants on nivolumab monotherapy and nivolumab in combination with ipilimumab with clinical outcomes (clinical benefit endpoints and AEs with an interest in immune-related AEs).<br>• Within each study intervention arm, explore the potential relationship of clinical response/resistance/AE with:<br>o mRNA quantity and expression in tumor and/or PBMCs<br>o Germline DNA and/or tumor genomics<br>o Multiparameter cytometry of tumor and/or PBMCs<br>o Immune markers in PBMCs<br>o Blood circulating analytes<br>o TCR repertoire in tumor and peripheral blood<br>o Baseline and on-treatment microbiome<br>• Identify molecular (genomic, metabolic, and/or proteomic) biomarkers that may be indicative of clinical response/resistance, safety, pharmacodynamic activity, and/or mechanism of

Objectives	Endpoints
	action of nivolumab and/or ipilimumab in combination with nivolumab.<br>• Change in PSA from baseline (advanced prostate cancer).<br>• Frequency of ApricityCare™ app usage by participants and frequency of ApricityOncology website access by study staff.

3.3 Determination of Sample Size

A total of up to approximately 200 participants with varying tumor types will be enrolled. Enrollment to initial biopsy may be about 10% higher (ie, approximately 220 participants) to account for unexpected challenges obtaining CD8 infiltration information. The selection of 200 participants allows minimum reasonable numbers in the CD8 high and CD8 low tumor groups in larger tumor populations to report subgroup response rates, the transformation from CD8 low to high, as well as to predict response from marker and clinical information. Details of the sample size determination can be found in the protocol section 9.1.

3.4 Analysis Timing

Analysis for the Advanced Metastatic Cancer and Advanced Prostate Cancer portions will be performed independently and at different times due to differences in enrollment timelines.

The analysis of complete data for the clinical study report (CSR), described in the main body of this document, will be performed after all participants have been followed for at least 9 months or have discontinued early from the study, and all data from the study through the clinical cutoff date have been entered and reviewed for completeness.

Analysis of efficacy and safety endpoint may be performed prior to the CSR analysis. These results may be presented and/or published prior to the CSR analysis.

The study will formally end once all participants have either completed the safety and survival follow-up periods or have discontinued early from the study, all data from the study are entered in the clinical database, and the clinical database is locked.

4 STUDY CONDUCT

4.1 Intervention Assignment

The advanced metastatic cancer portion of this study is not randomized. Treatment assignment will be based on the percentage of tumoral CD8 cells at the initial biopsy. Participants who have a tumor with ⩾ 15% CD8 cells will receive nivolumab monotherapy, and participants who have a tumor with < 15% CD8 cells will receive ipilimumab in combination with nivolumab.

Approximately 20 advanced prostate cancer participants who have a tumor with < 15% CD8 cells will be randomly allocated to receive nivolumab 1 mg/kg in combination with ipilimumab 3 mg/kg (Prostate Cohort A) or nivolumab 1 mg/kg in combination with ipilimumab 5 mg/kg (Prostate Cohort B) in a 1:1 ratio. All advanced prostate cancer participants who have a tumor with ⩾ 15% CD8 cells will receive nivolumab monotherapy. Participants who are randomly allocated but do not receive study intervention for any reason may be replaced. Randomization will be performed by the Sponsor.

Study intervention will be administered by site personnel and tracked using drug accountability records.

4.2 Blinding

This is an open-label trial; therefore, the Sponsor, Investigator, and participant will know the study intervention administered.

4.3 Safety Monitoring for Advanced Metastatic Cancer

The safety of nivolumab and ipilimumab therapies will be monitored during the trial as per the interim analyses described in Section 5.9.

4.4 Safety Monitoring for Advanced Prostate Cancer

As the combination regimen with ipilimumab 5 mg/kg has not been extensively studied, in addition to adverse events being reviewed by the Sponsor on an ongoing basis and investigator meetings convening approximately every 2 weeks to discuss and review safety events, a safety pause is included after the 5th patient has initiated treatment with ipilimumab at the 5 mg/kg dose level.

Enrollment will pause until all participants have been followed for at least 6 weeks. The safety pause will apply to both cohorts to preserve random allocation; however, the safety assessment as follows will apply to the ipilimumab 5 mg/kg cohort specifically. The initial 5 participants enrolled in the ipilimumab 5 mg/kg cohort will be observed for the occurrence of treatment-related adverse events (TRAEs) necessitating discontinuation of both agents from the beginning of treatment through completion of the first cycle (ie, 6 weeks).

If 3 or fewer of the first 5 participants in the ipilimumab 5 mg/kg cohort experience a TRAE leading to discontinuation of both agents, enrollment for that specific cohort will continue as planned until a total of approximately 10 participants are enrolled. If 4 or more of the first 5 participants of the ipilimumab 5 mg/kg cohort experience a TRAE leading to discontinuation of both agents, enrollment into the cohort will be suspended, and an ad hoc safety review (conducted by Investigators and Sponsor representatives) will be performed to assess the available data and provide a recommendation, which may include, but is not limited to, modification or discontinuation of the cohort, and will be documented in the study records. Only TRAEs leading to treatment discontinuation of both agents occurring up until the 5th

participant in each cohort completes Cycle 1 will be included—dose delays for adverse events not otherwise meeting criteria at that time will not be counted. The stopping rule was chosen as this corresponds to a > 80% chance that the toxicity rate in the cohort is > 40%, assuming a beta (0.8, 1.2) prior.

Should the ipilimumab 5 mg/kg cohort demonstrate 6 or greater treatment-related adverse events requiring discontinuation of therapy at any time, enrollment will be suspended until an ad hoc safety review convenes and provides a recommendation, as described above.

5 STATISTICAL ANALYSIS

Summary statistics will be presented by study intervention group. Categorical variables will be summarized using frequencies and percentages. Continuous variables will be summarized using means, standard deviations, medians, interquartile ranges (IQR; 25th to 75th percentile), minimums, and maximums.

5.1 Populations for Analysis

For purposes of analysis, the following populations are defined (Table 2):

Table 2: Populations for Analysis

Population	Description
Safety Population	All participants who received at least one dose of study drug. Participants will be analyzed according to the study intervention actually received.
Modified Intent-to-Treat (mITT) Population	All participants with sufficient biopsy results to be assigned to a treatment arm and who received at least 1 dose of study drug. Participants will be analyzed according to the study intervention to which they were assigned.
On-treatment Biopsy Population	All participants with at least 1 on-treatment biopsy with sufficient CD8 results will be included in the analyses of changes in CD8 counts.

5.2 Analysis of Study Conduct

The number of participants who were screened, enrolled/randomized, treated, and completed the study will be presented in a summary table. The reason for treatment discontinuation and study discontinuation will also be summarized.

The participant time on treatment and time on study will be presented in a summary table. Time on treatment is defined as the date from first dose of study intervention until the date of last dose of study intervention. Time on study is defined as the date from first dose of study intervention until the date of death or last contact. The number of participants currently on

treatment and study will presented along with the median time on treatment/study and the associated range. Time on treatment will be calculated using the Kaplan-Meier method, where participants who have discontinued treatment are considered to have an event and participants remaining on treatment are censored at their most recent treatment date. Time on study will be calculated using the reverse Kaplan-Meier method, where participants who remain on study (active treatment or in follow-up) are considered to have an event and participants who died or have discontinued the study are censored at their death or end of study date.

5.3 Analysis of Baseline Characteristics

Demographic and baseline characteristics of the study population will be summarized overall and for each study intervention group. Variables to be summarized include, but are not limited to, age, sex, race, ethnicity, Eastern Cooperative Oncology Group (ECOG) performance status, cancer location, stage at initial diagnosis, prior lines of systemic cancer therapy, and CD8 percentage at screening.

The baseline value of any variable will be defined as the last value recorded on or before the date of first administration of study intervention.

5.4 Efficacy Analysis

The efficacy analysis will be based on the modified Intent-to-Treat (mITT) Population, which comprises all enrolled participants with sufficient biopsy results to be assigned to a treatment arm and who received at least one dose of study drug, unless otherwise specified. Participants will be analyzed according to the study intervention to which they were assigned.

This study is not intended or powered for hypothesis testing, including comparisons between study intervention groups. Due to the exploratory nature of this study, no control of type I error will be applied for any of the endpoints.

5.4.1 Primary Efficacy Endpoints

The co-primary endpoint of CBR will be reported among all participants within each study intervention group with a 95% credible interval assuming a beta (0.4, 1.6) prior. CBR is the proportion of participants who show clinical benefit, defined as CR, PR, or SD for ⩾ 6 months as best response by RECIST v1.1 (Eisenhauer, 2009). Per RECIST, to be assigned a best overall response of complete response (CR) or partial response (PR), changes in tumor measurements must be confirmed by a repeat assessment that should be performed no less than 4 weeks after the criteria for response are first met. The duration of SD is defined as the time from the date of first dose of study intervention until the date of radiographic disease progression per RECIST. If no radiographic progression has occurred and the most recent tumor assessment with overall response of SD occurred < 6 months after the date of first dose of study intervention, the participant will not have met the SD duration criterion and will not

be considered a CBR responder. Participants who do not have a disease assessment will be counted as not having clinical benefit.

For the second co-primary endpoint, the absolute change in the percentage of CD8 cells in on-treatment biopsies from baseline will be reported with a 95% credible interval with no prior for participants in the on-treatment biopsy population. In addition, the number of participants with baseline percentage of CD8 counts < 15% who “convert” from a cold to hot tumor (any on-treatment biopsy with percentage of CD8 counts ≥ 15%) will be summarized. The proportion of participants who convert from cold to hot will be reported with a 95% credible interval with no prior.

5.4.2 Secondary Efficacy Endpoints

Secondary efficacy endpoints include ORR, PFS, and OS and the association between CD8 counts and response.

5.4.2.1 Objective Response Rate

Objective Response Rate (ORR), is defined as the proportion of participants who attain a best overall response of CR or PR, as determined by RECIST version 1.1. Confirmation of response by a repeat tumor assessment is required for a best overall response of CR or PR. Participants without a post-baseline tumor assessment will be considered non-responders, as well as patients with a best overall response of stable disease (SD), progressive disease (PD) or not evaluable (NE).

A 95% credible interval for ORR will be calculated assuming a beta (0.4, 1.6) prior.

5.4.2.2 Progression-Free Survival

Progression-Free Survival (PFS) is defined as the time from initiation of study intervention to the date of first documented radiographic progression of disease or date of death due to any cause, whichever occurs first. Participants who continue treatment beyond initial disease progression will be considered to have progression of disease (PD) at the time of the initial progression event.

Participants who do not have radiographic PD at the time of analysis will be censored as follows:

- Participants who do not have radiographic PD and are still on study at the time of analysis will be censored at the date of the last tumor assessment documenting absence of progressive disease.
- Participants who have discontinued study treatment and have started subsequent anti-cancer therapy or had subsequent cancer surgery or radiation prior to documentation of radiographic PD will be censored at the date of the last evaluable tumor assessment prior to the initiation of subsequent treatment.

- Participants who discontinued the study prior to documentation of radiographic PD will be censored at the date of the last tumor assessment documenting absence of progressive disease.
- Participants who do not have radiographic PD and who die more than 16 weeks from their last evaluable tumor assessment will be censored at the date of the last tumor assessment documenting absence of progressive disease. Participants who die within 16 weeks of their last evaluable tumor assessment will be considered as having an event at the date of death.

PFS will be estimated using Kaplan-Meier techniques, and the median survival time and 95% CIs will be estimated within each study intervention group.

5.4.2.3 **Overall Survival**

Overall Survival (OS) is defined as the time from initiation of study intervention until death due to any cause. Participants who are not reported as having died at the time of analysis will be censored at the most recent contact date they were known to be alive. See Section 5.8.2 for handling of missing or partial death dates.

OS will be estimated using Kaplan-Meier techniques, and the median survival time and 95% CIs will be estimated within each study intervention group.

5.4.2.4 **Association of CD8 with clinical outcomes**

Several analyses will be performed to investigate the association between the percentage of CD8 cells and clinical outcomes.

- Among the participants who had tumors that converted from cold (CD8 <15%) at baseline to hot (CD8 ≥ 15%) at any on-treatment biopsy, the following will be reported:
  - The number and proportion of patients who met ORR response criteria. A 95% credible interval will be estimated with no prior.
  - The number and proportion of patients who met CBR response criteria. A 95% credible interval will be estimated with no prior.
- Logistic regression models will be fit to assess the relationship between percentage of CD8 and clinical response (ORR and CBR). Each regression model will include an intercept term. P-values will be calculated using a Wald test to test whether the coefficient for the CD8 variable is significantly different from 0. See Table 3 for details about each regression model.

Table 3: Logistic Regression Model Parameters

Model number	Response variable (y)	Explanatory variable (x)	Analysis Population
1	CBR	Baseline percentage of CD8 cells	mITT Population
2	ORR	Baseline percentage of CD8 cells	mITT Population
3	CBR	Maximum percentage of CD8 cells across all on-treatment biopsies	On-treatment Biopsy Population
4	ORR	Maximum percentage of CD8 calls across all on-treatment biopsies	On-treatment Biopsy Population
5	CBR	Absolute change between baseline and maximum percentage of CD8 cells across all on-treatment biopsies	On-treatment Biopsy Population
6	ORR	Absolute change between baseline and maximum percentage of CD8 cells across all on-treatment biopsies	On-treatment Biopsy Population
7	CBR	CD8 Conversion Flag<br>(1 = CD8 converter, 0 = non-converter)	On-treatment Biopsy Population
8	ORR	CD8 Conversion Flag<br>(1 = CD8 converter, 0 = non-converter)	On-treatment Biopsy Population

5.4.3 Exploratory Efficacy Endpoints

5.4.3.1 Prostate-specific Antigen Analysis (Advanced Prostate Cancer)

For the Advanced Prostate Cancer participants, descriptive statistics will be used to evaluate PSA at each timepoint. The absolute and percent changes from baseline will be summarized at each timepoint.

5.4.4 Subgroup Analyses

Efficacy endpoints (CBR, ORR, CD8 conversion) may be presented by tumor type if a tumor type has at least 5 participants enrolled in a treatment group.

5.5 Biomarker Analysis

Biomarker exploratory analyses will be determined based on study outcomes and further described in a translational analysis plan.

5.6 Safety Analysis

The safety analysis will be based on the Safety Population, which comprises all participants who receive at least one dose of study drug. Participants will be analyzed according to the study intervention actually received.

Safety and tolerability will be assessed through AEs, clinical laboratory parameters, vital signs, and ECGs.

5.6.1 Exposure to Study Medication

The number of participants exposed to each study intervention and the extent of exposure (as number of doses and cumulative dose received) will be summarized by treatment arm using descriptive statistics. The number of skipped doses will also be summarized by treatment arm.

5.6.2 Adverse Events

All reported AEs will be coded using a recent version of the Medical Dictionary for Regulatory Activities (MedDRA) and graded using the NCI Common Terminology Criteria for Adverse Events (CTCAE) version 5.0.

A treatment-emergent adverse event (TEAE) is defined as any event that either occurs after the initiation of study intervention, having been absent at baseline, or, if present at baseline, appears to have worsened in severity or frequency, whether or not the event is considered related to study intervention. A treatment-related adverse event (TRAE) is defined as any TEAE assessed by the Investigator to be ‘Possibly’, ‘Probably’, or ‘Definitely’ related to any study intervention, as well as AEs with missing relationship. An immune-related adverse event (IRAE) is defined as a TRAE that matches a select list of coded preferred terms that have been identified by medical review.

Handling of missing and partial-missing AE dates is described in Section 5.8.1.

The incidence of AEs will be summarized by study intervention group for the following categories:

- All TEAEs
- All TRAEs
- Grade 3-4 TEAEs and TRAEs
- Serious adverse events (SAEs)
- Deaths due to an AE
- AEs leading to treatment discontinuation
- All irAEs

Treatment-emergent and treatment-related AEs will also be summarized by coded preferred Term, system organ class, and severity. In addition, separate summaries will be generated for SAEs.

The following listings will be generated:

- TEAEs
- SAEs
- AEs leading to treatment discontinuation
- Deaths, including primary cause of death

5.6.3 Laboratory Data

Clinical laboratory findings will be summarized by the proportion of participants with on-treatment values outside the normal range for each study intervention group.

Select laboratory tests (including but not limited to alanine aminotransferase, albumin, alkaline phosphatase, aspartate aminotransferase, creatinine, platelet count, potassium, sodium, and white blood cells [WBC]) will be graded according to CTCAE version 5.0, and the highest grade per laboratory test per participant will be summarized by study intervention group.

5.6.4 Vital Signs

Vital signs at each visit and change from baseline to each visit will be summarized using descriptive statistics for each study intervention group. The baseline value of any variable will be defined as the last value recorded prior to the first administration of study intervention. Participants with a missing baseline value will not be summarized for that variable.

5.6.5 Physical Examinations

Physical examination data will not be summarized because any significant finding will be reported and summarized as an AE.

5.7 Patient-Reported Outcomes Analysis

For participants enrolled into the Advanced Prostate Cancer cohort, an exploratory analysis may be performed with the patient-reported dataset collected via ApricityCare™, including but not limited to the frequency with which participants complete the daily symptom survey in ApricityCare™, and the frequency of Apricity website access by investigative sites.

5.8 Missing Data

5.8.1 Missing and Partial Missing Adverse Event Dates

If the AE start date is not a complete date, the following rules will be applied to determine whether the event is treatment emergent.

- If the start date is completely missing: The AE will be considered treatment emergent unless the AE stop date is earlier than the date of first dose of study intervention.
- If the day of the AE start date is missing:
    - If the month and year of the start date are later than the month and year of the date of first dose of study intervention, then the AE will be considered treatment emergent.
    - If the month and year of the start date are equal to the month and year of the date of first dose of study intervention and the stop date is unknown or later than the date of first dose of study intervention, then the AE will be considered treatment emergent.
- If the day and month of the start date are missing:
    - If the year of the start date is later than the year of the date of first dose of study intervention, then the AE will be considered treatment emergent.
    - If the year of the start date is equal to the year of the date of first dose of study intervention and the stop date is unknown or later than the date of first dose of study intervention, then the AE will be considered treatment emergent.

5.8.2 Missing and Partial Missing Death Dates

For death dates, the following conventions will be used for imputing partial dates:

- If the date of death is completely or partially missing, but there is an AE with the outcome as ‘Fatal’, the date of death will be replaced by the end date of the AE.
- If only the day of the month is missing and there is no AE with outcome as ‘Fatal’, the 1st of the month will be used to replace the missing day. The imputed date will be compared to the last known date alive plus 1 day, and the maximum will be considered as the death date.
- If the month or the year is missing, the death date will be imputed as the last known date alive plus 1 day.
- If the date is completely missing but the reason for death is present, the death date will be imputed as the last known date alive plus 1 day.

5.9 Interim Analyses

Ongoing monitoring for safety and futility will be implemented based on the method of Thall and colleagues (Thall et al, 1995) separately in the CD8 high and CD8 low tumor groups for advanced metastatic cancer and advanced prostate cancer participants. Monitoring will be ongoing during enrollment in each group, once a participant receives treatment and their first treatment biopsy is performed. Screening and accrual to each group will continue until

sufficient information is available to assess the stopping rules as defined in the protocol Section 9.3.

6 DIFFERENCES COMPARED TO PROTOCOL

The Protocol defines the secondary endpoint exploration of baseline CD8 counts association with CBR and with OS; this was removed from the SAP and will not be analyzed.

7 REFERENCES

Eisenhauer EA, Therasse P, Bogaerts J, Schwartz LH, Sargent D, Ford, R, et al. New response evaluation criteria in solid tumours: Revised RECIST guideline (version 1.1). Eur J Cancer 2009;45(2):228-47.

ICH Guidance for Industry E9 Statistical Principles for Clinical Trials. U.S. Department of Health and Human Services, Food and Drug Administration, September 1998.

Thall PF, Simon RM, Estey EH. Bayesian sequential monitoring designs for single-arm clinical trials with multiple outcomes. Stat Med. 1995;14:357-79.

8 APPENDICES

8.1 Protocol Synopsis

Protocol Title:

An Exploratory Study of Nivolumab with or without Ipilimumab According to the Percentage of Tumoral CD8 Cells in Participants with Advanced Metastatic Cancer

Short Title:

Treatment with Nivolumab and Ipilimumab or Nivolumab Alone According to the Percentage of Tumoral CD8 Cells in Advanced Metastatic Cancer

Rationale:

The aim of this study is to provide a prospective classification of CD8 high (immunologically "hot") versus CD8 low (immunologically "cold") tumors at the time of treatment, based on the percentage of CD8 cells in a tumor biopsy, and to address the predictive value of the CD8 biomarker for selecting patients for treatment with nivolumab with or without ipilimumab.

Key Objectives and Endpoints:

Objectives	Endpoints
Primary	
• To determine the clinical benefit rate (CBR) of nivolumab with or without ipilimumab in participants with advanced metastatic cancer, including advanced prostate cancer.<br>• To assess the proportion of participants in the nivolumab plus ipilimumab arm whose tumors will change from CD8 low to CD8 high as measured by a change in the percentage of tumoral CD8 cells.	• CBR is the proportion of participants who show clinical benefit, defined as CR, PR, or SD for ⩾ 6 months as best response by RECIST v1.1.<br>• Change in the percentage of CD8 cells in on-treatment biopsies from baseline.
Secondary	
• To determine the safety and tolerability of nivolumab with or without ipilimumab in participants with advanced metastatic cancer, including advanced prostate cancer.<br>• To determine the ORR of nivolumab with or without ipilimumab in participants	• Incidence and severity of AEs based on CTCAE v5.0.<br>• ORR: Defined as CR or PR as best response by RECIST v1.1 assessment.<br>• PFS: Defined as the time from initiation of study therapy to date of first

Objectives	Endpoints
with advanced metastatic cancer, including advanced prostate cancer.<br>• To assess the association of percentage of CD8 infiltration in tumor samples with clinical outcomes (ORR, PFS, and OS).	documented progression of disease or date of death due to any cause.<br>• OS: Defined as the time from initiation of study therapy until death due to any cause.

AE = adverse event; CBR = clinical benefit rate; CR = complete response; CTCAE = Common Terminology Criteria for Adverse Events; ORR = objective response rate; OS = overall survival; PFS = progression-free survival; PR = partial response; RECIST = Response Evaluation Criteria in Solid Tumors; SD = stable disease.

Overall Design:

This is an open-label, exploratory study to evaluate nivolumab with or without ipilimumab based on percentage of tumoral CD8 cells at the time of treatment in participants with varying advanced solid tumors. Participants who have a tumor with ⩾ 15% CD8 cells (classified as CD8 high) will receive nivolumab monotherapy, and participants who have a tumor with < 15% CD8 cells (classified as CD8 low) will receive ipilimumab in combination with nivolumab.

Number of Participants:

A total of up to approximately 200 participants will be treated. Beginning with Amendment 2, enrollment will be limited to tumor types known to be responsive to immunotherapy, have high prevalence (> 20%) CD8 ⩾ 15% tumors, and/or have been observed in the study to have tumors transition from CD8 low to CD8 high following initiation of immunotherapy. Beginning with Amendment 3, a total of approximately 20 participants with advanced prostate cancer and tumoral CD8 < 15% will be randomly allocated to 1 of 2 cohorts using combinations of ipilimumab and nivolumab. Advanced prostate cancer participants with tumoral CD8 ⩾ 15% will be enrolled in the nivolumab monotherapy arm. Ongoing monitoring for safety and futility will be implemented based on the method of Thall and colleagues (Thall et al, 1995) separately in the CD8 high and CD8 low tumor groups.

Intervention Groups and Duration:

Advanced Metastatic Cancer: Single-agent nivolumab will be administered at 360 mg intravenously (IV) every 3 weeks (Q3W). Participants who continue to show clinical benefit after the first disease assessment will receive nivolumab 480 mg IV every 4 weeks (Q4W) until progressive disease (PD) or intolerable toxicity. At PD, participants will be allowed to crossover to the CD8 low arm of ipilimumab (1 mg/kg) and nivolumab (360 mg).

For nivolumab and ipilimumab combination therapy, nivolumab will be administered at 360 mg IV Q3W, and ipilimumab will be administered at 1 mg/kg IV Q3W for the first 2 doses and then every 6 weeks for the 3rd and 4th doses, followed by nivolumab 480 mg IV Q4W until PD or intolerable toxicity. After receipt of the first dose of ipilimumab, the Investigator may determine (based on clinical symptoms) the number of future doses of ipilimumab the participant will receive, for a maximum of 4 doses. Participants who stop ipilimumab dosing early due to toxicities, may start nivolumab maintenance (ie, 4 doses [12 weeks] of nivolumab following the first dose).

Advanced Prostate Cancer (Amendment 3): Single-agent nivolumab administered at 360 mg intravenously (IV) every 3 weeks (Q3W). Participants who continue to show clinical benefit after the first disease assessment will receive nivolumab 480 mg IV every 4 weeks (Q4W) until progressive disease (PD) or intolerable toxicity. At PD, participants will be allowed to crossover to the advanced prostate cancer CD8 low arm of ipilimumab (3 mg/kg) and nivolumab (1 mg/kg).

For nivolumab and ipilimumab combination therapy, CD8 low arm will be randomly allocated into 1 of 2 cohorts, using different doses of ipilimumab administered in 6-week cycles. Prostate Cohort A will receive nivolumab 1 mg/kg Q3W and ipilimumab 3 mg/kg every 6 weeks (Q6W) for 2 cycles, then nivolumab maintenance 480 mg Q4W until PD or intolerable toxicity. Prostate Cohort B will receive nivolumab 1 mg/kg Q3W and ipilimumab 5 mg/kg Q6W for 2 cycles, then nivolumab maintenance 480 mg Q4W until PD or intolerable toxicity.

Safety and Futility Monitoring:

Monitoring will be ongoing during enrollment in each group to assess the stopping rules as defined in the protocol (see Section 9.3.1). There is no independent Data Safety Monitoring Committee.

DocuSign

Certificate Of Completion

Envelope Id: C63A1A965C51452399A01C9280CFD826 — Status: Completed

Subject: Please DocuSign: PICI0025 SAP v1.0 10Jan2022.docx

Source Envelope:

Document Pages: 22	Signatures: 2	Envelope Originator:
Certificate Pages: 5	Initials: 0	Shikha Gautam
AutoNav: Enabled		1 Letterman Drive
EnvelopeId Stamping: Disabled		San Francisco, CA 94129
Time Zone: (UTC-08:00) Pacific Time (US & Canada)		sgautam@parkerici.org
		IP Address: 162.228.89.160

Record Tracking

Status: Original<br>1/10/2022 11:21:33 AM	Holder: Shikha Gautam<br>sgautam@parkerici.org	Location: DocuSign

Signer Events	Signature	Timestamp
Christopher Cabanski<br>ccabanski@parkerici.org<br>Senior Director, Biometrics<br>Parker Institute for Cancer Immunotherapy<br>Security Level: Email, Account Authentication (Required)	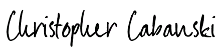<br>Signature Adoption: Pre-selected Style<br>Signature ID:<br>73CCABA7-49D1-4634-B5D6-314D83F02F85<br>Using IP Address: 24.6.146.143<br><br>With Signing Authentication via DocuSign password<br>With Signing Reasons (on each tab):<br>I approve this document	Sent: 1/10/2022 11:26:56 AM<br>Viewed: 1/10/2022 11:35:51 AM<br>Signed: 1/10/2022 11:36:04 AM

Electronic Record and Signature Disclosure:
Not Offered via DocuSign

Justin Fairchild<br>jfairchild@parkerici.org<br>VP, Clinical Development<br>Parker Institute for Cancer Immunotherapy<br>Security Level: Email, Account Authentication (Required)	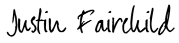<br>Signature Adoption: Pre-selected Style<br>Signature ID:<br>949277DE-9B08-4F27-973F-0D2BBB82C4F0<br>Using IP Address: 24.61.69.97<br><br>With Signing Authentication via DocuSign password<br>With Signing Reasons (on each tab):<br>I approve this document	Sent: 1/10/2022 11:26:56 AM<br>Viewed: 1/10/2022 1:27:43 PM<br>Signed: 1/10/2022 1:28:05 PM

Electronic Record and Signature Disclosure:
Accepted: 5/15/2019 9:13:01 AM
ID: b1573b3d-089b-41c9-8b03-72c198ebb255

In Person Signer Events	Signature	Timestamp

Editor Delivery Events	Status	Timestamp

Agent Delivery Events	Status	Timestamp

Intermediary Delivery Events	Status	Timestamp

Certified Delivery Events	Status	Timestamp

Carbon Copy Events	Status	Timestamp

Witness Events	Signature	Timestamp

Notary Events	Signature	Timestamp

Envelope Summary Events	Status	Timestamps
Envelope Sent	Hashed/Encrypted	1/10/2022 11:26:56 AM
Certified Delivered	Security Checked	1/10/2022 1:27:43 PM
Signing Complete	Security Checked	1/10/2022 1:28:05 PM
Completed	Security Checked	1/10/2022 1:28:05 PM

Payment Events	Status	Timestamps

Electronic Record and Signature Disclosure

ELECTRONIC RECORD AND SIGNATURE DISCLOSURE

From time to time, Parker Institute for Cancer Immunotherapy (we, us or Company) may be required by law to provide to you certain written notices or disclosures. Described below are the terms and conditions for providing to you such notices and disclosures electronically through the DocuSign system. Please read the information below carefully and thoroughly, and if you can access this information electronically to your satisfaction and agree to this Electronic Record and Signature Disclosure (ERSD), please confirm your agreement by selecting the check-box next to 'I agree to use electronic records and signatures' before clicking 'CONTINUE' within the DocuSign system.

Getting paper copies

At any time, you may request from us a paper copy of any record provided or made available electronically to you by us. You will have the ability to download and print documents we send to you through the DocuSign system during and immediately after the signing session and, if you elect to create a DocuSign account, you may access the documents for a limited period of time (usually 30 days) after such documents are first sent to you. After such time, if you wish for us to send you paper copies of any such documents from our office to you, you will be charged a $0.00 per-page fee. You may request delivery of such paper copies from us by following the procedure described below.

Withdrawing your consent

If you decide to receive notices and disclosures from us electronically, you may at any time change your mind and tell us that thereafter you want to receive required notices and disclosures only in paper format. How you must inform us of your decision to receive future notices and disclosure in paper format and withdraw your consent to receive notices and disclosures electronically is described below.

Consequences of changing your mind

If you elect to receive required notices and disclosures only in paper format, it will slow the speed at which we can complete certain steps in transactions with you and delivering services to you because we will need first to send the required notices or disclosures to you in paper format, and then wait until we receive back from you your acknowledgment of your receipt of such paper notices or disclosures. Further, you will no longer be able to use the DocuSign system to receive required notices and consents electronically from us or to sign electronically documents from us.

All notices and disclosures will be sent to you electronically

Unless you tell us otherwise in accordance with the procedures described herein, we will provide electronically to you through the DocuSign system all required notices, disclosures, authorizations, acknowledgements, and other documents that are required to be provided or made available to you during the course of our relationship with you. To reduce the chance of you inadvertently not receiving any notice or disclosure, we prefer to provide all of the required notices and disclosures to you by the same method and to the same address that you have given us. Thus, you can receive all the disclosures and notices electronically or in paper format through the paper mail delivery system. If you do not agree with this process, please let us know as described below. Please also see the paragraph immediately above that describes the consequences of your electing not to receive delivery of the notices and disclosures electronically from us.

How to contact Parker Institute for Cancer Immunotherapy:

You may contact us to let us know of your changes as to how we may contact you electronically, to request paper copies of certain information from us, and to withdraw your prior consent to receive notices and disclosures electronically as follows:
To contact us by email send messages to: pici-support@parkerici.org

To advise Parker Institute for Cancer Immunotherapy of your new email address

To let us know of a change in your email address where we should send notices and disclosures electronically to you, you must send an email message to us at pici-support@parkerici.org and in the body of such request you must state: your previous email address, your new email address. We do not require any other information from you to change your email address.

If you created a DocuSign account, you may update it with your new email address through your account preferences.

To request paper copies from Parker Institute for Cancer Immunotherapy

To request delivery from us of paper copies of the notices and disclosures previously provided by us to you electronically, you must send us an email to sbolisetty@parkerici.org and in the body of such request you must state your email address, full name, mailing address, and telephone number. We will bill you for any fees at that time, if any.

To withdraw your consent with Parker Institute for Cancer Immunotherapy

To inform us that you no longer wish to receive future notices and disclosures in electronic format you may:

i. decline to sign a document from within your signing session, and on the subsequent page, select the check-box indicating you wish to withdraw your consent, or you may;

ii. send us an email to pici-support@parkerici.org and in the body of such request you must state your email, full name, mailing address, and telephone number. We do not need any other information from you to withdraw consent.. The consequences of your withdrawing consent for online documents will be that transactions may take a longer time to process..

Required hardware and software

The minimum system requirements for using the DocuSign system may change over time. The current system requirements are found here: https://support.docusign.com/guides/signer-guide-signing-system-requirements.

Acknowledging your access and consent to receive and sign documents electronically

To confirm to us that you can access this information electronically, which will be similar to other electronic notices and disclosures that we will provide to you, please confirm that you have read this ERSD, and (i) that you are able to print on paper or electronically save this ERSD for your future reference and access; or (ii) that you are able to email this ERSD to an email address where you will be able to print on paper or save it for your future reference and access. Further, if you consent to receiving notices and disclosures exclusively in electronic format as described herein, then select the check-box next to ‘I agree to use electronic records and signatures’ before clicking ‘CONTINUE’ within the DocuSign system.

By selecting the check-box next to ‘I agree to use electronic records and signatures’, you confirm that:

- You can access and read this Electronic Record and Signature Disclosure; and
- You can print on paper this Electronic Record and Signature Disclosure, or save or send this Electronic Record and Disclosure to a location where you can print it, for future reference and access; and
- Until or unless you notify Parker Institute for Cancer Immunotherapy as described above, you consent to receive exclusively through electronic means all notices, disclosures, authorizations, acknowledgements, and other documents that are required to be provided or made available to you by Parker Institute for Cancer Immunotherapy during the course of your relationship with Parker Institute for Cancer Immunotherapy.
